# A strategic approach to [6,6]-bicyclic lactones: application towards the CD fragment of DHβE

**DOI:** 10.3762/bjoc.13.98

**Published:** 2017-05-22

**Authors:** Tue Heesgaard Jepsen, Emil Glibstrup, François Crestey, Anders A Jensen, Jesper Langgaard Kristensen

**Affiliations:** 1 Department of Drug Design and Pharmacology, Faculty of Health and Medical Sciences, University of Copenhagen, Universitetsparken 2, 2100 Copenhagen, Denmark

**Keywords:** DhβE, Mizoroki–Heck cross-coupling reaction, 6π-electrocyclization, [6,6]-bicyclic lactone, vinyl halide

## Abstract

We report an effective synthetic protocol to access [6,6]-bicyclic lactone moieties through a regio- and stereoselective intramolecular Mizoroki–Heck cross-coupling reaction followed by a 6π-electrocyclization. This method enabled the first synthesis of the elusive CD fragment of the *Erythrina* alkaloid DHβE. Preliminary pharmacological evaluations support the notion that the key pharmacophores of DHβE are located in the A and B rings.

## Introduction

The neuronal nicotinic acetylcholine receptors (nAChRs) have been extensively investigated as potential drug targets for a diverse array of central nervous system (CNS) related medical conditions such as Alzheimer’s and Parkinson’s disease, depression, ADHD, pain relief, nicotine addiction and drug abuse [1]. Dihydro-β-erythroidine (DHβE) is a member of the family of tetracyclic *Erythrina* alkaloids which were isolated from *Erythrina* species in the end of the 19th century; the majority of this family possesses neuromuscular blocking effects [2]. DHβE is one of the most potent nAChR antagonists of this class and displays prominent selectivity for the α4β2 subtype (*K*_i_ = 0.82 µM in a [^3^H]epibatidine binding assay) [3]. So far, DHβE represents one of the simplest reference competitive antagonists for the α4β2 nAChR subtype. Although its chemical structure has been known for several decades [4–5], no comprehensive SAR study of DHβE can be found in the literature, except from our previous deconstruction approach [3] and a few degradation studies [6–7].

**Figure 1:**
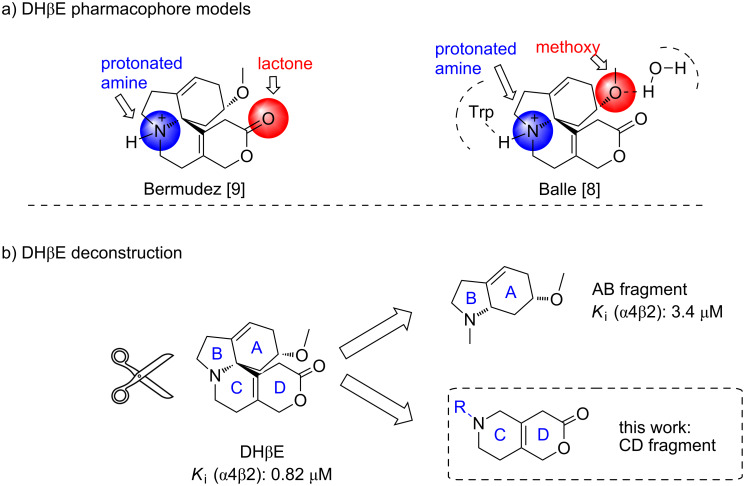
DHβE and related structures. The *K*_i_ values of the compounds at the rat α4β2 nAChR subtype determined in a [^3^H]cytisine binding assay are given [3].

Balle and co-workers recently published an X-ray structure of the acetylcholine binding protein (AChBP) in complex with DHβE [8] and based on this structure, two key pharmacophores of DHβE were proposed as shown in Figure 1a: the methoxy group in the A ring which interacts via hydrogen bonding with a tightly bound water molecule in the protein and the protonated amine which forms hydrogen bonds directly with the backbone of the protein. Thus, this structure indicates that the key pharmacophores are located in the A ring and the B–C ring, which contrasts a mutational-computational study by Bermudez and co-workers suggesting that the lactone carbonyl is a hydrogen bond acceptor and hence locating the key pharmacophores in the C and D rings [9]. In order to weigh these hypotheses against one another, we have recently published a SAR on the deconstructed AB fragments of DHβE [3]; while reducing the molecular size and complexity considerably, we were able to retain the affinity, α4β2-subtype selectivity and competitive antagonist properties in the direct AB-analogue of DHβE (see Figure 1b). On the other hand, we have previously deconstructed a selection of aromatic erythrinanes, and interestingly the SAR showed CD fragments with retained affinity, subtype specificity, and competitive antagonist property relative to the parent natural product [10]. Inspired by these results, we embarked on the synthesis of the CD fragment of DHβE.

Previously, the aromatic CD fragments were straightforwardly synthesized due to the advantageous reactivity of the aromatic D ring [10]. However, the syntheses of the lactonic *Erythrina* alkaloids are more complex [11–12] as illustrated by more than 150 total syntheses reported for aromatic erythrinanes [2] whereas only four total syntheses of lactonic erythrinanes have been published so far [13–16]. Hence, for the DHβE-based CD fragments, we faced a significantly more challenging synthesis due to the complex nature of the [6,6]-bicyclic lactone moiety for which synthetic procedures are extremely scarce. Herein, we wish to provide different strategies used to synthesize the CD fragment of DHβE as a general and simple method for the construction of [6,6]-bicyclic lactones which includes a stereoselective synthesis of vinyl halides, a regio- and stereoselective intramolecular Mizoroki–Heck cross-coupling reaction and a 6π-electrocyclization as key steps.

## Results and Discussion

### First strategy with Ts and Cbz protecting groups

**Scheme 1:**
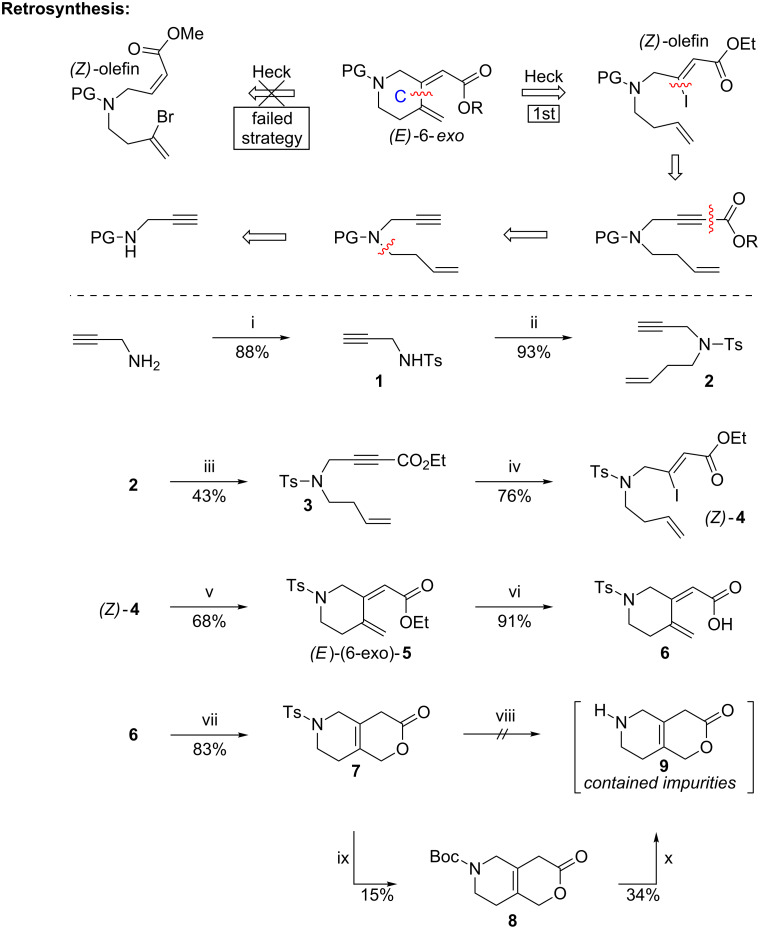
First strategy towards the CD fragment (Ts-strategy). i) TsCl, TEA, DCM, 0 °C. ii) NaH, DMF, 0 °C, then 3-buten-1-yl methanesulfonate, 100 °C. iii) *n*-BuLi, ClCO_2_Et, THF, −78 °C. iv) LiI, AcOH, 70 °C. v) PdCl_2_(PPh_3_)_2_, Ag_2_CO_3_, THF, rt. vi) LiOH, H_2_O, THF, rt. vii) BHT (cat.), PhMe, reflux. viii) SmI_2_. ix) Sodium naphthalenide, DME, −78 °C, then Boc_2_O, rt. x) TFA, DCM, rt. For more details regarding the failed strategy, see Supporting Information File 1.

As depicted in Scheme 1, our first strategy featured a late stage installation of the lactonic D ring by a 6π-electrocyclization and formation of the C ring by an intramolecular Mizoroki–Heck cross-coupling reaction from a *Z*-configured olefin which would be crucial to the stereochemical outcome of the Heck cyclization event. We envisioned a *Z*-stereoselective synthesis of a vinyl halide [17] which should secure the desired *E*-stereochemistry for the Mizoroki–Heck coupling. We were aware that we would perhaps face a greater challenge in terms of generating the desired 6-membered exocyclic product rather than the undesired 7-membered endocyclic product [18]. Starting from commercially available propargylamine, tosylated compound **1** was prepared in 88% yield followed by its alkylation using mesylated homoallylic alcohol to provide **2** in 93% yield. The incorporation of the ester functionality proved to be more troublesome than anticipated based on literature precedence [19]. After extensive optimization, it was found that deprotonation of the terminal alkyne with *n*-BuLi and subsequent quenching with ethyl chloroformate provided the desired ester **3** in 43% yield. The subsequent stereoselective addition of lithium iodide [17] provided the *Z*-vinyl iodide **4** in 76% yield with no trace of the undesired *E*-isomer.

After extensive screening (see Supporting Information File 1 for more details), the key Mizoroki–Heck cross-coupling reaction was performed at room temperature providing the desired (*E*)-(6-exo)-**5** product in 68% yield using PdCl_2_(PPh_3_)_2_ as catalyst and Ag_2_CO_3_ as base in THF. Hydrolysis of the ester (*E*)-(6-exo)-**5** with LiOH was achieved in 91% yield to furnish carboxylic acid **6**. The subsequent 6π-electrocyclization performed in refluxing toluene in the presence of a catalytic amount of 2,6-di-*tert*-butyl-4-methylphenol (BHT) led to lactone **7** in 83% yield. The removal of the Ts-protecting group was initially attempted with SmI_2_ but unfortunately this reaction proceeded without a trace of the desired lactone **9**. Recently Szostak et al. have shown that 6-membered lactones undergo reduction with SmI_2_ [20] which may explain this result. However, upon treatment with sodium napthalenide lactone **7** was fully converted but all attempts to isolate and purify the deprotected amine **9** were unsuccessful. Therefore, the detosylated amine was reprotected in situ with Boc_2_O to provide **8** in 15% yield, anticipating a clean cleavage of the Boc group to circumvent subsequent purification of the free amine. Indeed, Boc removal in the presence of TFA in dichloromethane (DCM) was successful and provided, after purification by preparative TLC, the volatile derivative **9** in 34% (6.5 mg) yield. Although this material contained some impurities (see Supporting Information File 1 for copies of ^1^H and ^13^C spectra), it was of sufficient purity for preliminary pharmacological evaluations.

**Scheme 2:**
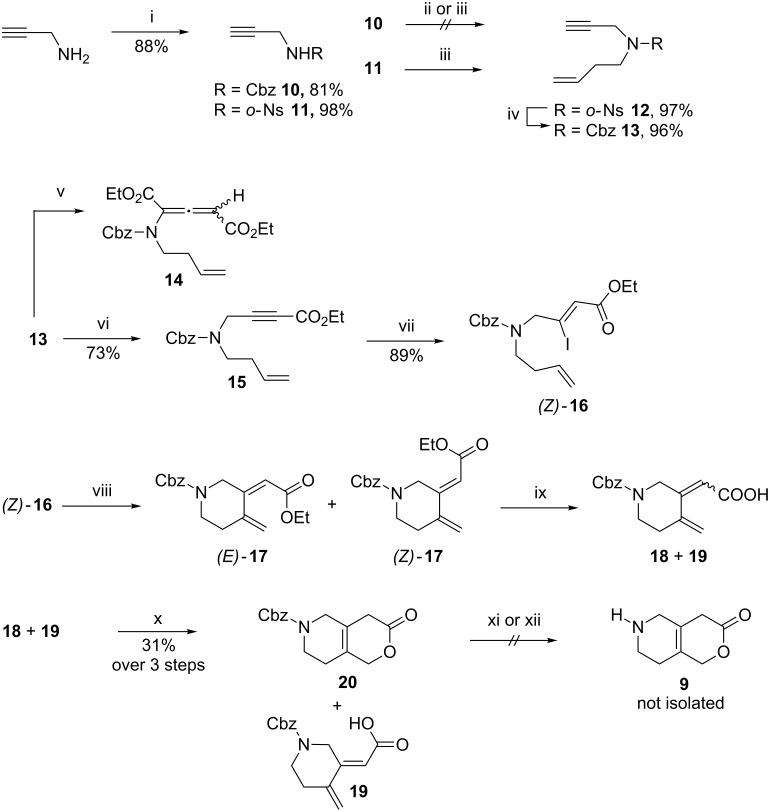
First strategy towards the CD fragment (Cbz-strategy). i) R-Cl, TEA, CH_2_Cl_2_, 0 °C. ii) NaH, DMF, 0 °C, then 3-buten-1-yl methanesulfonate, 100 °C. iii) 4-Bromobut-1-ene, K_2_CO_3_, DMF, 60 °C. iv) a) *p*-MePhSH, aq NaOH, CH_3_CN, 50 °C; b) CbzCl, TEA, DCM, 0 °C to rt. v) LiHMDS, THF, −78 °C, 1 h, then ClCO_2_Et, −78 °C to rt. vi) LiHMDS, THF, −78 °C, 2 h, then ClCO_2_Et, −78 °C, 1 h. vii) LiI, AcOH, 70 °C. viii) PdCl_2_(PPh_3_)_2_, Ag_2_CO_3_, THF, 60 °C. ix) LiOH, H_2_O, THF, rt. x) BHT (cat.), PhMe, reflux. xi) Pd/C, H_2_, EtOAc or MeOH or AcOH. xii) Pd(OH)_2_, H_2_, EtOAc or MeOH or AcOH.

However, since our aim was to develop a strategy for the late stage N-functionalization applicable for a medicinal chemistry SAR approach, the route described above was unsatisfactory. Therefore we turned our attention to an alternative protecting group, namely the Cbz group (see Scheme 2). We envisioned that the reductive removal of this protective group would allow for an easier isolation of the volatile final product. Unfortunately, an alkylation of the Cbz-protected propargylamine **10** was unsuccessful. To circumvent this issue an initial protection of the amine **10** using *o*-NsCl (*o*-nosyl chloride) led to nosyl derivative **11** in 98% yield. The subsequent alkylation of **11** with homoallyl bromide provided the *o*-Ns-protected derivative **12** in 97% yield. A straightforward deprotection–reprotection procedure then furnished the Cbz-protected species **13** in 96% yield. Unfortunately, the attempted functionalization by treatment with *n*-BuLi and quenching with ethyl chloroformate led to a complex mixture of products; the same trend was also observed when switching to LDA as the base. When LiHMDS was applied in this reaction a double addition of ethyl formate took place giving rise to allenamide **14** as the major product [21]. After extensive optimization, the optimal results were obtained by treatment of **13** with 1 equiv of LiHMDS at −78 °C for 2 h before the addition of 5 equiv of ethyl chloroformate. The resulting mixture was then left at –78 °C for 1 h before quenching at low temperature, which proved vital to avoid the allenamide formation. The careful control of the reaction conditions in this way provided the desired product **15** in 73% yield and the subsequent *Z*-stereoselective addition of LiI proceeded without problems to give (*Z*)-**16** in 89% yield. Unfortunately, performing the intramolecular Mizoroki–Heck cyclization at room temperature using the optimized reaction conditions described above, led to the formation of byproducts. However, carrying out the cross-coupling reaction at 60 °C afforded the desired product (*E*)-**17** along with byproduct (*Z*)-**17** (which was believed to be the (*Z*)-6-*exo* isomer). Hydrolysis of this mixture was achieved with LiOH giving carboxylic acids **18** and **19** and running the reaction at a 0.01–0.02 molar scale was important for it to go to completion overnight. Higher concentrations of the starting material seemed to slow down the reaction, which also caused hydrolysis of the Cbz group to some extent.

The final ring-closure to the CD-ring fragment **20** was successfully achieved with 31% yield over 3 steps. Only the desired (*E*)-6-*exo* **18** isomer reacted, leaving the (*Z*)-*exo* isomer **19** uncyclized, as anticipated. Inspired by the successful Cbz deprotection of a very similar system [22], the final hydrogenolysis of the Cbz protecting group with H_2_ and either Pd/C or Pd(OH)_2_ (up to 50 mol % catalyst loading) in EtOAc, MeOH or AcOH was attempted, but no trace of the desired lactone **9** was observed. A control experiment with the addition of tosyl chloride after the hydrogenolysis to form the known tosyl-protected intermediate **7** indicated no signs of product and therefore the CBz strategy was also abandoned.

### Second strategy without protecting group

**Scheme 3:**
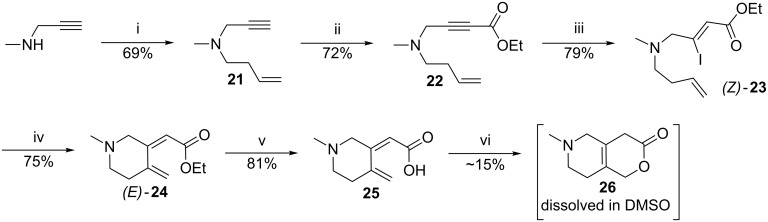
Second strategy towards the CD fragment. i) 4-Bromobut-1-ene, K_2_CO_3_, acetone, 70 °C. ii) *n*-BuLi, THF, −78 °C, 1 h, then ClCO_2_Et, −78 °C, 1 h. iii) LiI, AcOH, 50 °C. iv) PdCl_2_(PPh_3_)_2_, Ag_2_CO_3_, THF, 50 °C. v) LiOH, H_2_O, THF, rt. vi) HFIP, 80 °C.

Since the protective group removal was problematic we decided to preinstall the desired N-substituent and thereby avoid any N-protective group (see Scheme 3). The second strategy started with an N-alkylation of the commercially available *N*-methyl propargylamine with homoallyl bromide providing the tertiary amine **21** in 69% yield which was used without further purification. The subsequent treatment with *n*-BuLi and trapping with ethyl chloroformate provided alkyne **22** in 72% yield which reacted with LiI in acetic acid furnishing the desired (*Z*)-vinyl iodide **23** in 79% isolated yield. A concise screening of the Mizoroki–Heck reaction conditions (which involved Jeffery conditions [23], Pd_2_dba_3_/Xantphos [24] or Fu’s salt [25], and PdCl_2_(PPh_3_)_2_ in combination with either K_2_CO_3_ or Ag_2_CO_3_) revealed that the optimized conditions from the first strategy still performed quite well for this new approach. Indeed, when using a combination of PdCl_2_(PPh_3_)_2_ as catalyst and Ag_2_CO_3_ as base to secure cationic Heck conditions, no trace of the undesired 6-*endo* product was observed. However, when the reaction mixture was heated at 60 °C elimination of HI to form alkyne **22** was observed as the major product and at 25 °C and 40 °C conversion was very slow. Interestingly, at 50 °C the iodide **23** was selectively converted into the desired (*E*)-6-*exo* product **24** in 75% isolated yield with only 13% formation of the elimination product **22**, and no trace of the undesired (*Z*)-6-*exo* product. The subsequent hydrolysis of the ethyl ester **24** at room temperature smoothly provided carboxylic acid **25** in 81% yield with retention of the *E*-configuration, whereas isomerization of the olefin occurred at higher temperatures.

Subjecting **25** to the standard conditions for the final 6π-electrocyclization (toluene, THF, or DME) at 80–150 °C either led to no conversion of the starting material or complete decomposition at elevated temperatures. We speculated that the low solubility of carboxylic acid **25** in these apolar solvents was the reason. However, addition of small amounts of MeOH in order to increase polarity was detrimental and led to decomposition upon heating. Since the desired cyclization could be also approached as an intramolecular Michael addition we attempted to mediate the reaction by applying basic (K_2_CO_3_, LiOH) and acidic (TFA, BF_3_) conditions but unfortunately this caused complete decomposition of the starting material upon heating. However, the cyclization was successful by heating **25** at 80 °C in the slightly acidic hexafluoro-2-propanol (HFIP). HFIP seemed exactly acidic enough to mediate the reaction without causing decomposition. Although **25** was fully converted into a single product the targeted compound **26** proved to be extremely difficult to isolate and purify. The compound as the free amine was very volatile and co-evaporated with different solvents (HFIP, MeOH, DCM) and was found to be unstable on silica. Thus, all attempts to purify the material through column chromatography or preparative TLC led to decomposition of the material. Also an attempted isolation of the amine as its hydrochloride or trifluoroacetate failed. Finally, the [6,6]-bicyclic lactone **26** was isolated as a 0.31 mM solution in DMSO after purification by preparative LCMS in a very modest yield of 15% (see Supporting Information File 1 for more details regarding the preparation of the DMSO solution and the calculation of its concentration for the pharmacological evaluation). Nonetheless, this route provided us with sufficient material to perform a preliminary pharmacological evaluation. Even though the purification of our target molecule proved difficult, the chemistry of the final 6π-electrocyclization was very effective with full conversion of the precursor **25** into the desired [6,6]-bicyclic lactone **26**.

### Pharmacological evaluation

**Figure 2:**
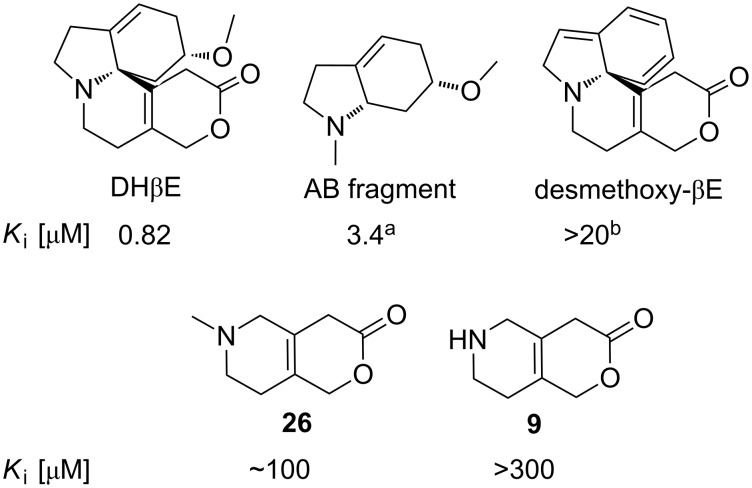
The binding affinities of compounds **9** and **26** at the rat α4β2 nAChR. a) The AB fragment was evaluated in a [^3^H]-epibatidine binding assay [3]. b) Desmethoxy-βE was evaluated by Wildeboer in a [^3^H]-cytisine binding assay [27].

The binding properties of two synthesized CD fragments (compounds **9** and **26**) were characterized in a [^3^H]-epibatidine binding assay using membranes from HEK293 cells stably expressing the rat heteromeric nAChR subtypes α4β2, α4β4 and α3β4 as previously described [10,26]. The pharmacological evaluation of the CD fragments revealed that the absence of the methoxy group in the A ring was detrimental to the affinity for the α4β2 nAChR subtype as depicted in Figure 2 (see Supporting Information File 1 for assay details). This contrasts our recent results obtained for the AB fragments which retained the affinity comparable to the parent natural product (DHβE), and indicates that the Balle’s pharmacophore model is the best description of the key binding interactions of DHβE to α4β2, provided that the AB and CD fragments bind similar to DHβE in the active site. This is further supported by Wildeboer’s study from 2005 who reported a much lower affinity of desmethoxy-βE compared to the parent DHβE [27].

## Conclusion

In summary, we have successfully developed a strategy to construct the CD ring system of DHβE and to efficiently access the synthetically challenging [6,6]-bicyclic lactone fragment in general through an expedient regio- and stereoselective Mizoroki–Heck cyclization approach. This method enabled the synthesis of the elusive and volatile CD fragments ([6,6]-bicyclic lactones **9** and **26**) of the *Erythrina* alkaloid DHβE. This allowed the investigation of their pharmacological effects lacking the AB ring substructure present in the parent natural product. Even though the CD fragment proved exceedingly difficult to handle and to purify our results indicate that the absence of the methoxy group on the A ring is detrimental to the affinity. Further studies concerning the construction of new designed [6,6]-bicyclic lactones and the deconstruction of the DHβE scaffold are currently underway in our laboratory.

## Supporting Information

File 1 Full experimental details, synthetic procedures, optimization study, failed strategies and pharmacological characterization of the compounds.

File 2 Copies of NMR spectra.

## References

[1] Arneric S P, Holladay M, Williams M (2007). Biochem Pharmacol.

[2] Reimann E (2007). Synthesis Pathways to Erythrina Alkaloids and Erythrina Type Compounds. Progress in the Chemistry of Organic Natural Products.

[3] Jepsen T H, Jensen A A, Lund M H, Glibstrup E, Kristensen J L (2014). ACS Med Chem Lett.

[4] Weinstock J, Boekelheide V (1953). J Am Chem Soc.

[5] Boekelheide V, Agnello E (1951). J Am Chem Soc.

[6] Hider R C, Walkinshaw M D, Saenger W (1986). Eur J Med Chem.

[7] Megirian D, Leary D E, Slater I H (1955). J Pharmacol Exp Ther.

[8] Shahsavar A, Kastrup J S, Nielsen E Ø, Kristensen J L, Gajhede M, Balle T (2012). PLoS One.

[9] Iturriaga-Vásquez P, Carbone A, García-Beltrán O, Livingstone P D, Biggin P C, Cassels B K, Wonnacott S, Zapata-Torres G, Bermudez I (2010). Mol Pharmacol.

[10] Crestey F, Jensen A A, Borch M, Andreasen J T, Andersen J, Balle T, Kristensen J L (2013). J Med Chem.

[11] Tsuda Y, Sano T (1996). The Alkaloids: Chemistry and Pharmacology.

[12] Tsuda Y, Hosoi S, Mohri K, Isobe K (1992). Chem Pharm Bull.

[13] He Y, Funk R L (2006). Org Lett.

[14] Funk R L, Belmar J (2012). Tetrahedron Lett.

[15] Kawasaki T, Onoda N, Watanabe H, Kitahara T (2001). Tetrahedron Lett.

[16] Fukumoto H, Takahashi K, Ishihara J, Hatakeyama S (2006). Angew Chem, Int Ed.

[17] Romero D L, Manninen P R, Han F, Romero A G (1999). J Org Chem.

[18] Jepsen T H, Larsen M, Jørgensen M, Nielsen M B (2012). Synlett.

[19] Wang Z, Lin X, Luck R L, Gibbons G, Fang S (2009). Tetrahedron.

[20] Szostak M, Spain M, Procter D J (2014). J Am Chem Soc.

[21] Paul A, Einsiedel J, Waibel R, Heinemann F W, Meyer K, Gmeiner P (2009). Tetrahedron.

[22] Macdonald S J F, Montana J G, Buckley D M, Dowle M D (1998). Synlett.

[23] Jeffery T (1996). Tetrahedron.

[24] Barder T E, Walker S D, Martinelli J R, Buchwald S L (2005). J Am Chem Soc.

[25] Littke A F, Dai C, Fu G C (2000). J Am Chem Soc.

[26] Jensen A A, Mikkelsen I, Frølund B, Bräuner-Osborne H, Falch E, Krogsgaard-Larsen P (2003). Mol Pharmacol.

[27] Wildeboer K M (2005). Structure activity relationships of nicotine analogs and Erythrina alkaloids on the alpha 4 beta 2 nicotinic acetylcholine receptor.

