# Supplementary material for: A strategic approach to [6,6]-bicyclic lactones: application towards the CD fragment of DHβE
Source: Beilstein J Org Chem. 2017 May 22;13:988–94. doi: 10.3762/bjoc.13.98 (PMC5480346; doi:10.3762/bjoc.13.98)
Supplement: File 1 — Full experimental details, synthetic procedures, optimization study, failed strategies and pharmacological characterization of the compounds. [file Beilstein_J_Org_Chem-13-988-s001.pdf]

# Supporting Information File 1

# for

# A strategic approach to [6,6]-bicyclic lactones: application towards the CD fragment of DHβE

Tue Heesgaard Jepsen§, Emil Glibstrup§, François Crestey, Anders A. Jensen and Jesper Langgaard Kristensen*

Address: Department of Drug Design and Pharmacology, Faculty of Health and Medical Sciences, University of Copenhagen, Universitetsparken 2, 2100 Copenhagen, Denmark

Email: Jesper Langgaard Kristensen - jesper.kristensen@sund.ku.dk

*Corresponding author

§Equally contributing authors

## Full experimental details, synthetic procedures, optimization study, failed strategies and pharmacological characterization of the compounds

**Table of contents:**

1. Material and methods ........ S2
2. Failed strategies ........ S4
3. Optimization studies of the key Mizoroki–Heck reaction ........ S8
4. Pharmacological data of [6,6]-bicyclic lactones **9** and **26** ........ S10
5. Synthetic procedures ........ S11

## 1. Material and methods

All reactions were carried out under a nitrogen atmosphere or argon atmosphere in reactions involving anhydrous conditions. Reagents were purchased at the highest commercial quality and used without further purification. Solvents were HPLC-grade quality. Dry THF, DMF, and DCM were obtained by passing commercially available pre-dried, oxygen-free formulations through activated alumina columns or dried with activated molecular sieves. Reactions were monitored by either LC–MS (ESI) or GC–MS (EI) and TLC carried out on 0.25 mm silica gel plates using UV light visualization, and either ninhydrin or potassium permanganate staining as an indicator. Silica gel (60 Å, particle size 15–40 µm) was used for short plugs and for dry column vacuum chromatography[1] (DCVC) and silica gel (60 Å, particle size 35–70 µm) was used for flash chromatography. Melting points were measured using a Büchi B-540 apparatus. NMR spectra were recorded on 300, 400, and 600 MHz instruments and calibrated using residual solvent signals as an internal reference. Coupling constants (*J*) are given in hertz (Hz). Multiplicities of $^{1}H$ NMR signals are reported as follows: s, singlet; d, doublet; dd, doublet of doublets; dt, doublet of triplets; ddt, doublet of doublets of triplets; t, triplet; q: quartet; m: multiplet; br, broad signal. High-resolution mass spectra (HRMS) were recorded on a SolariX XR 7 TESI/MALDI-FT-ICR-MS instrument and data were handled with the DataAnalysis Version 4.0SP 4. ESI samples were dissolved in acetonitrile or mixtures of acetonitrile and water containing 0.1% HCOOH. MALDI samples were dissolved in dichloromethane and applied with dithranol as matrix. Preparative TLC was performed on 20 × 20 cm plates (Analtech, Uniplate, Silica Gel GF, with UV absorption at 254 nm) and was eluted in a preparative TLC glass-container using 200 mL of solvent mixture. Bands were scraped off and extracted with the polar solvent. Preparative LC–MS was performed on an Agilent 1100 series HPLC system coupled with a Hewlett-Packard 1100 series MSD (ESP+) with an Ascentis Express RP-amide column (100 × 4.6 mm; 2.7 µm particle size). A gradient with eluent A (0.5% TFA (v/v) in water) containing 0% of eluent B (0.5% TFA (v/v) in acetonitrile) rising linearly to 100% of B during $t$ = 0–20 min at a flow of 1.5 mL/min

[1] Pedersen, D. S.; Rosenbohm, C. *Synthesis*, **2001**, 2431.

was used for the purification of compound **26** (injection volume 500 µL). UV absorption was measured at 254 nm and 210 nm.

The following abbreviations are used: CBz-Cl: benzyl chloroformate; *p*-NsCl: 4-nitrobenzenesulfonyl chloride; *o*-NsCl: 2-nitrobenzenesulfonyl chloride; PE: petroleum ether; DCM: dichloromethane; TEA: triethylamine; TFA: trifluoroacetic acid; DME: dimethoxyethane; DMF: dimethylformamide; EtOAc: ethyl acetate; DMP: Dess–Martin periodinane; LiHMDS: lithium bis(trimethylsilyl)amide; KHMDS: potassium bis(trimethylsilyl)amide; THF: tetrahydrofuran; $Et_2O$: diethyl ether; TsCl: tosyl chloride; AcOH: acetic acid; BHT: 2,6-di-*tert*-butyl-4-methylphenol; HFIP: hexafluoro-2-propanol; MW: microwave; SPhos: 2-dicyclohexylphosphino-2',6'-dimethoxybiphenyl; DMSO: dimethylsulfoxide; nAChRs: neuronal nicotinic acetylcholine receptors.

## 2. Failed strategies

Our first initial strategy (Scheme S1) involving a stereoselective Mizoroki–Heck cross-coupling reaction followed by an electrocyclization was inspired by Funk's previous total synthesis of β-erythroidine.[2] Starting from the known 3-bromobut-3-en-1-amine,[3] we introduced the *p*-Ns (*p*-nosyl)-protecting group in 97% yield (compound **27**) followed by alkylation of the sulfonamide with methyl bromoacetate to furnish **28** in 97% yield. The ester derivative was transformed into its corresponding aldehyde **29** by reduction with $LiBH_4$ followed by oxidation with Dess–Martin periodinane (DMP) in 65% yield over two steps. A previous work[4] indicated that the stereochemistry of the key Mizoroki–Heck cyclization product would be guided by the stereochemistry of the α,β-unsaturated ester **30**. Hence, a *Z*-selective olefination of **29** was crucial to the strategy. Unfortunately, the Still–Gennari olefination[5] gave an unsatisfying 2:3 ratio of regioisomers (*Z*)-**30** and **31**. Changing to standard Wittig conditions[6] using methyl (triphenylphosphoranylidene)acetate afforded the α,β-unsaturated ester **30** in 96% yield as a 1:9 mixture of *Z*/*E* isomers. A brief screening of conditions for the intramolecular Mizoroki–Heck-coupling reaction showed that (*E*)-**30** could be fully converted and did provide the coupled product (*Z*)-**32** along with numerous by-products using of $K_2CO_3$ (3 equiv) as base, $Pd(PPh_3)_4$ (10 mol %) as catalyst in acetonitrile under microwave (MW) conditions within 1 h at 120 °C. When the exact same conditions were applied to (*Z*)-**30**, a mixture of co-eluting isomers (*E*)-**32** and (*Z*)-**32** was obtained in an unsatisfying 1:2 ratio and again flanked by numerous by-products and hence, this initial strategy was discarded. The unsatisfactory results from the Mizoroki–Heck cross-coupling reactions and the lack of stereoselectivity in the olefination step made us reconsider how to access the precursor for the electrocyclization.

---

[2] He, Y.; Funk, R. L. *Org. Lett.*, **2006**, 8, 3689.

[3] Padwa, A.; Waterson, A. G. *Tetrahedron*, **2000**, *56*, 10159.

[4] Nagasawa, K.; Ishihara, H.; Zako, Y.; Shimizu, I. *J. Org. Chem.*, **1993**, *58*, 2523.

[5] Still, W. C.; Gennari, C. *Tetrahedron Lett.*, **1983**, *24*, 4405.

[6] a) Wittig, G.; Schöllkopf, U. *Chem. Ber.*, **1954**, *87*, 1318; b) Maryanoff, B. E.; Reitz, A. B. *Chem. Rev.*, **1989**, *89*, 863.

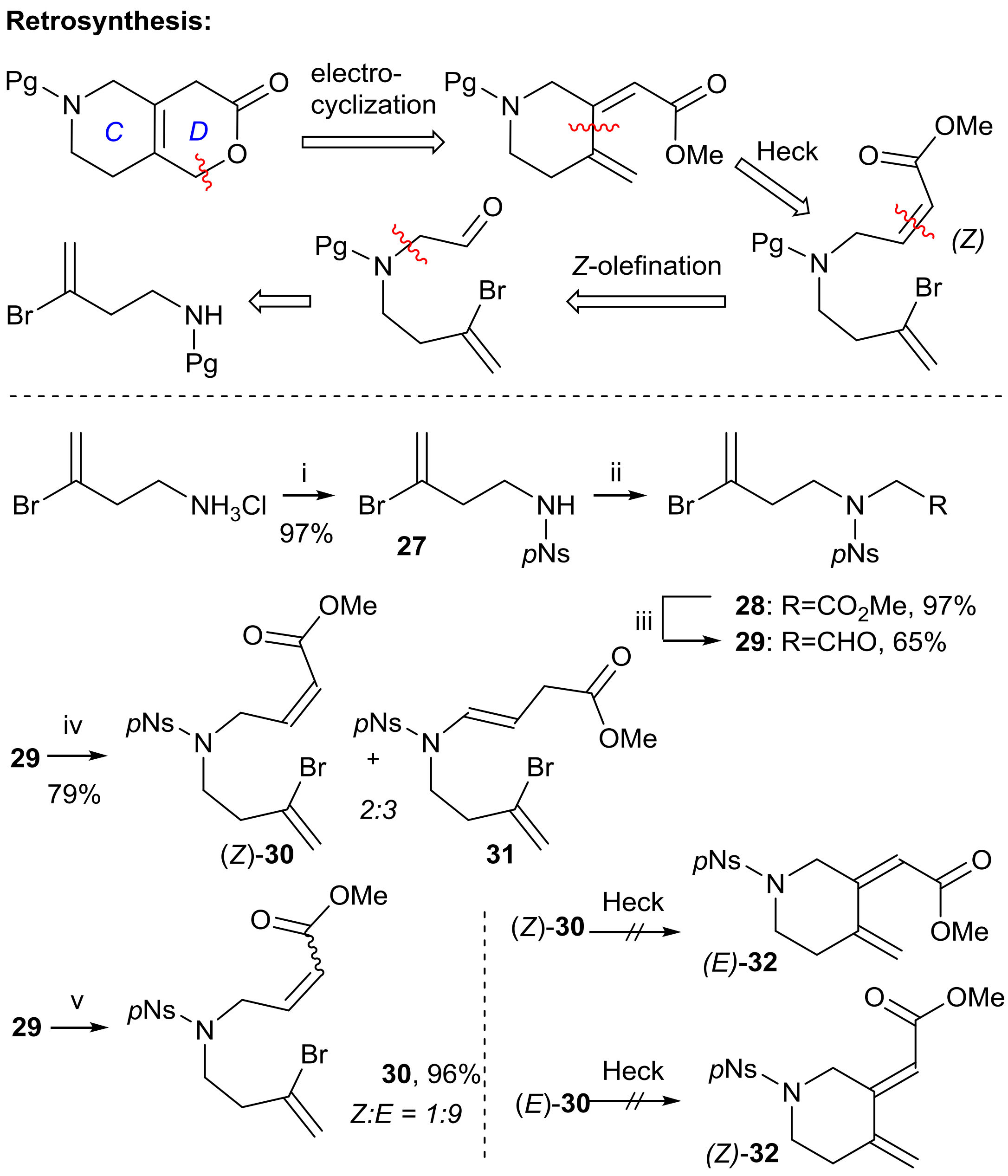


**Scheme S1:** Strategy towards the CD fragment based on Funk's total synthesis. i) *p*-NsCl, TEA, DCM, 0 °C to rt. ii) Methyl bromoacetate, $Cs_2CO_3$, $CH_3CN$, rt. iii) a) $LiBH_4$, THF, 0 °C to rt. b) DMP, DCM, rt to reflux. iv) Bis(2,2,2-trifluoroethyl)(methoxycarbonylmethyl)phosphonate, 18-crown-6, KHMDS, THF, −78 °C then **4**, 0 °C to rt. v) Methyl (triphenylphosphoranylidene)acetate, THF, 0 °C.

In parallel, another strategy based on a pyridine scaffold with an early or a late stage reduction–alkylation procedure to provide the desired CD fragment was investigated as

illustrated in Scheme S2 and Scheme S3. 3-Chloropyridine-4-carbaldehyde was quantitatively reduced to its corresponding alcohol **33**. A subsequent Suzuki cross-coupling reaction using (*E*)-2-(2-ethoxyvinyl)-4,4,5,5-tetramethyl-1,3,2-dioxaborolane[7] provided the disubstituted pyridine **34** which was treated with TFA furnishing acetal **35**. However, all attempts failed to obtain the lactone **36** from the corresponding acetal even though it was only a simple adjustment in the oxidation state.

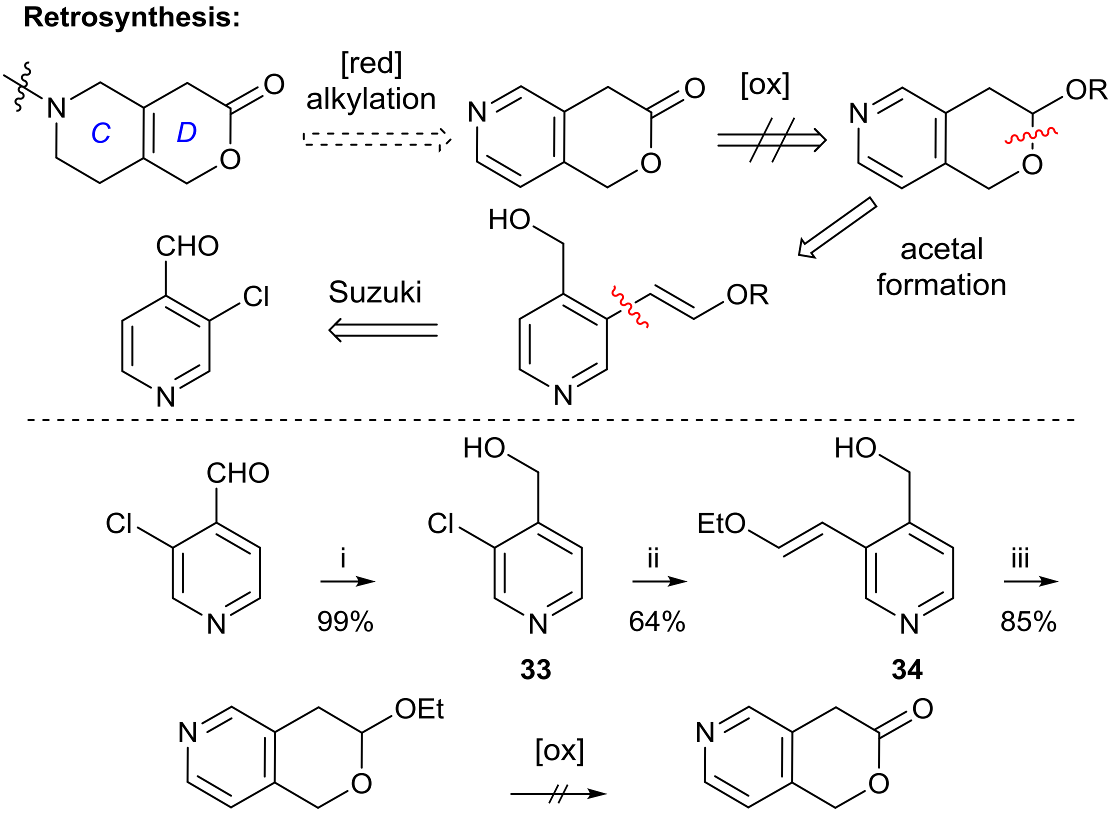


35 36

**Scheme S2:** Strategy towards the CD fragment based on a pyridine scaffold. i) $NaBH_4$, MeOH, 0 °C to rt. ii) (*E*)-2-(2-Ethoxyvinyl)-4,4,5,5-tetramethyl-1,3,2-dioxaborolane, $K_3PO_4$, SPhos, $Pd(OAc)_2$, $CH_3CN$, $H_2O$, MW, 120 °C. iii) TFA, DCM, rt.

In addition, performing the alkylation–reduction procedure on an appropriately decorated pyridine ring **38** led to its corresponding piperidine derivative **39**. Unfortunately, all

---

[7] Crestey, F.; Hooyberghs, G.; Kristensen, J. L. *Tetrahedron*, **2012**, *68*, 1417.

attempts to synthesize hemiacetal **40** failed and this pyridine-based strategy was also discarded.

**Retrosynthesis:**

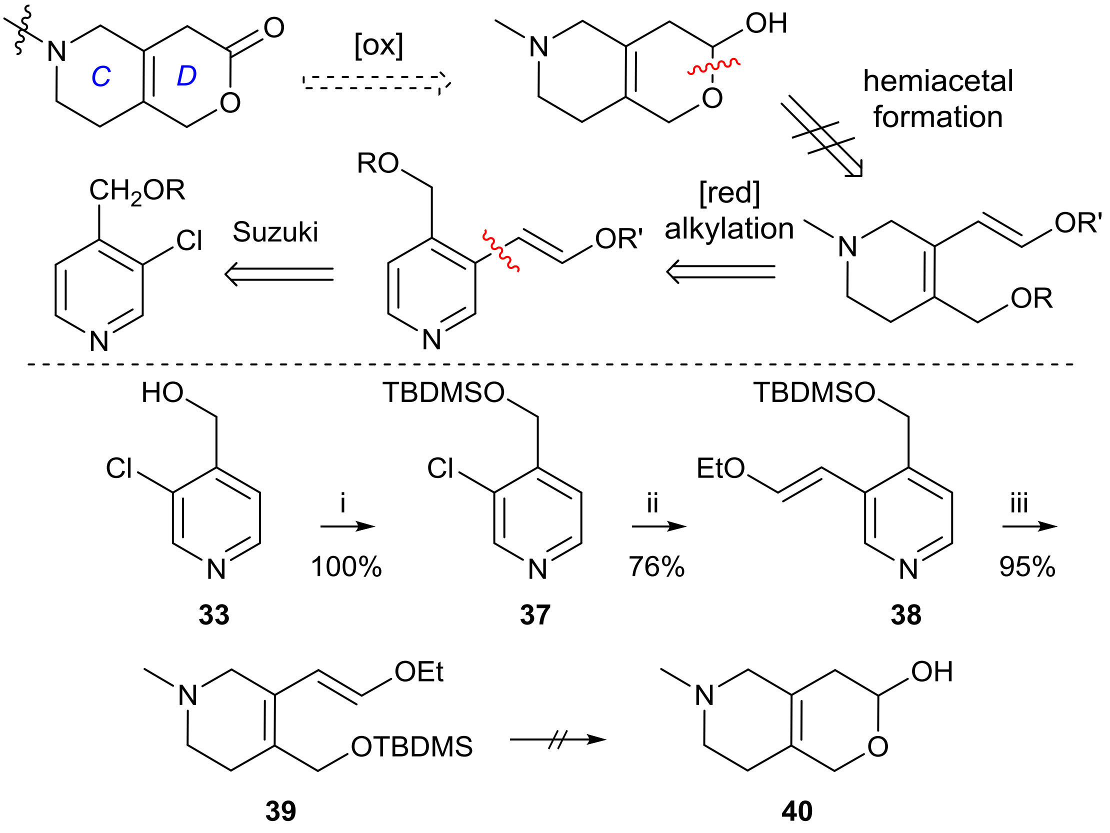


**Scheme S3:** Strategy towards the CD fragment based on a pyridine scaffold. i) TBDMSiCl, imidazole, DMF, rt. ii) (*E*)-2-(2-Ethoxyvinyl)-4,4,5,5-tetramethyl-1,3,2-dioxaborolane, $K_3PO_4$, SPhos, $Pd(OAc)_2$, $CH_3CN$, $H_2O$, MW, 85 °C. iii) a) MeI, DCM, rt. b) $NaBH_4$, MeOH, 0 °C to rt. TBDMS: *tert*-Butyldimethylsilyl.

## 3. Optimization studies of the key Mizoroki–Heck reaction

**Table S1.** Mizoroki–Heck optimization.

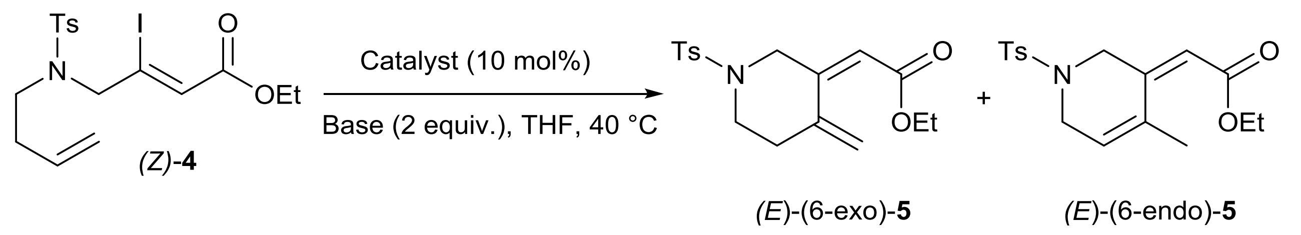


| Entry | Catalyst | Base | Time | 6-*exo* | 6-*endo* |
|---|---|---|---|---|---|
| 1[b] | $PdCl_2(PPh_3)_2$ | $K_2CO_3$ | 5 days | 16% | 13% |
| 2[b] | $PdCl_2(PPh_3)_2$ | TEA | 5 days | 42% | 38% |
| 3 | $PdCl_2(PPh_3)_2$ | $Ag_2CO_3$ | 3 hours | 51% | 40% |
| 4[b] | $PdCl_2$(dppe) | $K_2CO_3$ | 5 days | 13% | 20% |
| 5[b] | $PdCl_2$(dppe) | TEA | 5 days | 9% | 2% |
| 6 | $PdCl_2$(dppe) | $Ag_2CO_3$ | 3 hours | 55% | 31% |
| 7[c] | $PdCl_2(PPh_3)_2$ | $Ag_2CO_3$ | 15 hours | 68% | 21% |
| 8[c] | $PdCl_2$(dppe) | $Ag_2CO_3$ | 48 hours | 62% | 21% |

[a]Yield determined by HPLC. [b]More solvent and catalyst were added after 2 days. [c]Rt instead of 40 °C.

The key Mizoroki–Heck coupling was subjected to a screening of the reaction conditions (Table S1), including a silver(I) base to direct the reaction towards a cationic pathway.[8] Using THF as solvent and a temperature of 40 °C, two types of palladium complexes were investigated, i.e., the monodentate $PdCl_2(PPh_3)_2$ and the bidentate $PdCl_2$(dppe). These palladium complexes were combined with three different bases (TEA, $K_2CO_3$ and $Ag_2CO_3$). $PdCl_2(PPh_3)_2$ in combination with $K_2CO_3$ as base, entry 1, required long reaction

[8] Ozawa, F.; Kubo, A.; Hayashi, T. *J. Am. Chem. Soc.*, **1991**, *113*, 1417.

times and along with the product several other minor by-products. Using $PdCl_2(PPh_3)_2$ and TEA, entry 2, the reaction showed higher and cleaner conversion, but still long reaction times. With $Ag_2CO_3$ and $PdCl_2(PPh_3)_2$, entry 3, the reaction was complete after 3 h providing a 5:4 ratio of the (*E*)-(6-exo)-**5** and the undesired (*E*)-(6-endo)-**5** product, with no trace of the 7-*endo* product. As observed in entries 4 and 5, $PdCl_2$(dppe) was ineffective, whereas $Ag_2CO_3$ in entry 6, the reaction was again complete after 3 h and showed results similar to $PdCl_2(PPh_3)_2$ in entry 3. Upon isolation of the regioisomers, the desired 6-exo product seemed to increase during workup. To improve the exo:endo ratio the temperature was decreased to rt and repeated using $Ag_2CO_3$ and the two ligands, entries 7 and 8 respectively.

Performing the reaction in the presence of $PdCl_2(PPh_3)_2$ showed full conversion after 15 h and an improved 3:1 ratio favouring the desired 6-exo product whereas using $PdCl_2$(dppe) required longer reaction time (48 h) to reach a similar ratio. With the optimized conditions (entry 7) in hand, the Mizoroki–Heck coupling could be performed at rt providing the desired (6-exo)-**5** product in 55% isolated yield on gram scale.

## 4. Pharmacological data of [6,6]-bicyclic lactones 9 and 26

**Binding assay:** The compounds were characterized in a [$^3$H]epibatidine binding assay using membranes from HEK293 cells stably expressing the rat heteromeric nAChR subtypes α4β2, α4β4 and α3β4. The $K_i$ values of the compounds are given in μM (with p$K_i$ ± S.E.M in parentheses). The data are the means of 3 individual experiments.

**Functional assay:** Functional characteristics of the analogues at a HEK293T cell line stably expressing the mouse α4β2 nAChR and at a HEK293 cell line stably expressing the rat α3β4 nAChR in the FLIPR Membrane Potential Blue assay. $EC_{50}$ and $IC_{50}$ values are given in μM with p$EC_{50}$ ± S.E.M. and p$IC_{50}$ ± S.E.M. values in parentheses. The data are the means of 3 individual experiments performed in duplicate. (*S*)-Nicotine ($EC_{70}$–$EC_{80}$ for the respective receptors) was used as agonist in the antagonist experiments.

| | **Binding $K_i$ (μM)** | | | **Functional $IC_{50}$ (μM)** | |
|---|---|---|---|---|---|
| **Compound** | **α4β2** | **α4β4** | **α3β4** | **α4β2** | **α3β4** |
| DHβE | 0.82 | ~100 | ~100 | 1.2 | ~100 |
| **26** | ~100 [4.0] [a] | ~300 [~3.5] [a] | >300 [<3.5] | >300 [<3.5] | >300 [<3.5] |
| **9** | >300 [<3.5] | >300 [<3.5] | >300 [<3.5] | >300 [<3.5] | >300 [<3.5] |

[a]Since a complete concentration–inhibition curve could not be obtained for the compound in the concentration ranges tested the $IC_{50}$ value given is an estimate.

## 5 Synthetic procedures

**1:** To a cooled solution of propargylamine (5.85 mL, 91.4 mmol) in dry DCM (200 mL) at 0 °C was added TEA (30 mL). After 5 min, TsCl (16.59 g, 87.00 mmol) was added portionwise over 10 min and the reaction mixture was stirred at rt for 1 h then diluted with $H_2O$ (200 mL) and washed with aqueous 6 M HCl. The organic phase was washed with brine (2 × 200 mL). The aqueous phase was extracted with DCM (100 mL) and the combined organic phases were dried over $Na_2SO_4$, filtered and concentrated in vacuo to provide a colorless oil. The crude material was dissolved in $Et_2O$ (100 mL) and washed twice with aqueous 6 M HCl (50 mL) followed by $H_2O$ (50 mL). Evaporation of the organic phase in vacuo yielded the product **1** (15.76 g, 87%) as a white solid. The reaction was repeated once on the same scale with a yield of 88%. Spectral data was in accordance with previous characterizations.[9] $^1$H NMR (600 MHz, $CDCl_3$) *δ* 7.77 (d, *J* = 8.2 Hz, 2H), 7.31 (d, *J* = 8.2 Hz, 2H), 4.87 (br s, 1H), 3.82 (s, 2H), 2.42 (s, 3H), 2.10 (t, *J* = 2.5 Hz, 1H); $^{13}$C NMR (151 MHz, $CDCl_3$) *δ* 144.0, 136.6, 129.8, 127.5, 78.1, 73.1, 33.0, 21.7; LC-MS (*m/z*) = 210.0 $[M+H]^+$, 232.0 $[M+Na]^+$.

**2:** To a cooled solution of **1** (5.02 g, 24.0 mmol) in dry DMF (50 mL) at 0 °C was added NaH (1.27 g, 60%, 31.7 mmol) portionwise. After $H_2$ evolution ceased, dry DMF (25 mL) was added prior to the addition of a solution of 3-buten-1-yl methanesulfonate[9] (4.32 g, 28.7 mmol) in dry DMF (20 mL). The reaction mixture was heated at 100 °C for 6 h, cooled to rt and diluted with DCM (100 mL), washed with $H_2O$ (2 × 100 mL) and the aqueous phase was extracted with DCM (2 × 50 mL). The combined organic phases were washed once more with $H_2O$ (50 mL) then dried over $Na_2SO_4$. The solvent was evaporated in vacuo and the crude material was dissolved in $Et_2O$ (50 mL), washed with $H_2O$ (2 × 100 mL), the aqueous phase was extracted with $Et_2O$ (50 mL) and the combined organic phases were evaporated in vacuo. The crude was purified by flash column chromatography using DCM as eluent to yield the product **2** (5.10 g, 81%) as a pale yellow oil. The reaction was repeated twice up to a scale of 18.7 g of product **2** with an average yield of 93%. Spectral data was in accordance with previous characterizations.[9] $^1$H NMR

[9] Kavanagh, Y.; Chaney, C. M.; Muldoon, J.; Evans, P. *J. Org. Chem.*, **2008**, *73*, 8601.

(600 MHz, $CDCl_3$) $\delta$ 7.72 (d, $J$ = 8.1 Hz, 2H), 7.28 (d, $J$ = 8.1 Hz, 2H), 5.76 (ddt, $J$ = 17.0 Hz and $J$ = 10.2 Hz and $J$ = 6.9 Hz, 1H), 5.04–5.13 (m, 2H), 4.14 (d, $J$ = 2.5 Hz, 2H), 3.26 (t, $J$ = 7.5 Hz, 2H), 2.41 (s, 3H), 2.34 (dt, $J$ = 7.5 Hz and $J$ = 6.9 Hz, 2H), 2.03 (t, $J$ = 2.5 Hz, 1H); $^{13}$C NMR (151 MHz, $CDCl_3$) $\delta$ 143.6, 136.0, 134.6, 129.6, 127.8, 117.4, 76.7, 73.9, 45.8, 36.5, 32.3, 21.7; LC-MS (*m*/*z*) = 264.0 $[M+H]^+$, 286.0 $[M+Na]^+$.

**3:** To a cooled solution of **2** (2.73 g, 10.4 mmol) in dry THF (50 mL) at −78 °C was added *n*-BuLi (9.2 mL, 18 mmol, 2 M in hexanes) dropwise turning the reaction mixture black. After 30 min at this temperature, $ClCO_2Et$ (1.80 mL, 18.9 mmol) was added and the reaction mixture was stirred at −78 °C for 6 h. The reaction was quenched with saturated aqueous $NH_4Cl$ (75 mL) and diluted with DCM (75 mL). The organic phase was washed with $H_2O$ (2 × 50 mL) and the aqueous phase was extracted with DCM (50 mL). The combined organic phases were dried over $Na_2SO_4$, filtered and concentrated in vacuo. The crude material was purified by flash column chromatography using DCM as eluent to afford product **3** (1.59 g, 47%) as a pale yellow oil. The reaction was repeated 3 times up to a scale of 4.47 g of product **3** and an average yield of 43%. $^1$H NMR (600 MHz, $CDCl_3$) $\delta$ 7.72 (d, $J$ = 8.2 Hz, 2H), 7.30 (d, $J$ = 8.2 Hz, 2H), 5.75 (ddt, $J$ = 17.0, 10.2, 6.9 Hz, 1H), 5.04–5.13 (m, 2H), 4.24 (s, 2H), 4.15 (q, $J$ = 7.1 Hz, 2H), 3.26 (t, $J$ = 7.5 Hz, 2H), 2.40 (s, 3H), 2.33 (dt, $J$ = 7.5, 6.9 Hz, 2H), 1.26 (t, $J$ = 7.0 Hz, 3H); $^{13}$C NMR (151 MHz, $CDCl_3$) $\delta$ 152.6, 144.0, 135.5, 134.3, 129.8, 127.7, 117.6, 80.2, 77.3, 62.1, 46.3, 36.6, 32.3, 21.6, 14.1; HRMS (MALDI): calcd. for $C_{17}H_{22}NO_4S$ $[M+H]^+$ 336.1264; found 336.1262.

**(*Z*)-4:** To a solution of **3** (552 mg, 1.65 mmol) in AcOH (2 mL) was added LiI (245 mg, 1.83 mmol) in AcOH (1 mL) at rt. The reaction mixture was heated at 70 °C for 2 h then LiI (100 mg, 0.75 mmol) was added. After four additional hours, the reaction mixture was cooled to rt and toluene was added to azeotropically remove AcOH. The crude material was filtered through a short plug of silica using $Et_2O$ as eluent to provide product (*Z*)-**4** (746 mg, 98%) as a yellowish solid. The reaction was repeated twice up to a scale of 5.25 g of product and an average yield of 76%. $^1$H NMR (600 MHz, $CDCl_3$) $\delta$ 7.69 (d, $J$ = 8.2 Hz, 2H), 7.30 (d, $J$ = 8.2 Hz, 2H), 6.66 (t, $J$ = 1.6 Hz, 1H), 5.58–5.66 (m, 1H), 4.98–5.03 (m, 2H), 4.20 (q, $J$ = 7.2 Hz, 2H), 4.17 (d, $J$ = 1.6 Hz, 2H), 3.20–3.24 (m, 2H), 2.41 (s, 3H), 2.21 (dt, $J$ = 7.8 Hz and $J$ = 7.3 Hz, 2H), 1.28 (t, $J$ = 7.2 Hz, 3H); $^{13}$C NMR (151 MHz, $CDCl_3$) $\delta$ 164.2, 143.9, 136.5, 134.2, 130.0, 127.3, 125.8, 117.7, 114.7, 61.8, 60.9, 48.6,

32.7, 21.6, 14.3; HRMS (MALDI): calcd. for $C_{17}H_{23}INO_4S$ $[M+H]^+$ 464.0387;found 464.0376.

**(*E*)-(6-exo)-5:** To a solution of (*Z*)-**4** (1.15 g, 2.48 mmol) in dry THF (10 mL) were successively added $PdCl_2(PPh_3)_2$ (175 mg, 249 µmol) and $Ag_2CO_3$ (1.39 mg, 5.02 mmol) under argon atmosphere at rt. After 15 h the reaction mixture was evaporated and the crude material was purified by flash column chromatography using DCM as eluent to lead to product (*E*)-(6-exo)-**5** (565 mg, 68%) as a white solid. The reaction was repeated twice up to a scale of 1.94 g of product (*E*)-(6-exo)-**5** with an average yield of 55%. $^1$H NMR (600 MHz, $CDCl_3$) $\delta$ 7.64 (d, $J$ = 8.2 Hz, 2H), 7.30 (d, $J$ = 8.2 Hz, 2H), 5.75 (s, 1H), 5.05 (s, 2H), 4.11 (q, $J$ = 7.1 Hz, 2H), 3.63 (s, 2H), 3.20 (t, $J$ = 5.8 Hz, 2H), 2.39–2.41 (m, 5H), 1.23 (t, $J$ = 7.1 Hz, 3H); $^{13}$C NMR (151 MHz, $CDCl_3$) $\delta$ 165.7, 147.2, 144.0, 140.1, 133.5, 129.9, 127.8, 117.7, 115.7, 60.4, 54.25, 47.4, 34.4, 21.6, 14.1; HRMS (MALDI): calcd. for $C_{17}H_{22}NO_4S$ $[M+H]^+$ 336.1264; found 336.1263.

**6:** To a solution of (*E*)-(6-exo)-**5** (512 mg, 1.53 mmol) in dry THF (10 mL) was added LiOH (1 M in $H_2O$, 10 mL) at rt. The reaction mixture was stirred at this temperature for 20 h then volatiles were removed under reduced pressure. After the addition of aqueous 1 M HCl, the aqueous layer was extracted with DCM and EtOAc. The organic layers were dried over $Na_2SO_4$, filtered and concentrated in vacuo to furnish product **6** (444 mg, 95%) as a white fluffy solid which over time collapsed to a sticky semi-solid. The reaction was repeated twice up to a scale of 1.67 g of product **6** with an average yield of 91%. $^1$H NMR (600 MHz, $CDCl_3$) $\delta$ 7.65 (d, $J$ = 8.2 Hz, 2H), 7.32 (d, $J$ = 8.2 Hz, 2H), 5.75 (s, 1H), 5.09 (d, $J$ = 4.6 Hz, 2H), 3.66 (s, 2H), 3.22 (t, $J$ = 5.8 Hz, 2H), 2.41–2.43 (m, 5H); $^{13}$C NMR (151 MHz, $CDCl_3$) $\delta$ 170.6, 150.4, 144.1, 139.7, 133.5, 129.9, 127.8, 116.6, 116.5, 54.5, 47.5, 34.5, 21.7; HRMS (MALDI): calcd. for $C_{15}H_{17}NNaO_4S$ $[M+Na]^+$ 330.0770; found 330.0770.

**7:** To a solution of **6** (268 mg, 0.87 mmol) in toluene (20 mL) was added BHT (ca. 10 mg) at rt under nitrogen atmosphere. The reaction mixture was heated under reflux conditions for 41 h then cooled to rt. The crude material was filtered through a plug of silica using toluene then EtOAc as eluent. The fractions eluted with EtOAc were concentrated under reduced pressure to provide product **7** (237 mg, 88%) as a milky sticky oil/semi-solid. The reaction was repeated 3 times up to a scale of 1.34 g of product **7** with an average yield of 83%. $^1$H NMR (600 MHz, $CDCl_3$) $\delta$ 7.67 (d, $J$ = 8.2 Hz, 2H), 7.33 (d, $J$ = 8.2 Hz, 2H), 4.68–

4.71 (m, 2H), 3.46 (s, 2H), 3.25 (t, *J* = 5.5 Hz, 2H), 2.93 (s, 2H), 2.43 (s, 3H), 2.14 (dt, *J* = 5.5, 3.2 Hz, 2H); $^{13}$C NMR (151 MHz, $CDCl_3$) *δ* 167.8, 144.2, 133.1, 130.0, 127.7, 123.5, 120.9, 71.0, 46.3, 42.5, 31.5, 25.1, 21.7; HRMS (MALDI): calcd. for $C_{15}H_{18}NO_4S$ $[M+H]^+$ 308.0951; found 308.0955.

**8:** A freshly prepared solution of sodium napthalenide (11 mL, 0.5 M in DME, 5.5 mmol, obtained by dissolving Na (345 mg, 15.0 mmol) and napthalene (2.12 g, 16.5 mmol) in dry DME (30 mL) and stirred under argon atmosphere for 2 h as a dark green solution) was added dropwise to a solution of **7** (502 mg, 1.63 mmol) in dry DME (25 mL) at −78 °C. After 1 h at this temperature, the reaction mixture was quenched with $H_2O$ (0.7 mL) and allowed to warm to rt. $Boc_2O$ (642 mg, 2.94 mmol) was added and the reaction mixture was stirred at rt for 1 h. Volatiles were removed under reduced pressure then the crude material was purified by DCVC using a gradient elution (heptane followed by EtOAc–heptane 1:10 to 2:1) to afford the desired product **8** (138 mg, 34%) as an oil. The reaction was repeated twice with an average yield of 15%. $^1$H NMR (600 MHz, $CDCl_3$) *δ* 4.74 (br s, 2H), 3.77 (br s, 2H), 3.55 (t, *J* = 5.6 Hz, 2H), 2.96 (br s, 2H), 2.03 (br s, 2H), 1.45 (s, 9H); $^{13}$C NMR (151 MHz, $CDCl_3$) broadened/doublet signals were observed *δ* 168.4, 154.7, 123.6, 123.4, 122.4, 121.9, 80.3, 71.3, 45.3, 44.7, 40.7, 39.4, 31.6, 28.5 (3C), 24.7 (3C); LC-MS (*m*/*z*) = 276.2 ($M+Na^+$), 198.1 ($M\text{-}tBu+H^+$), 154.1 ($M\text{-}Boc+H^+$).

**9:** To a solution of **8** (31.7 mg, 0.13 mmol) in dry DCM (4 mL) was slowly added TFA (0.2 mL) at 0 °C. After 2 h at this temperature, the solvents were removed under reduced pressure and the crude TFA salt was purified by preparative TLC (500 microns) using MeOH ($NH_3$)–DCM (1:4) as eluent. Because the product **9** was extremely volatile, careful evaporation of solvents provided nearly pure product **9** (6.5 mg, 34%) as an oil. $^1$H NMR (600 MHz, $CD_3OD$) *δ* 4.08 (s, 2H), 3.45 (br s, 2H), 3.10 (t, *J* = 5.9 Hz, 2H), 2.99 (s, 2H), 2.35 (t, *J* = 5.9 Hz, 2H), 1.89 (s, 1H); $^{13}$C NMR (151 MHz, $CD_3OD$) *δ* 178.7, 132.3, 128.1, 62.2, 47.7, 43.0, 40.5, 26.5; HRMS (ESI): calcd. for $C_8H_{12}NO_2$ $[M+H]^+$ 154.0863; found 154.0863.

**10:** To a cooled solution of propargylamine (5.00 mL, 78.1 mmol) at 0 °C in dry DCM (200 mL) were successively added Cbz-Cl (12.5 mL, 87.6 mmol) and TEA (16.5 mL, 118 mmol). The reaction mixture was allowed to warm to rt overnight then the organic layer was washed with aqueous 1 M HCl (200 mL) and brine (150 mL). The aqueous layer

was extracted with DCM (100 mL) and the combined organic phases were dried over $Na_2SO_4$, filtered and concentrated under reduced pressure. The crude material was filtered through a short plug of silica using EtOAc–heptane 1:1 as eluent to provide the pure product **10** (11.91 g, 81%) as a light yellow solid with spectral data in accordance with previous characterizations.[10] $^1$H NMR (600 MHz, $CDCl_3$) $\delta$ 7.30–7.39 (m, 5H), 5.13 (s, 2H), 4.94 (br s, 1H), 3.94–4.03 (m, 2H), 2.24 (t, $J$ = 2.5 Hz, 1H); $^{13}$C NMR (151 MHz, $CDCl_3$) $\delta$ 156.0, 136.3, 128.7, 128.4, 128.3, 79.7, 71.8, 67.3, 31.0; LC–MS (*m*/*z*) = 190.1 $[M+H]^+$.

**11:** To a cooled solution of propargylamine (5.00 mL, 78.1 mmol) at 0 °C in dry DCM (200 mL) was added *o*-NsCl (8.65 g, 39.0 mmol) in one portion. After 30 min, a second portion of *o*-NsCl (8.65 g, 39.0 mmol) was added. After 2 additional hours the reaction was quenched with aqueous 1 M HCl (100 mL) and the organic phase was washed with $H_2O$ (100 mL), dried over $Na_2SO_4$, filtered and concentrated under reduced pressure to lead to product **11** (18.33 g, 98%) as a light brown solid with spectral data in accordance with previous characterizations.[11] $^1$H NMR (600 MHz, $CDCl_3$) $\delta$ 8.18–8.21 (m, 1H), 7.89–7.93 (m, 1H), 7.74–7.77 (m, 2H), 5.70 (br t, $J$ = 6.3 Hz, 1H), 4.01 (dd, $J$ = 6.3, 2.5 Hz, 2H), 1.98 (t, $J$ = 2.5 Hz, 1H); $^{13}$C NMR (151 MHz, $CDCl_3$) $\delta$ 148.1, 134.1, 134.0, 133.1, 131.7, 125.6, 77.5, 73.4, 33.5; LC–MS (*m*/*z*) = 186.0 ($M-C_3H_4N$).

**12:** To a solution of **11** (10.06 g, 41.6 mmol) in dry DMF (100 mL) were successively added $K_2CO_3$ (17.27 g, 125.0 mmol) and 4-bromo-but-1-ene (6.17 g, 45.7 mmol) at rt. The reaction mixture was heated at 60 °C for 16 h. A second portion of 4-bromo-but-1-ene (3.12 g, 23.1 mmol) was added and the reaction mixture was stirred at 60 °C for another hour before cooling to rt. The reaction was quenched with $H_2O$ (200 mL) and extracted with $Et_2O$ (3 × 100 mL). The combined organic phases were washed with $H_2O$ (100 mL), dried over $Na_2SO_4$, filtered and concentrated under reduced pressure to afford product **12** (12.2 g, 99%) as a dark oil. The reaction was repeated twice more with an average yield of 97%. $^1$H NMR (600 MHz, $CDCl_3$) $\delta$ 7.98–8.00 (m, 1H), 7.65–7.72 (m, 2H), 7.59–7.61 (m, 1H), 5.66–5.73 (m, 1H), 4.98–5.09 (m, 2H), 4.18 (d, $J$ = 2.5 Hz, 2H), 3.46 (t, $J$ = 7.2 Hz,

[10] Shang, S.; Zhang-Negrerie, D.; Du, Y.; Zhao, K. *Angew. Chem. Int. Ed.*, **2014**, *53*, 6216.

[11] Deschamps, N. M.; Elitzin, V. I.; Liu, B.; Mitchell, M. B.; Sharp, M. J.; Tabet, E. A. *J. Org. Chem.*, **2010**, *76*, 712.

2H), 2.33 (q, $J$ = 7.2 Hz, 2H), 2.19 (t, $J$ = 2.5 Hz, 1H); $^{13}$C NMR (151 MHz, $CDCl_3$) $\delta$ 148.2, 134.1, 133.9, 132.6, 131.8, 130.7, 124.2, 117.6, 76.7, 74.1, 46.2, 36.5, 32.0; HRMS (MALDI): calcd. for $C_{13}H_{15}N_2O_4S$ $[M+H]^+$ 295.0747; found 295.0748.

**13:** To a cooled solution of *p*-thiocresol (8.83 g, 71.1 mmol) in acetonitrile (50 mL) at 0 °C was added aqueous NaOH (7.5 mL, 35%, 28 mmol). After a few minutes a solution of **12** (8.16 g, 27.7 mmol) in MeCN (75 mL) was slowly added and the reaction mixture was heated at 50 °C for 1.5 h then cooled to rt and carefully acidified with aqueous 1 M HCl until pH 1. The aqueous layer was extracted with DCM (100 mL) then carefully basified with aqueous 1 M NaOH until pH 12. The resulting aqueous layer was extracted with DCM (3 × 50 mL), and the resulting organic layer was washed with brine, dried over $Na_2SO_4$, filtered and cooled to 0 °C prior to the addition of TEA (10 mL, 72 mmol). After 5 min CbzCl (6.4 mL, 45 mmol) was slowly added and the reaction mixture was allowed to warm to rt overnight. The solvents were removed under reduced pressure to give a crude material which was purified by DCVC using a gradient elution (heptane followed by EtOAc–heptane 1:5 to 1:1) to yield pure product **13** (6.25 g, 93%) as a greenish yellow oil. The reaction was repeated up to a scale of 10 g of product **13** with an average yield of 96%. $^{1}$H NMR (600 MHz, $CDCl_3$) broadened signals observed $\delta$ 7.29–7.40 (m, 5H), 5.69–5.85 (m, 1H), 5.16 (s, 2H), 5.03–5.12 (m, 2H), 4.03–4.21 (m, 2H), 3.43–3.52 (m, 2H), 2.35 (s, 2H), 2.26 (t, $J$ = 2.5 Hz, 1H); $^{13}$C NMR (151 MHz, $CDCl_3$) doublet signals observed $\delta$ 155.7, 155.3, 136.5, 135.0, 134.9, 128.4, 127.9, 127.8, 127.7, 116.9, 79.2, 72.0, 71.9, 67.3, 46.5, 45.7, 36.5, 36.4, 32.6, 32.2; HRMS (MALDI): calcd. for $C_{15}H_{18}NO_2$ $[M+H]^+$ 244.1332; found 244.1332.

**15:** To a cooled solution of **13** (515 mg, 2.12 mmol) in dry THF (10 mL) at −78 °C was added LiHMDS (2.1 mL, 1.0 M in THF, 2.1 mmol) dropwise. The resulting mixture was stirred at −78 °C for 2 h prior to the addition of $ClCO_2Et$ (0.98 mL, 10 mmol). The resulting mixture was stirred at −78 °C for 1 h then quenched at −78 °C with brine (15 mL). The mixture was allowed to warm up to rt then extracted with DCM. The combined organic phases were dried over $Na_2SO_4$, filtered and concentrated under reduced pressure. The crude material was purified by DCVC using a gradient elution (toluene followed by EtOAc–toluene 1:10 to 1:4) to yield a mixture of the product **15** and starting material **13** (561.4 mg) as a slightly yellow oil; NMR data showed 16% of remaining starting material giving the

calculated yield of product **15** (500 mg, 75%). A small portion was purified by preparative TLC (500 microns) using toluene–EtOAc 9:1 as eluent to furnish pure product **15** as colorless oil. The reaction was repeated three times up to a scale of 4.22 g of product **15** with an average yield of 73%. $^1$H NMR (600 MHz, $CDCl_3$) rotamer broadened/doublet signals observed *δ* 7.29–7.40 (m, 5H), 5.67–5.84 (m, 1H), 5.16 (s, 2H), 4.99–5.14 (m, 2H), 4.18–4.31 (m, 4H), 3.46 (t, *J* = 6.0 Hz, 2H), 2.34 (br, 2H), 1.31 (t, *J* = 7.1 Hz, 3H); $^{13}$C NMR (151 MHz, $CDCl_3$) rotamer doublet signals observed *δ* 155.9, 155.3, 153.3, 136.4, 134.9, 134.8, 128.6, 128.3, 128.1, 117.4, 83.0, 75.8, 75.7, 67.8, 62.3, 47.2, 46.3, 36.8, 32.9, 32.4, 14.1; HRMS (MALDI): calcd. for $C_{18}H_{22}NO_4$ $[M+H]^+$ 316.1543; found 316.1548.

**(*Z*)-16:** A solution of protected amine **15** (3.39 g, 85 wt %, 9.12 mmol) and LiI (1.96 g, 14.6 mmol) in AcOH (100 mL) was heated at 70 °C for 1 h. The reaction mixture was cooled to rt and AcOH was removed azeotropically with toluene. The crude material was purified by DCVC using a gradient elution (toluene followed by EtOAc–toluene 1:15 to 1:9) to provide a mixture of the product (*Z*)**-16** and the starting material **15** (3.95 g) as a slightly yellow oil. NMR data showed 10% of remaining starting material giving the calculated yield of product (*Z*)**-16** (3.65 g, 90%). A small portion was purified by preparative TLC (500 microns) using toluene–EtOAc 9:1 as eluent to furnish the pure product (*Z*)**-16** as a pale yellow oil. The reaction was repeated up to a scale of 4.56 g of product (*Z*)**-16** with an average yield of 89%. $^1$H NMR (600 MHz, $CDCl_3$) rotamer broadened/doublet signals observed *δ* 7.28–7.39 (m, 5H), 6.37–6.40 (m, 1H), 5.65–5.82 (m, 1H), 5.16–5.18 (m, 2H), 5.00–5.12 (m, 2H), 4.27-4.32 (m 2H), 4.19–4.25 (m, 2H), 3.32-3.41 (m, 2H), 2.25–2.35 (m, 2H), 1.30 (t, *J* = 7.1 Hz, 3H); $^{13}$C NMR (151 MHz, $CDCl_3$) rotamer doublet signals observed *δ* 164.2, 156.2, 155.7, 136.5, 136.4, 134.9, 134.7, 128.64, 128.58, 128.3, 128.2, 128.0, 124.4, 124.0, 117.5, 117.4, 116.24, 116.20, 67.8, 67.6, 60.9, 60.8, 60.7, 47.5, 46.8, 33.0, 32.5, 14.3; HRMS (MALDI): calcd. for $C_{18}H_{23}INO_4$ $[M+H]^+$ 444.0666; found 444.0687.

**(*E*)-17:** To a solution of (*Z*)-**16** (2.60 g, 5.88 mmol) in dry THF (50 mL) under argon atmosphere were successively added $PdCl_2(PPh_3)_2$ (413 mg, 0.59 mmol) and $Ag_2CO_3$ (3.24 g, 11.7 mmol) at rt. The reaction mixture was heated at 60 °C for 4 h then filtered through a short plug of silica using EtOAc as eluent. The crude material was purified by DCVC using a gradient elution (toluene followed by EtOAc–toluene 1:15 to 1:9) but unfortunately the two isomers (*E*)-**17** and (*Z*)-**17** could not be separated (1.25 g, 67%). A

small portion was purified by preparative TLC (500 microns) using toluene–EtOAc 9:1 as eluent to furnish the desired isomer (*E*)-**17**. $^1$H NMR (600 MHz, $CDCl_3$) $\delta$ 7.29–7.41 (m, 5H), 5.69–5.77 (m, 1H), 5.15 (s, 2H), 5.14 (s, 2H), 4.14 (q, $J$ = 7.1 Hz, 2H), 4.05 (s, 2H), 3.62 (t, $J$ = 5.3 Hz, 2H), 2.43 (br s, 2H), 1.26 (t, $J$ = 7.1 Hz, 3H); $^{13}$C NMR (151 MHz, $CDCl_3$) $\delta$ 166.1, 155.1, 149.2, 141.2, 136.7, 128.7, 128.3, 128.1, 116.8, 116.5, 115.4, 67.6, 60.4, 52.6, 52.5, 45.4, 35.5, 35.3, 14.2; HRMS (MALDI): calcd. for $C_{18}H_{22}NO_4$ $[M+H]^+$ 316.1543; found 316.1543.

**18+19:** To a solution of the isomers of (*E*)-**17** and (*Z*)-**17** (1.97 g, 6.24 mmol) in THF (200 mL) and $H_2O$ (200 mL) was added LiOH (1.50 g, 62.6 mmol) at rt. The reaction mixture was stirred at this temperature overnight then quenched with aqueous 1 M HCl. The aqueous layer was extracted with DCM and the combined organic layer were washed with brine, dried over $Na_2SO_4$, filtered and concentrated in vacuo to yield a mixture of two isomeric products **18** and **19** (1.75 g, 98%) as a sticky oil. Unfortunately, the two isomers could not be separated. $^1$H NMR (600 MHz, $CDCl_3$) rotamer/isomer signals observed $\delta$ 7.31–7.36 (m, 5H), 5.70-5.78 (m, 1H), 5.17–5.20 (m, 2H), 5.14 (s, 2H), 4.70 (s, 1H), 4.07 (br s, 2H), 3.63 (t, $J$ = 5.6 Hz, 2H), 2.44 (br s, 2H); $^{13}$C NMR (151 MHz, $CDCl_3$) rotamer/isomer signals observed $\delta$ 170.2, 155.2, 151.9, 151.7, 141.0, 140.8, 136.5, 128.7, 128.3, 128.1, 116.1, 115.8, 115.6, 68.1, 67.7, 52.8, 52.6, 45.4, 35.5; HRMS (MALDI): calcd. for $C_{16}H_{18}NO_4$ $[M+H]^+$ 288.1230; found 288.1234.

**20:** To a solution of the isomers of **18** and **19** (1.01 g, 3.53 mmol) in toluene (150 mL) was added BHT (ca. 10 mg) at rt. The reaction mixture was heated under reflux conditions for 5 h. The solvent was removed under reduced pressure and the crude material was purified by DCVC using a gradient elution (toluene followed by EtOAc–toluene 1:5 to 1:1) to lead to product **20** (453 mg, 45%) as a slightly yellow sticky oil (27% over 3 steps from derivative **16**). The reaction sequence was repeated 3 times with an average yield of 31% over 3 steps from **16**. $^1$H NMR (600 MHz, $CDCl_3$) rotamer broadened signals observed $\delta$ 7.30–7.37 (m, 5H), 5.15 (s, 2H), 4.75 (s, 2H), 3.86 (s, 2H), 3.64 (t, $J$ = 5.7 Hz, 2H), 2.96 (br s, 2H), 2.06 (br s, 2H); $^{13}$C NMR (151 MHz, $CDCl_3$) rotamer doublet signals observed $\delta$ 168.2, 155.3, 136.5, 128.6, 128.3, 128.2, 123.7, 123.3, 122.2, 121.6, 71.2, 67.5, 45.0, 40.5, 40.1, 31.4, 24.8, 24.5; HRMS (MALDI): calcd. for $C_{16}H_{17}NNaO_4$ $[M+Na]^+$ 310.1050; found 310.1052.

**21**: Potassium carbonate (10.50 g, 76 mmol) was added to a solution of *N*-methylprop-2-yn-1-amine (4.27 mL, 50.6 mmol) and 4-bromobut-1-ene (5.14 mL, 50.6 mmol) in acetone (150 mL) at rt. The reaction mixture was heated at 70 °C in a sealed tube or under reflux conditions for 24 h. Volatiles were removed under reduced pressure to provide the desired product **21** (4.29 g, 69%) as a colorless oil which was used in the next step without further purification. $R_f$ = 0.52 (EtOAc); $^1$H NMR (600 MHz, $CDCl_3$) δ 5.74 (ddt, *J* = 17.0 Hz and *J* = 10.2 Hz and *J* = 6.7 Hz, 1H), 5.01 (dq, *J* = 17.1 Hz and *J* = 1.7 Hz, 1H), 4.95 (dq, *J* = 10.2 Hz and *J* = 1.4 Hz, 1H), 3.30 (d, *J* = 2.4 Hz, 2H), 2.42–2.48 (m, 2H), 2.26 (s, 3H), 2.16–2.19 (m, 2H), 2.15 (t, *J* = 2.4 Hz, 1H); $^{13}$C NMR (151 MHz, $CDCl_3$) δ 135.4, 114.7, 77.3, 72.1, 53.9, 44.4, 40.6, 31.0; LC-MS (*m*/*z*) = 124 $[M+H]^+$.

**22**: *n*-BuLi (1.5 M in hexane, 34.1 mL, 51.1 mmol) was added dropwise over 5 min to a cooled solution of **21** (4.20 g, 34.1 mmol) in dry THF (150 mL) at −78 °C. After complete addition, the reaction mixture was stirred for another 5 min at the same temperature prior to the dropwise addition of ethyl chloroformate (6.50 mL, 68.2 mmol). The resulting mixture was stirred at −78 °C for 1 h then quenched cold by adding saturated aqueous $NH_4Cl$ (50 mL) at −78 °C and allowed to reach rt. Solvents were removed under reduced pressure and the crude material was extracted with EtOAc (3 × 100 mL). The combined organic layers were washed with brine (2 × 30 mL), dried over $MgSO_4$, filtered, evaporated onto Celite, and purified by DCVC (heptane followed by EtOAc–heptane 1:10 followed by EtOAc–MeOH 10:1) to provide product **22** (4.76 g, 72%) as a pale yellow oil. $R_f$ = 0.70 (EtOAc); $^1$H NMR (600 MHz, $CDCl_3$) δ 5.73 (ddt, *J* = 17.0 Hz and *J* = 10.3 Hz and *J* = 6.7 Hz, 1H), 5.02 (dt, *J* = 17.2 Hz and *J* = 1.7 Hz, 1H), 4.96 (dq, *J* = 10.2 Hz and *J* = 1.5 Hz, 1H), 4.17 (q, *J* = 7.1 Hz, 2H), 3.46 (s, 2H), 2.48 (t, *J* = 7.3 Hz, 2H), 2.29 (s, 3H), 2.13–2.22 (m, 2H), 1.25 (t, *J* = 7.1 Hz, 3H); $^{13}$C NMR (151 MHz, $CDCl_3$) δ 153.3, 136.0, 116.0, 82.7, 77.9, 62.0, 55.0, 45.5, 41.7, 31.9, 14.0; LC-MS (*m*/*z*) = 196 $[M+H]^+$; HRMS (ESI): calcd. for $C_{11}H_{18}NO_2$ $[M+H]^+$ 196.1332; found 196.1339.

***(Z)*-23**: Lithium iodide (4.89 g, 36.6 mmol) was added to a solution of compound **22** (4.76 g, 24.38 mmol) in AcOH (150 mL) at rt. The reaction mixture was heated at 50 °C for 3 h then volatiles were removed in vacuo and the remaining crude material was dissolved in EtOAc (100 mL) and washed with aqueous 1 M $Na_2S_2O_3$ (2 × 50 mL). The aqueous layer was extracted with EtOAc (2 × 30 mL) and the combined organic layers were washed

with water (20 mL), dried over $MgSO_4$, filtered, evaporated onto Celite, and purified by DCVC (heptane followed by EtOAc–heptane 1:20 ) to provide *(Z)*-**23** (6.26 g, 79%) as a yellow oil. $R_f$ = 0.86 (heptane–EtOAc, 1:1); $^1$H NMR (400 MHz, $CDCl_3$) δ 6.72 (s, 1H), 5.75 (ddt, *J* = 17.0 Hz and *J* = 10.2 Hz and *J* = 6.7 Hz, 1H), 5.00 (dq, *J* = 17.1, 1.7 Hz, 1H), 4.95 (ddt, *J* = 10.2 Hz and *J* = 2.0 Hz and *J* = 1.2 Hz, 1H), 4.16 (q, *J* = 7.1 Hz, 2H), 3.30 (s, 2H), 2.44 (t, *J* = 7.1 Hz, 2H), 2.12– 2.28 (m, 5H), 1.24 (t, *J* = 7.1 Hz, 3H); $^{13}$C NMR (101 MHz, $CDCl_3$) δ 164.8, 136.4, 125.2, 119.7, 115.9, 71.5, 60.6, 56.7, 41.9, 31.9, 14.2; LC-MS (*m*/*z*) = 324 $[M+H]^+$; HRMS (ESI): calcd. for $C_{11}H_{19}INO_2$ $[M+H]^+$ 324.0455; found 324.0464.

***(E)*-24**: Silver carbonate (2.39 g, 8.66 mmol) and bis(triphenylphosphine)palladium(II) chloride (304 mg, 0.43 mmol) were added to a flame-dried Schlenk tube, evacuated and purged again with argon. Dry THF (60 mL) and *(Z)*-**23** (1.40 g, 4.33 mmol) were successively added to the Schlenk tube, evacuated and purged again with argon. The mixture was stirred at 50 °C for 20 h, filtered through a small plug of Celite, washed with EtOAc and the resulting filtrate was evaporated onto Celite then purified by DCVC (heptane followed by EtOAc–heptane 1:10 followed by EtOAc–MeOH 20:1) to provide *(E)*-**24** (630 mg, 75%) as colorless oil along with minor amounts of alkyne **22** (110 mg, 13%). $R_f$ = 0.58 (EtOAc–MeOH, 1:2); $^1$H NMR (600 MHz, $CDCl_3$) δ 5.62 (s, 1H), 5.03 (q, *J* = 1.5 Hz, 1H), 5.01 (d, *J* = 1.7 Hz, 1H), 4.07 (q, *J* = 7.1 Hz, 2H), 2.98 (s, 2H), 2.59 (t, *J* = 5.8 Hz, 2H), 2.41 (t, *J* = 5.7 Hz, 2H), 2.32 (s, 3H), 1.17 (t, *J* = 7.1 Hz, 3H); $^{13}$C NMR (151 MHz, $CDCl_3$) δ 170.1, 165.0, 149.4, 115.2, 113.1, 62.7, 59.1, 55.3, 44.0, 33.5, 13.0; LC-MS (*m*/*z*) = 196 $[M+H]^+$; HRMS (ESI): calcd. for $C_{11}H_{18}NO_2$ $[M+H]^+$ 196.1332; found 196.1336.

**25**: Lithium hydroxide (155 mg, 6.45 mmol) was added to a solution of *(E)*-**24** (630 mg, 3.23 mmol) in water (5 mL) and THF (15 mL) and stirred at rt for 20 h. The mixture was evaporated directly onto Celite and purified by DCVC (heptane followed by EtOAc–heptane 1:4 followed by EtOAc–MeOH 20:1) to provide **25** (435 mg, 81%) as a hygroscopic white semi-solid which was pure enough for the next step. $R_f$ = 0.21 (EtOAc–MeOH, 1:2); $^1$H NMR (600 MHz, $CD_3OD$) δ 5.75 (s, 1H), 5.14 (s, 1H), 4.91 (s, 1H), 2.97 (d, *J* = 1.3 Hz, 2H), 2.57 (dd, *J* = 6.6 Hz and *J* = 4.9 Hz, 2H), 2.43 (t, *J* = 5.8 Hz, 2H), 2.30 (s, 3H); LC-MS (*m*/*z*) = 168 $[M+H]^+$; HRMS (ESI): calcd. for $C_9H_{14}NO_2$ $[M+H]^+$ 168.1019; found 168.1016.

**26:** A solution of compound **25** (21 mg, 0.126 mmol) in HFIP (2 mL) was heated at 80 °C in a sealed MW vial for 15 h. Upon cooling, DMSO (1 mL) was added to the mixture and could now be concentrated in vacuo (without co-evaporating the desired compound) to provide the crude reaction mixture as a DMSO solution, which was purified by preparative LC–MS to provide **26** (5.2 mg, 0.019 mmol, 15%) as a TFA salt. Compound **26** was isolated as a 0.31 mM DMSO solution for handling purposes, as an increased concentration of the compound seemed to cause decomposition. The concentration of the DMSO solution was determined by NMR using a standard Bruker experiment for concentration determination (15 s relaxation time). NB: It was not possible to isolate the product by any sorts of regular chromatography (flash chromatography, preparative TLC, DCVC) or crystallization of the amine salts due to decomposition of the product. $R_f$ = 0.47 (EtOAc–MeOH–TEA, 20:20:1); $^1$H NMR (600 MHz, DMSO-$d_6$) δ 11.08 (s, 1H), 4.81 (d, $J$ = 2.5 Hz, 2H), 3.71 (d, $J$ = 15.6 Hz, 1H), 3.49–3.58 (m, 1H), 3.42–3.49 (m, 1H), 3.11–3.16 (m, 1H), 3.03–3.07 (m, 2H), 2.80 (d, $J$ = 4.5 Hz, 3H), 2.42–2.52 (m, 1H), 2.21–2.30 (m, 1H); $^{13}$C NMR (151 MHz, DMSO-$d_6$) δ 168.4, 158.8 (q, $J$ = 38.2 Hz), 123.4, 118.9, 115.5 (q, $J$ = 288.4 Hz), 70.3, 51.9, 49.4, 41.9, 31.4, 21.9; HRMS (ESI): calcd. for $C_9H_{14}NO_2$ $[M+H]^+$ 168.1019; found 168.1022.

**27**: To a solution of 3-bromobut-3-en-1-amine hydrochloride (1.99 g, 10.8 mmol) in DCM (25 mL) at rt was added TEA (3 mL, 21.6 mmol). The mixture was cooled to 0 °C and stirred at this temperature 5 min prior to the addition of *p*-NsCl (2.39 g, 10.8 mmol) in one portion. The reaction mixture was allowed to warm up to rt and stirred at this temperature for 1 h. After addition of DCM (25 mL) the organic layer was washed successively with an aqueous solution of HCl (1 N, 50 mL) and brine (50 mL), dried over $Na_2SO_4$, filtered and concentrated in vacuo. The resulting solid was purified by column chromatography using a gradient elution (EtOAc–PE, 1:3 to 1:2) to afford pure sulfonamide **27** (3.49 g, 97%) as a pale yellow solid; mp 92–94 °C; $R_f$ = 0.65 (EtOAc–PE, 1:2); $^1$H NMR (300 MHz): δ 8.37 (d, $J$ = 9.1 Hz, 2H), 8.06 (d, $J$ = 9.1 Hz, 2H), 5.65–5.67 (m, 1H), 5.52–5.54 (m, 1H), 4.73 (br t, $J$ = 6.1 Hz, 1H), 3.23–3.31 (m, 2H), 2.63 (t, $J$ = 6.9 Hz, 2H); $^{13}$C NMR (75 MHz): δ 150.4, 146.0, 129.5, 128.6 (2C), 124.7 (2C), 121.0, 41.8, 41.4; HRMS (ESI): calcd. $C_{10}H_{12}BrN_2O_4S$ $[M+H]^+$ 334.9701; found 334.9699.

**28**: To a solution of sulfonamide **27** (4.14 g, 12.4 mmol) in $CH_3CN$ (110 mL) at rt were successively added $Cs_2CO_3$ (5.00 g, 14.8 mmol) and methyl bromoacetate (1.4 mL, 14.8 mmol). The mixture was stirred at this temperature for 1 h then the solvent was removed in vacuo. The crude material was taken up in EtOAc (100 mL) and the organic layer was washed with water (100 mL). The aqueous layer was extracted with EtOAc (100 mL) and the combined organic layers were washed with brine (150 mL), dried over $Na_2SO_4$, filtered and concentrated in vacuo. The resulting solid was purified by column chromatography using a gradient elution (EtOAc–PE, 1:3 to 1:2) to furnish ester **28** (4.88 g, 97%) as a pale yellow solid; mp 110–112 °C; $R_f$ = 0.70 (EtOAc–PE, 1:2); $^1$H NMR (300 MHz, $CDCl_3$): δ 8.37 (d, $J$ = 8.5, 2H), 8.05 (d, $J$ = 8.5 Hz, 2H), 5.72–5.74 (m, 1H), 5.51–5.53 (m, 1H), 4.22 (s, 2H), 3.67 (s, 3H), 3.51 (t, $J$ = 6.9 Hz, 2H), 2.77 (t, $J$ = 6.9 Hz, 2H); $^{13}$C NMR (75 MHz, $CDCl_3$): δ 169.2, 150.4, 145.5, 129.9, 129.1 (2C), 124.5 (2C), 120.4, 52.9, 49.4, 47.6, 41.6; HRMS (ESI): calcd. for $C_{13}H_{16}BrN_2O_6S$ $[M+H]^+$ 406.9912; found 406.9909.

**29**: To a cooled solution of ester **28** (1.06 g, 2.6 mmol) in dry THF (25 mL) at 0 °C under nitrogen atmosphere was added lithium borohydride (0.11 g, 5.2 mmol) portionwise. The mixture was stirred 5 min at this temperature then allowed to warm up to rt and stirred at this temperature for 1.5 h. The solvent was removed in vacuo then the crude material was taken up in EtOAc (30 mL). The organic layer was washed successively with water (30 mL) and the aqueous layer was extracted with EtOAc (2 × 30 mL). The combined organic layers were washed with brine (100 mL), dried over $Na_2SO_4$, filtered and concentrated in vacuo. The resulting oil was dissolved in DCM (20 mL) and added dropwise to a solution of DMP reagent (1.55 g, 3.6 mmol) in dry DCM (15 mL) at rt. The mixture was stirred at this temperature for 1 h then at 40 °C for 1 additional hour. Another portion of DMP reagent (0.66 g, 1.6 mmol) was added and the mixture was stirred under reflux conditions for 3 additional hours then allowed to cool to rt. After successive washings with saturated aqueous solution of $Na_2S_2O_3$ (30 mL), saturated aqueous solution of $NaHCO_3$ (30 mL) and brine (30 mL), the organic layer was dried over $Na_2SO_4$, filtered and concentrated in vacuo. The resulting crude material was purified by column chromatography using a gradient elution (EtOAc–PE, 1:2 to 2:3) to give aldehyde **29** (0.64 g, 65% over two steps) as a pale yellow solid; mp 118–121 °C; $R_f$ = 0.5 (EtOAc–PE, 2:3); $^1$H NMR (300 MHz, $CDCl_3$): δ 9.56 (s, 1H), 8.38 (d, $J$ = 8.7 Hz, 2H), 8.02 (d, $J$ = 8.7

Hz, 2H), 5.73–5.75 (m, 1H), 5.52–5.54 (m, 1H), 4.20 (s, 2H), 3.48 (t, *J* = 6.3 Hz, 2H), 2.76 (t, *J* = 6.3 Hz, 2H); $^{13}$C NMR (75 MHz, $CDCl_3$): δ 196.4, 150.6, 144.9, 129.8, 129.0 (2C), 124.8 (2C), 120.8, 58.4, 48.4, 42.0; HRMS (ESI): calcd. for $C_{12}H_{14}BrN_2O_5S$ $[M+H]^+$ 376.9807; found 376.9802.

**(*Z*)-30/31**: To a cooled homogenous solution of bis(2,2,2-trifluoroethyl) (methoxycarbonylmethyl)phosphonate (0.35 g, 1.1 mmol) and 18-crown-6 (1.06 g, 4.0 mmol) in dry THF (5 mL) at −78 °C under nitrogen atmosphere was added a solution of KHMDS (2.24 mL, 1.1 mmol, 0.5 M in toluene) dropwise over 10 min. After stirring 40 min at this temperature, a pre-cooled solution of aldehyde **29** (0.30 g, 0.8 mmol) in dry THF (5.5 mL) at 0 °C was added dropwise over 10 min. The mixture was allowed to slowly warm to rt then quenched with a saturated aqueous solution of $NH_4Cl$ (25 mL). THF was removed in vacuo and the aqueous layer was extracted with EtOAc (2 × 30 mL). The combined organic layers were washed with brine (60 mL), dried over $Na_2SO_4$, filtered and concentrated in vacuo. The resulting oil was purified by column chromatography using EtOAc–PE 1:3 as the eluent to provide a mixture of **(*Z*)-30/31** in a 2:3 ratio (0.27 g, 79%) as a yellow oil.

**(*Z*)-30**: $R_f$ = 0.55 (EtOAc–PE, 1:3); $^1$H NMR (300 MHz, $CDCl_3$): δ 8.37 (d, *J* = 8.8 Hz, 2H), 8.02 (d, *J* = 8.8 Hz, 2H), 6.20 (dt, *J* = 11.6 Hz and *J* = 6.0 Hz, 1H), 5.91 (dt, *J* = 11.6 Hz and *J* = 1.9 Hz, 1H), 5.66 (d, *J* = 1.9 Hz, 1H), 5.45 (d, *J* = 1.7 Hz, 1H), 4.47 (dd, *J* = 6.0 Hz and *J* = 2.2 Hz, 2H), 3.72 (s, 3H), 3.43 (t, *J* = 7.4 Hz, 2H), 2.73 (t, *J* = 7.3 Hz, 2H); $^{13}$C NMR (75 MHz, $CDCl_3$): δ 166.2, 148.0, 145.3, 145.2, 129.6, 128.6 (2C), 124.7 (2C), 122.1, 120.0, 51.9, 48.0, 47.4, 41.3; HRMS (ESI): calcd. for $C_{15}H_{18}BrN_2O_6S$ $[M+H]^+$ 433.0069; found 433.0065.

**31**: $R_f$ = 0.6 (EtOAc–PE, 1:3); $^1$H NMR (300 MHz, $CDCl_3$): δ 8.35 (d, *J* = 8.8 Hz, 2H), 7.98 (d, *J* = 8.8 Hz, 2H), 6.64 (d, *J* = 14.3 Hz, 1H), 5.68 (d, *J* = 1.9 Hz, 1H), 5.47 (d, *J* = 1.7 Hz, 1H), 5.09 (dt, *J* = 14.3 Hz and *J* = 7.4 Hz, 1H), 3.69 (s, 3H), 3.58 (t, *J* = 7.7 Hz, 2H), 3.10 (d, *J* = 7.2 Hz, 2H), 2.75 (t, *J* = 7.7 Hz, 2H).

**(*E*)-30**: To a cooled solution of aldehyde **29** (0.79 g, 2.1 mmol) in dry THF (14 mL) at 0 °C under nitrogen atmosphere was added methyl (triphenylphosphoranylidene)acetate (0.73 g, 2.2 mmol) portionwise. The resulting mixture was stirred for 2 h at this

temperature then quenched with a saturated aqueous solution of $NH_4Cl$ (15 mL). THF was removed in vacuo then the aqueous layer was extracted with EtOAc (2 × 30 mL). The combined organic layers were washed with brine (50 mL), dried over $Na_2SO_4$, filtered and concentrated in vacuo. The resulting crude was purified by column chromatography using EtOAc–PE 1:3 as the eluent to afford ester ***(Z)*–30** (0.09 g, 10%) as a colorless oil and ester ***(E)*–30** (0.78 g, 86%) as a pale yellow solid. mp 91–93 °C; $R_f$ = 0.35 (EtOAc–PE, 1:3); $^1$H NMR (300 MHz, $CDCl_3$): δ 8.33 (d, *J* = 8.8, 2H), 7.99 (d, *J* = 8.8 Hz, 2H), 6.67 (dt, *J* = 15.3 Hz and *J* = 6.1 Hz, 1H), 5.91 (d, *J* = 15.3 Hz, 1H), 5.63 (br s, 1H), 5.45 (d, *J* = 3.1 Hz, 1H), 4.05 (t, *J* = 6.1 Hz, 2H), 3.72 (s, 3H), 3.42 (t, *J* = 6.1 Hz, 2H), 2.70 (t, *J* = 6.1 Hz, 2H); $^{13}$C NMR (75 MHz, $CDCl_3$): δ 165.8, 150.3, 145.4, 141.6, 129.6, 128.6 (2C), 124.8 (2C), 124.4, 120.2, 52.2, 49.8, 47.2, 41.5; HRMS (ESI): calcd. for $C_{15}H_{18}BrN_2O_6S$ $[M+H]^+$ 433.0069; found 433.0065.

**33**: To a solution of 3-chloropyridine-4-carbaldehyde (3.58 g, 25.3 mmol) in MeOH (150 mL) at 0 °C was added $NaBH_4$ (1.91 g, 50.6 mmol) portionwise. The mixture was stirred at this temperature for 5 min then allowed to warm up to rt and stirred at this temperature for 1.5 h. The reaction mixture was quenched with saturated aqueous solution of $NH_4Cl$. Volatiles were removed in vacuo and the aqueous layer was extracted with EtOAc (150 mL). The organic layer was washed successively with water (150 mL) and brine (150 mL), dried over $Na_2SO_4$, filtered and concentrated in vacuo to give chloropyridine **33** (3.58 g, 99%) as a white solid which was used in the next step without further purification; mp 110–114 °C; $R_f$ = 0.4 (EtOAc–heptane, 2:1); $^1$H NMR (300 MHz, $CDCl_3$): δ 8.48–8.49 (m, 1H), 8.48 (d, *J* = 5.0, 1H), 7.54 (dd, *J* = 5.0 and *J* = 0.5, 1H), 4.83 (s, 2H), 2.10 (br s, 1H); $^{13}$C NMR (75 MHz, $CDCl_3$): δ 148.6, 148.1, 147.9, 129.8, 122.1, 61.3; HRMS (ESI): calcd. for $C_6H_7ClNO$ $[M+H]^+$ 144.0216; found 144.0212.

**34**: In a MW vial were successively added SPhos (74 mg, 6 mol %), $Pd(OAc)_2$ (20 mg, 3 mol %), $K_3PO_4$ (1.59 g, 7.5 mmol), pyridine **33** (431 mg, 3.0 mmol), (*E*)-2-(2-ethoxyvinyl)-4,4,5,5-tetramethyl-1,3,2-dioxaborolane (1.13 g, 5.7 mmol), $CH_3CN$ (12 mL) and water (8 mL). The MW vial was purged with $N_2$ for 5 min then heated at 120 °C for 2 h under MW conditions. The resulting mixture was filtered through a pad of Celite and the pad was washed several times with EtOAc. The filtrate was washed with brine, dried over $Na_2SO_4$, filtered and concentrated in vacuo. The crude material was purified by column

chromatography using EtOAc (100%) as eluent to provide pyridine **34** (343 mg, 64%) as a colorless oil; $R_f$ = 0.35 (EtOAc, 100%) $^1$H NMR (300 MHz, $CDCl_3$): δ 8.28 (s, 1H), 8.21 (d, *J* = 5.0, 1H), 7.38 (d, *J* = 5.0, 1H), 6.78 (d, *J* = 12.9, 1H), 5.73 (d, *J* = 12.9, 1H), 4.66 (s, 2H), 4.59 (br s, 1H), 3.88 (q, *J* = 6.9, 2H), 1.32 (t, *J* = 6.9, 3H); $^{13}$C NMR (75 MHz, $CDCl_3$): δ 150.1, 147.2, 146.7, 145.4, 130.2, 121.2, 99.3, 66.3, 61.5, 15.2; HRMS (ESI): calcd. for $C_{10}H_{14}NO_2$ $[M+H]^+$ 180.1025; found 180.1029. In addition, chloropyridine **33** (75 mg, 17%) was also recovered.

**35**: To a solution of pyridine **34** (40 mg, 0.2 mmol) in DCM (2.2 mL) was added TFA (1.1 mL) dropwise at rt. The mixture was stirred at this temperature for 3 h then solvents were removed in vacuo. The crude product was purified by column chromatography using EtOAc (100%) as eluent to provide pyridine **35** (34 mg, 85%) as a pale yellow oil; $R_f$ = 0.35 (EtOAc, 100%); $^1$H NMR (300 MHz, $CDCl_3$): δ 8.29–8.36 (m, 2H), 6.90 (d, *J* = 5.0, 1H), 5.06–5.11 (m, 1H), 4.78 (d, *J* = 15.9, 1H), 4.63 (d, *J* = 15.9, 1H), 3.80–3.92 (m, 1H), 3.51–3.64 (m, 1H), 3.04 (dd, *J* = 16.8 and *J* = 4.1, 1H), 2.79 (dd, *J* = 16.8 and *J* = 2.5, 1H), 1.22 (t, *J* = 7.1, 3H); $^{13}$C NMR (75 MHz, $CDCl_3$): δ 150.2, 146.9, 143.1, 127.3, 118.9, 96.5, 63.8, 61.1, 30.8, 15.4; HRMS (ESI): calcd. for $C_{10}H_{14}NO_2$ $[M+H]^+$ 180.1025; found 180.1024.

**37**: To a solution of chloropyridine **33** (1.46 g, 10.2 mmol) and imidazole (1.52 g, 22.4 mmol) in dry DMF (30 mL) at rt under $N_2$ was added a solution of *tert*-butyldimethylsilyl chloride (1.84 g, 12.2 mmol) in dry DMF (10 mL). The mixture was stirred at this temperature for 2 h then EtOAc (50 mL) and water (150 mL) were added. The aqueous layer was extracted with EtOAc (2 × 50 mL) and the combined organic layers were washed with brine, dried over $Na_2SO_4$, filtered and concentrated in vacuo. The crude product was purified by column chromatography using a gradient eluent (EtOAc–PE, 1:10 to 1:3) to provide pyridine **37** (2.62 g, 100%) as a colorless oil; $R_f$ = 0.4 (EtOAc–PE, 1:10); $^1$H NMR (300 MHz, $CDCl_3$): δ 8.45–8.52 (m, 2H), 7.51 (d, *J* = 5.2, 1H), 0.98 (s, 9H), 0.16 (s, 6H); $^{13}$C NMR (75 MHz, $CDCl_3$): δ 149.4, 149.04, 148.99, 130.0, 122.6, 62.5, 27.1 (3C), 19.6, -4.1 (2C); HRMS (ESI): calcd. for $C_{12}H_{21}ClNOSi$ $[M+H]^+$ 258.1081; found 258.1087.

**38**: In a MW vial were successively added SPhos (38 mg, 6 mol %), $Pd(OAc)_2$ (10 mg, 3 mol %), $K_3PO_4$ (652 mg, 3.1 mmol), chloropyridine **37** (396 mg, 1.5 mmol), (*E*)-2-(2-

ethoxyvinyl)-4,4,5,5-tetramethyl-1,3,2-dioxaborolane (608 mg, 3.1 mmol), $CH_3CN$ (3.3 mL) and water (2.2 mL). The MW vial was purged with $N_2$ for 5 min then heated at 85 °C for 2 h under MW conditions. The resulting mixture was filtered through a pad of Celite and the pad was washed several times with EtOAc. The filtrate was washed with brine, dried over $Na_2SO_4$, filtered and concentrated in vacuo. The crude material was purified by column chromatography using gradient eluent (EtOAc–PE, 1:1 to 3:2) to lead to pyridine **38** (343 mg, 76%) as a pale yellow oil; $R_f$ = 0.55 (EtOAc–PE, 3:2); $^1$H NMR (300 MHz, $CDCl_3$): δ 8.42 (s, 1H), 8.40 (d, *J* = 5.2, 1H), 7.38 (d, *J* = 5.2, 1H), 6.82 (d, *J* = 12.6, 1H), 5.72 (d, *J* = 12.7, 1H), 4.69 (s, 2H), 3.94 (q, *J* = 7.2, 2H), 1.37 (t, *J* = 7.3, 3H), 0.96 (s, 9H), 0.13 (s, 6H); $^{13}$C NMR (75 MHz, $CDCl_3$): δ 149.8, 147.5, 146.3, 145.9, 129.3, 120.4, 99.5, 66.3, 62.1, 26.2 (3C), 18.7, 15.1, -5.0 (2C); HRMS (ESI): calcd. for $C_{16}H_{28}NO_2Si$ $[M+H]^+$ 294.1889; found 294.1883.

**39**: To a solution of pyridine **38** (494 mg, 1.7 mmol) in dry DCM (2.2 mL) was added MeI (1.57 mL, 25.2 mmol) dropwise at rt. The mixture was stirred at this temperature for 16 h then volatiles were removed in vacuo. The crude product was taken up in methanol (20 mL) and the solution was cooled to 0 °C then $NaBH_4$ (255 mg, 6.7 mmol) was added portionwise. The mixture was stirred at this temperature for 5 min then allowed to warm to rt and stirred at this temperature for 4 h. The reaction mixture was quenched with a saturated aqueous solution of $NH_4Cl$. Methanol was removed in vacuo and the aqueous layer was extracted with EtOAc (50 mL). The organic layer was washed successively with water (50 mL) and brine (50 mL), dried over $Na_2SO_4$, filtered and concentrated in vacuo. The crude material was purified by column chromatography using EtOAc–MeOH 2:1 as eluent to provide piperidine **39** (331 mg, 85%) as a pale yellow oil; $R_f$ = 0.35 (EtOAc–MeOH, 2:1); $^1$H NMR (300 MHz, $CDCl_3$): δ 6.38 (d, *J* = 12.9, 1H), 5.77 (d, *J* = 12.9, 1H), 4.23 (s, 2H), 3.80 (q, *J* = 7.2, 2H), 3.13 (s, 2H), 2.65–2.78 (m, 2H), 2.49 (s, 3H), 2.37–2.46 (m, 2H), 1.30 (t, *J* = 7.2, 3H), 0.90 (s, 9H), 0.07 (s, 6H); $^{13}$C NMR (75 MHz, $CDCl_3$): δ 146.9, 128.6, 124.0, 102.7, 66.2, 62.1, 54.7, 51.9, 45.2, 26.9, 26.3 (3C), 18.8, 15.3, -4.8 (2C); HRMS (ESI): calcd. for $C_{17}H_{34}NO_2Si$ $[M+H]^+$ 312.2359; found 312.2368.
