# Supplementary material for: A strategic approach to [6,6]-bicyclic lactones: application towards the CD fragment of DHβE
Source: Beilstein J Org Chem. 2017 May 22;13:988–94. doi: 10.3762/bjoc.13.98 (PMC5480346; doi:10.3762/bjoc.13.98)

Supporting Information File 2

for

A strategic approach to [6,6]-bicyclic lactones: application towards the CD fragment of DHβE

Tue Heesgaard Jepsen[§], Emil Glibstrup[§], François Crestey, Anders A. Jensen and Jesper Langgaard Kristensen*

Address: Department of Drug Design and Pharmacology, Faculty of Health and Medical Sciences, University of Copenhagen, Universitetsparken 2, 2100 Copenhagen, Denmark

Email: Jesper Langgaard Kristensen - jesper.kristensen@sund.ku.dk

*Corresponding author

[§]Equally contributing authors

Copies of NMR spectra

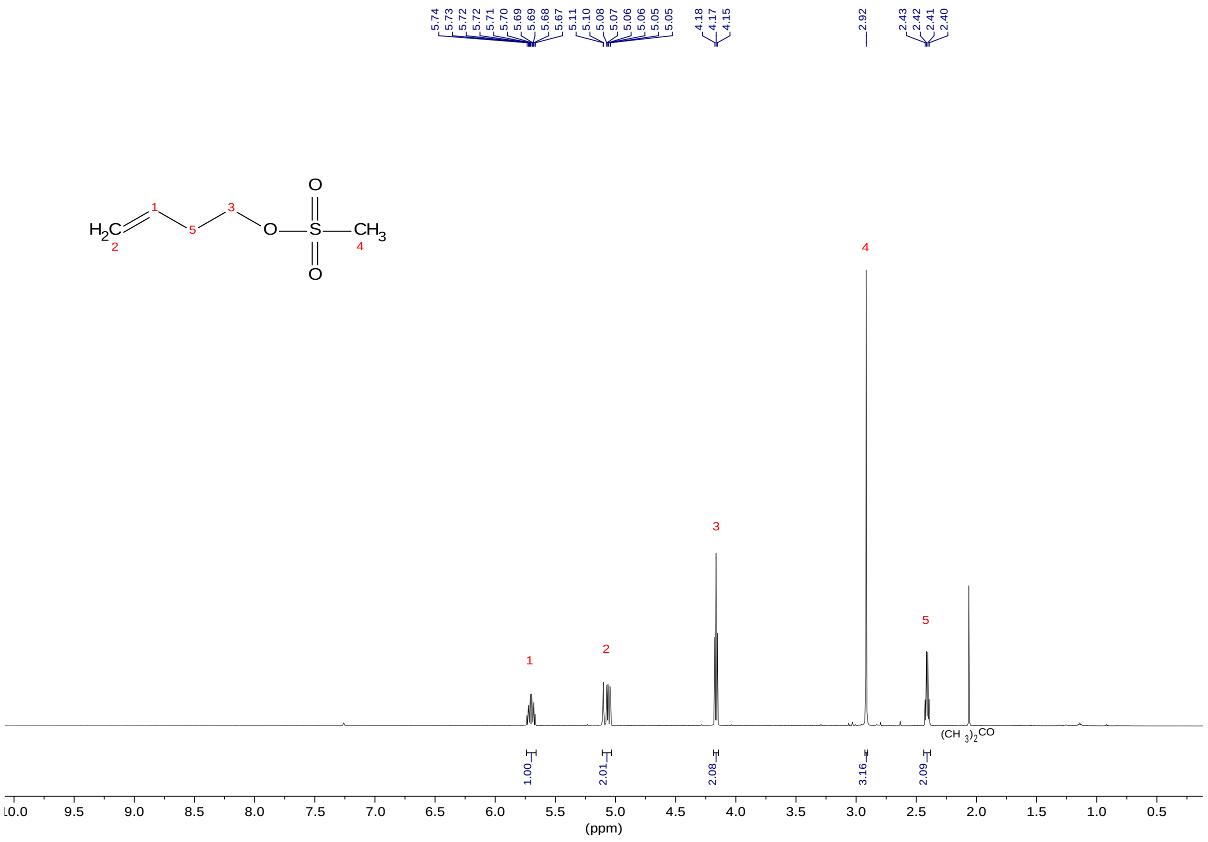
5.74 5.73 5.72 5.72 5.71 5.70 5.69 5.69 5.68 5.67 5.11 5.10 5.08 5.07 5.06 5.06 5.05 5.05 4.18 4.17 4.15 2.92 2.43 2.42 2.41 2.40
1.00 2.01 2.08 3.16 2.09
(CH3)2CO
(ppm)

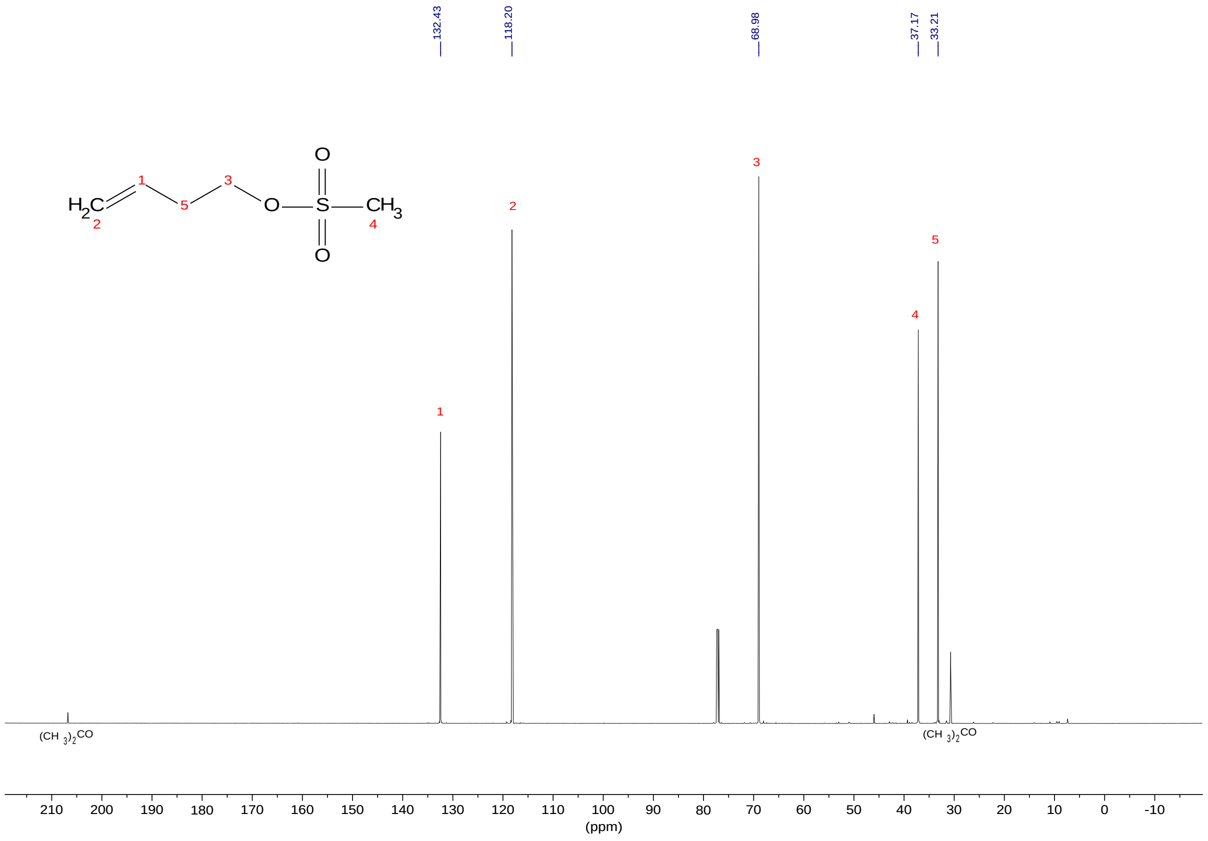
132.43 118.20 68.98 37.17 33.21
(CH3)2CO
(CH3)2CO
(ppm)

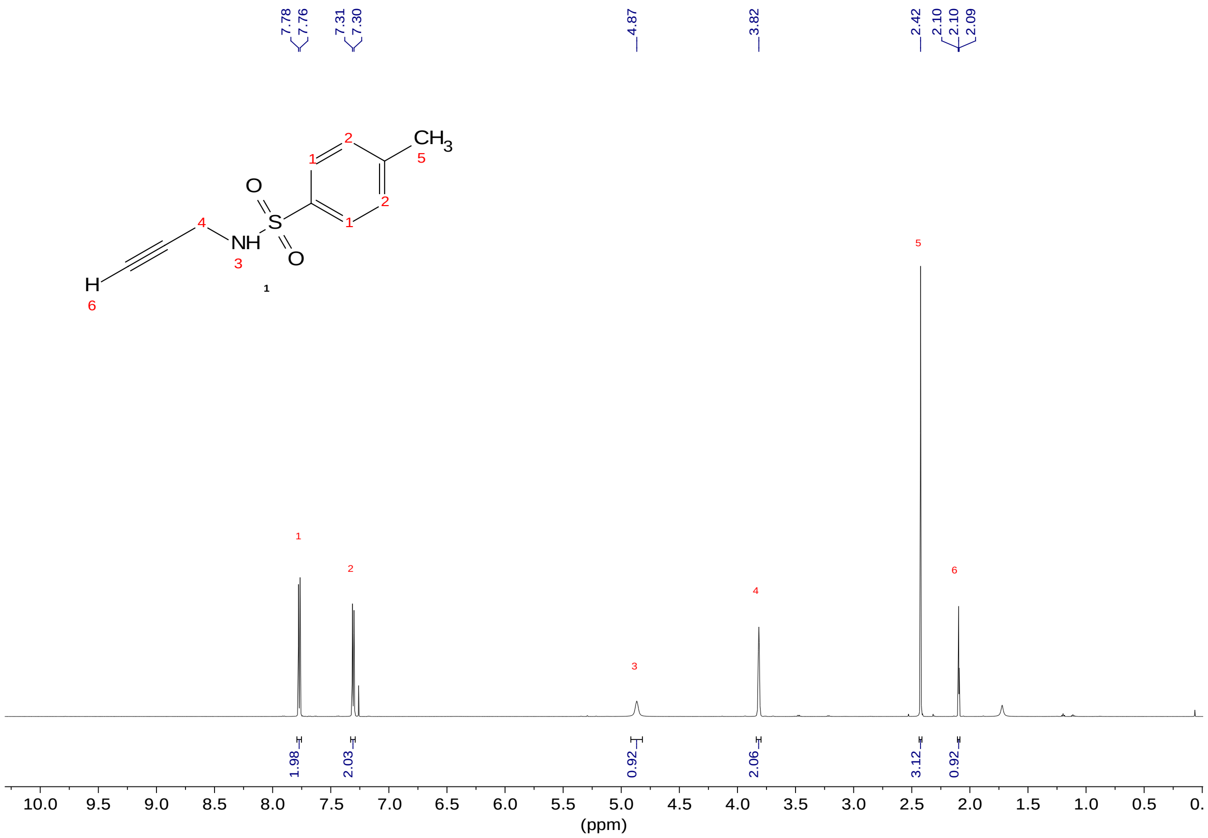
7.78
7.76
7.31
7.30
4.87
3.82
2.42
2.10
2.10
2.09
CH3
O
NH
S
O
H
1
1.98
2.03
0.92
2.06
3.12
0.92
10.0 9.5 9.0 8.5 8.0 7.5 7.0 6.5 6.0 5.5 5.0 4.5 4.0 3.5 3.0 2.5 2.0 1.5 1.0 0.5
(ppm)

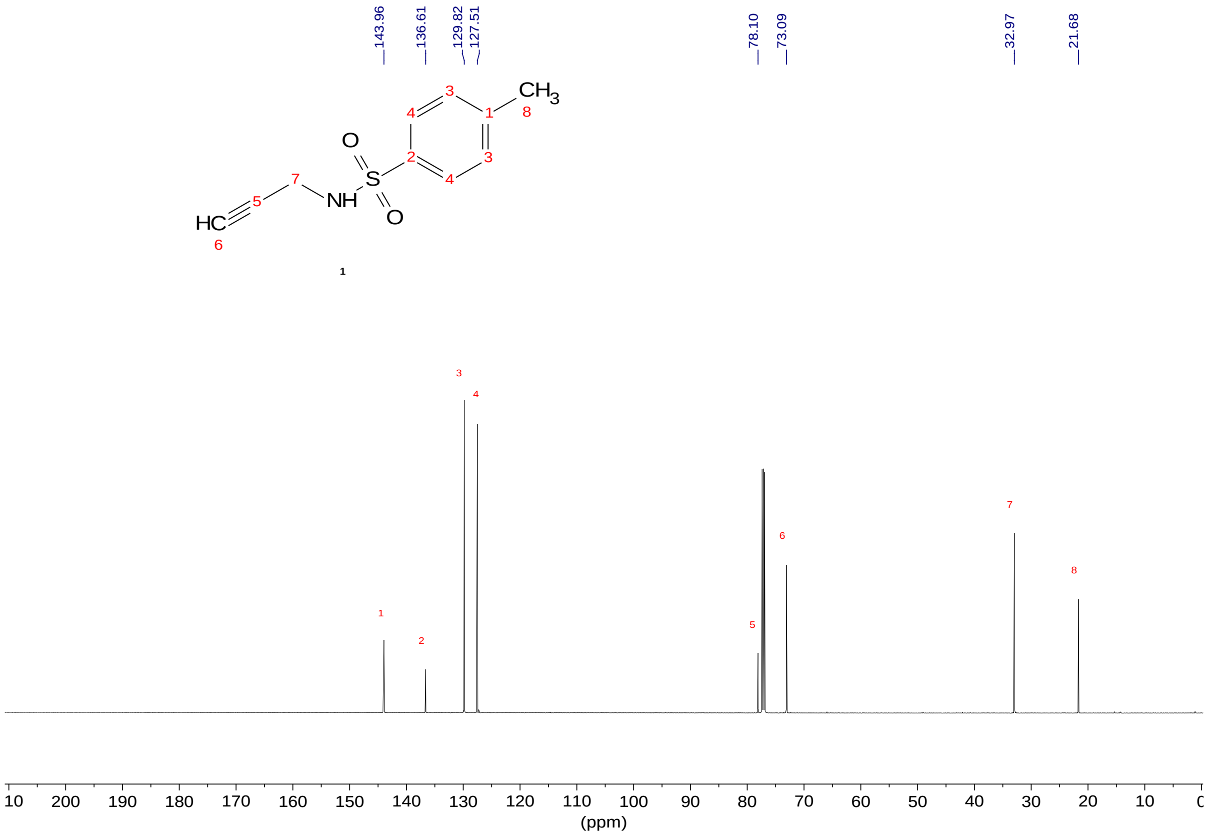
143.96
136.61
129.82
127.51
78.10
73.09
32.97
21.68
CH3
O
NH
S
O
HC
1
200 190 180 170 160 150 140 130 120 110 100 90 80 70 60 50 40 30 20 10
(ppm)

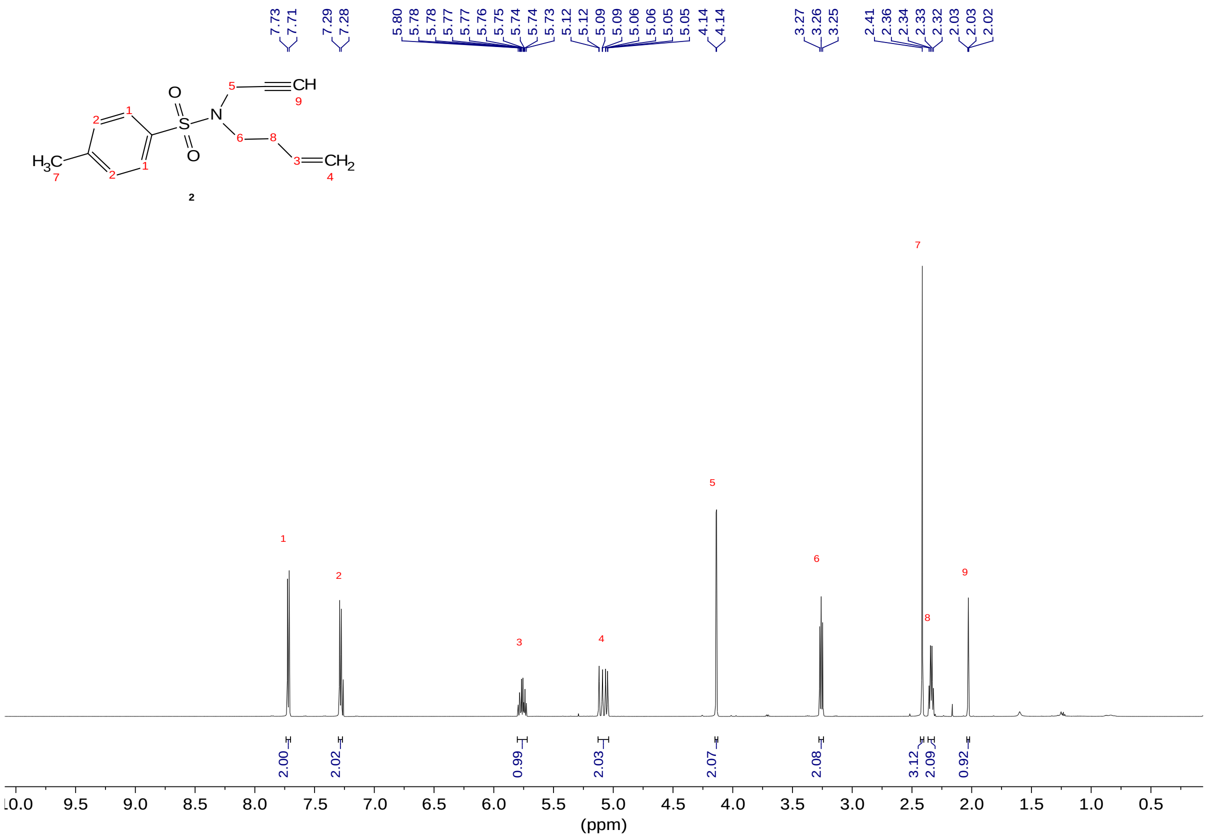
7.73
7.71
7.29
7.28
5.80
5.78
5.78
5.77
5.77
5.76
5.75
5.74
5.74
5.73
5.12
5.12
5.09
5.09
5.06
5.06
5.05
5.05
4.14
4.14
3.27
3.26
3.25
2.41
2.36
2.34
2.33
2.32
2.03
2.03
2.02
H_3C
O
O
S
N
CH
CH_2
2
2.00
2.02
0.99
2.03
2.07
2.08
3.12
2.09
0.92
(ppm)

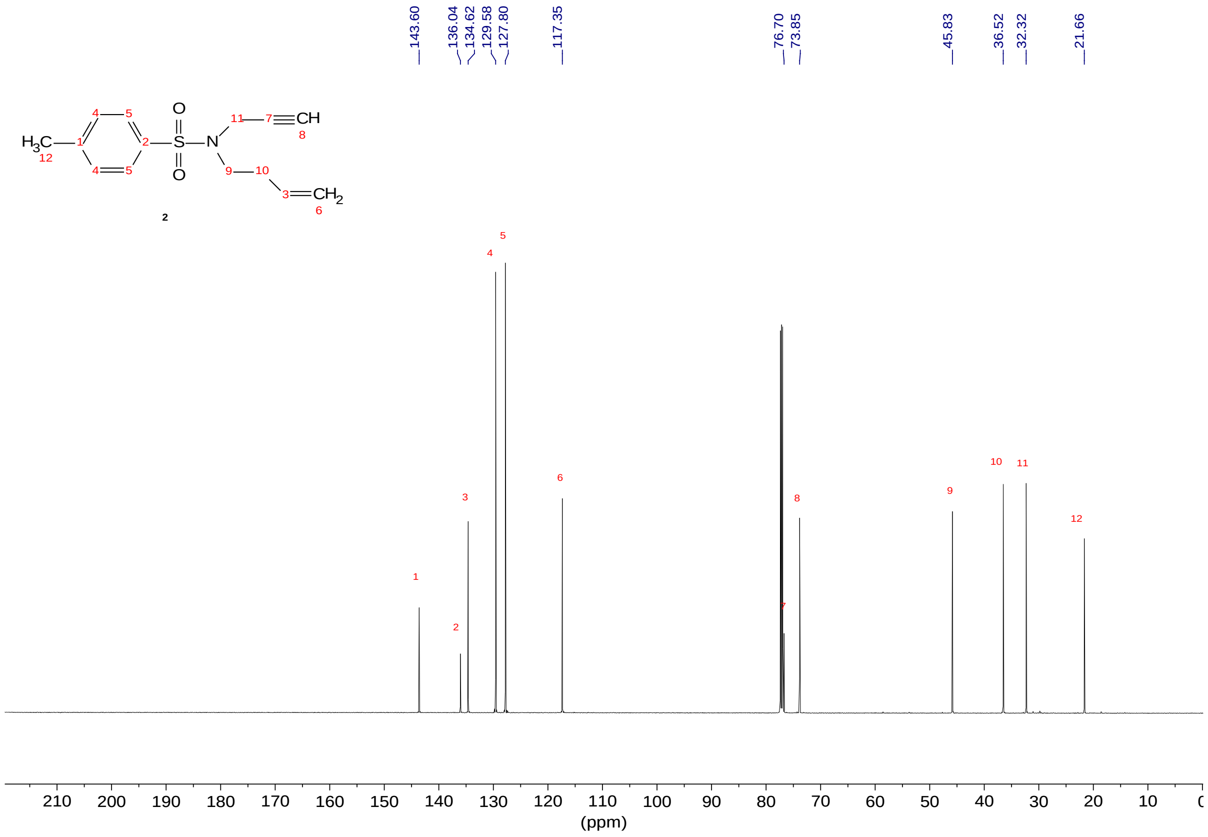
143.60
136.04
134.62
129.58
127.80
117.35
76.70
73.85
45.83
36.52
32.32
21.66
H_3C
O
O
S
N
CH
CH_2
2
(ppm)

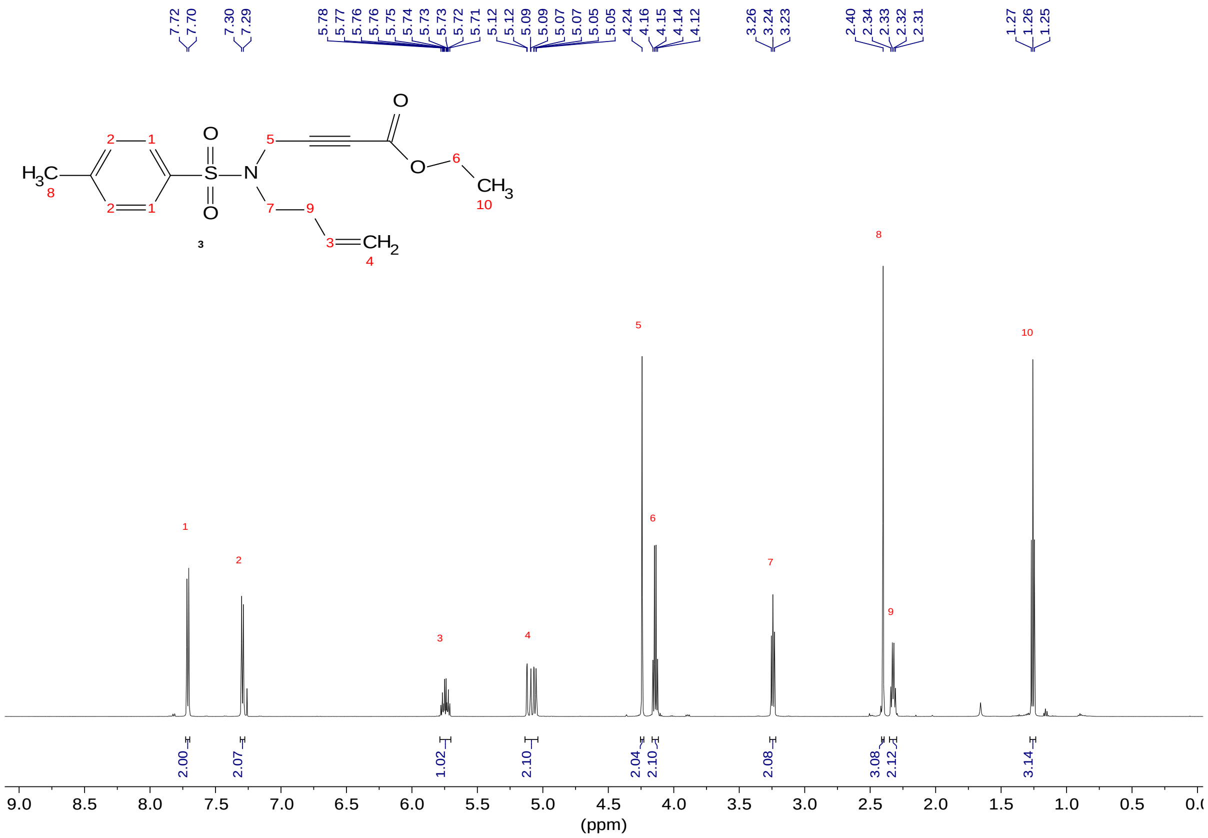
7.72 7.70 7.30 7.29 5.78 5.77 5.76 5.76 5.75 5.74 5.73 5.73 5.72 5.71 5.12 5.12 5.09 5.09 5.07 5.07 5.05 5.05 4.24 4.16 4.15 4.14 4.12 3.26 3.24 3.23 2.40 2.34 2.33 2.32 2.31 1.27 1.26 1.25
2.00 2.07 1.02 2.10 2.04 2.10 2.08 3.08 2.12 3.14
9.0 8.5 8.0 7.5 7.0 6.5 6.0 5.5 5.0 4.5 4.0 3.5 3.0 2.5 2.0 1.5 1.0 0.5
(ppm)
3

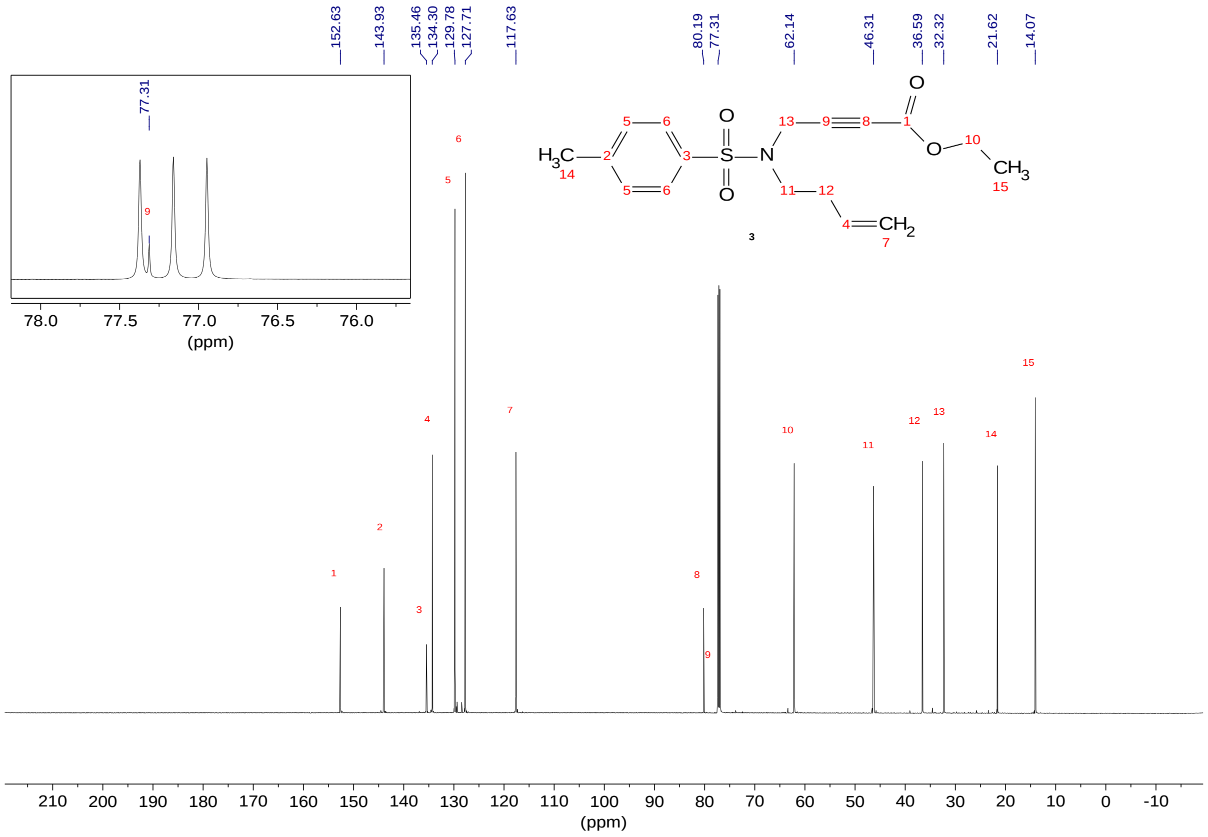
152.63 143.93 135.46 134.30 129.78 127.71 117.63 80.19 77.31 62.14 46.31 36.59 32.32 21.62 14.07
77.31
78.0 77.5 77.0 76.5 76.0
(ppm)
210 200 190 180 170 160 150 140 130 120 110 100 90 80 70 60 50 40 30 20 10 0 -10
(ppm)
3

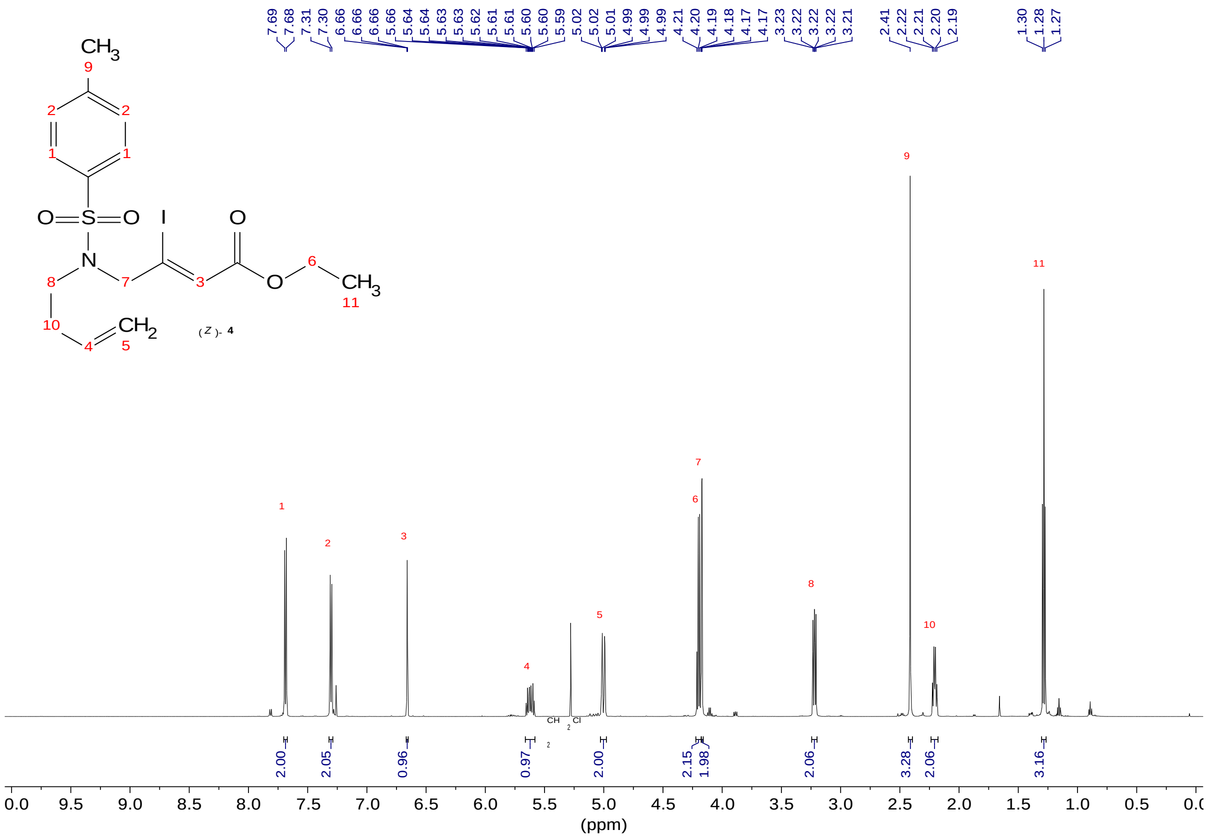
CH3
O=S=O
N
I
O
O
CH3
CH2
(Z)- 4
1 2 3 4 5 6 7 8 9 10 11
7.69 7.68 7.31 7.30 6.66 6.66 6.66 5.66 5.64 5.64 5.63 5.63 5.62 5.61 5.61 5.60 5.60 5.59 5.02 5.02 5.01 4.99 4.99 4.99 4.21 4.20 4.19 4.18 4.17 4.17 3.23 3.22 3.22 3.22 3.21 2.41 2.22 2.21 2.20 2.19 1.30 1.28 1.27
CH2Cl2
2.00 2.05 0.96 0.97 2.00 2.15 1.98 2.06 3.28 2.06 3.16
(ppm)

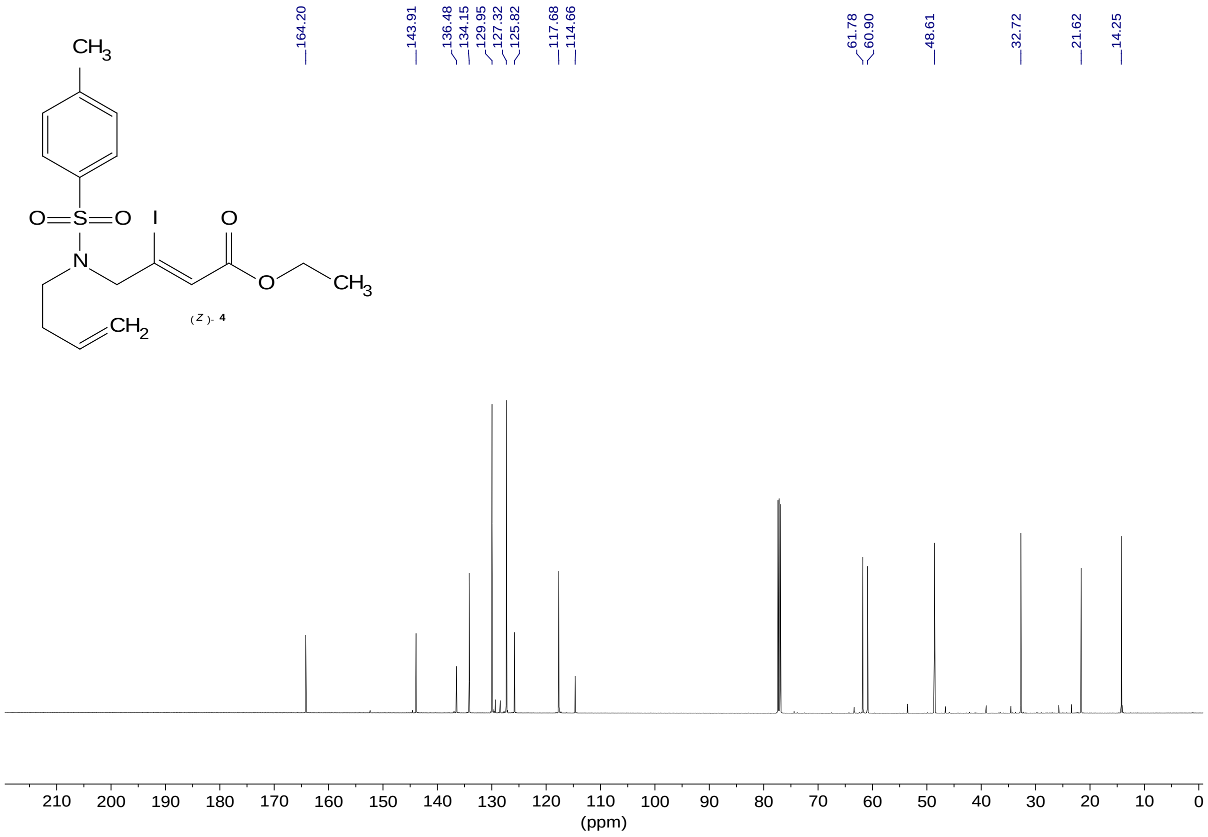
CH3
O=S=O
N
I
O
O
CH3
CH2
(Z)- 4
164.20 143.91 136.48 134.15 129.95 127.32 125.82 117.68 114.66 61.78 60.90 48.61 32.72 21.62 14.25
(ppm)

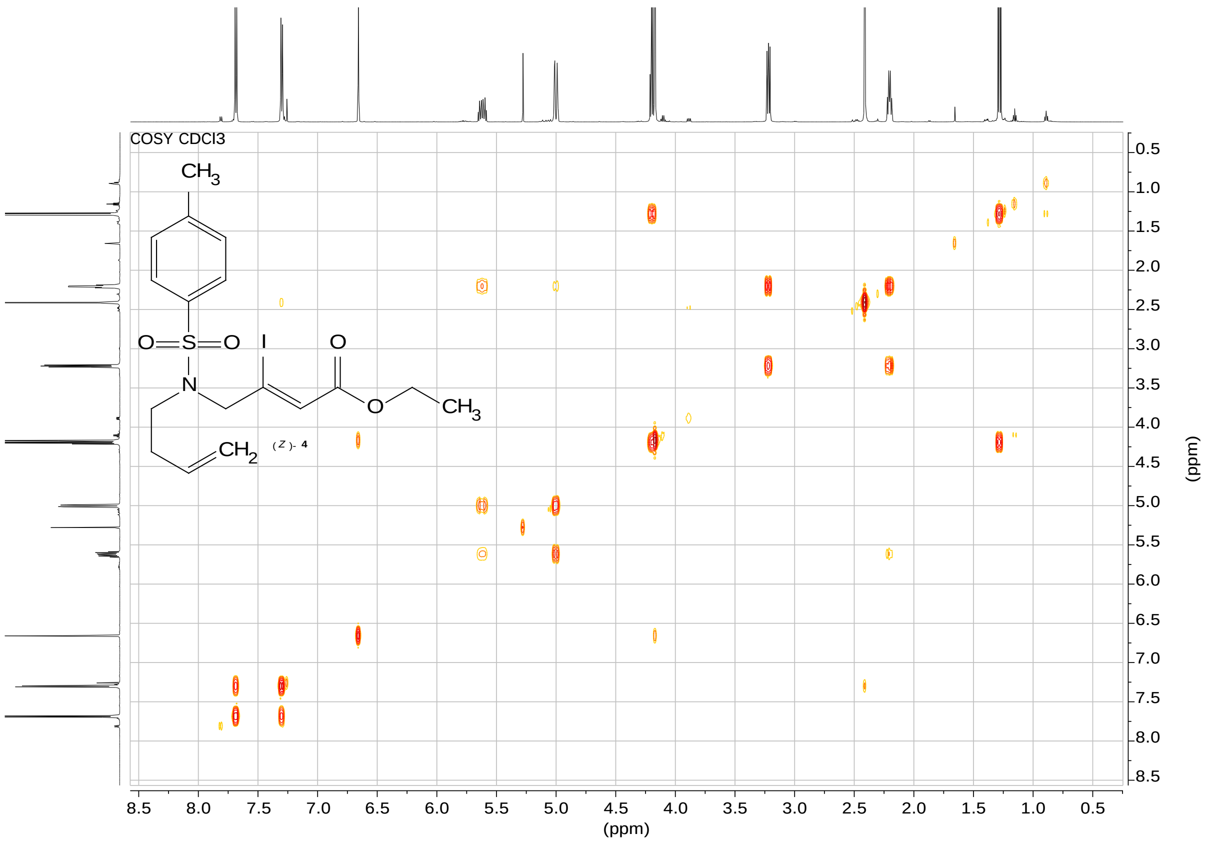
COSY CDCl3
CH3
O=S=O
I
O
N
O
CH3
CH2
(Z)- 4
8.5
8.0
7.5
7.0
6.5
6.0
5.5
5.0
4.5
4.0
3.5
3.0
2.5
2.0
1.5
1.0
0.5
(ppm)

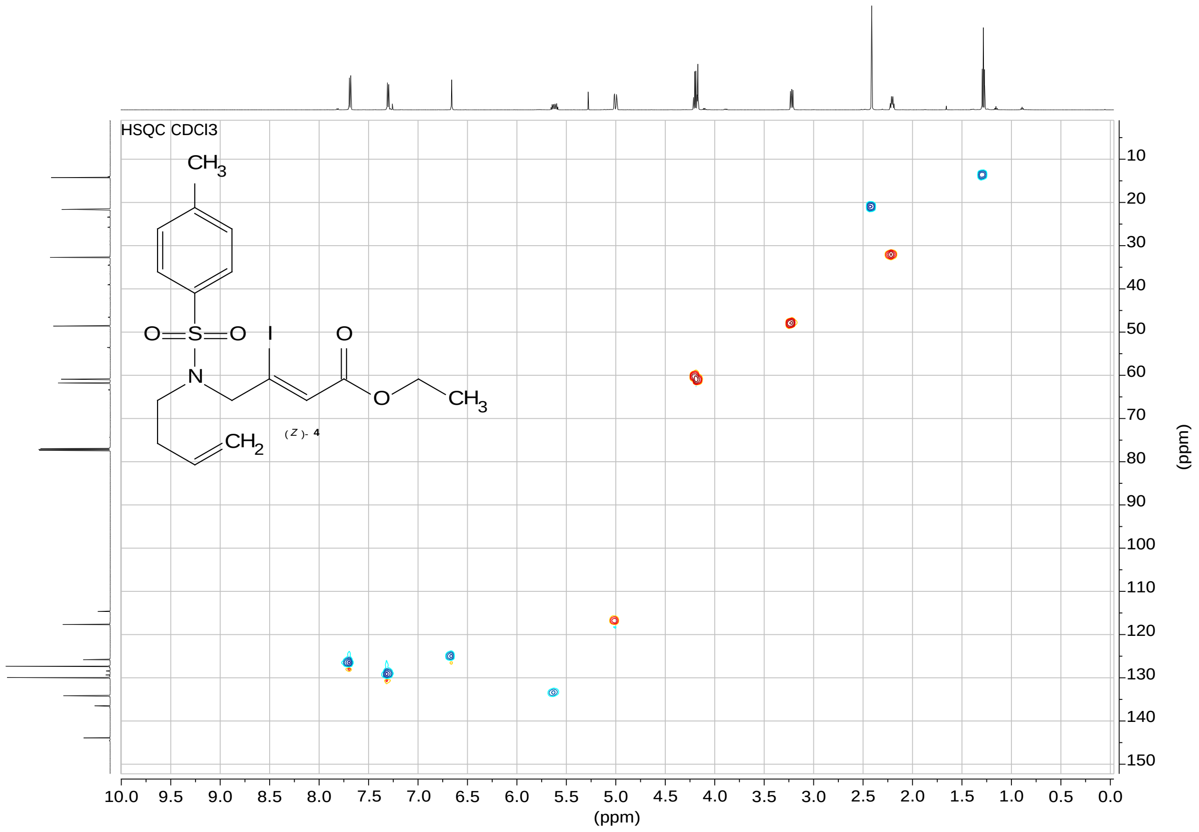
HSQC CDCl3
CH3
O=S=O
I
O
N
O
CH3
CH2
(Z)- 4
10.0
9.5
9.0
8.5
8.0
7.5
7.0
6.5
6.0
5.5
5.0
4.5
4.0
3.5
3.0
2.5
2.0
1.5
1.0
0.5
0.0
(ppm)
10
20
30
40
50
60
70
80
90
100
110
120
130
140
150

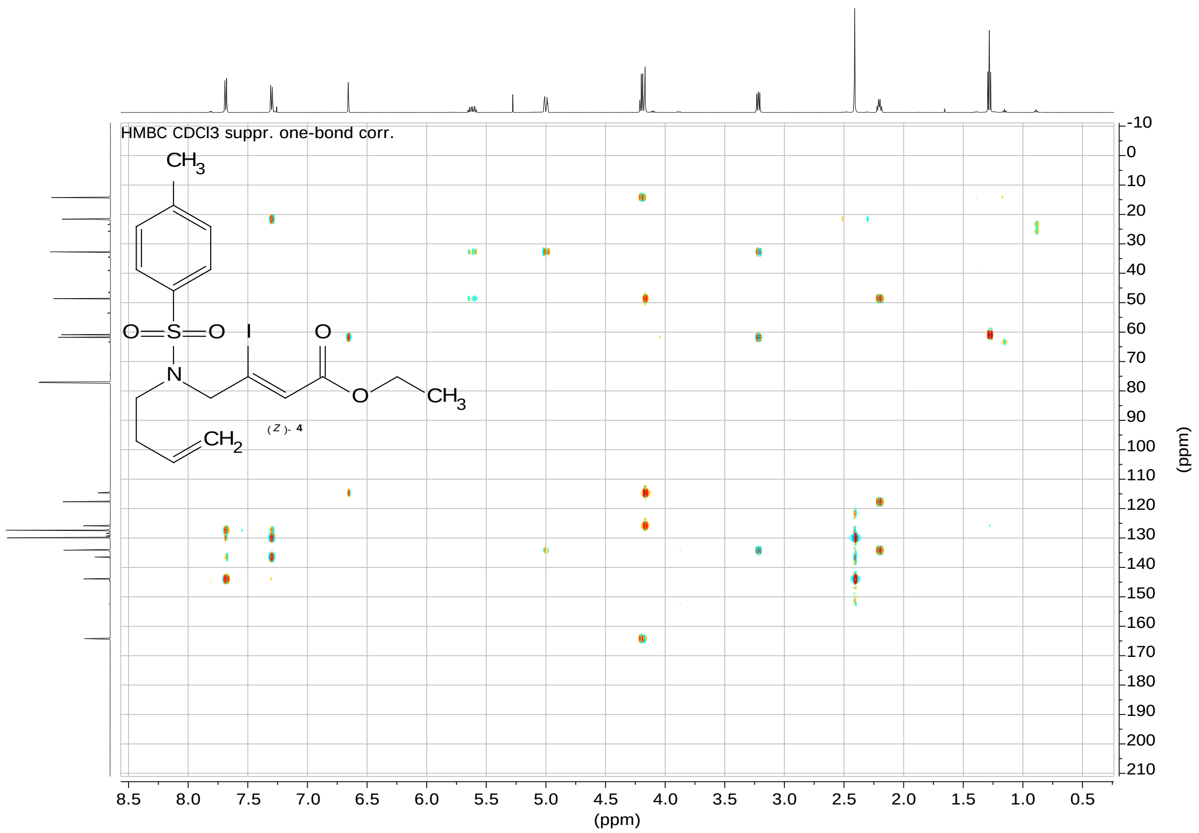

HMBC CDCl3 suppr. one-bond corr.
CH3
O=S=O
I
O
N
O
CH3
CH2
(Z)- 4
(ppm)
(ppm)

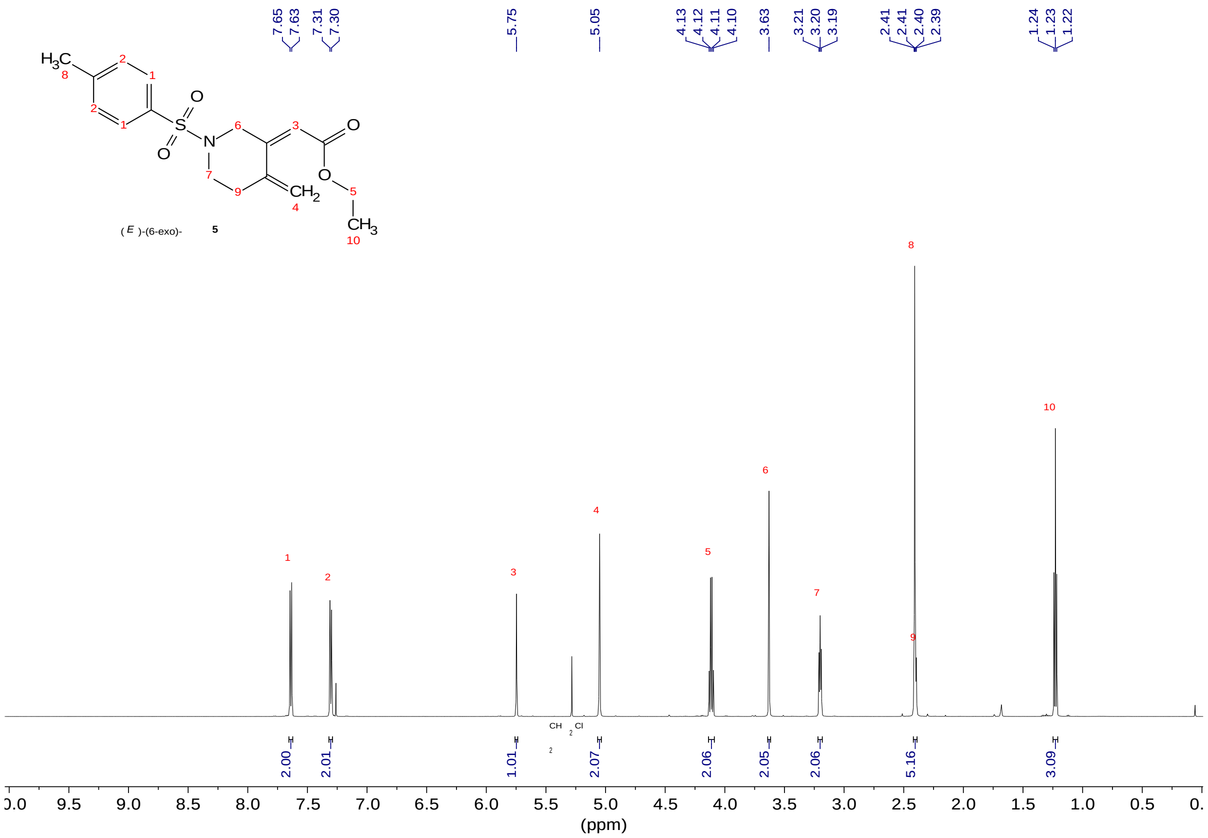
7.65
7.63
7.31
7.30
5.75
5.05
4.13
4.12
4.11
4.10
3.63
3.21
3.20
3.19
2.41
2.41
2.40
2.39
1.24
1.23
1.22
(E)-(6-exo)- 5
2.00
2.01
1.01
2.07
2.06
2.05
2.06
5.16
3.09
(ppm)

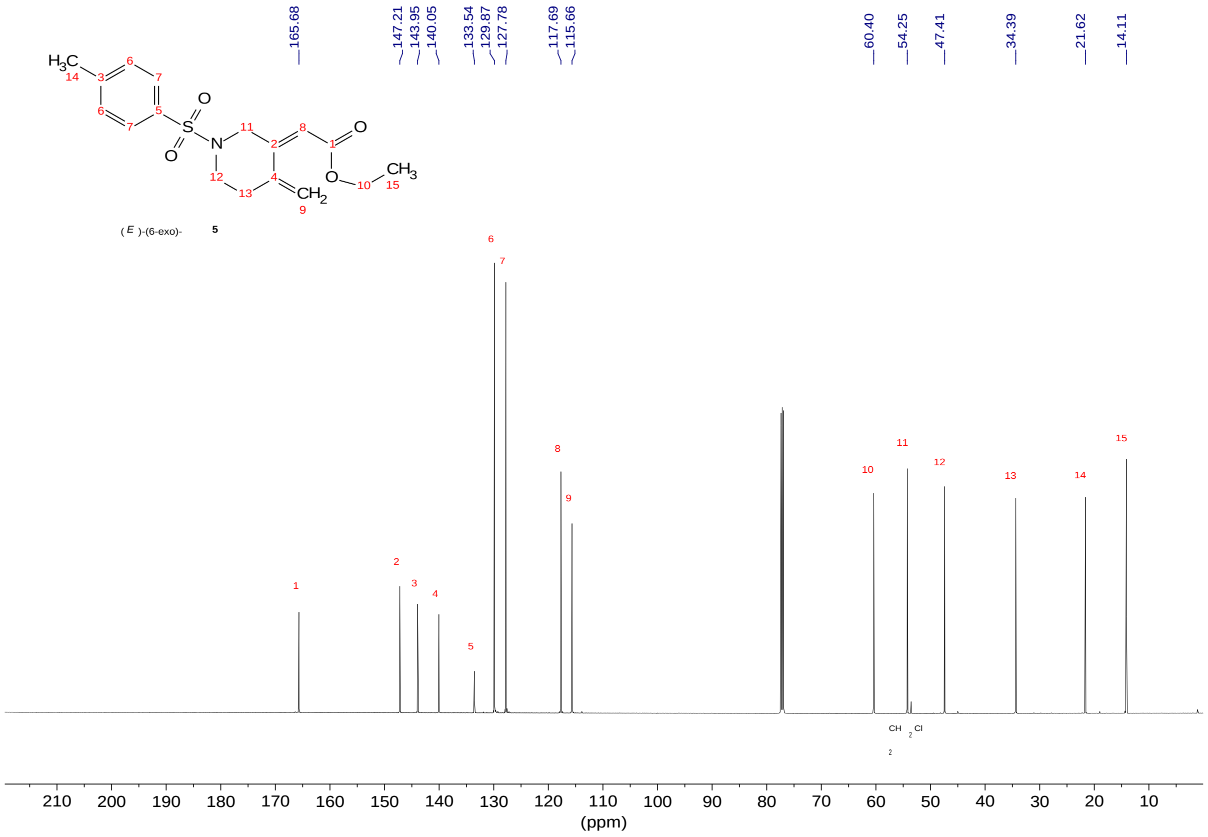
165.68
147.21
143.95
140.05
133.54
129.87
127.78
117.69
115.66
60.40
54.25
47.41
34.39
21.62
14.11
(E)-(6-exo)- 5
(ppm)

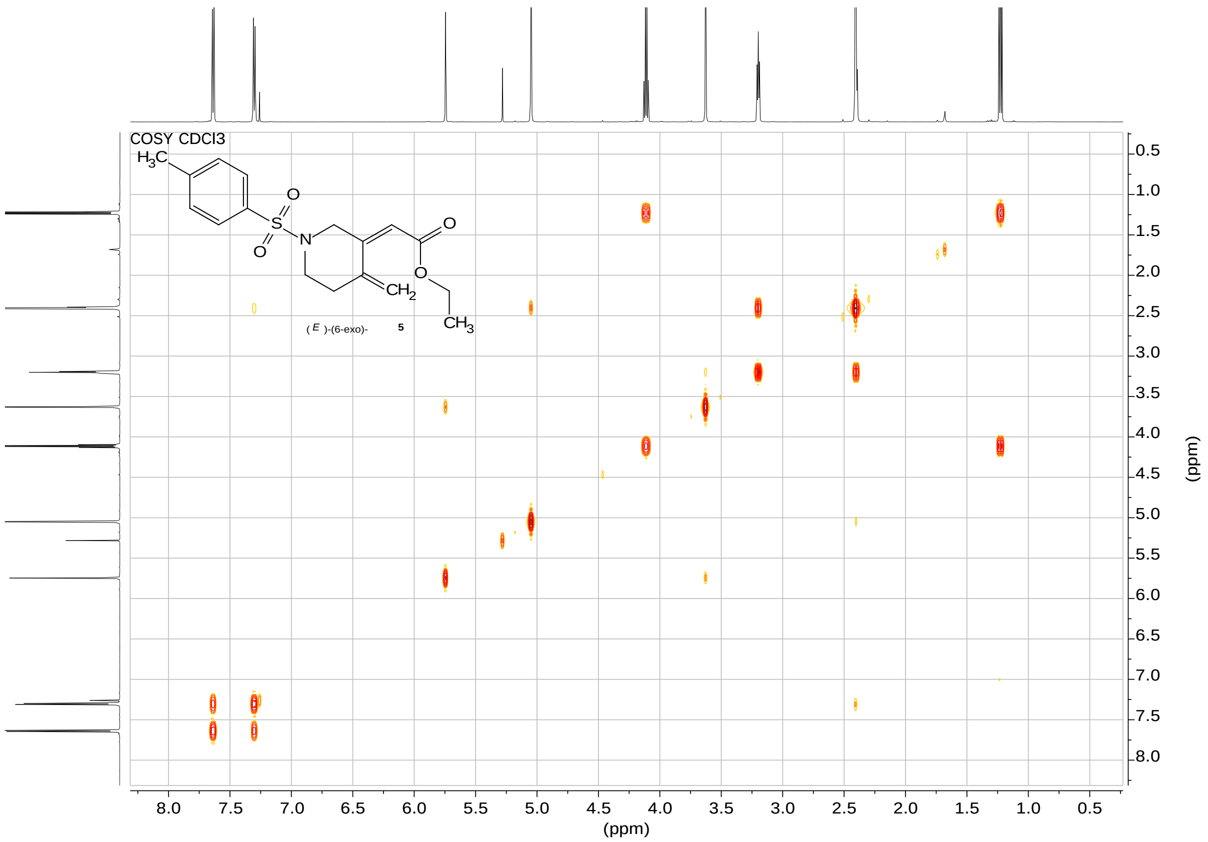
COSY CDCl3
H_3C
O
S
N
O
O
O
CH_2
CH_3
(E)-(6-exo)- 5
8.0
7.5
7.0
6.5
6.0
5.5
5.0
4.5
4.0
3.5
3.0
2.5
2.0
1.5
1.0
0.5
(ppm)

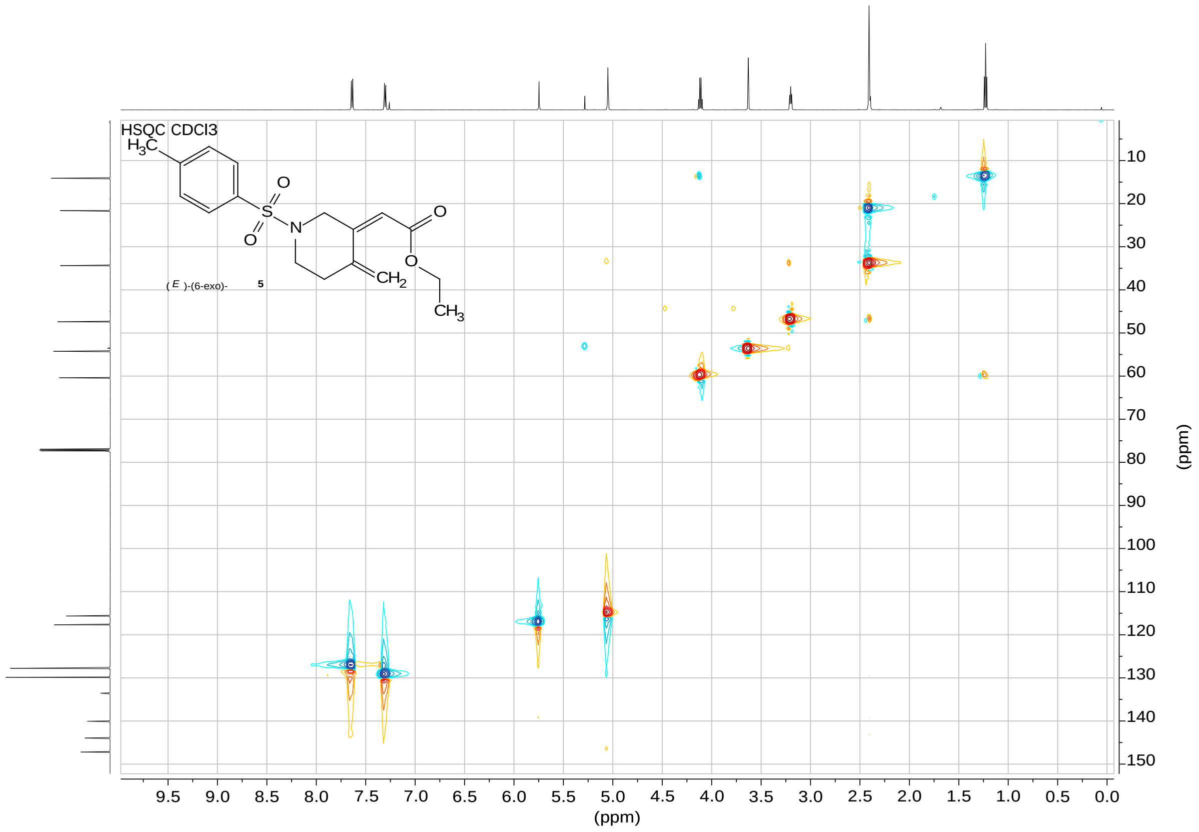
HSQC CDCl3
H_3C
O
S
N
O
O
O
CH_2
CH_3
(E)-(6-exo)- 5
9.5
9.0
8.5
8.0
7.5
7.0
6.5
6.0
5.5
5.0
4.5
4.0
3.5
3.0
2.5
2.0
1.5
1.0
0.5
0.0
(ppm)

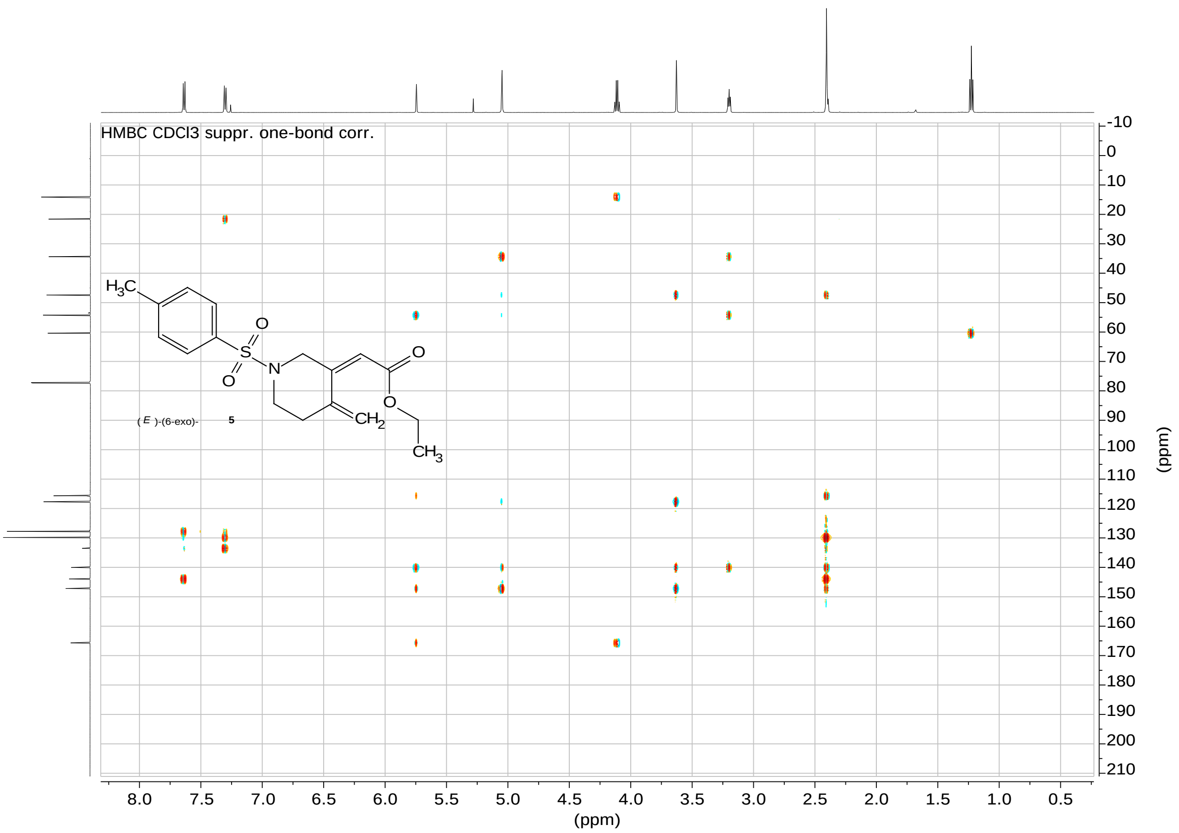
HMBC CDCl3 suppr. one-bond corr.
(E)-(6-exo)- 5
(ppm)
(ppm)

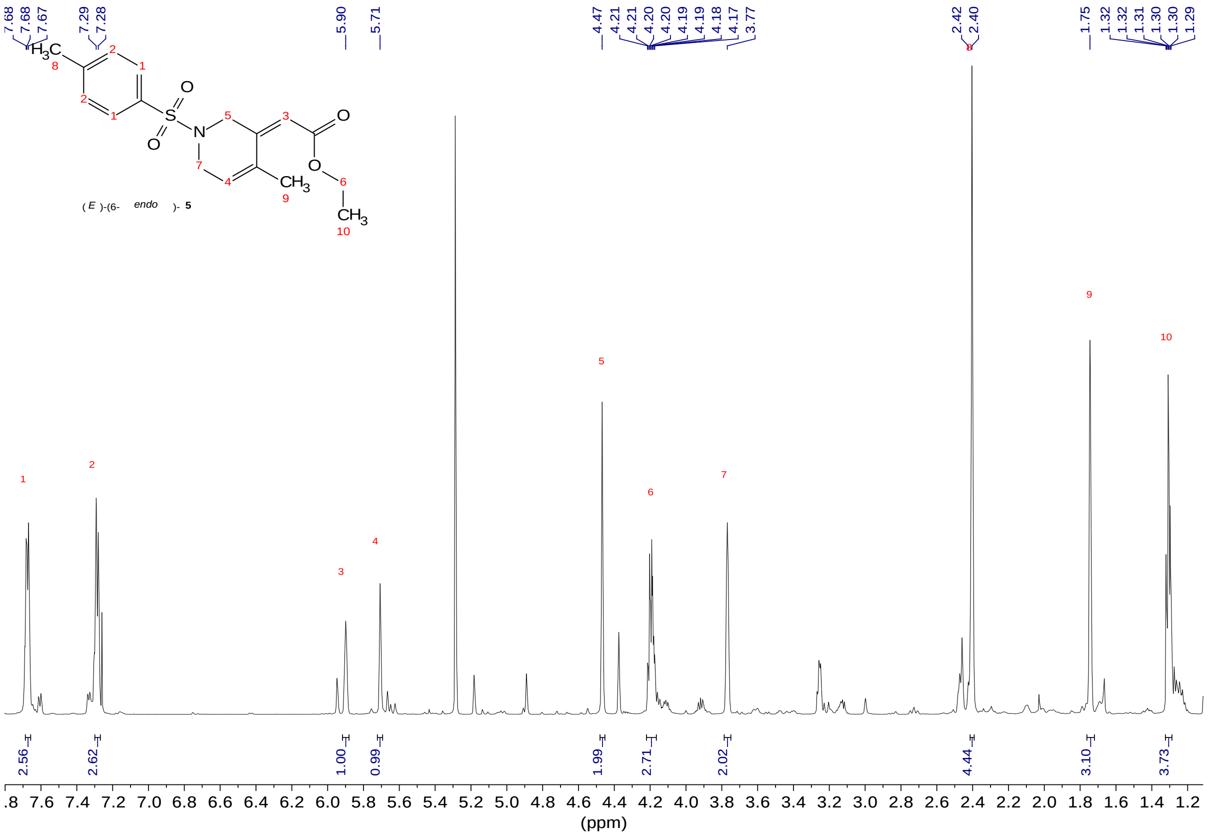
7.68
7.68
7.67
7.29
7.28
5.90
5.71
4.47
4.21
4.21
4.20
4.20
4.19
4.19
4.18
4.17
3.77
2.42
2.40
1.75
1.32
1.32
1.31
1.30
1.30
1.29
(E)-(6-endo)-5
2.56
2.62
1.00
0.99
1.99
2.71
2.02
4.44
3.10
3.73
(ppm)

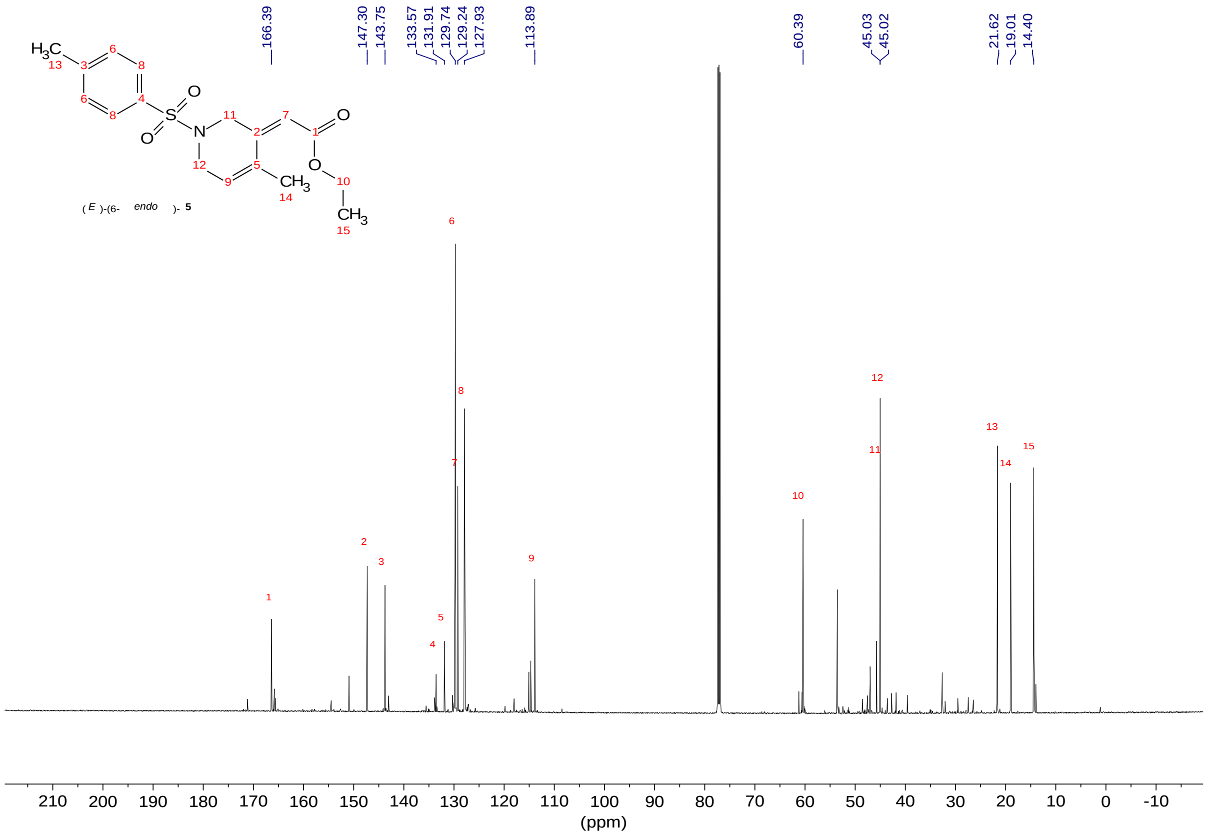
166.39
147.30
143.75
133.57
131.91
129.74
129.24
127.93
113.89
60.39
45.03
45.02
21.62
19.01
14.40
(E)-(6-endo)-5
(ppm)

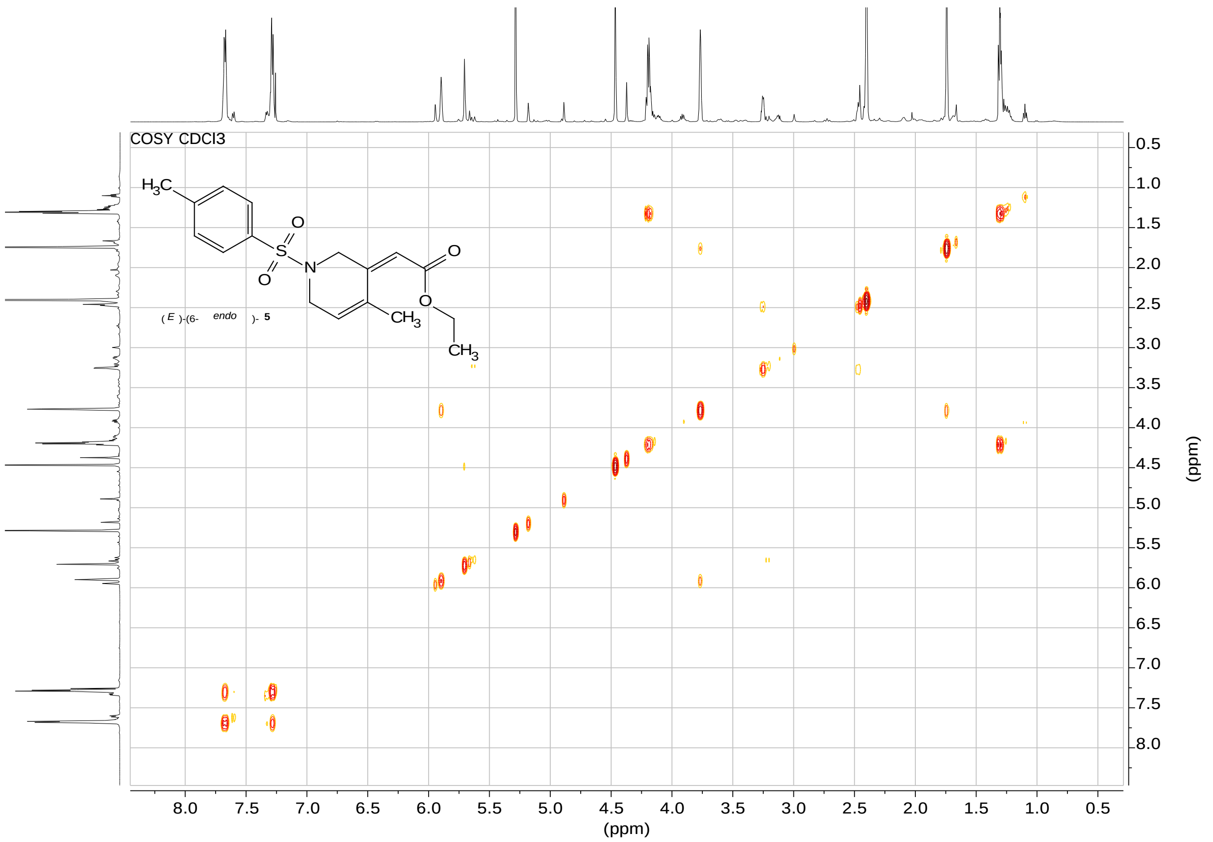
COSY CDCl3
H3C
O
S
N
O
O
O
CH3
CH3
(E)-(6-endo)-5
(ppm)
(ppm)

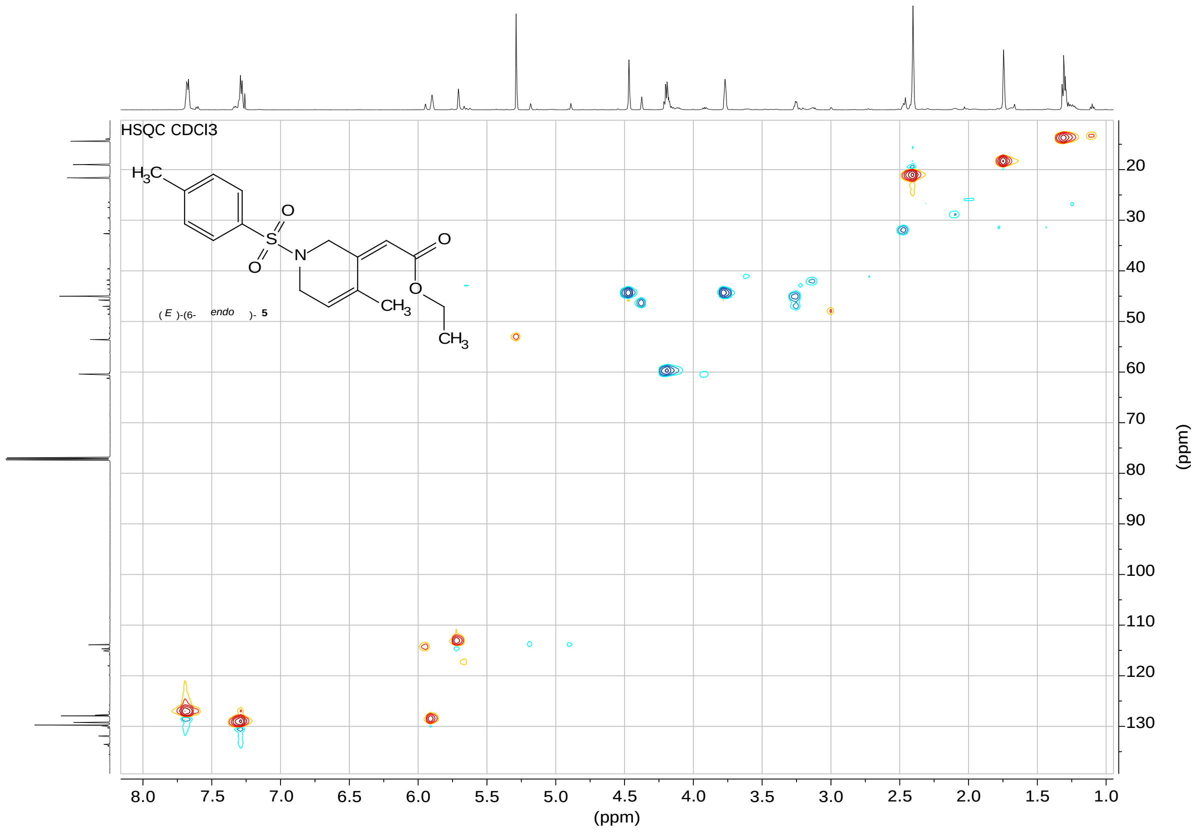
HSQC CDCl3
H3C
O
S
N
O
O
O
CH3
CH3
(E)-(6-endo)-5
(ppm)
(ppm)

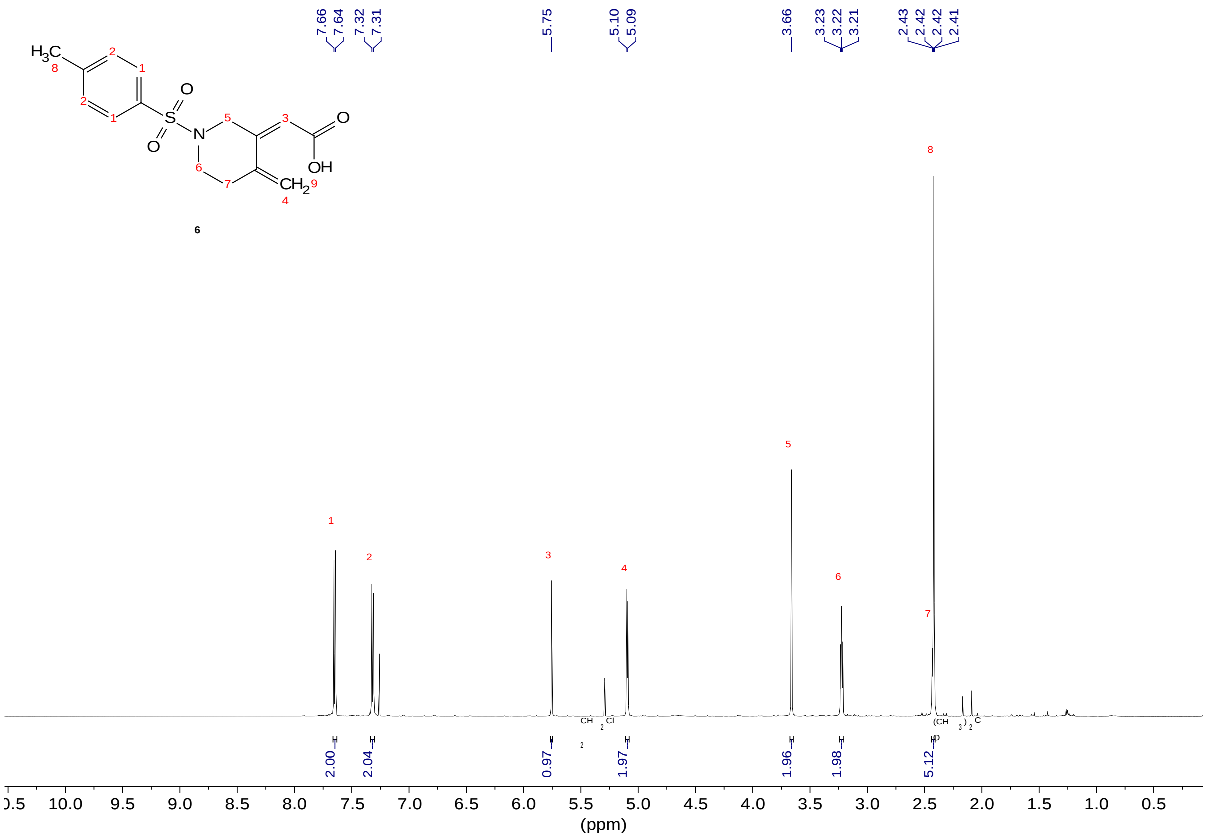
7.66
7.64
7.32
7.31
5.75
5.10
5.09
3.66
3.23
3.22
3.21
2.43
2.42
2.42
2.41
6
2.00
2.04
0.97
1.97
1.96
1.98
5.12
(ppm)

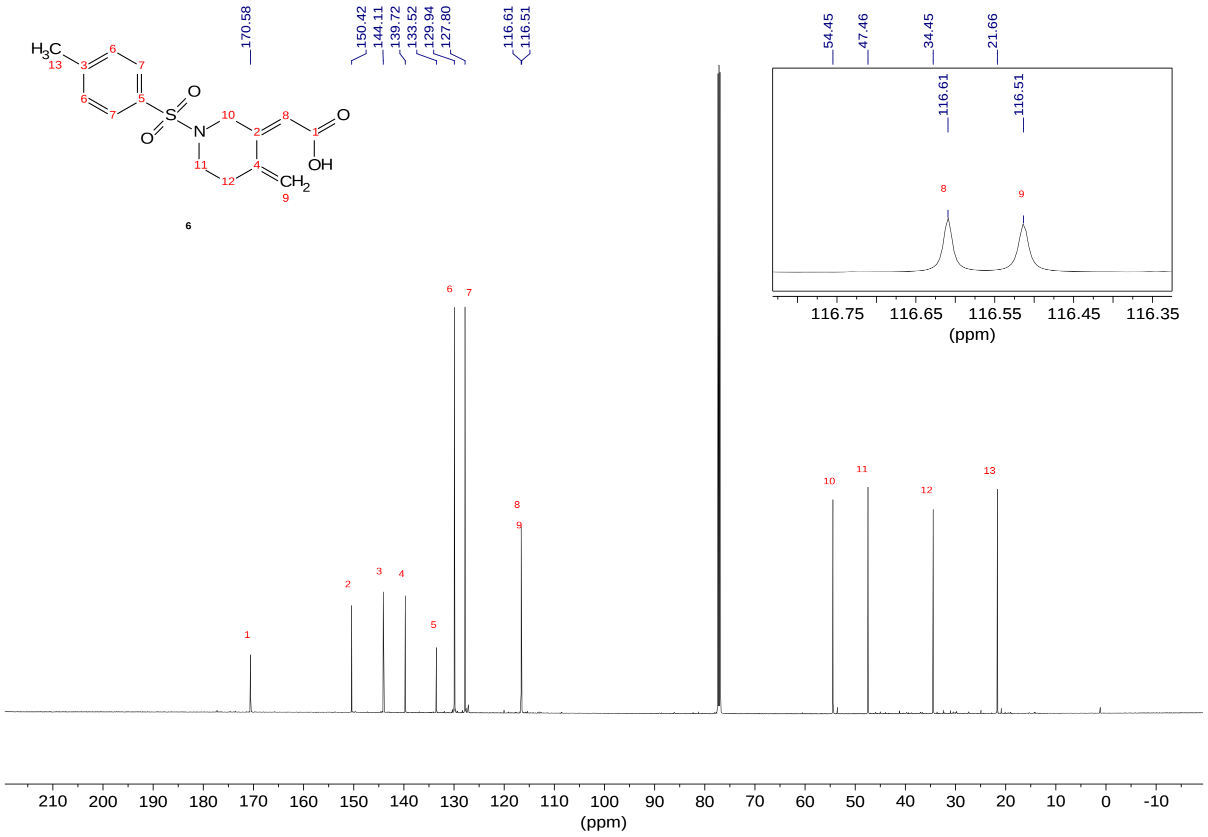
170.58
150.42
144.11
139.72
133.52
129.94
127.80
116.61
116.51
54.45
47.46
34.45
21.66
6
(ppm)

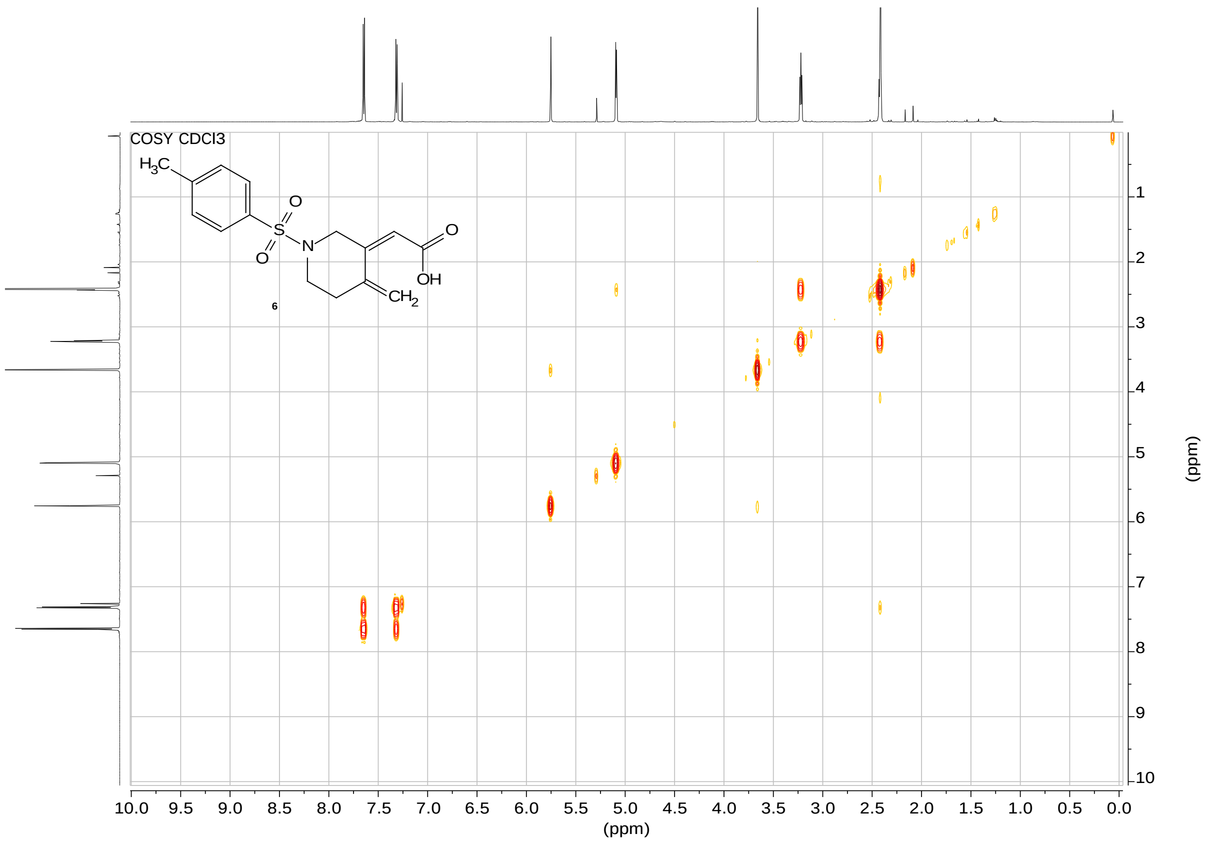
COSY CDCl3
H3C
O
S
O
N
O
OH
CH2
6
(ppm)
(ppm)

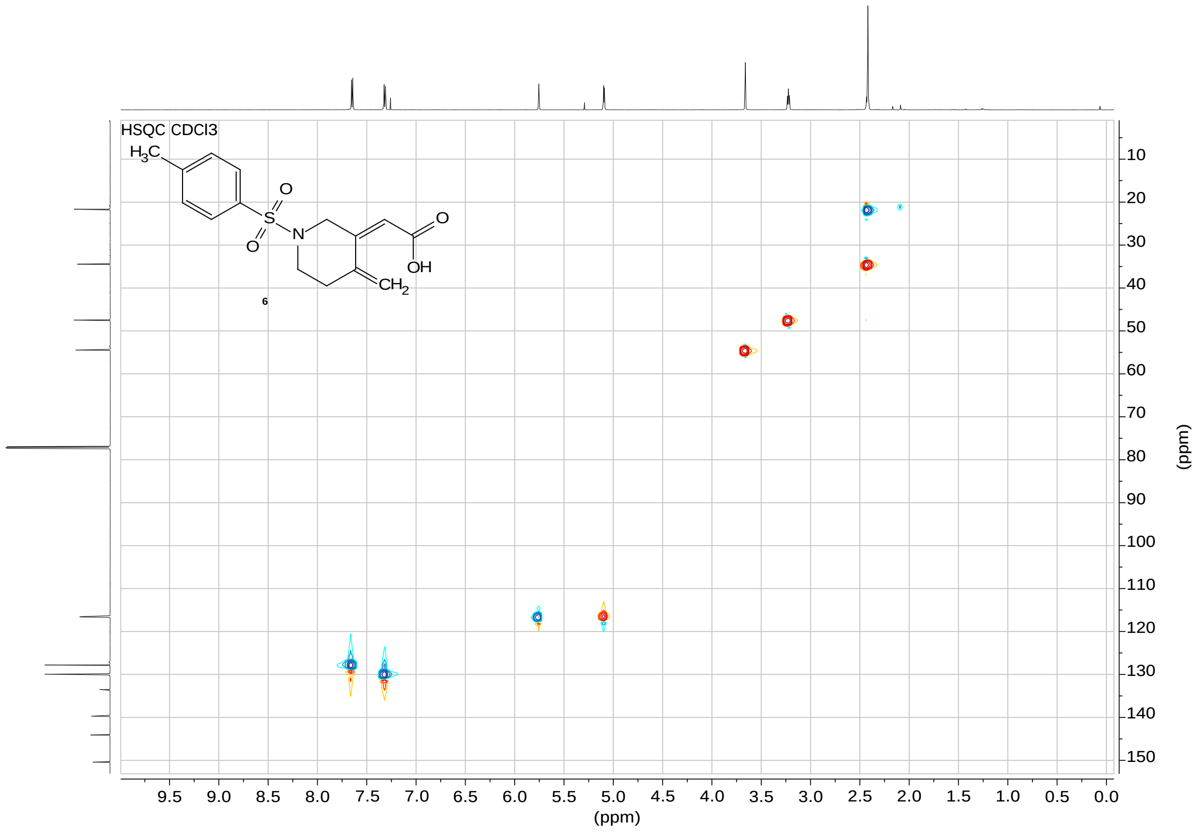
HSQC CDCl3
H3C
O
S
O
N
O
OH
CH2
6
(ppm)
(ppm)

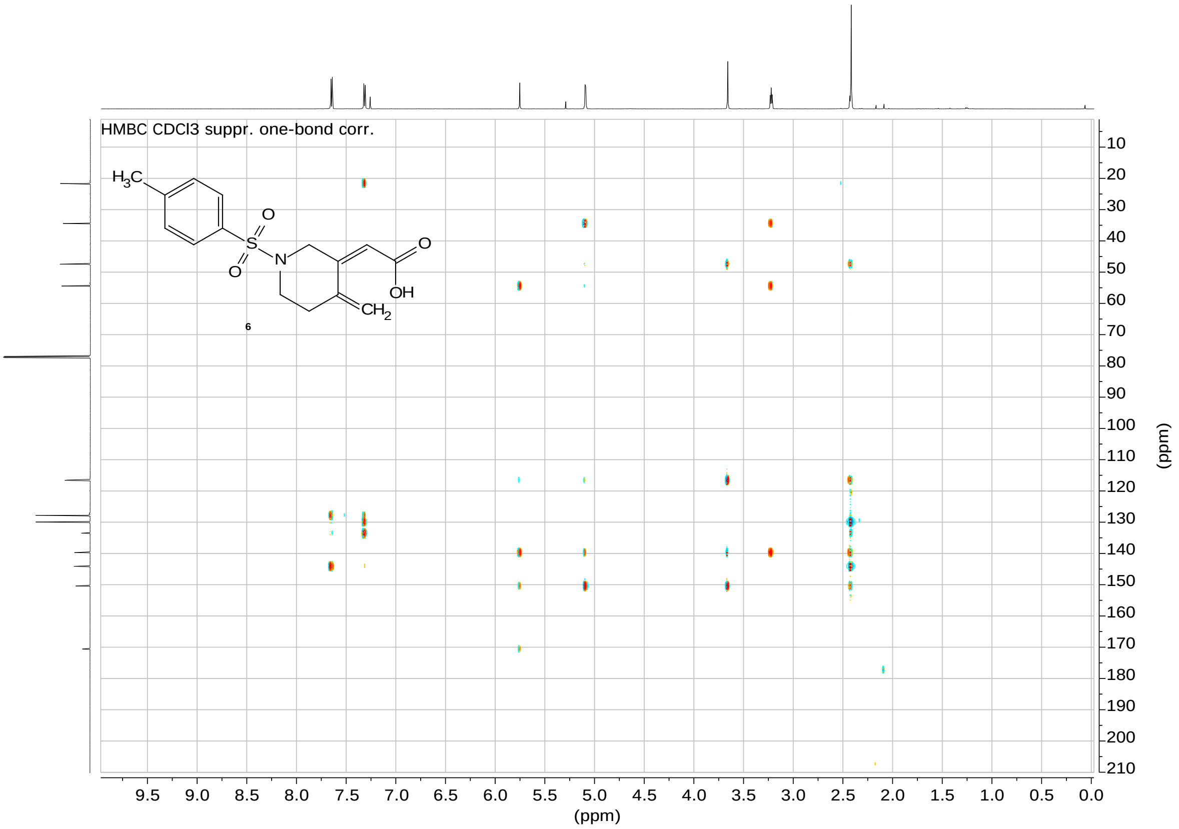
HMBC CDCl3 suppr. one-bond corr.
H3C
O
S
O
N
O
OH
CH2
6
9.5 9.0 8.5 8.0 7.5 7.0 6.5 6.0 5.5 5.0 4.5 4.0 3.5 3.0 2.5 2.0 1.5 1.0 0.5 0.0
(ppm)
10 20 30 40 50 60 70 80 90 100 110 120 130 140 150 160 170 180 190 200 210
(ppm)

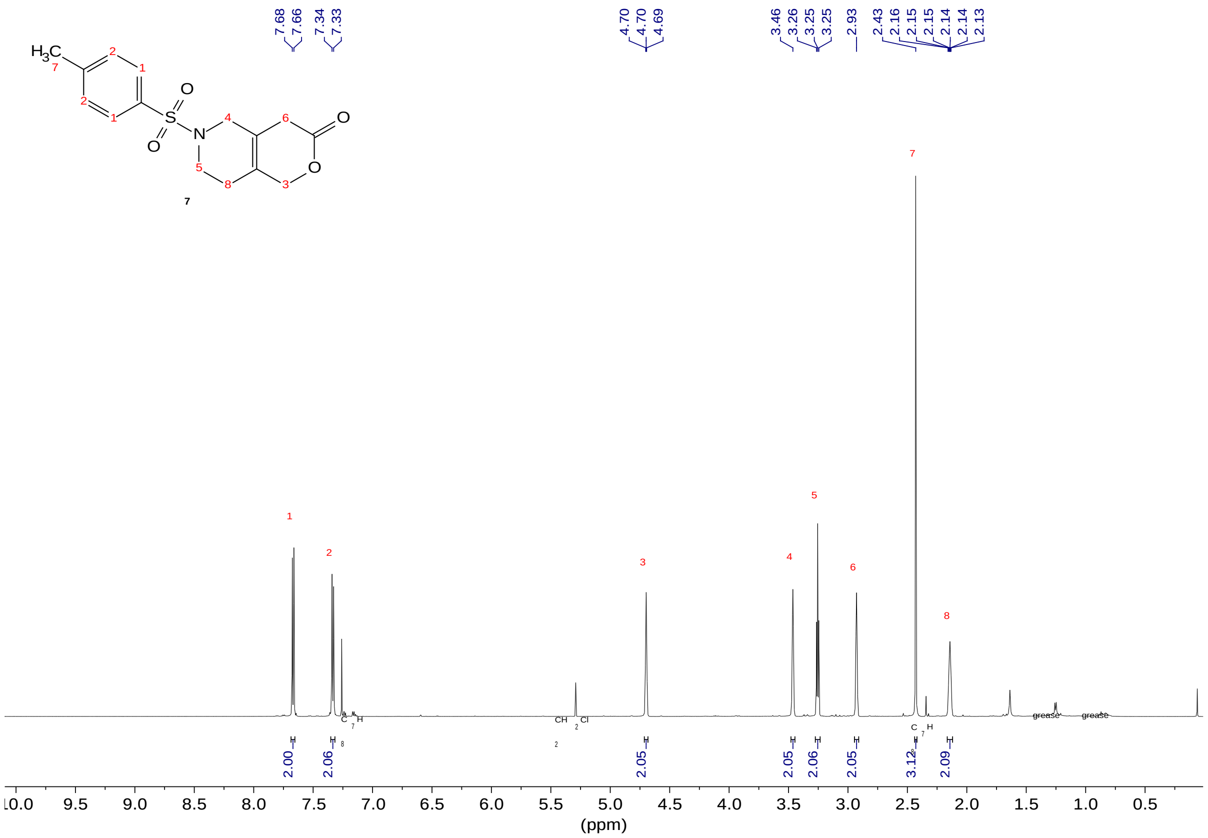

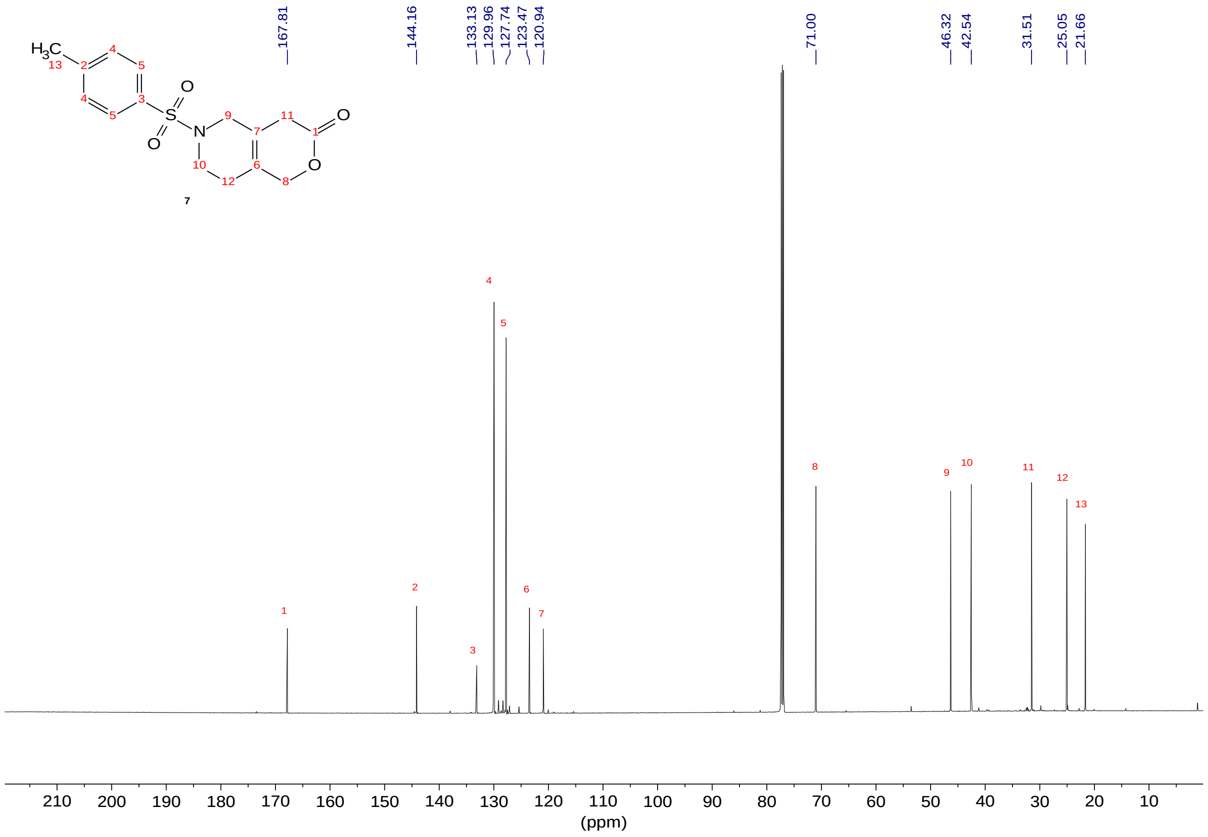

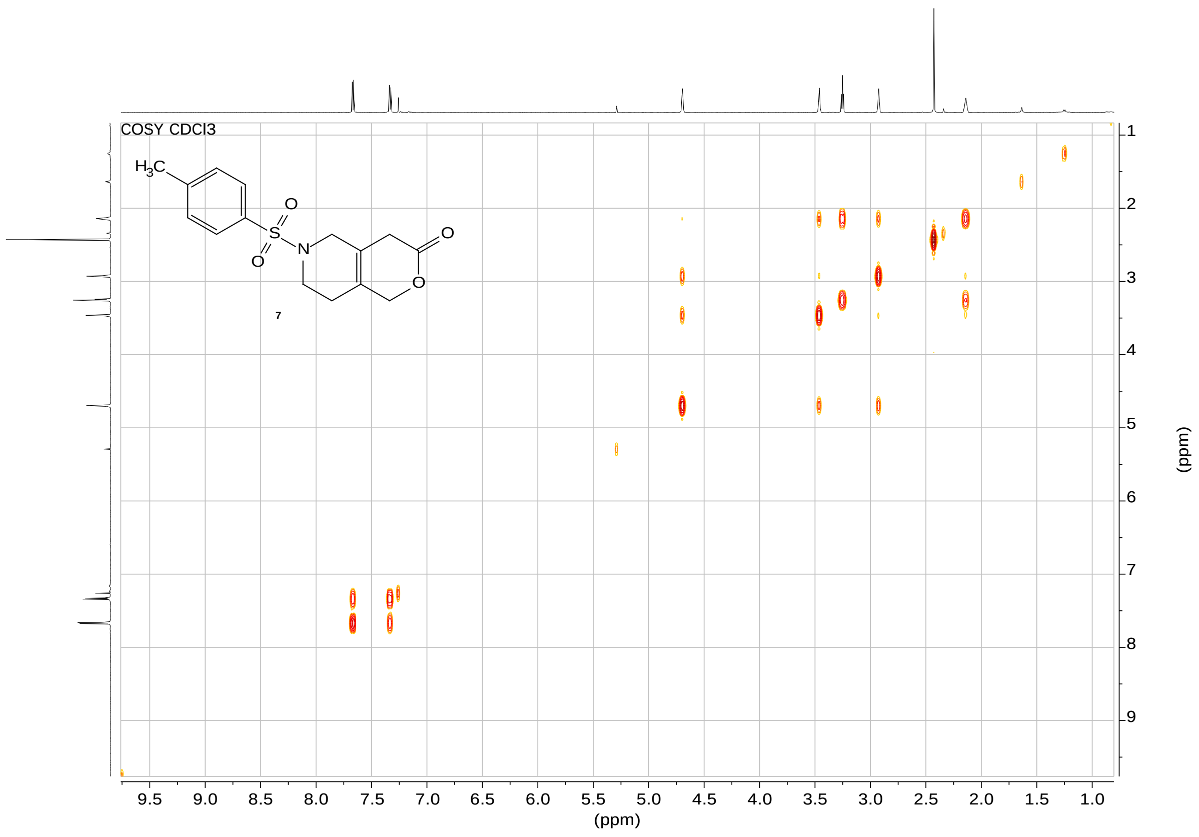
COSY CDCl3
H3C
O
S
O
N
O
O
7
(ppm)
(ppm)

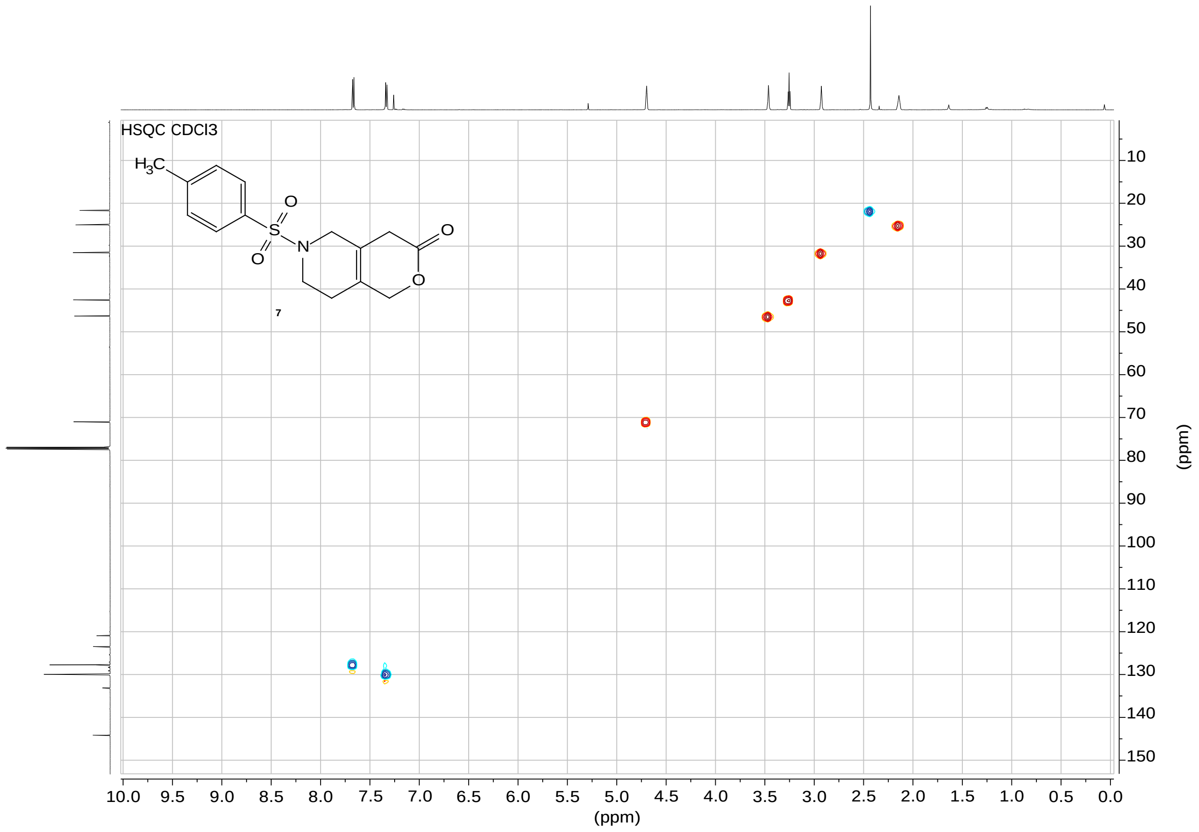
HSQC CDCl3
H3C
O
S
O
N
O
O
7
(ppm)
(ppm)

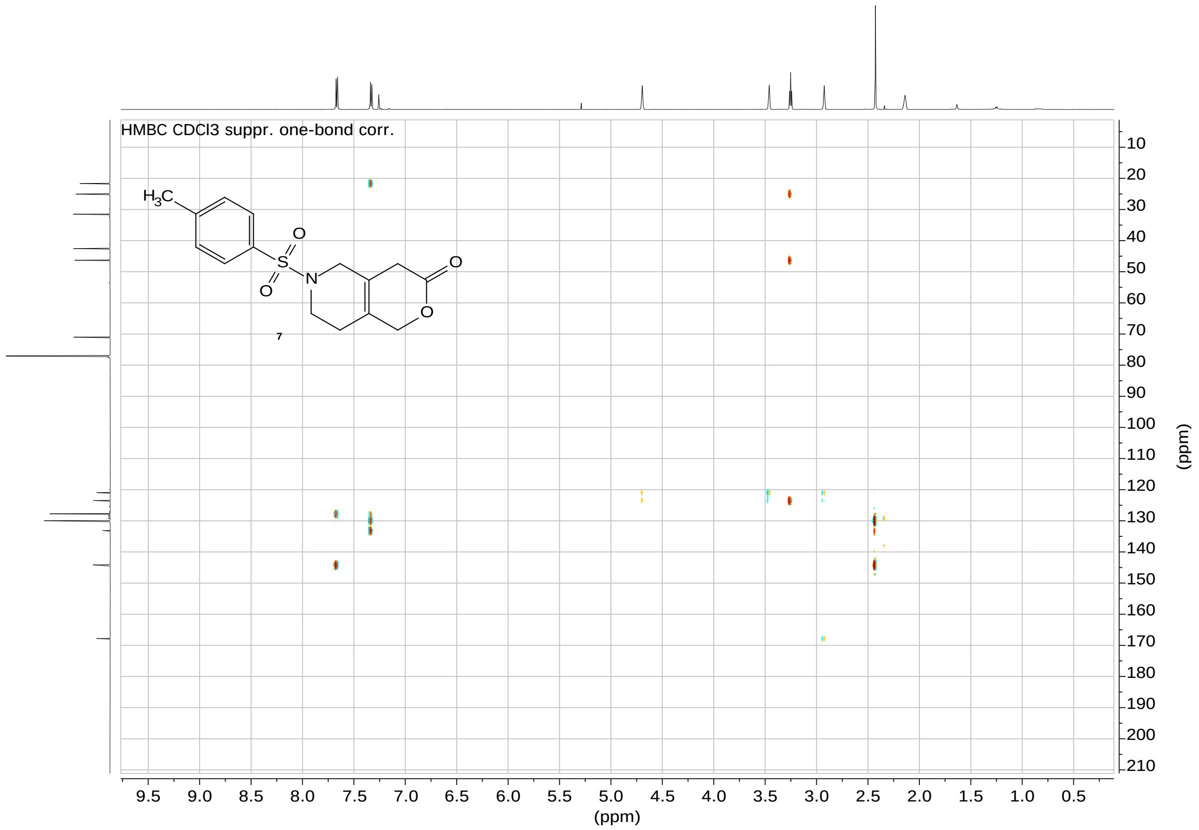

HMBC CDCl3 suppr. one-bond corr.
H3C
O
S
O
N
O
O
7
(ppm)
(ppm)

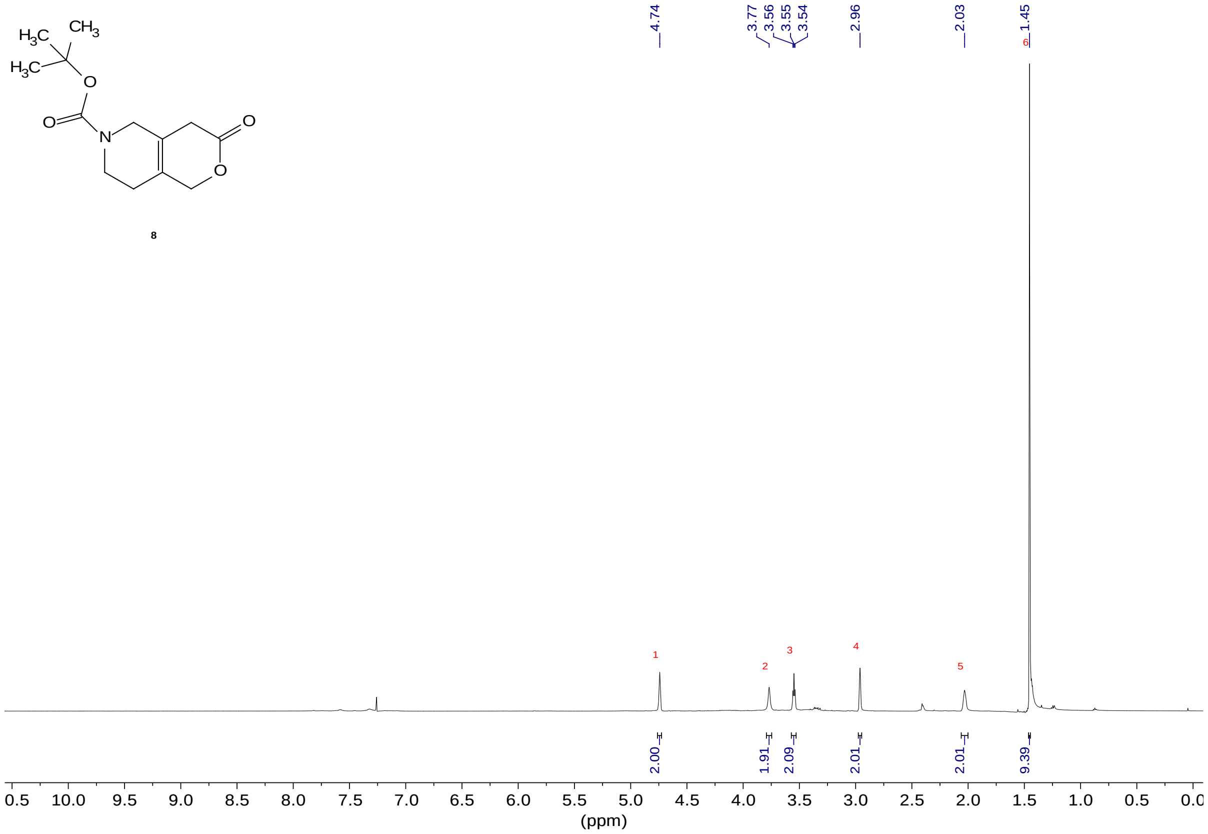

8
4.74
3.77
3.56
3.55
3.54
2.96
2.03
1.45
2.00
1.91
2.09
2.01
2.01
9.39
(ppm)

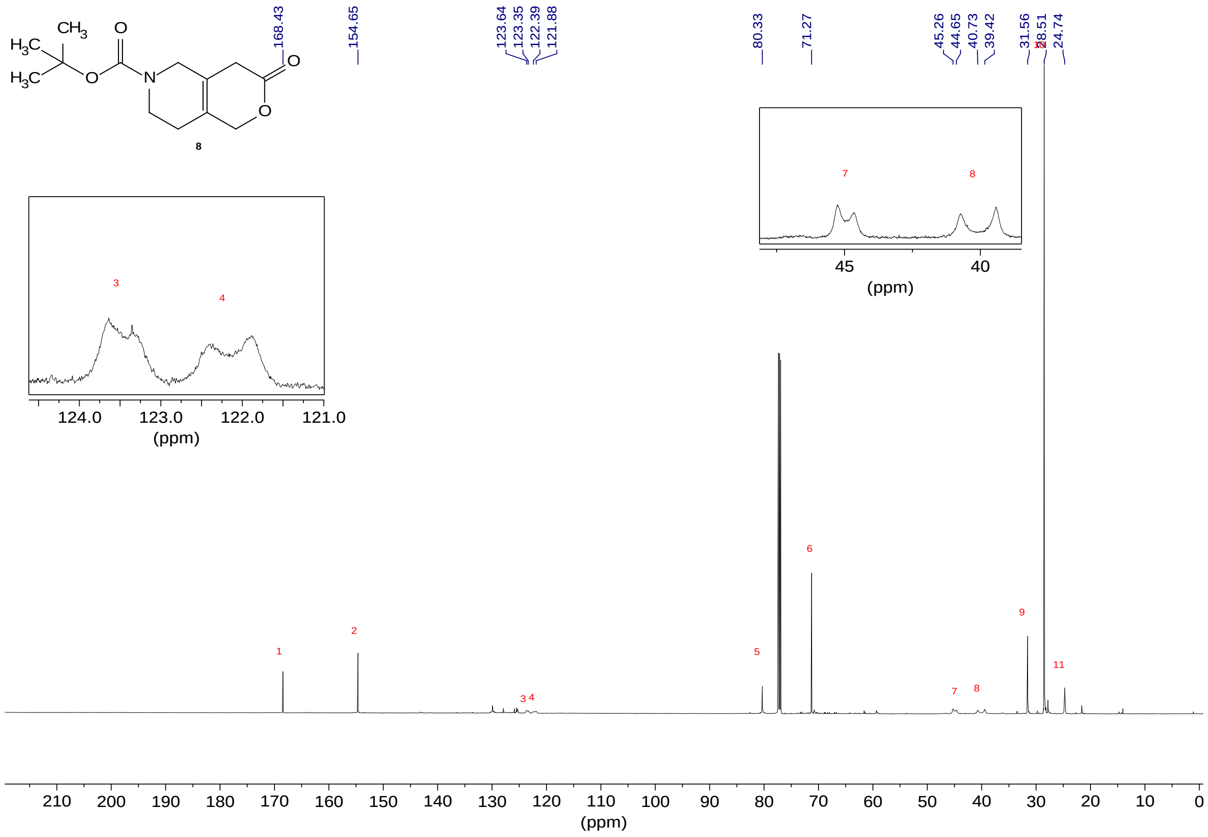

8
168.43
154.65
123.64
123.35
122.39
121.88
80.33
71.27
45.26
44.65
40.73
39.42
31.56
28.51
24.74
(ppm)

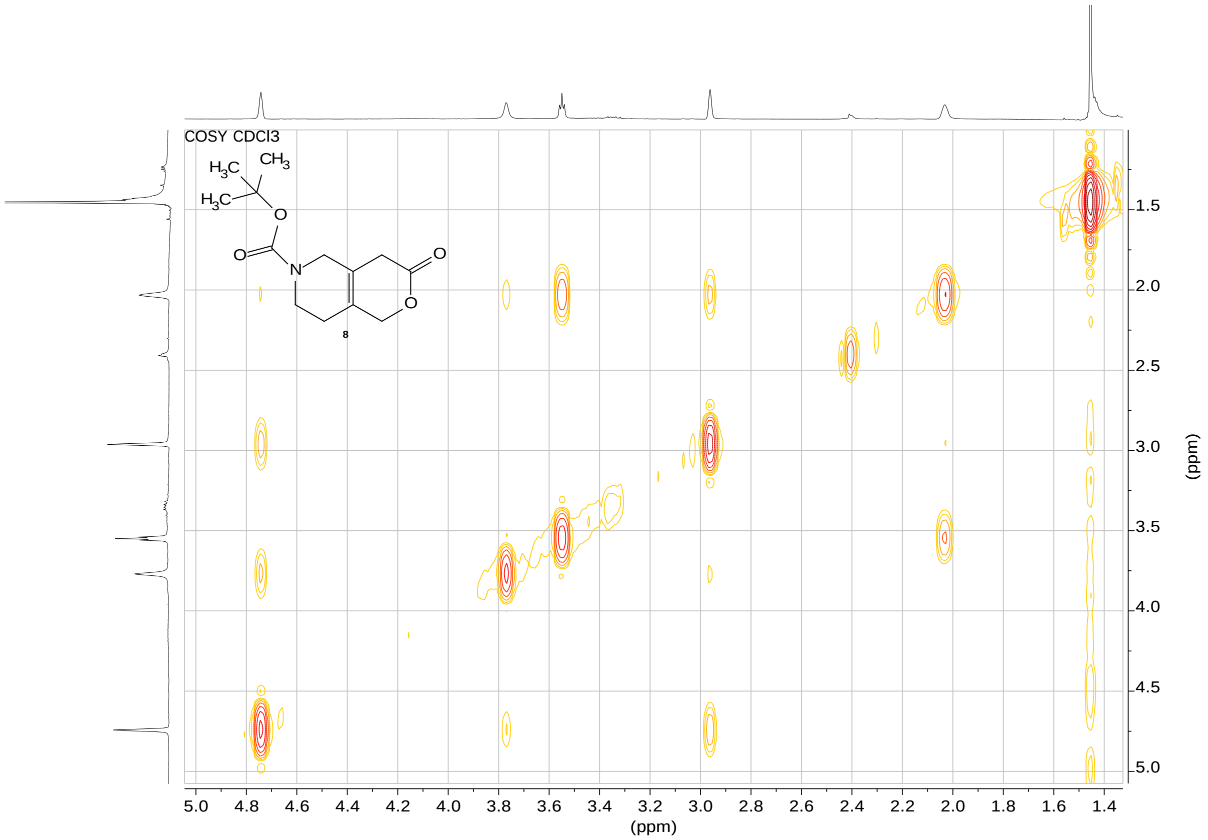
COSY CDCl3
H3C
CH3
H3C
O
O
N
O
O
8
(ppm)
(ppm)

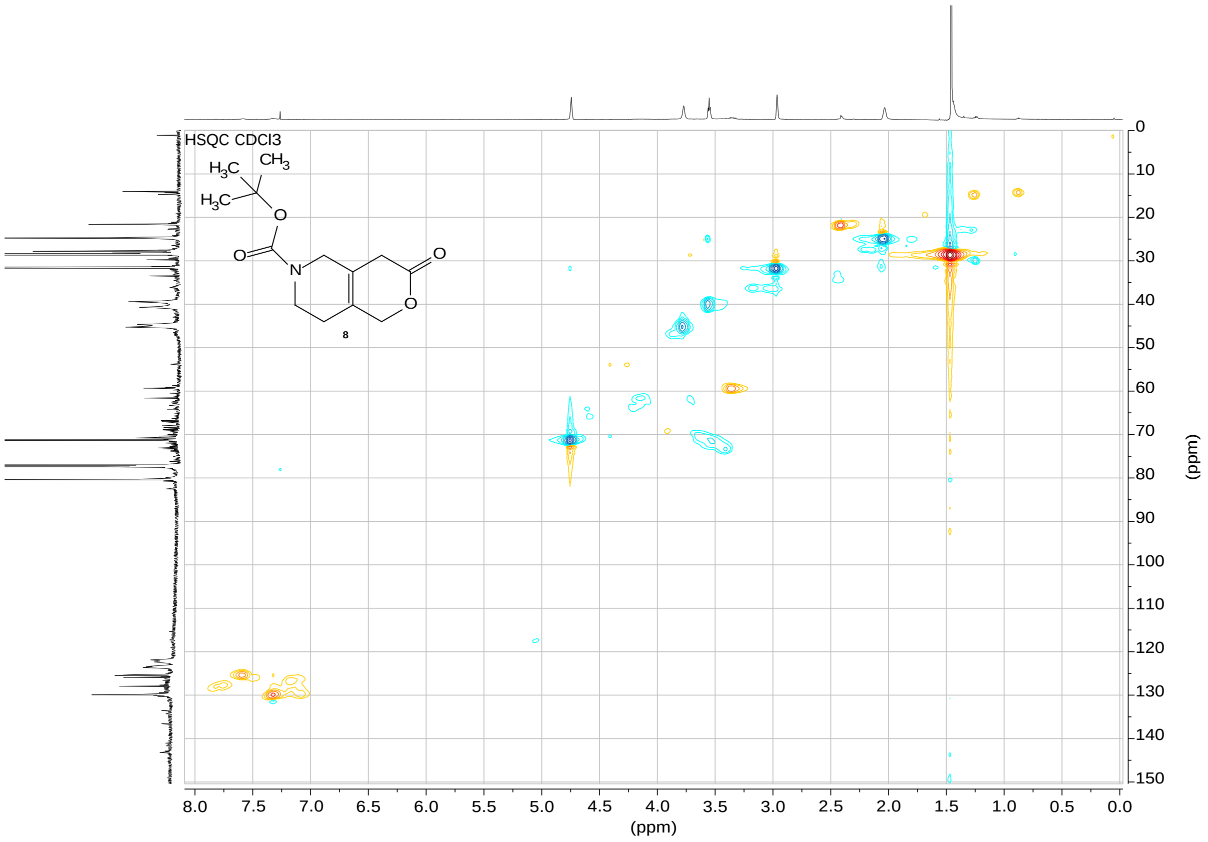
HSQC CDCl3
H3C
CH3
H3C
O
O
N
O
O
8
(ppm)
(ppm)

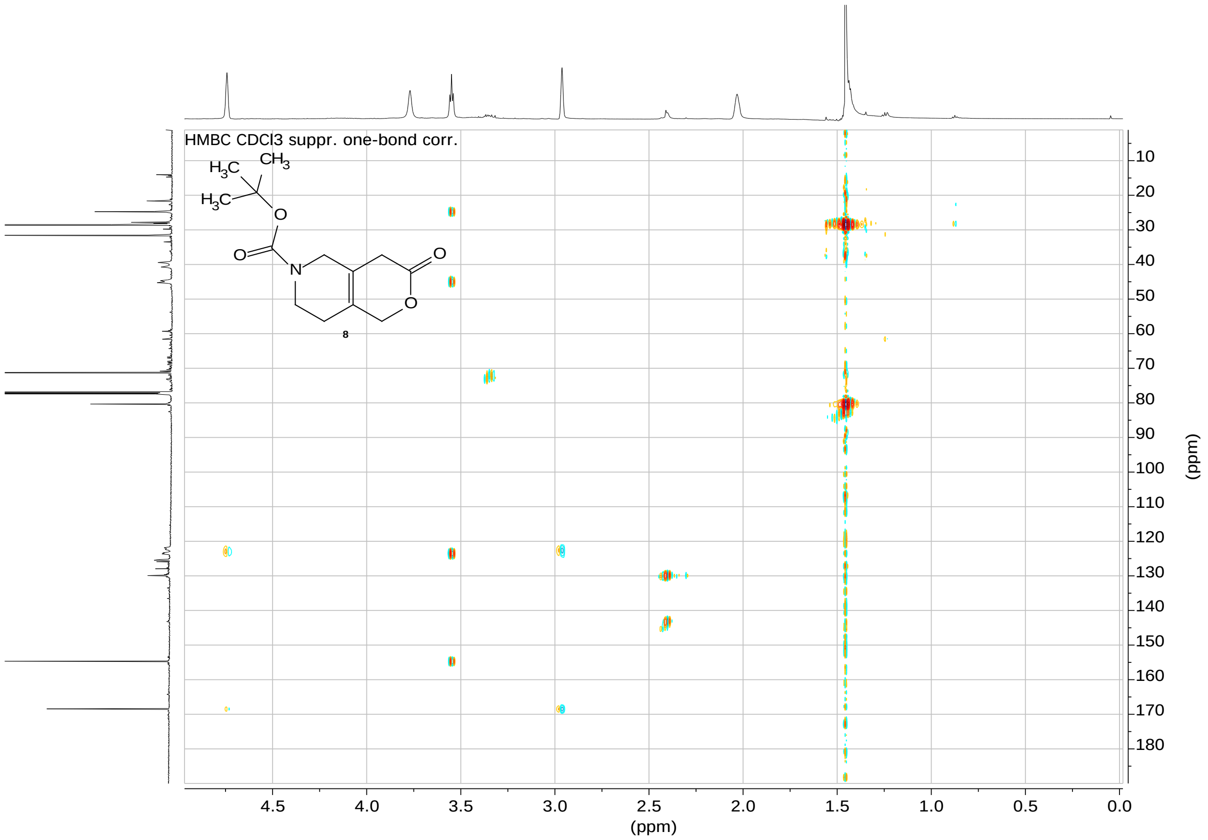
HMBC CDCl3 suppr. one-bond corr.
8
(ppm)
(ppm)

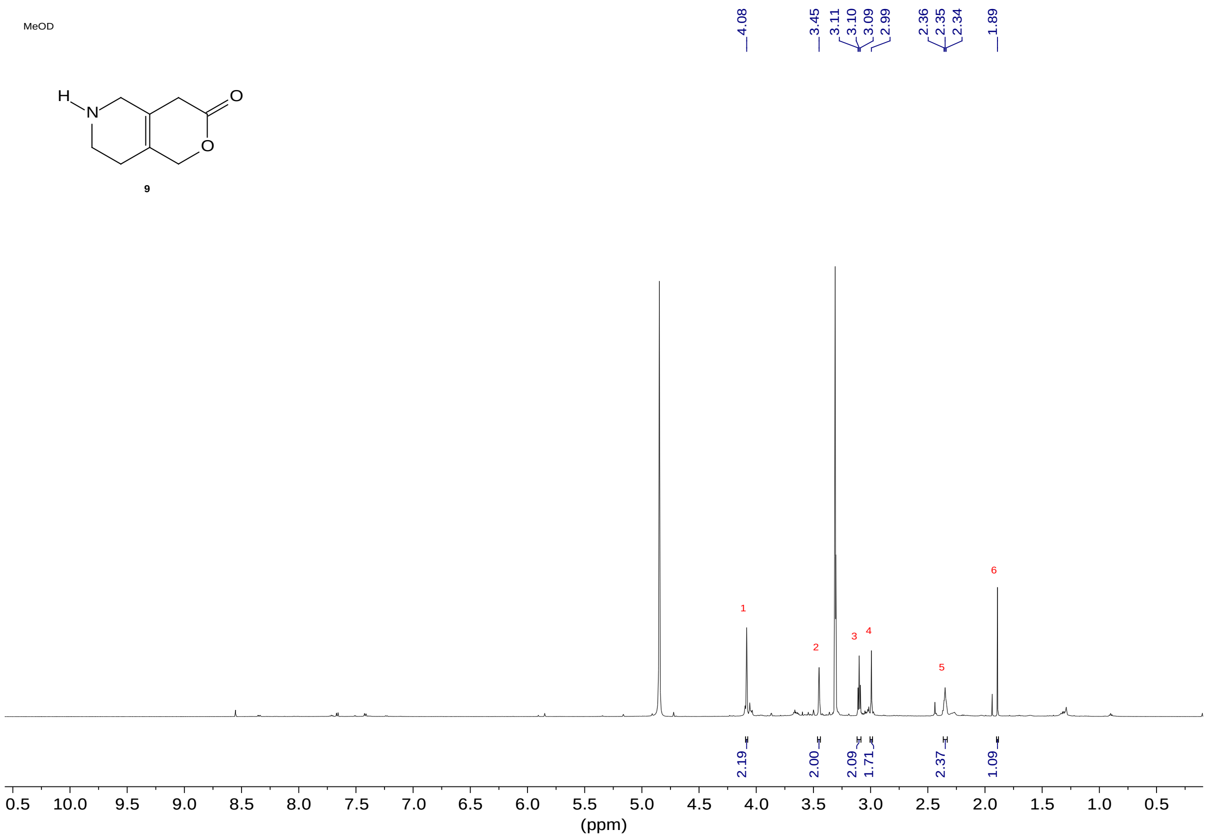
MeOD
4.08
3.45
3.11
3.10
3.09
2.99
2.36
2.35
2.34
1.89
9
2.19
2.00
2.09
1.71
2.37
1.09
(ppm)

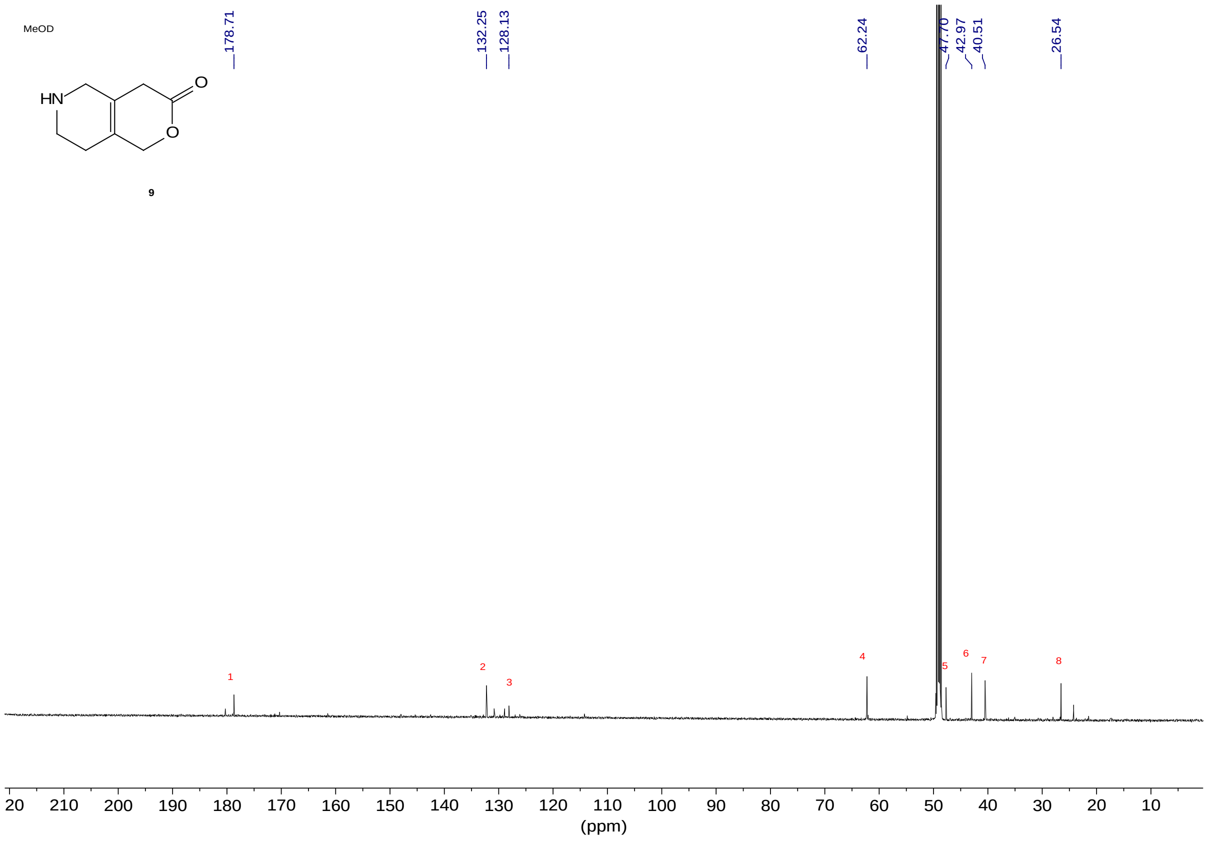
MeOD
178.71
132.25
128.13
62.24
47.70
42.97
40.51
26.54
9
(ppm)

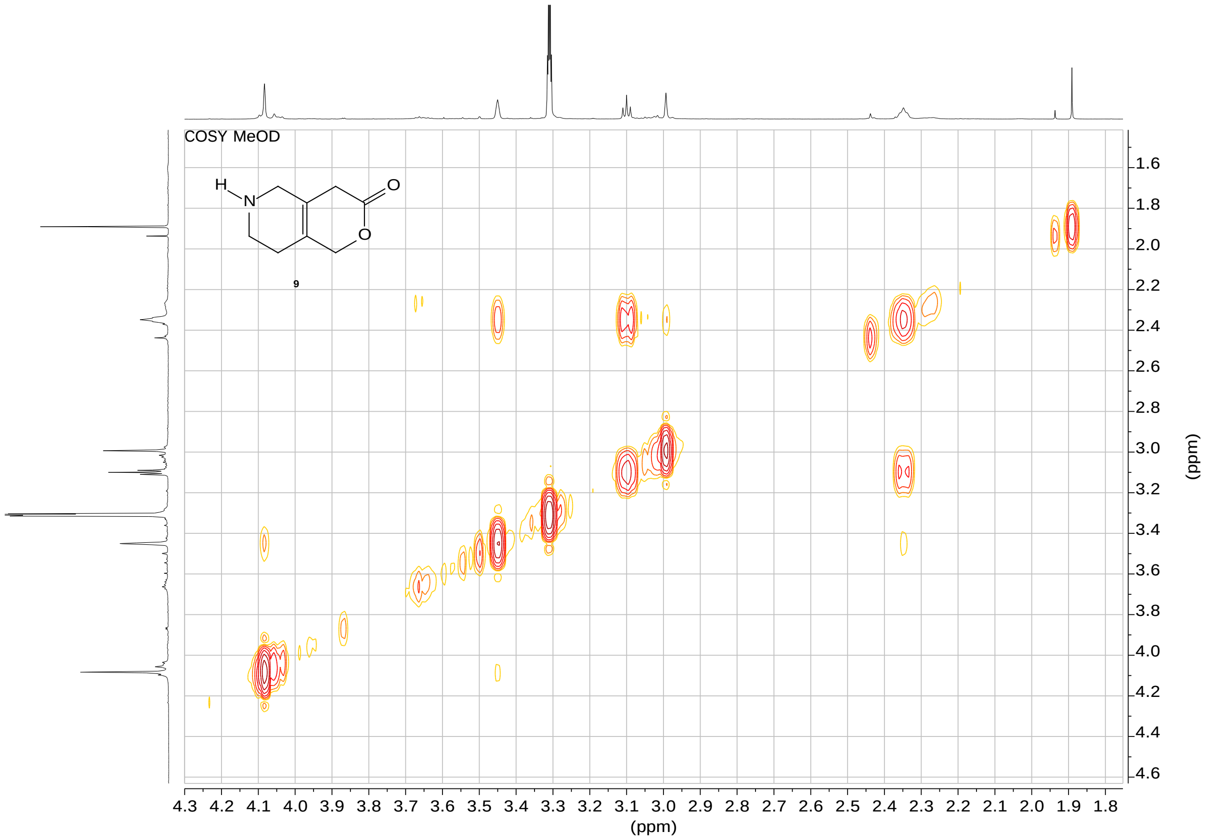
COSY MeOD
9
(ppm)
(ppm)

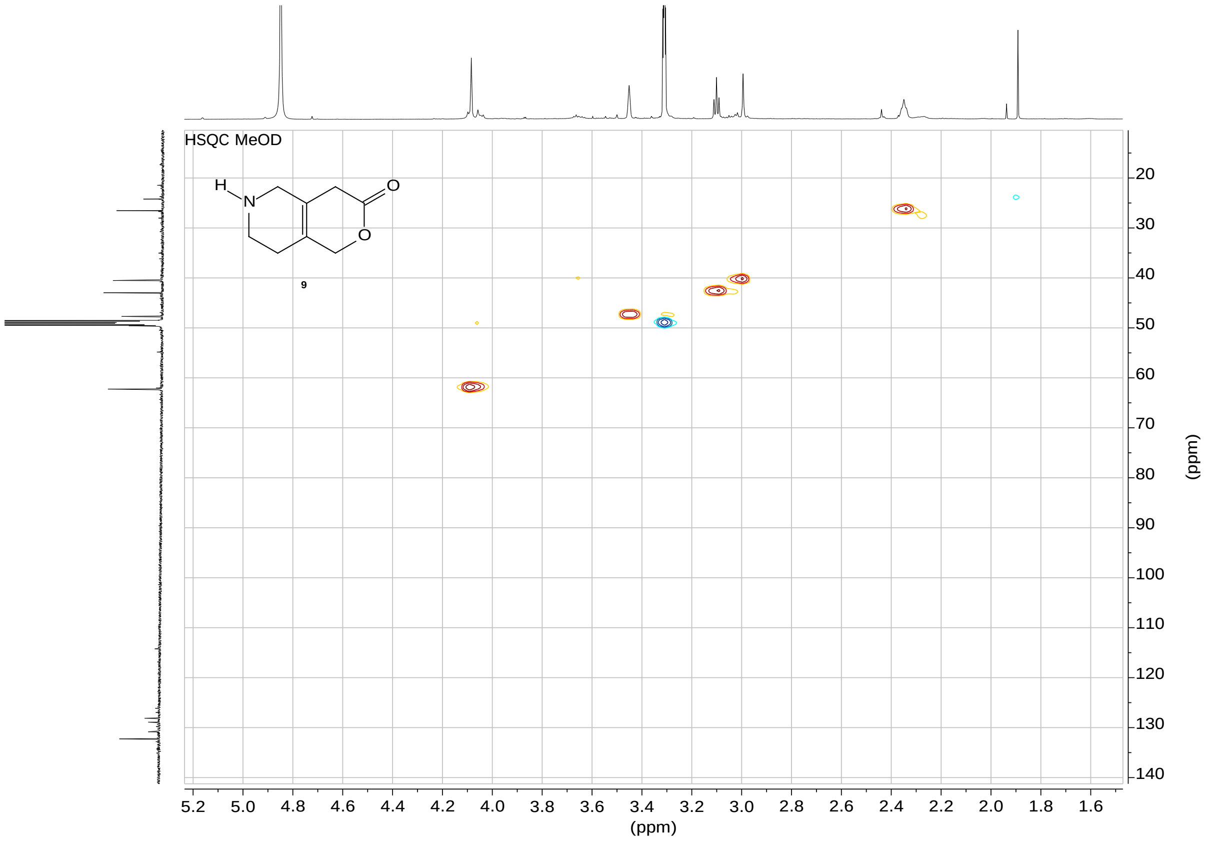
HSQC MeOD
9
(ppm)
(ppm)

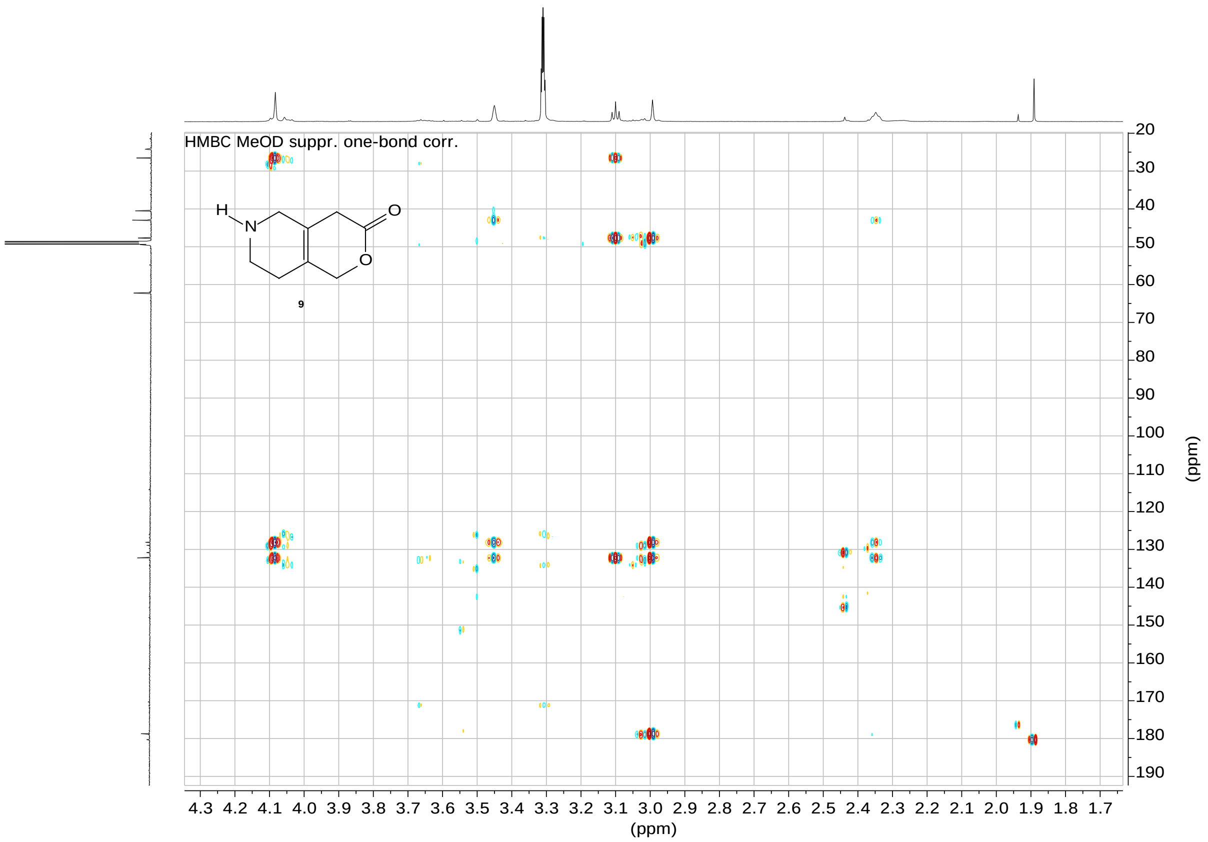
HMBC MeOD suppr. one-bond corr.
H
N
O
O
9
(ppm)
(ppm)

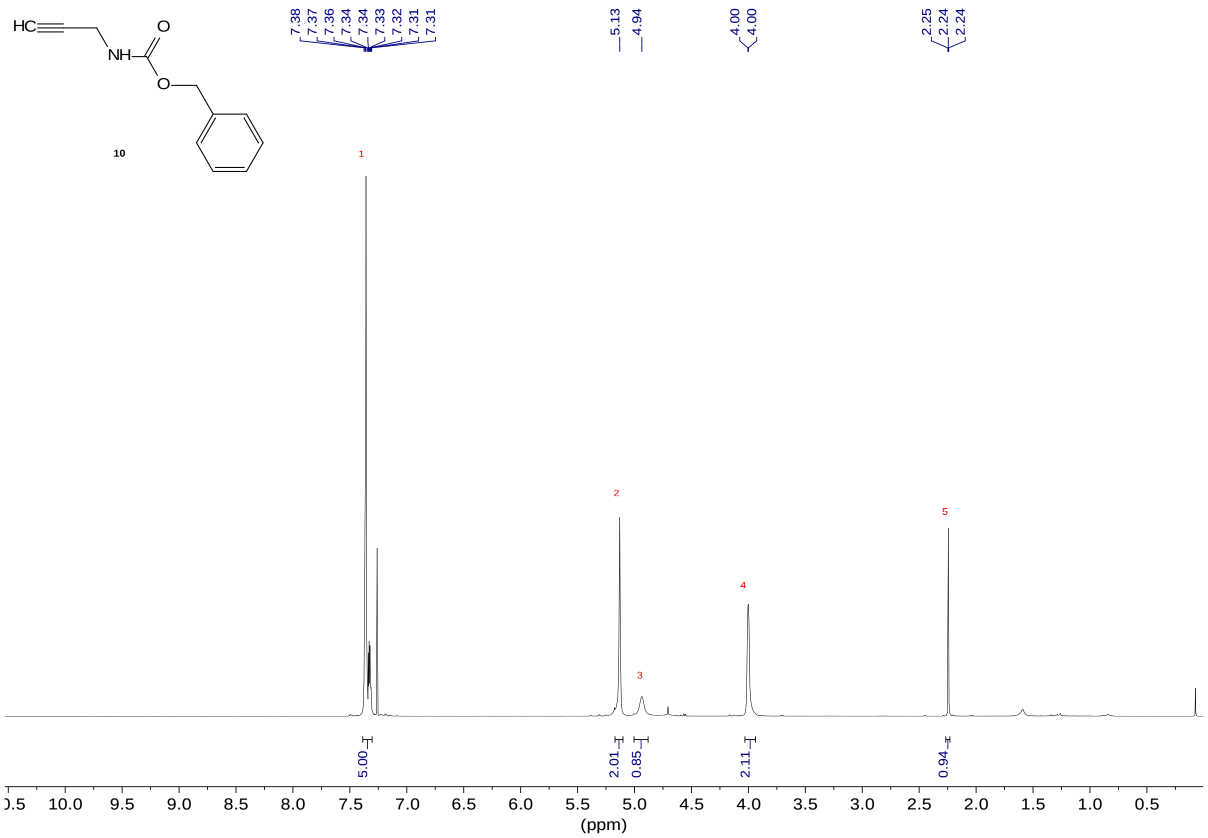
HC
NH
O
O
10
7.38
7.37
7.36
7.34
7.34
7.33
7.32
7.31
7.31
5.13
4.94
4.00
4.00
2.25
2.24
2.24
1
2
3
4
5
5.00
2.01
0.85
2.11
0.94
10.0 9.5 9.0 8.5 8.0 7.5 7.0 6.5 6.0 5.5 5.0 4.5 4.0 3.5 3.0 2.5 2.0 1.5 1.0 0.5
(ppm)

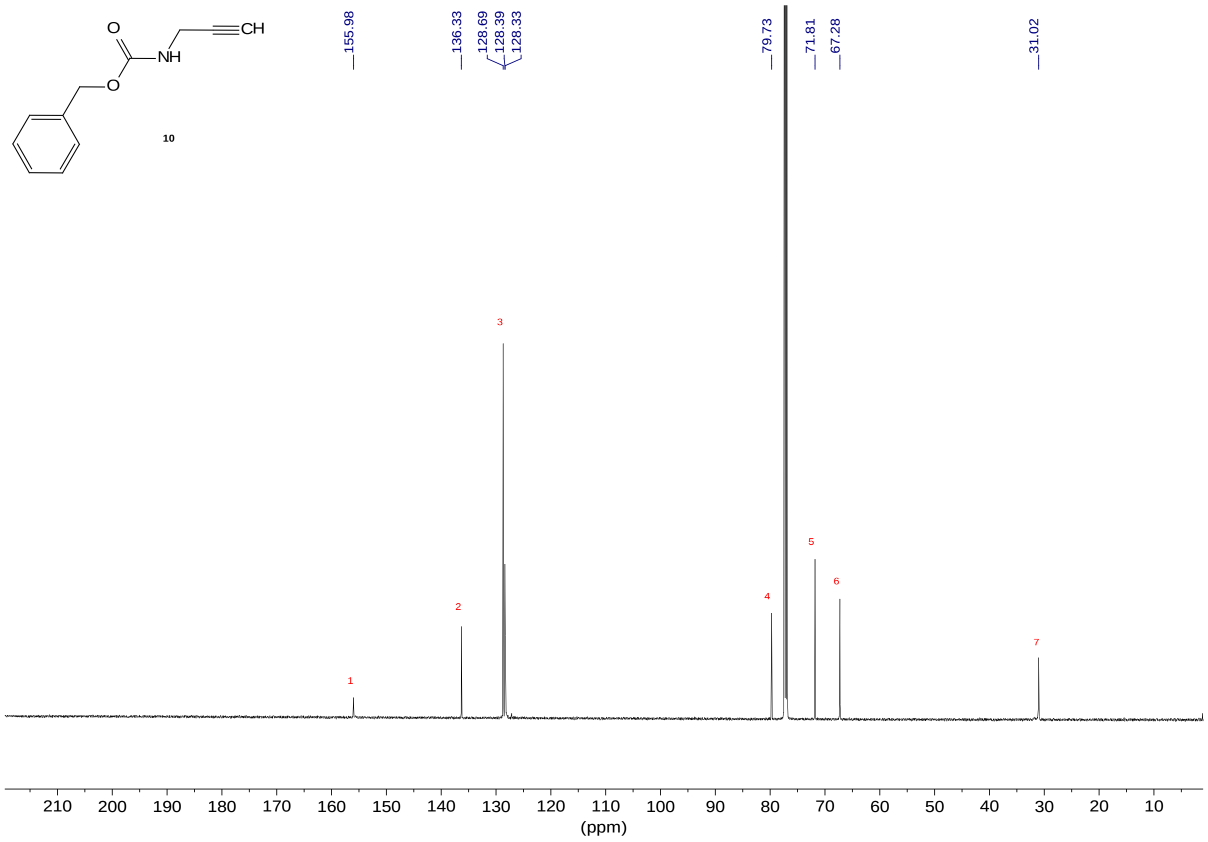
O
NH
CH
O
10
155.98
136.33
128.69
128.39
128.33
79.73
71.81
67.28
31.02
1
2
3
4
5
6
7
210 200 190 180 170 160 150 140 130 120 110 100 90 80 70 60 50 40 30 20 10
(ppm)

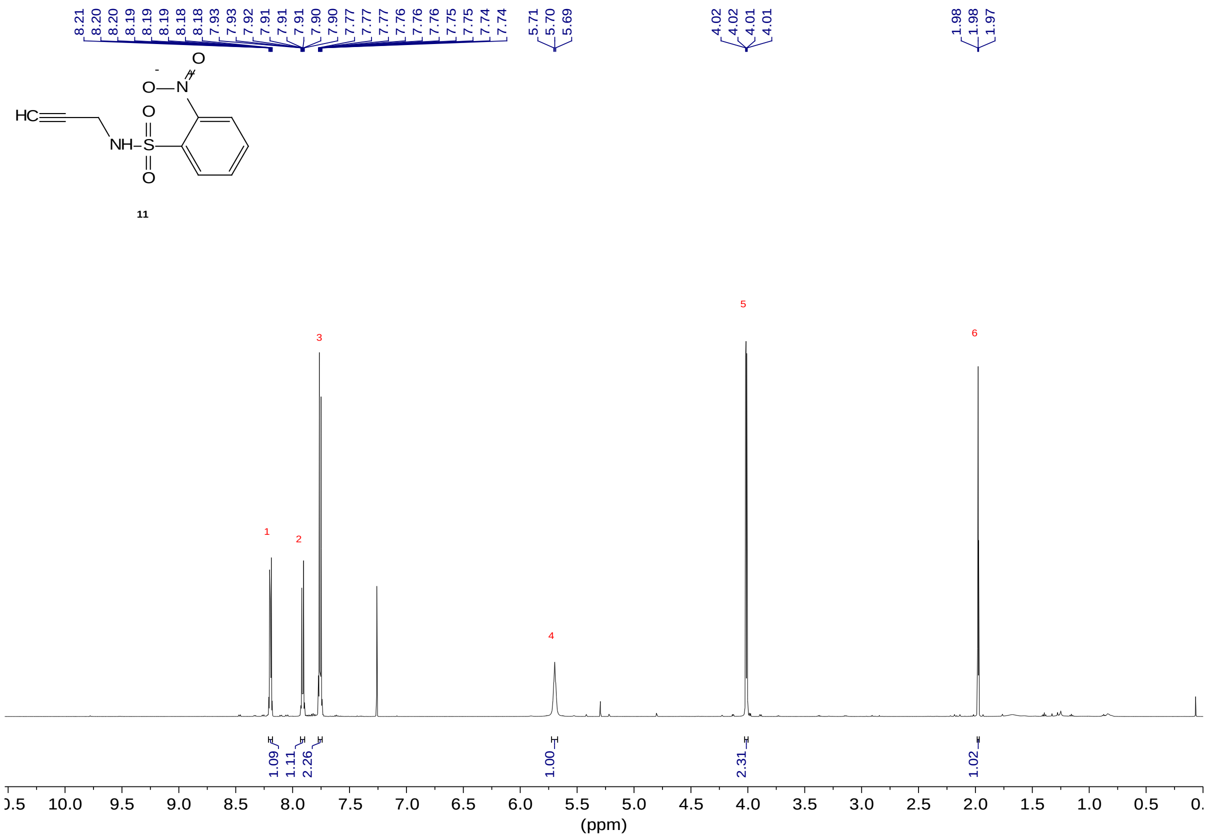

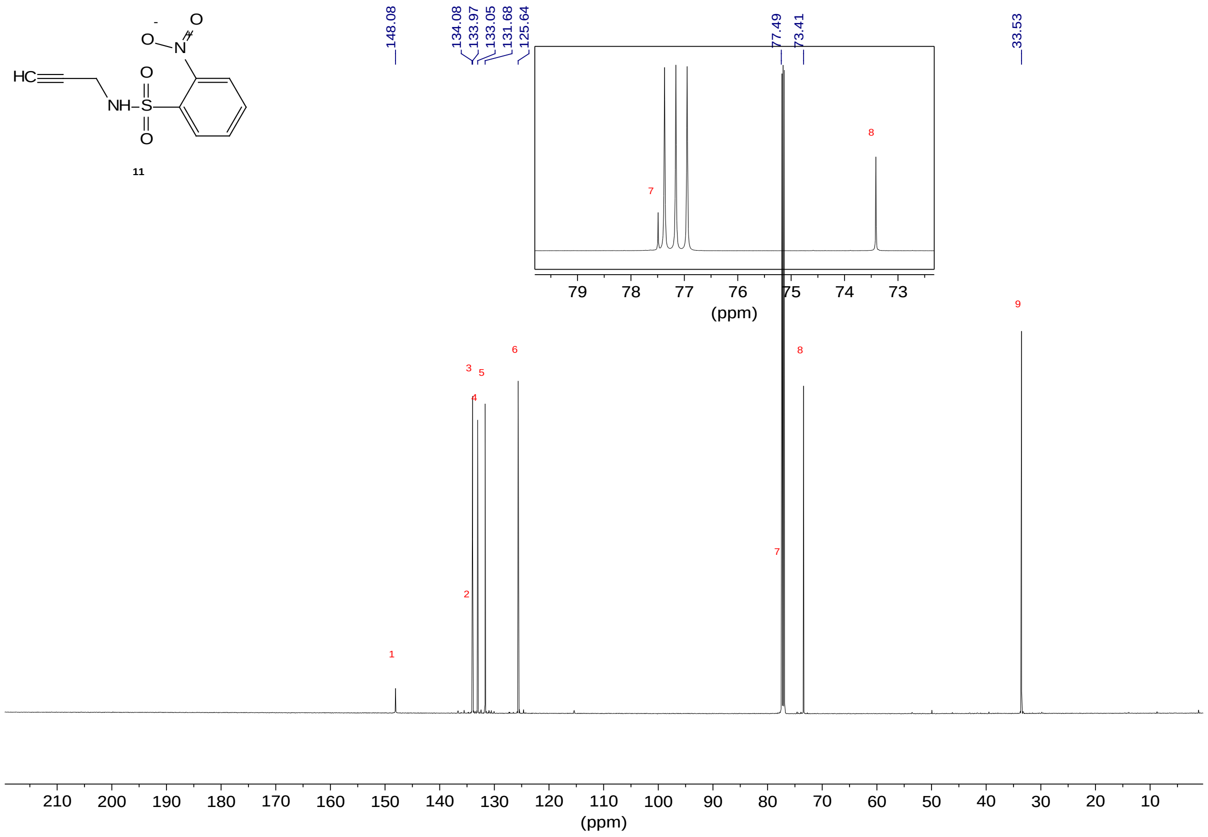

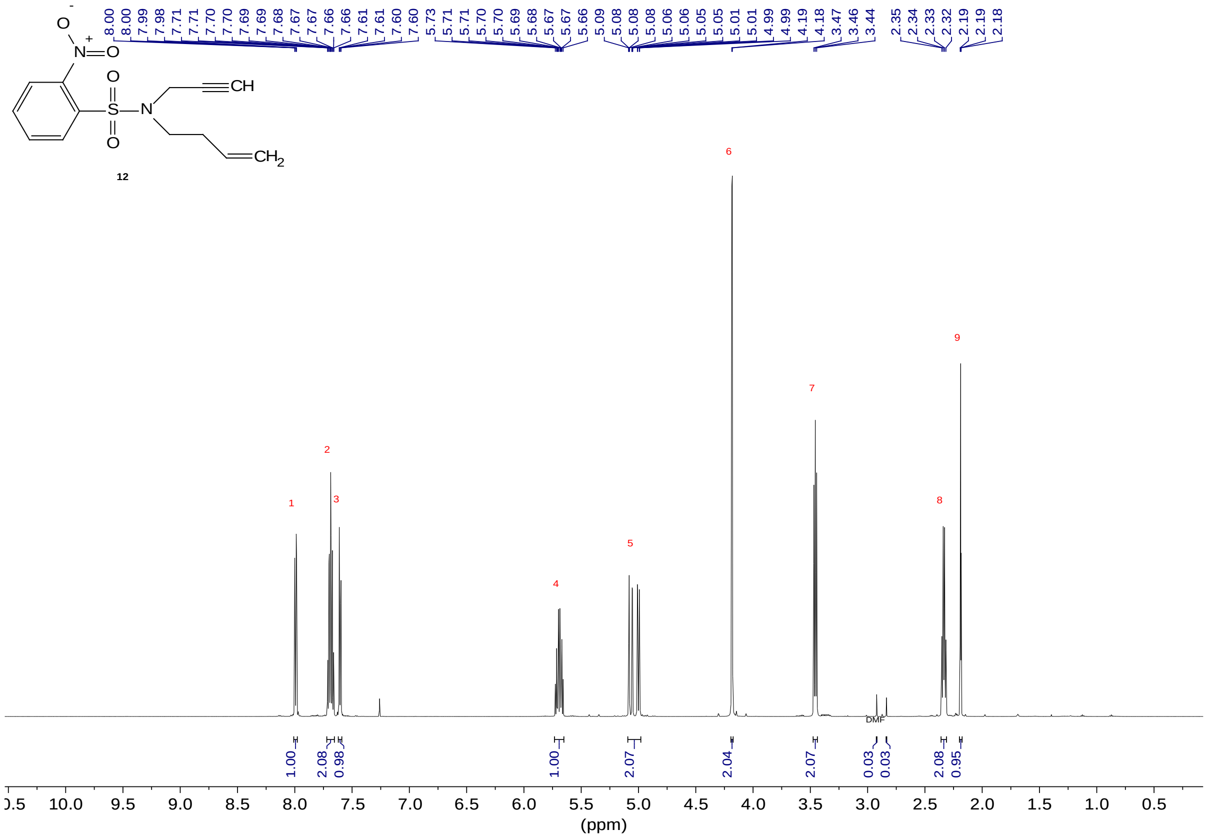
8.00 8.00 7.99 7.98 7.71 7.71 7.70 7.70 7.69 7.69 7.68 7.67 7.67 7.66 7.66 7.61 7.61 7.60 7.60 5.73 5.71 5.71 5.70 5.70 5.69 5.68 5.67 5.67 5.66 5.09 5.08 5.08 5.08 5.06 5.06 5.05 5.05 5.01 5.01 4.99 4.99 4.19 4.18 3.47 3.46 3.44 2.35 2.34 2.33 2.32 2.19 2.19 2.18
12
DMF
1.00 2.08 0.98 1.00 2.07 2.04 2.07 0.03 0.03 2.08 0.95
(ppm)

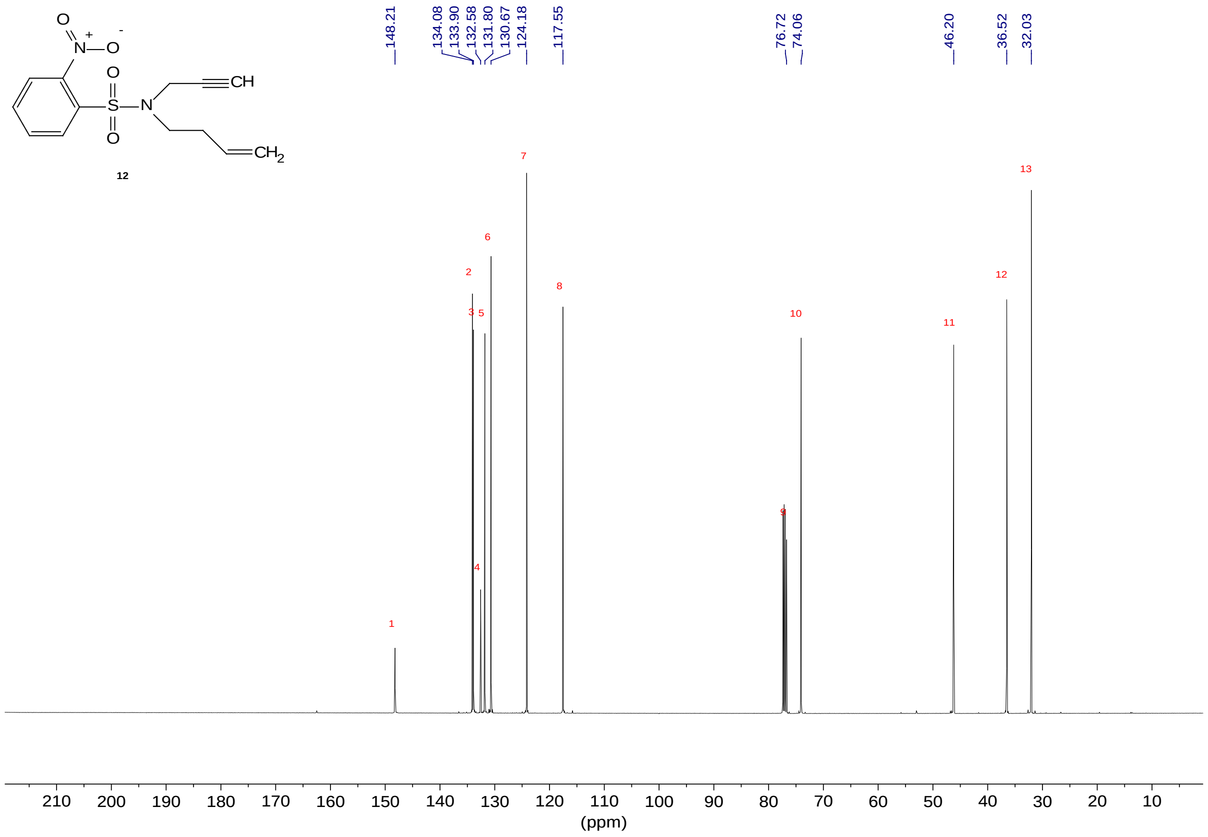
148.21 134.08 133.90 132.58 131.80 130.67 124.18 117.55 76.72 74.06 46.20 36.52 32.03
12
(ppm)

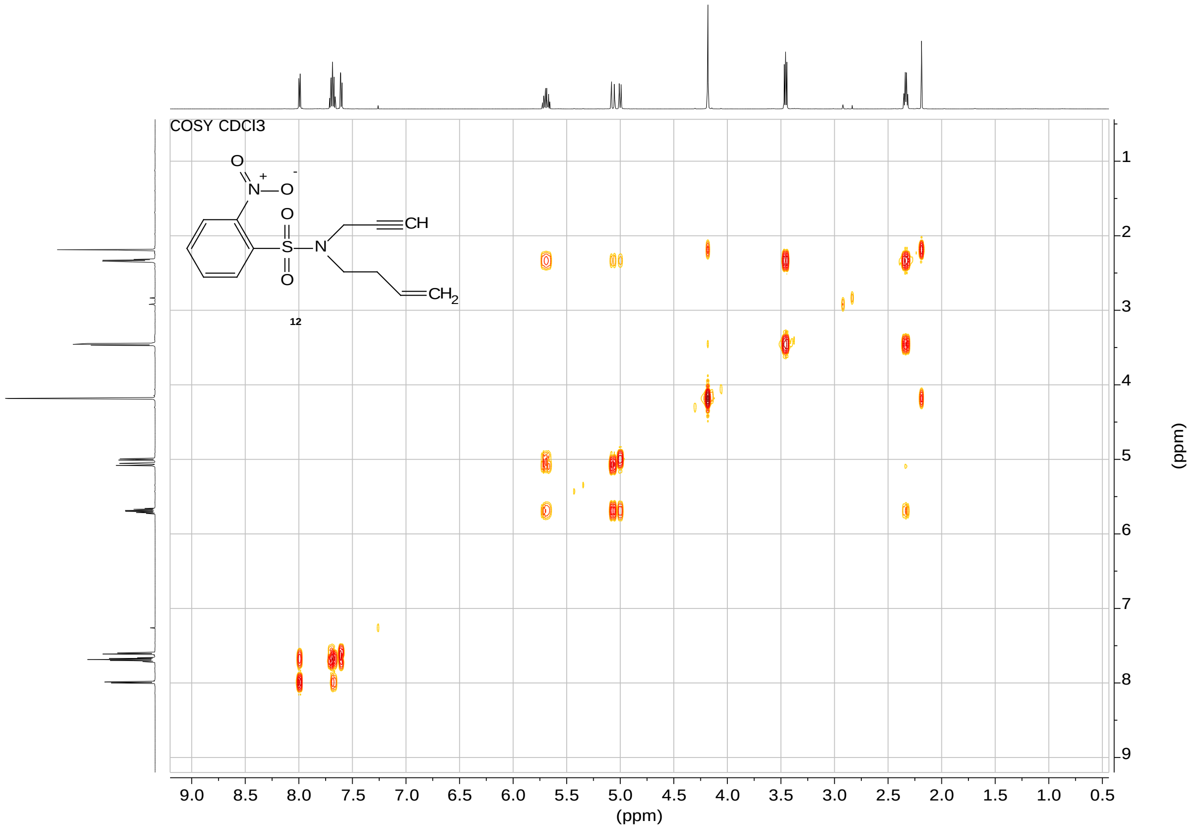
COSY CDCl3
12
(ppm)
(ppm)

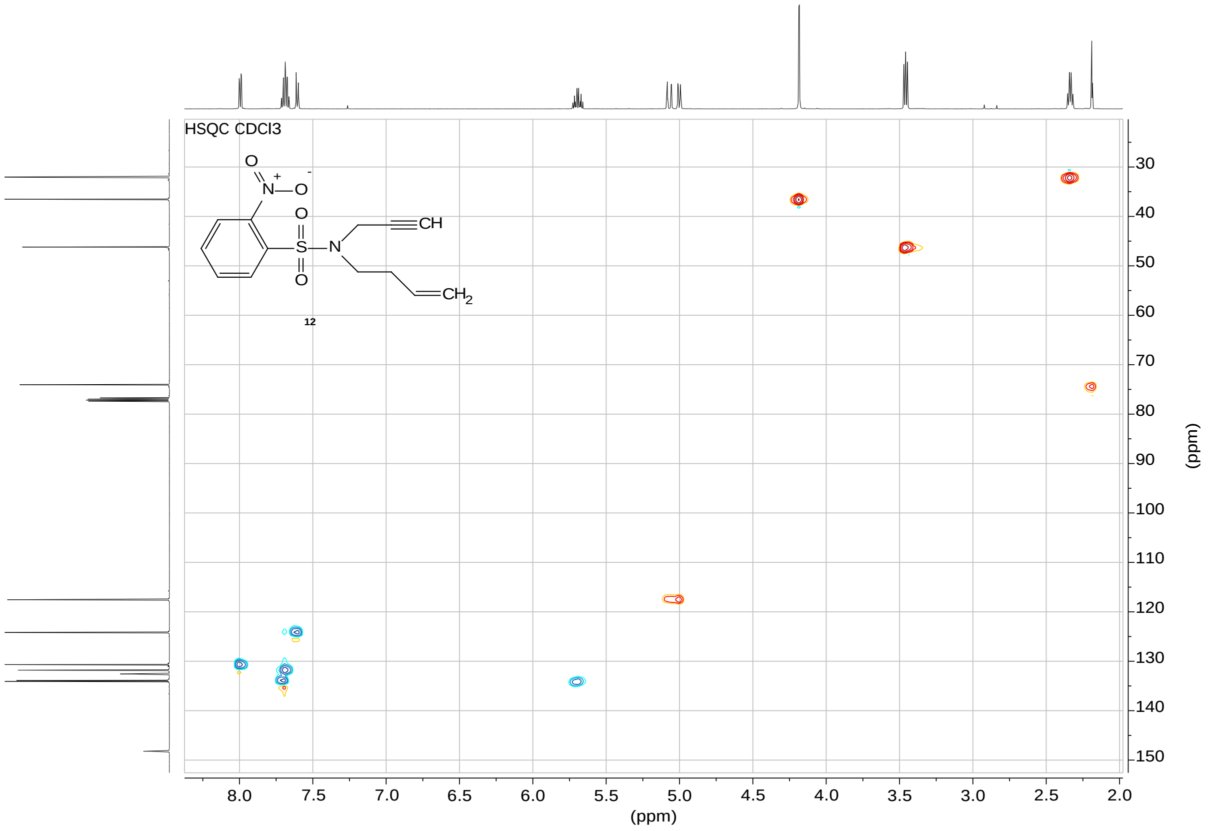
HSQC CDCl3
12
(ppm)
(ppm)

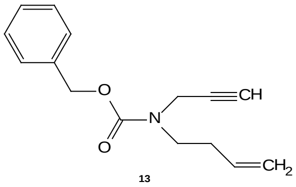

O
N
CH
O
CH_2

13

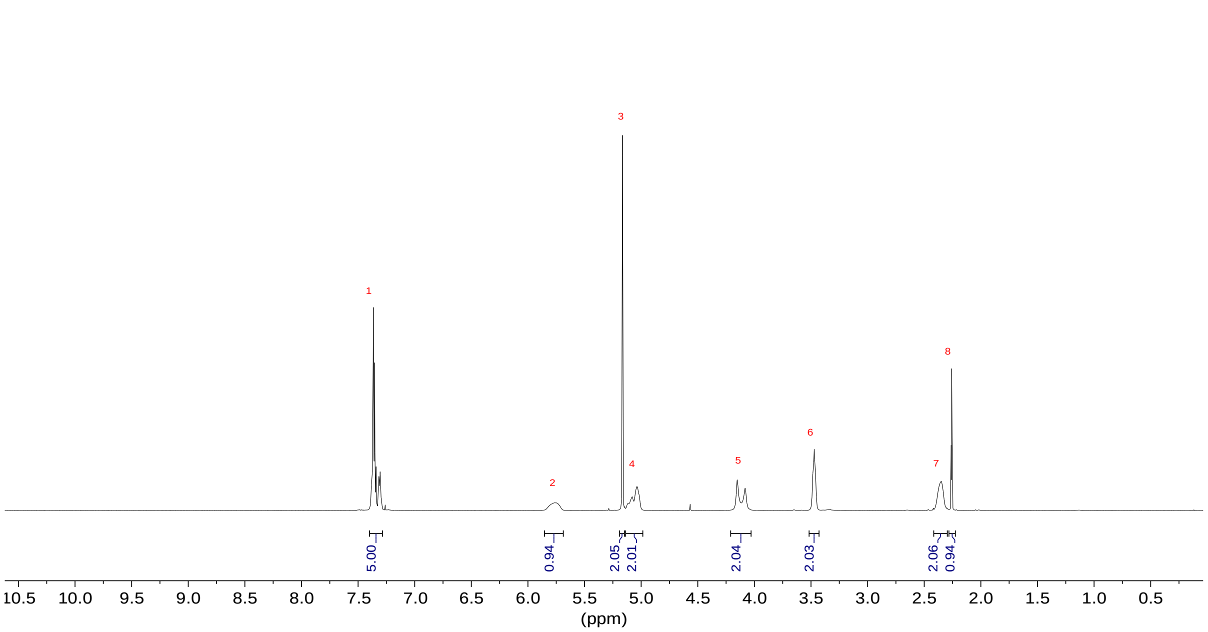

7.37
7.35
7.34
7.32
7.32
7.31
7.31
7.29
5.79
5.76
5.75
5.73
5.16
5.12
5.08
5.04
5.03
4.15
4.08
3.48
3.47
3.46
2.35
2.26
2.26
2.25
5.00
0.94
2.05
2.01
2.04
2.03
2.06
0.94
10.5 10.0 9.5 9.0 8.5 8.0 7.5 7.0 6.5 6.0 5.5 5.0 4.5 4.0 3.5 3.0 2.5 2.0 1.5 1.0 0.5
(ppm)

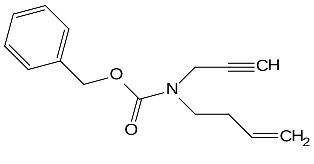

O
N
CH
O
CH_2

13

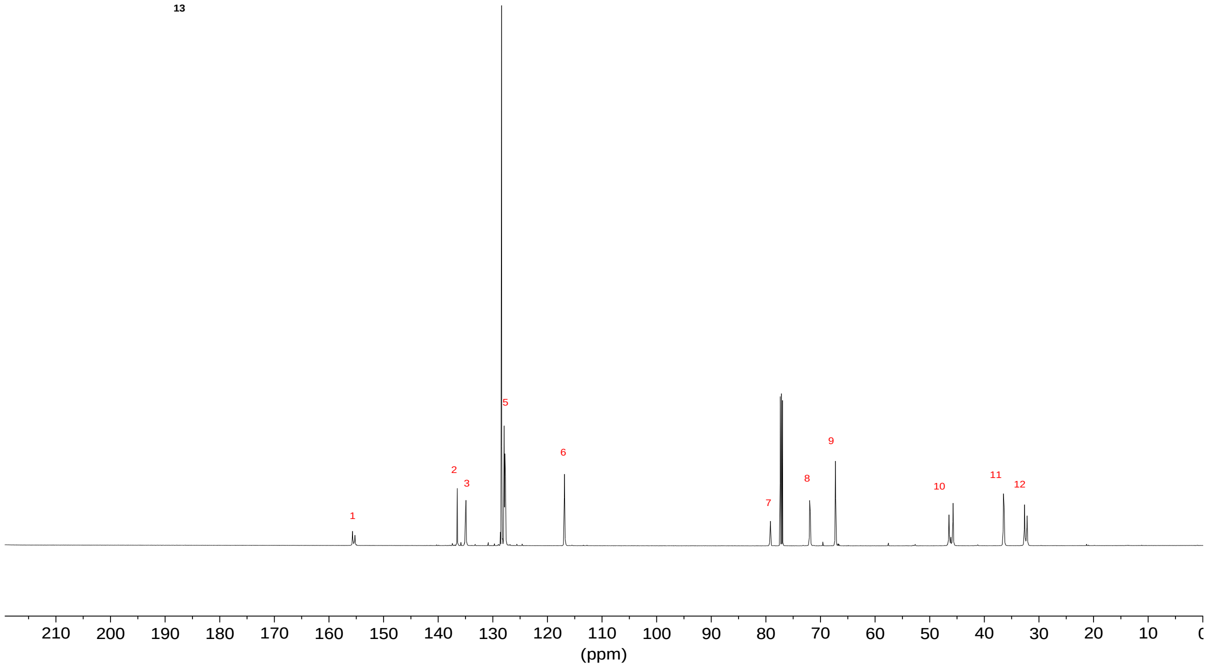

155.69
155.25
136.51
135.00
134.91
128.38
127.92
127.79
127.68
116.88
79.17
71.99
71.87
67.27
46.47
45.72
36.50
36.37
32.64
32.16
210 200 190 180 170 160 150 140 130 120 110 100 90 80 70 60 50 40 30 20 10
(ppm)

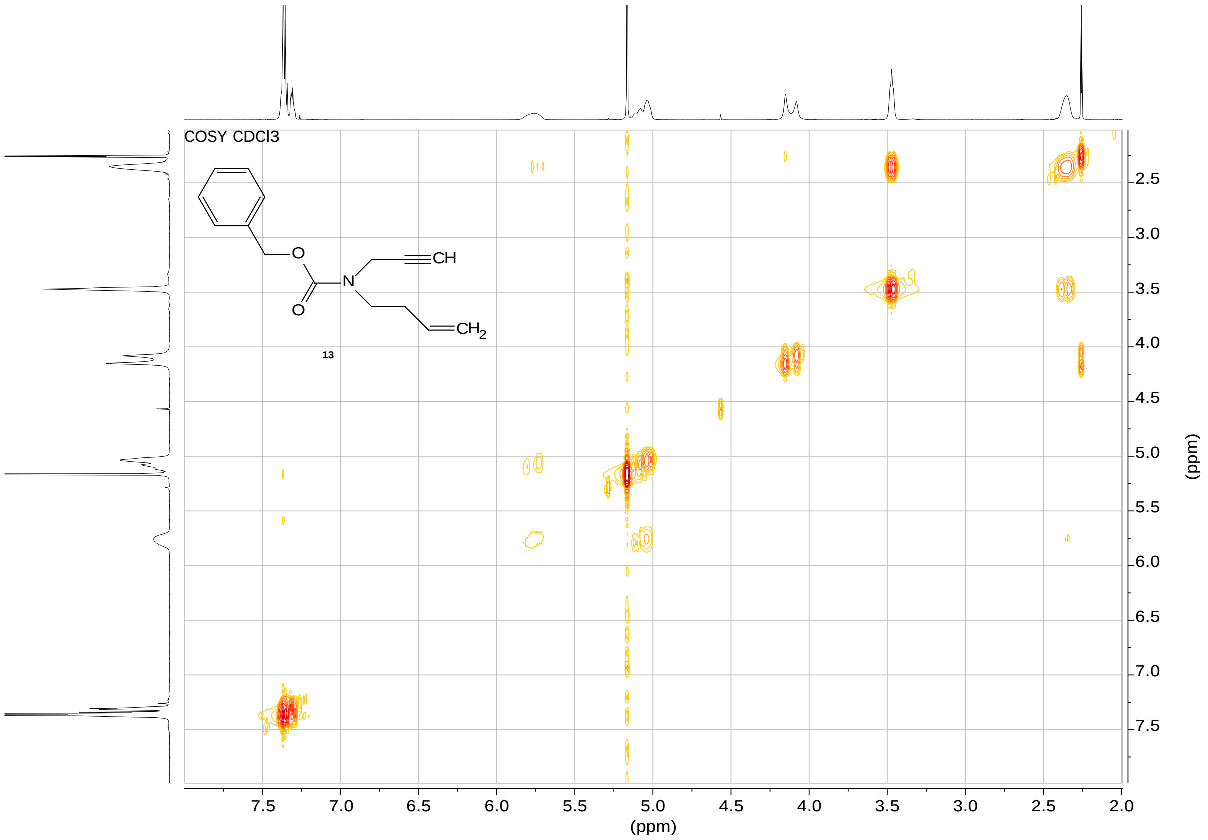
COSY CDCl3
CH
N
O
O
CH2
13
(ppm)
2.5
3.0
3.5
4.0
4.5
5.0
5.5
6.0
6.5
7.0
7.5
7.5 7.0 6.5 6.0 5.5 5.0 4.5 4.0 3.5 3.0 2.5 2.0
(ppm)

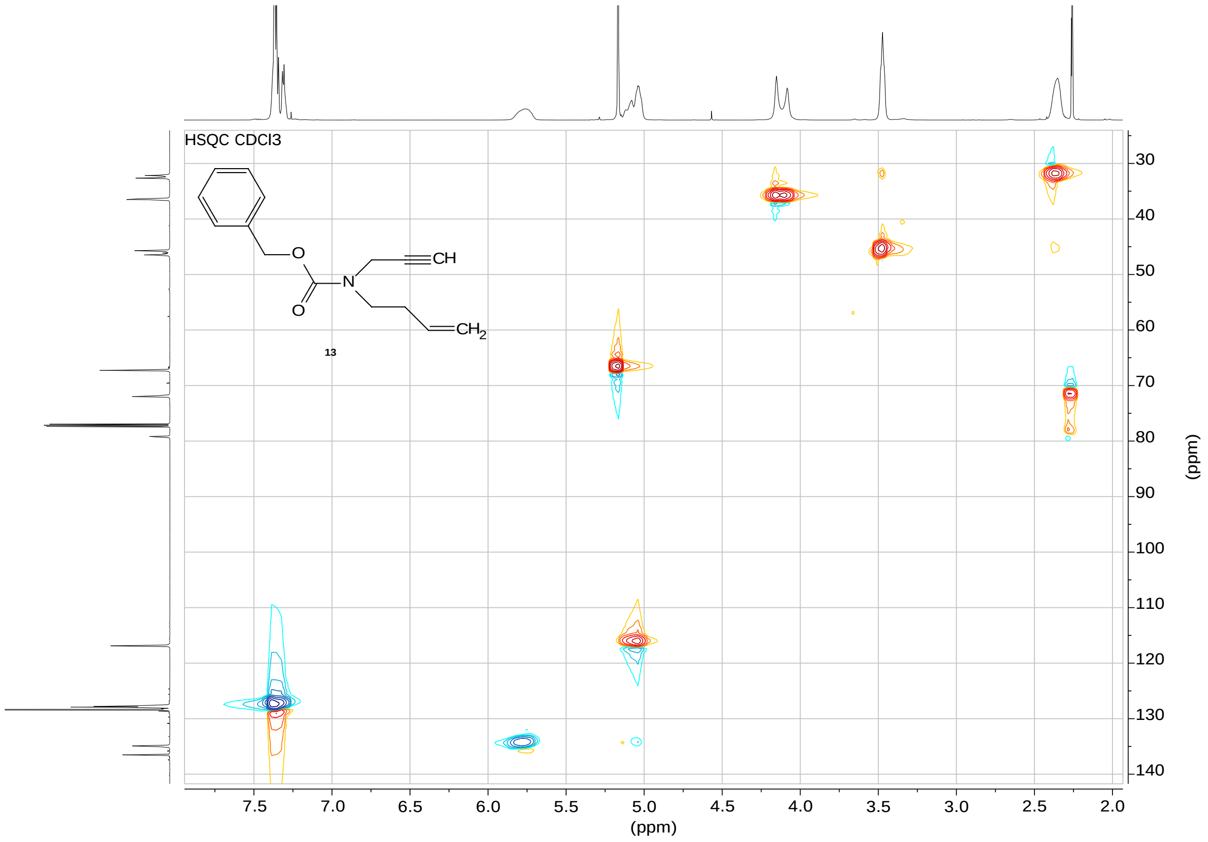
HSQC CDCl3
CH
N
O
O
CH2
13
(ppm)
30
40
50
60
70
80
90
100
110
120
130
140
7.5 7.0 6.5 6.0 5.5 5.0 4.5 4.0 3.5 3.0 2.5 2.0
(ppm)

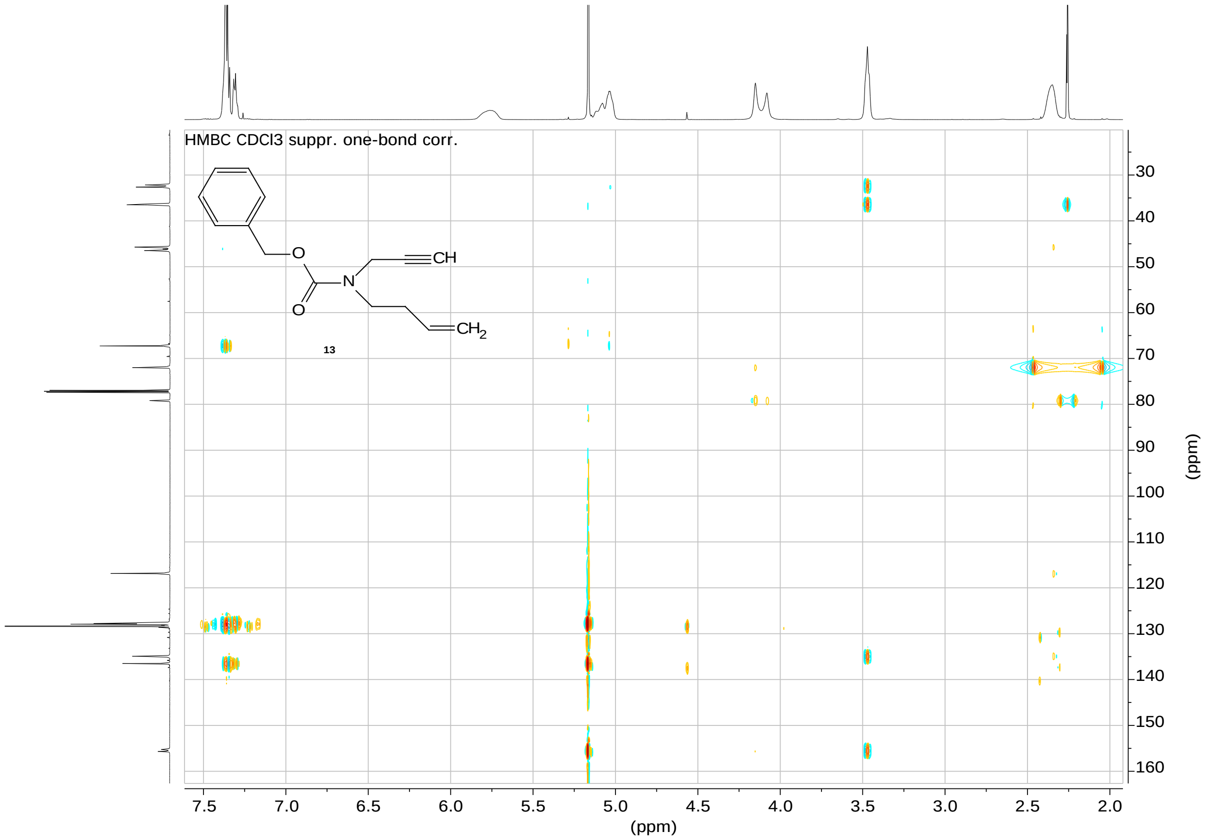
HMBC CDCl3 suppr. one-bond corr.
O
N
O
CH
CH2
13
30
40
50
60
70
80
90
100
110
120
130
140
150
160
(ppm)
7.5
7.0
6.5
6.0
5.5
5.0
4.5
4.0
3.5
3.0
2.5
2.0
(ppm)

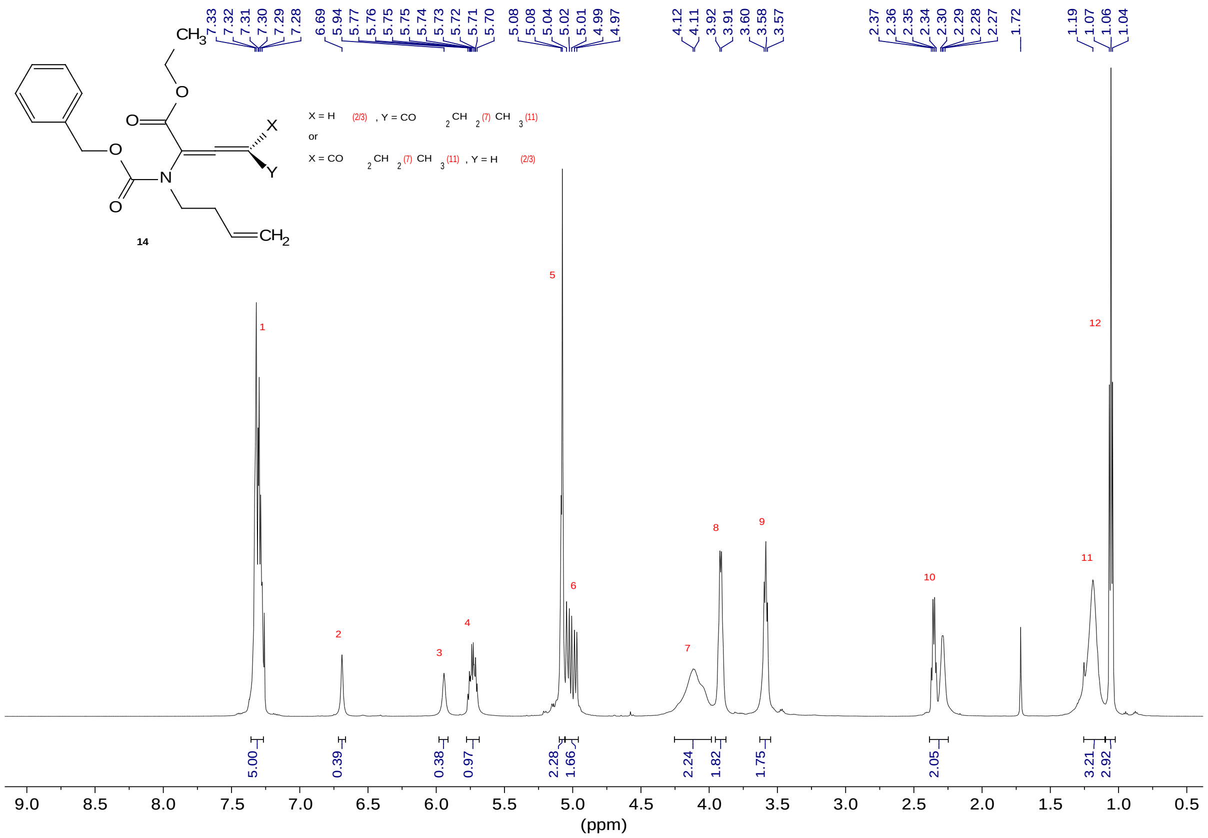

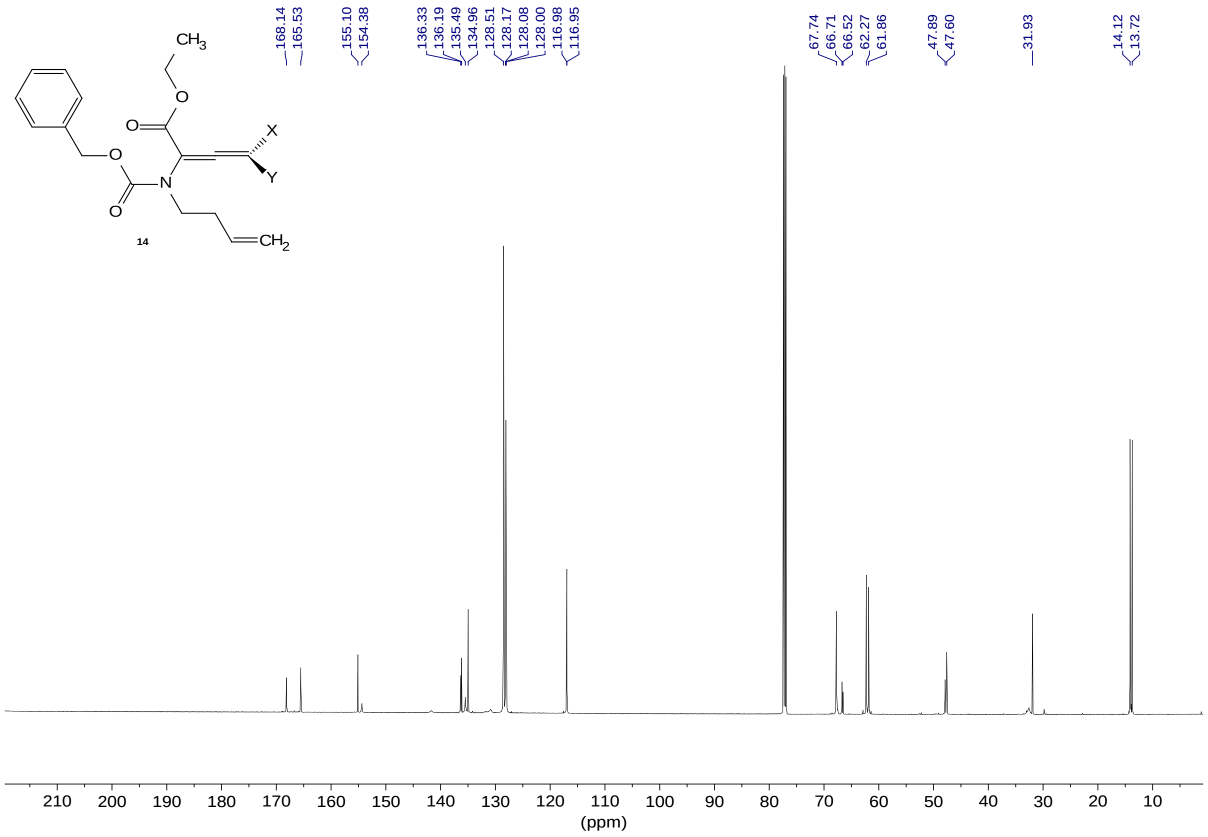

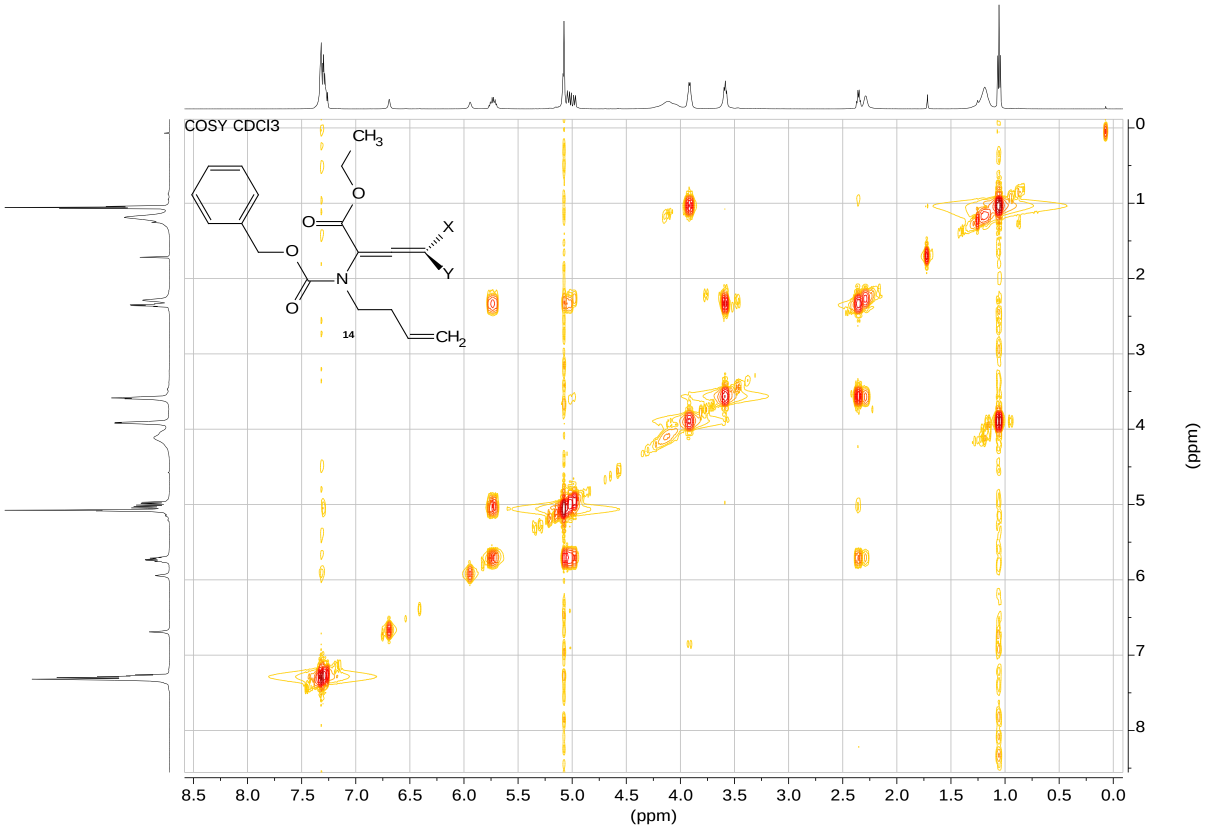
COSY CDCl3
CH3
O
O
O
N
X
Y
O
14
CH2
(ppm)
(ppm)

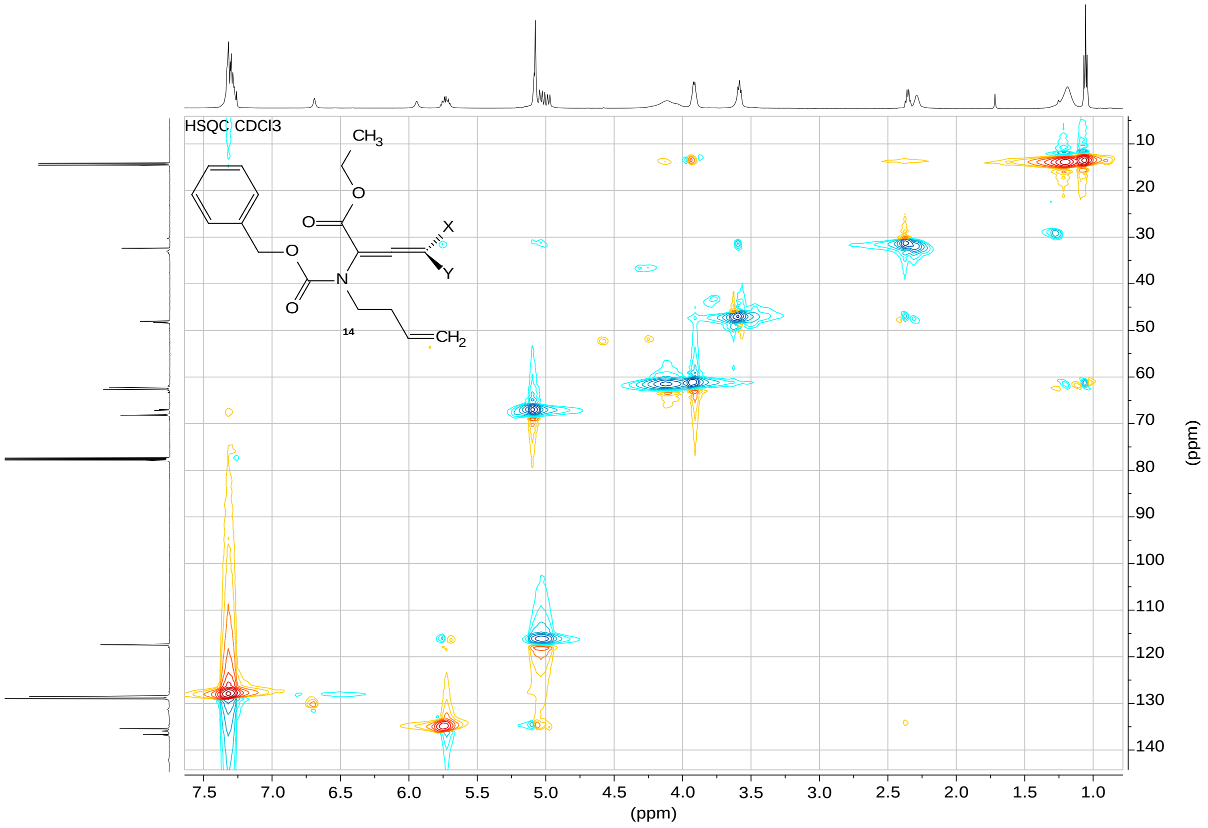
HSQC CDCl3
CH3
O
O
O
N
X
Y
O
14
CH2
(ppm)
(ppm)

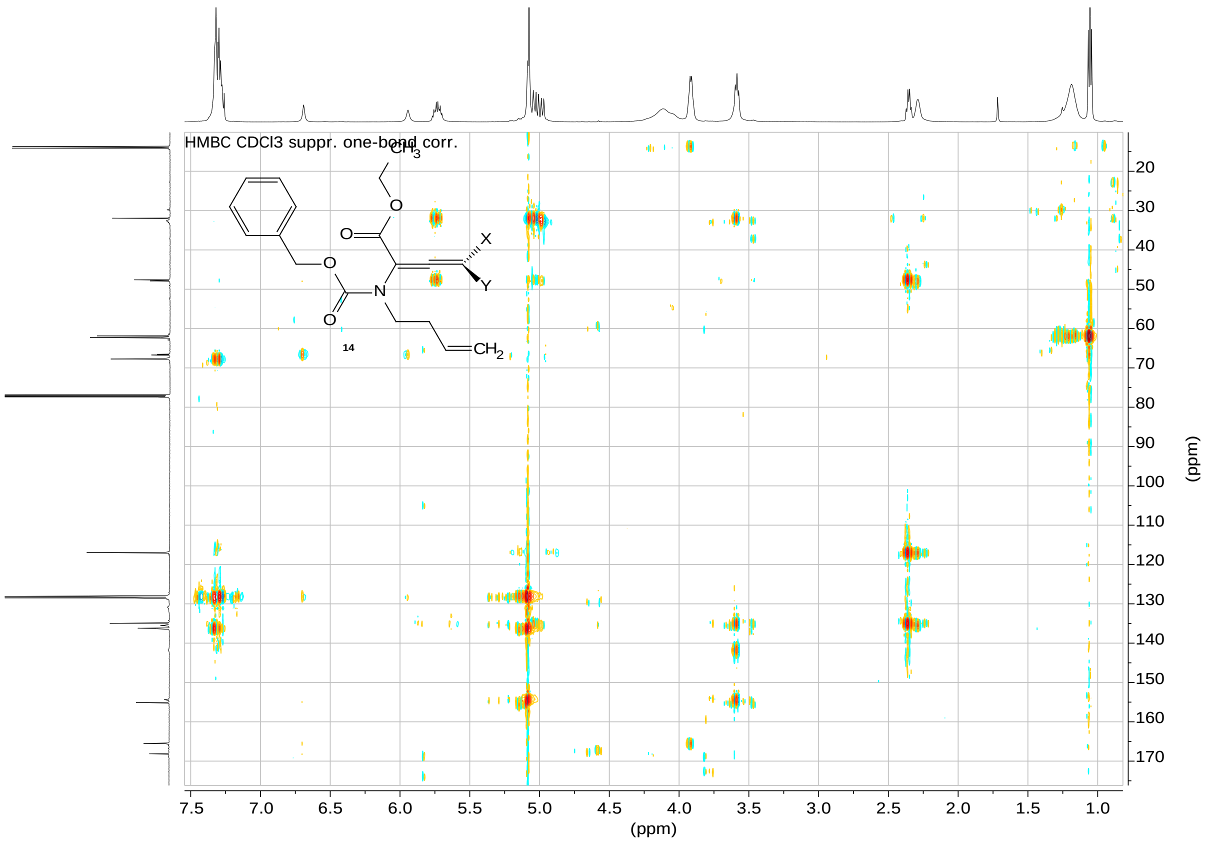

HMBC CDCl3 suppr. one-bond corr.
CH3
O
O
O
N
O
X
Y
CH2
14
(ppm)
(ppm)

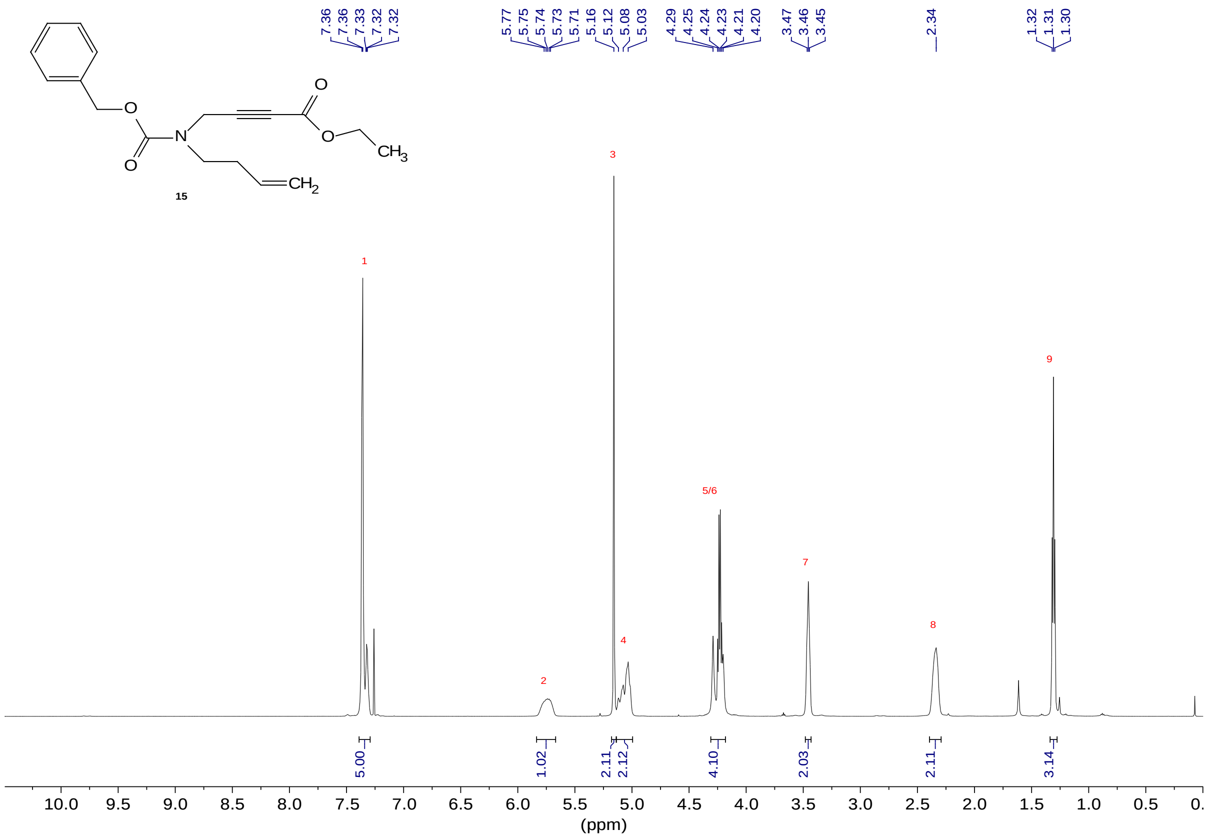
7.36 7.36 7.33 7.32 7.32
5.77 5.75 5.74 5.73 5.71 5.16 5.12 5.08 5.03
4.29 4.25 4.24 4.23 4.21 4.20
3.47 3.46 3.45
2.34
1.32 1.31 1.30
O
O
N
O
O
CH3
CH2
15
5.00 1.02 2.11 2.12 4.10 2.03 2.11 3.14
(ppm)

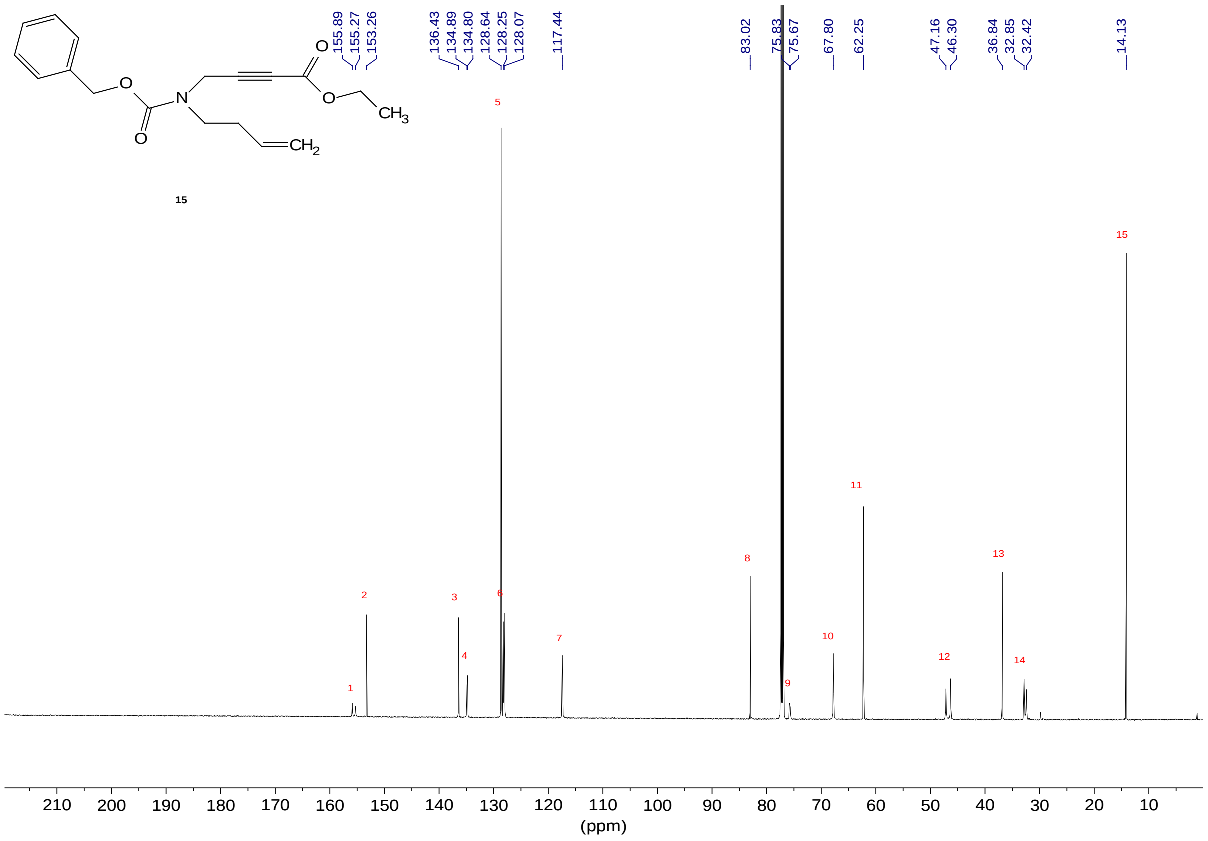
155.89 155.27 153.26
136.43 134.89 134.80 128.64 128.25 128.07
117.44
83.02 75.83 75.67 67.80 62.25
47.16 46.30 36.84 32.85 32.42
14.13
O
O
N
O
O
CH3
CH2
15
(ppm)

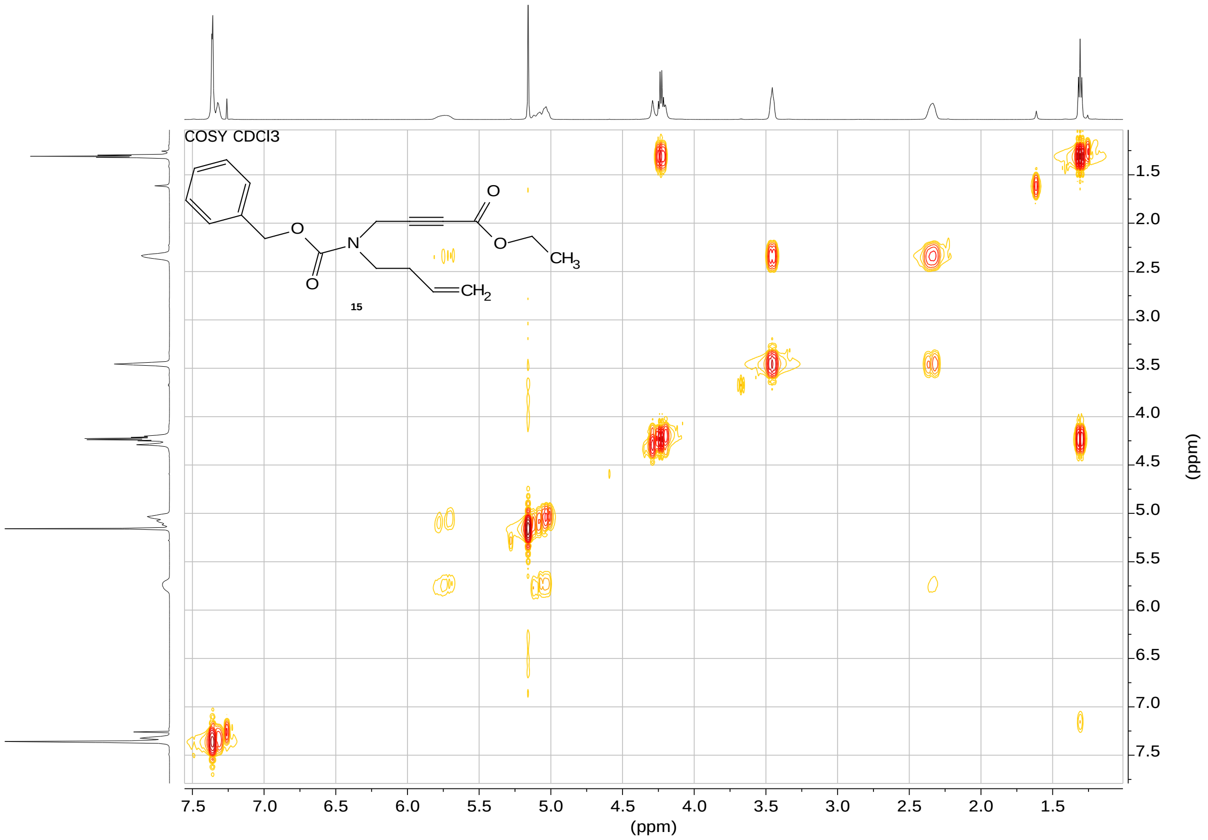
COSY CDCl3
O
O
N
O
O
CH3
CH2
15
7.5
7.0
6.5
6.0
5.5
5.0
4.5
4.0
3.5
3.0
2.5
2.0
1.5
(ppm)

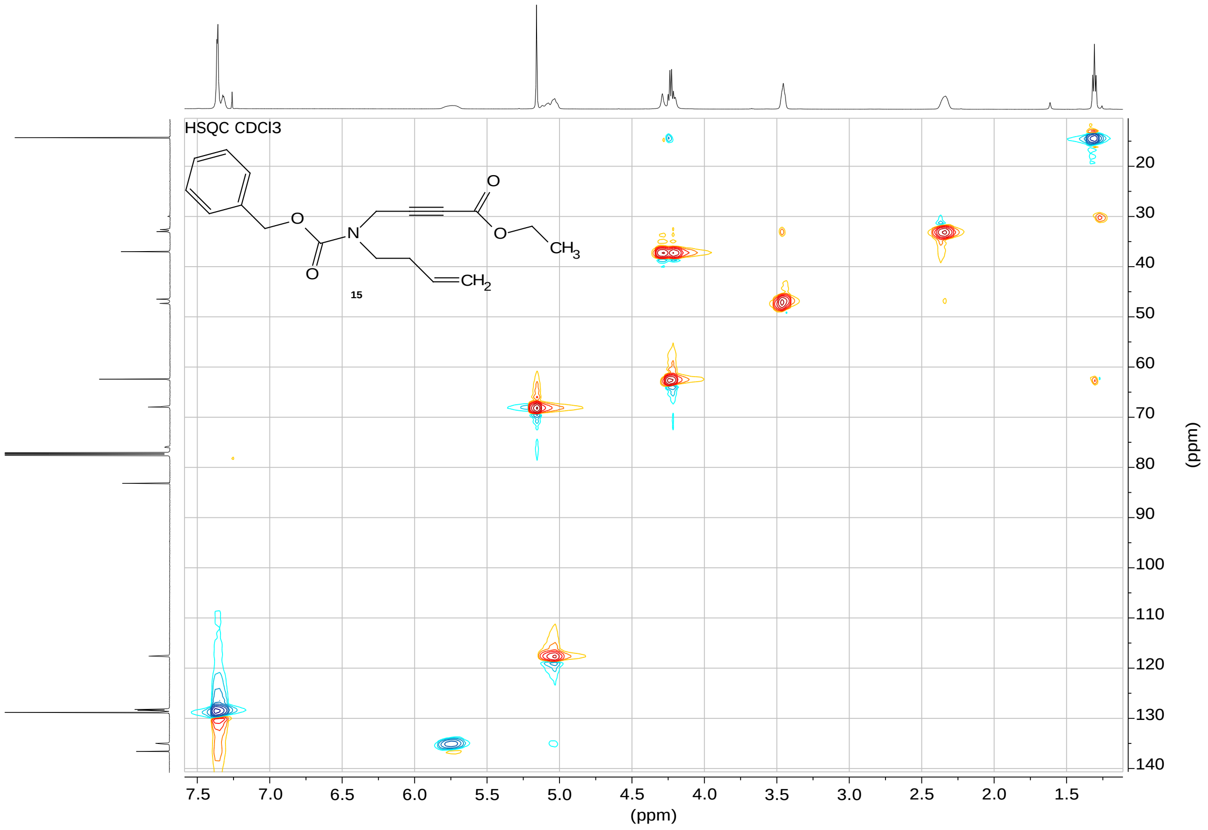
HSQC CDCl3
O
O
N
O
O
CH3
CH2
15
20
30
40
50
60
70
80
90
100
110
120
130
140
(ppm)
7.5
7.0
6.5
6.0
5.5
5.0
4.5
4.0
3.5
3.0
2.5
2.0
1.5
(ppm)

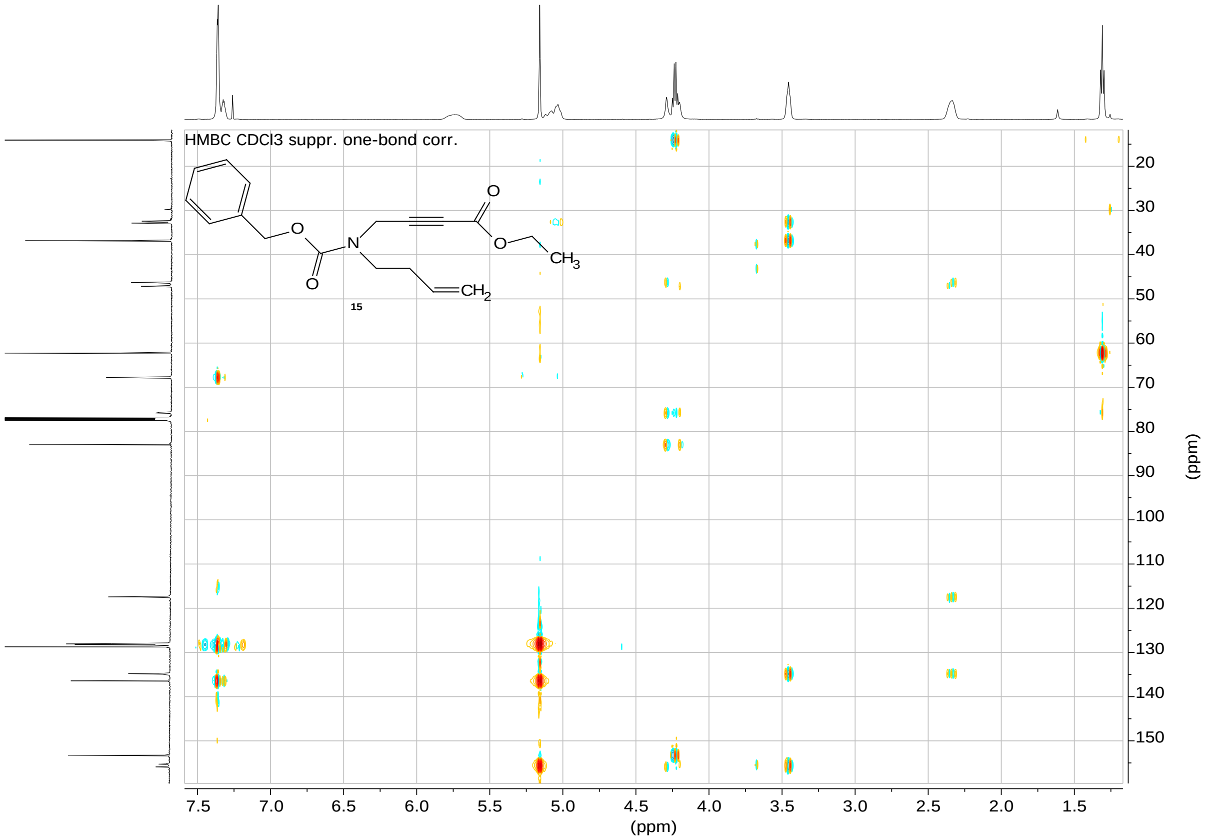

HMBC CDCl3 suppr. one-bond corr.
O
O
N
O
O
CH3
CH2
15
(ppm)
(ppm)

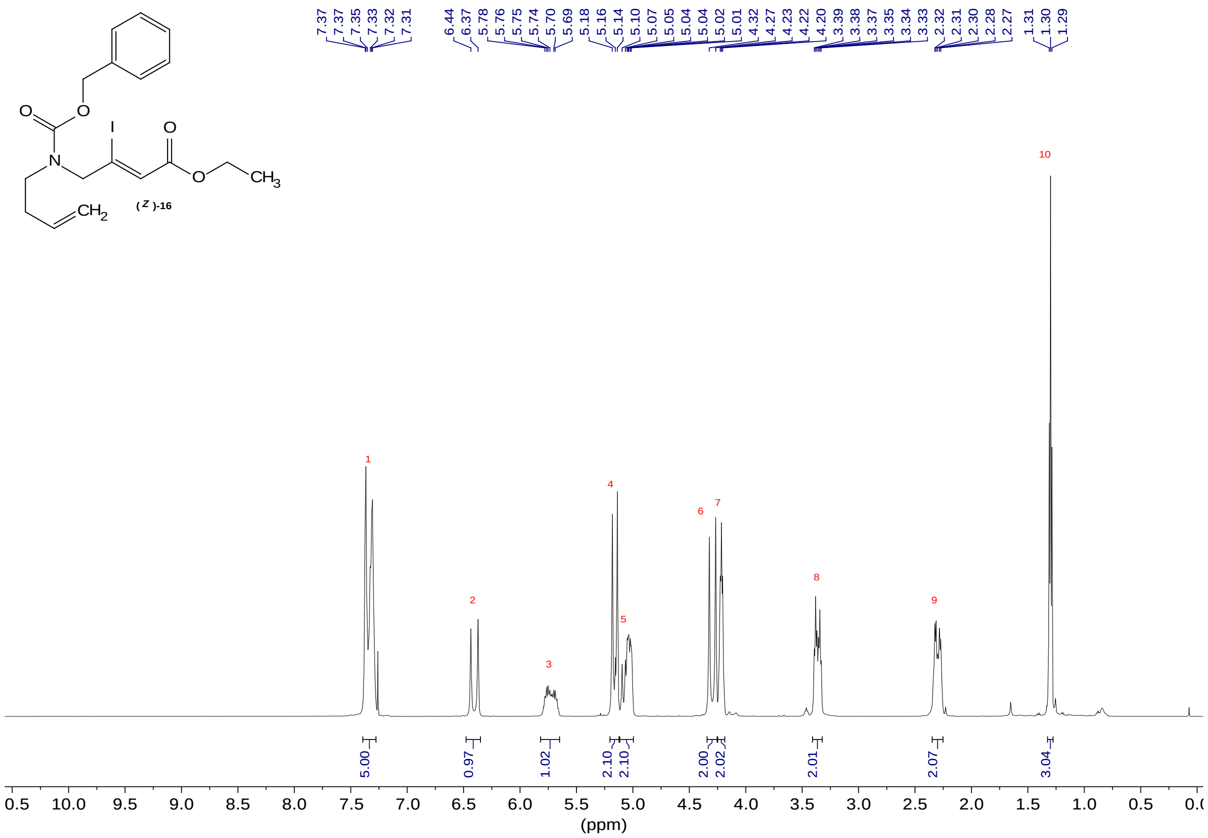
(Z)-16
(ppm)

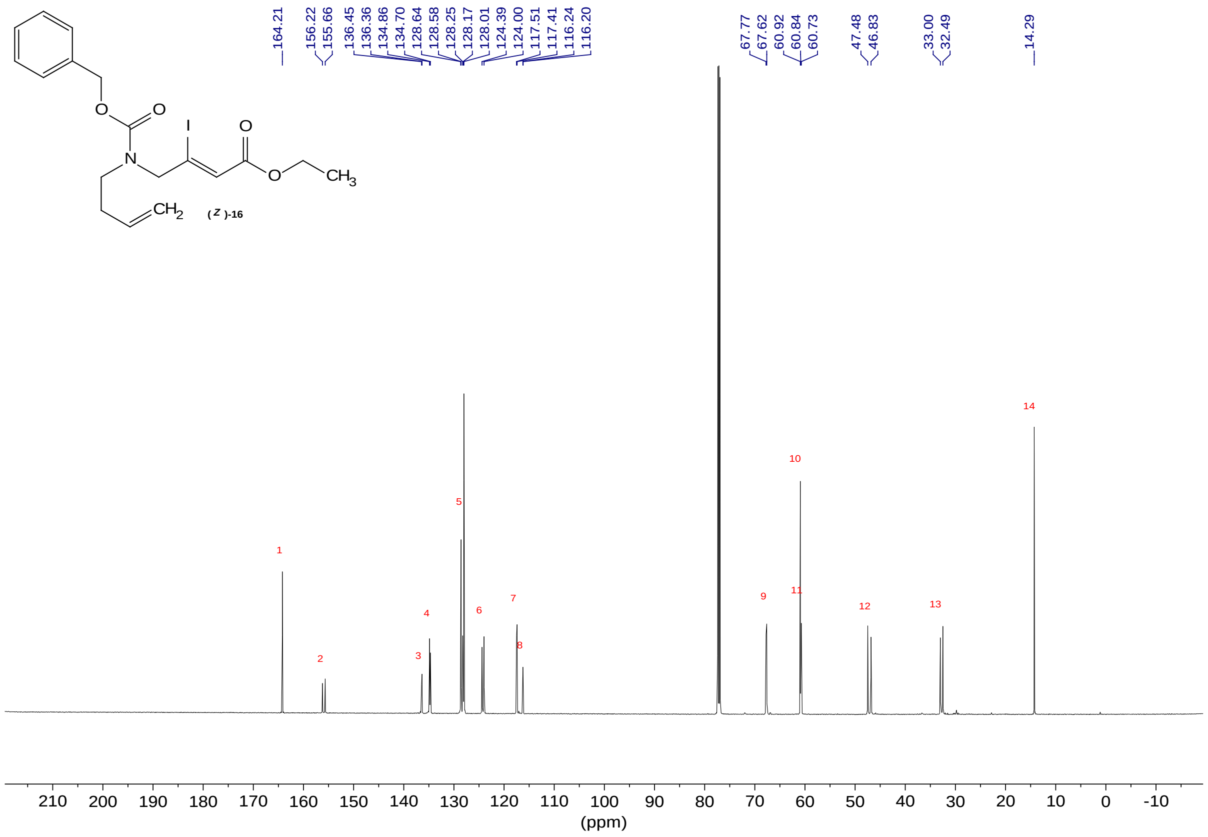
(Z)-16
(ppm)

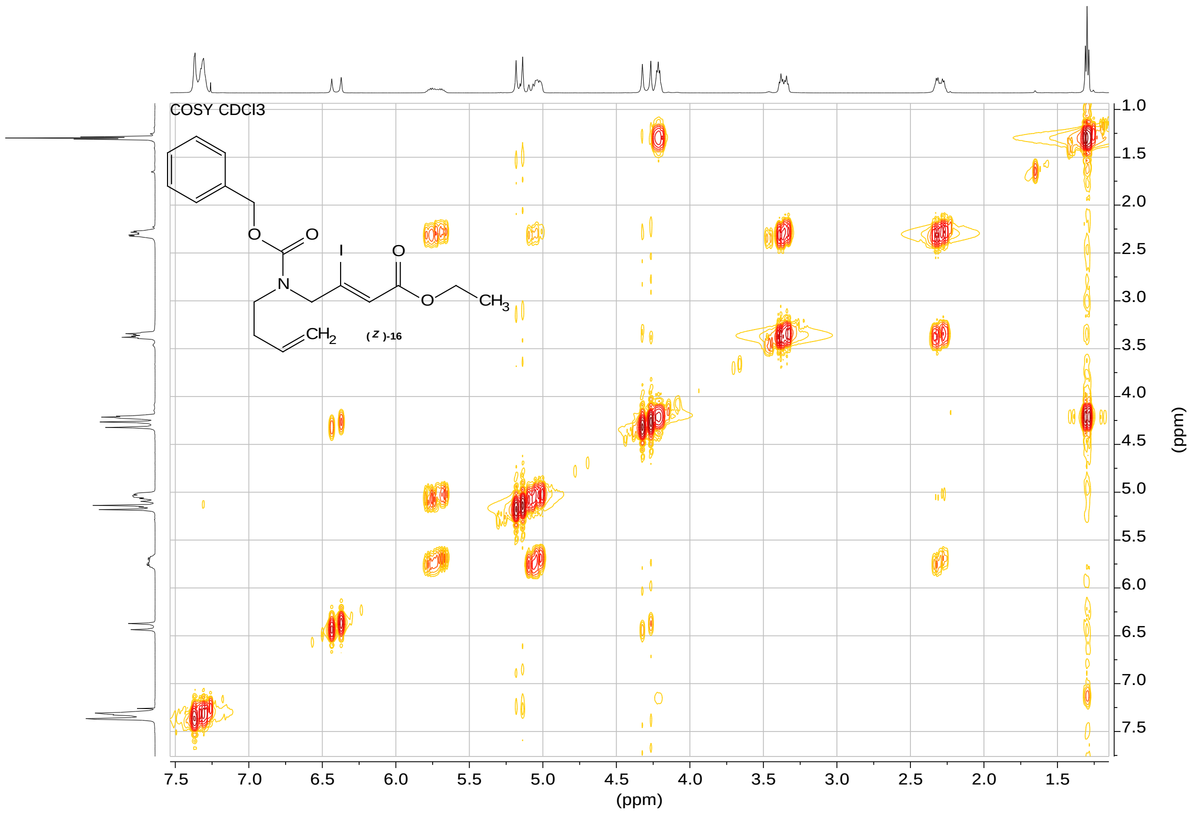
COSY CDCl3
O
O
I
O
N
O
CH3
CH2
(Z)-16
(ppm)
(ppm)

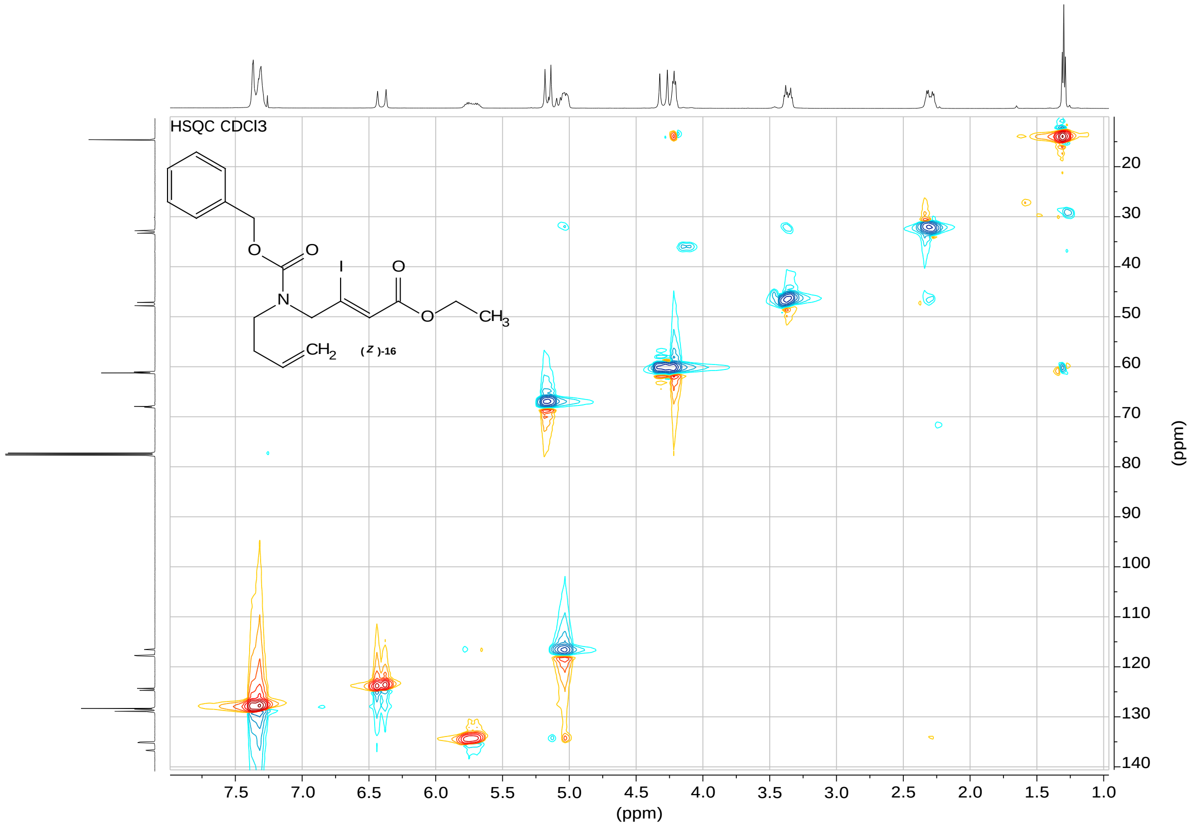
HSQC CDCl3
O
O
I
O
N
O
CH3
CH2
(Z)-16
(ppm)
(ppm)

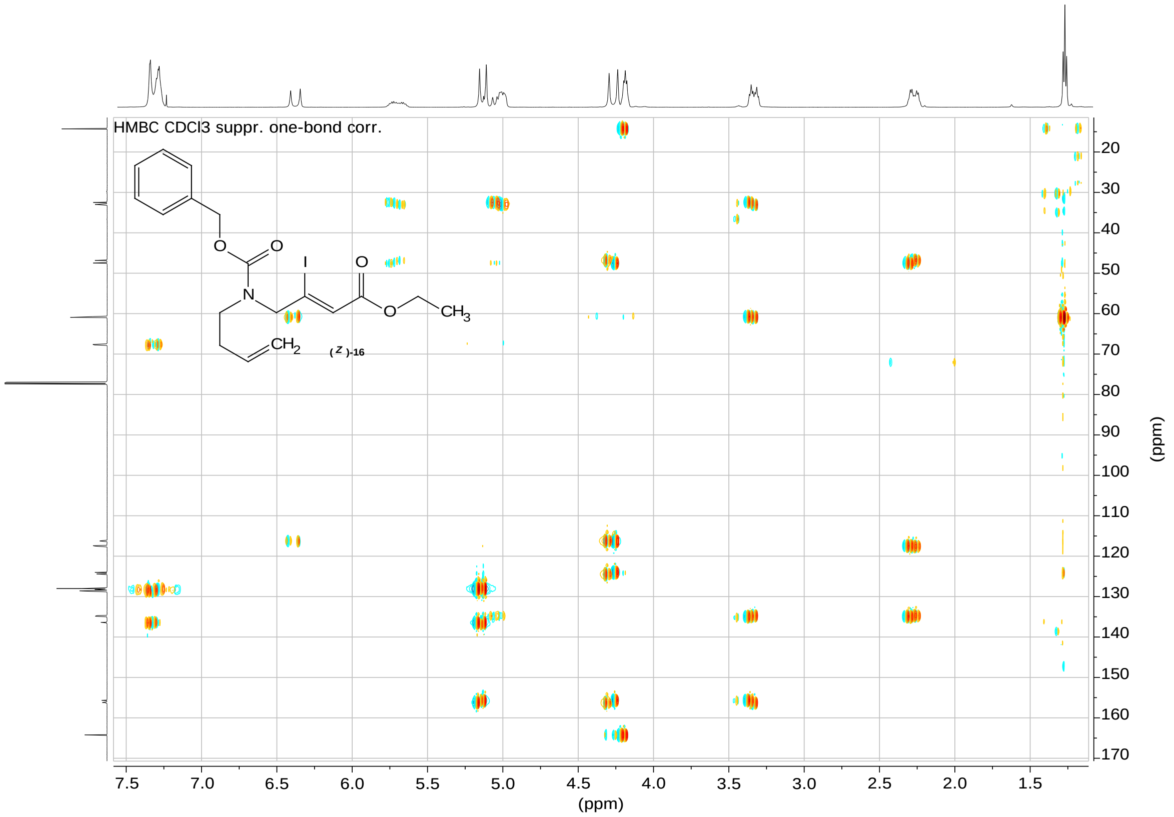

23 DMSO Temp exp.

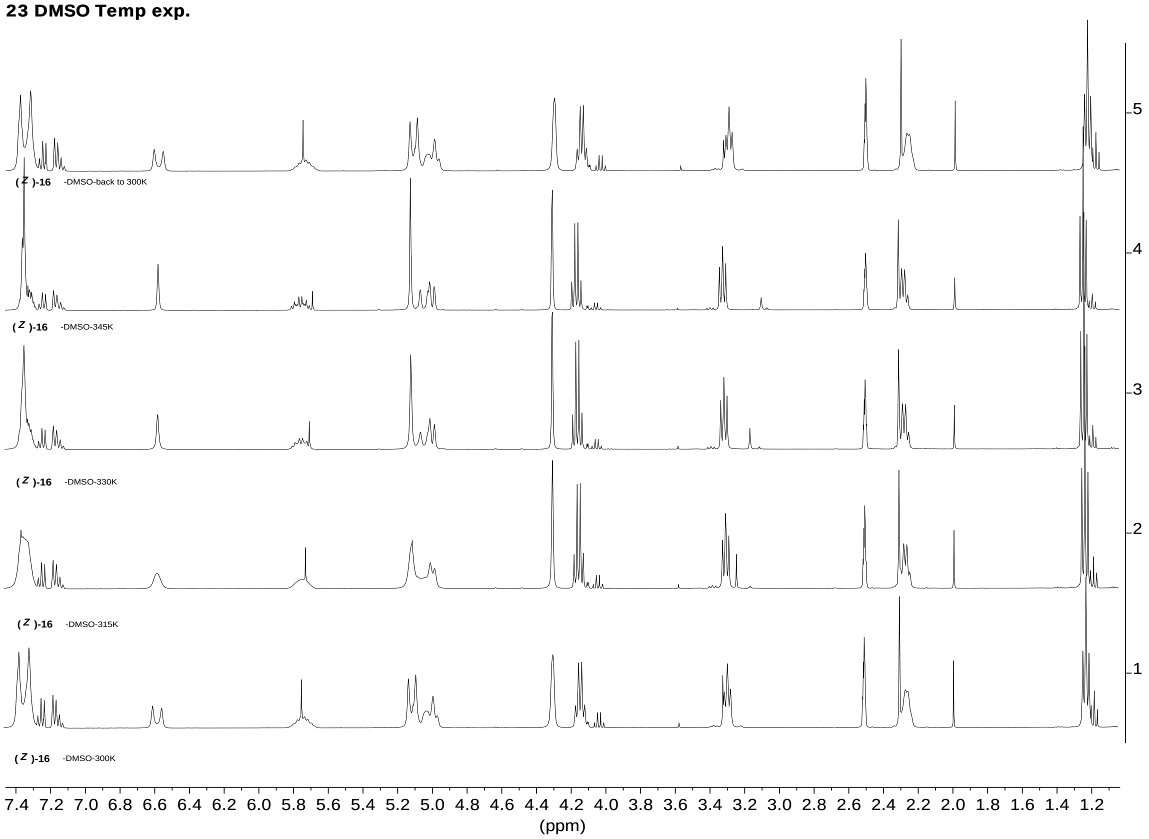

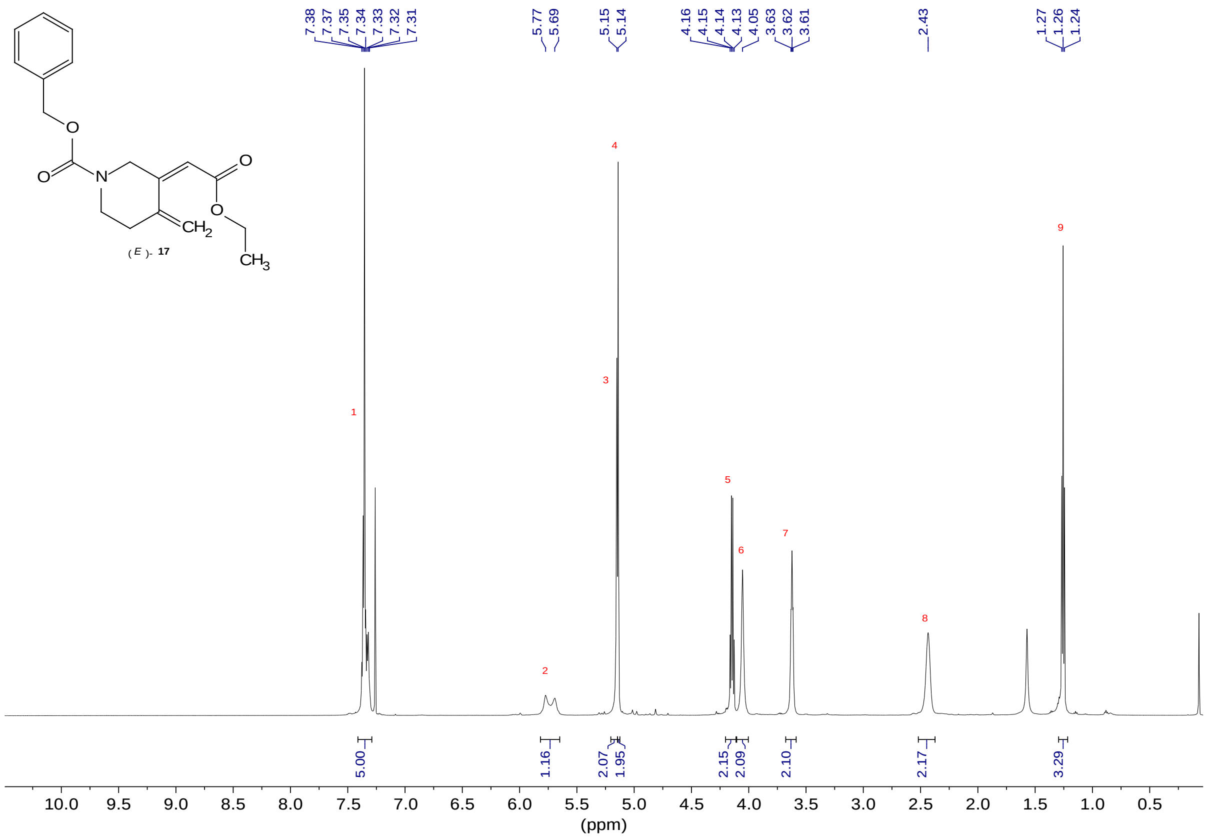
(E)- 17
(ppm)

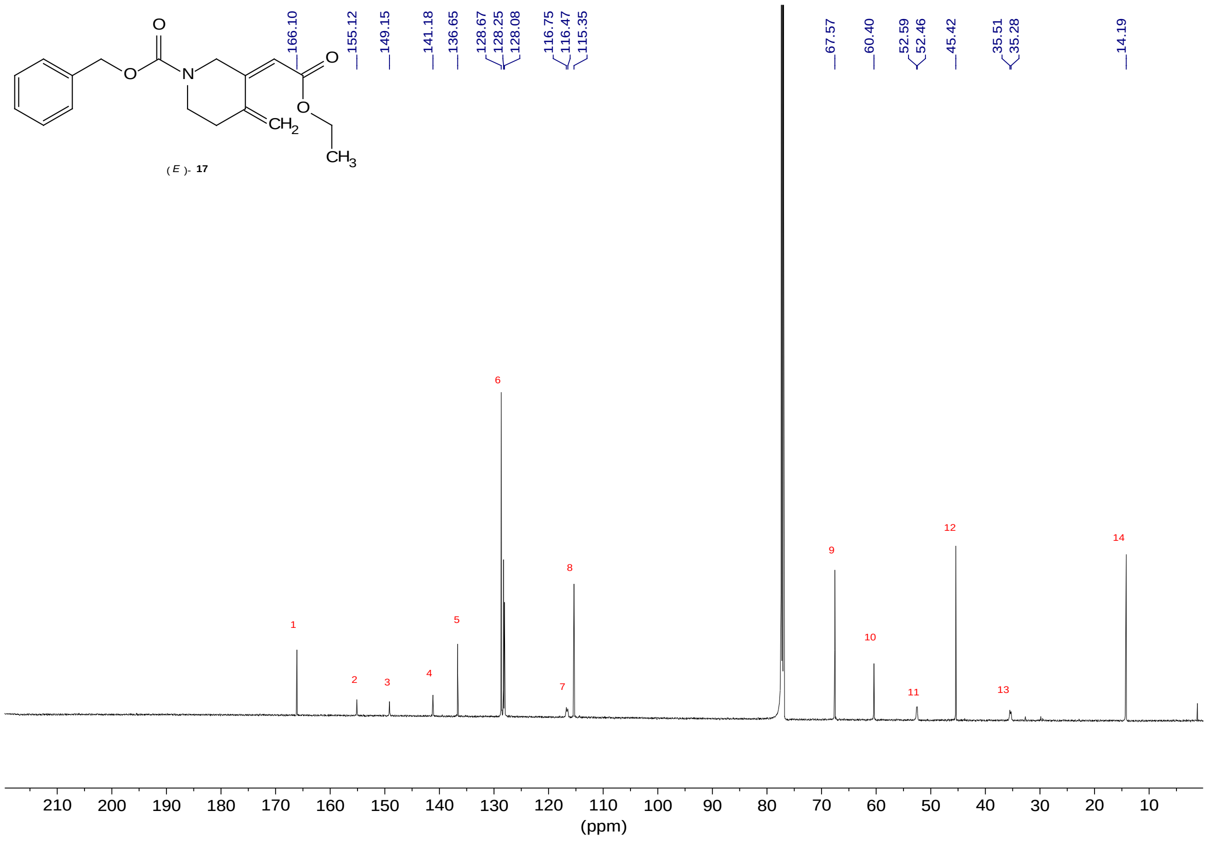
(E)- 17
(ppm)

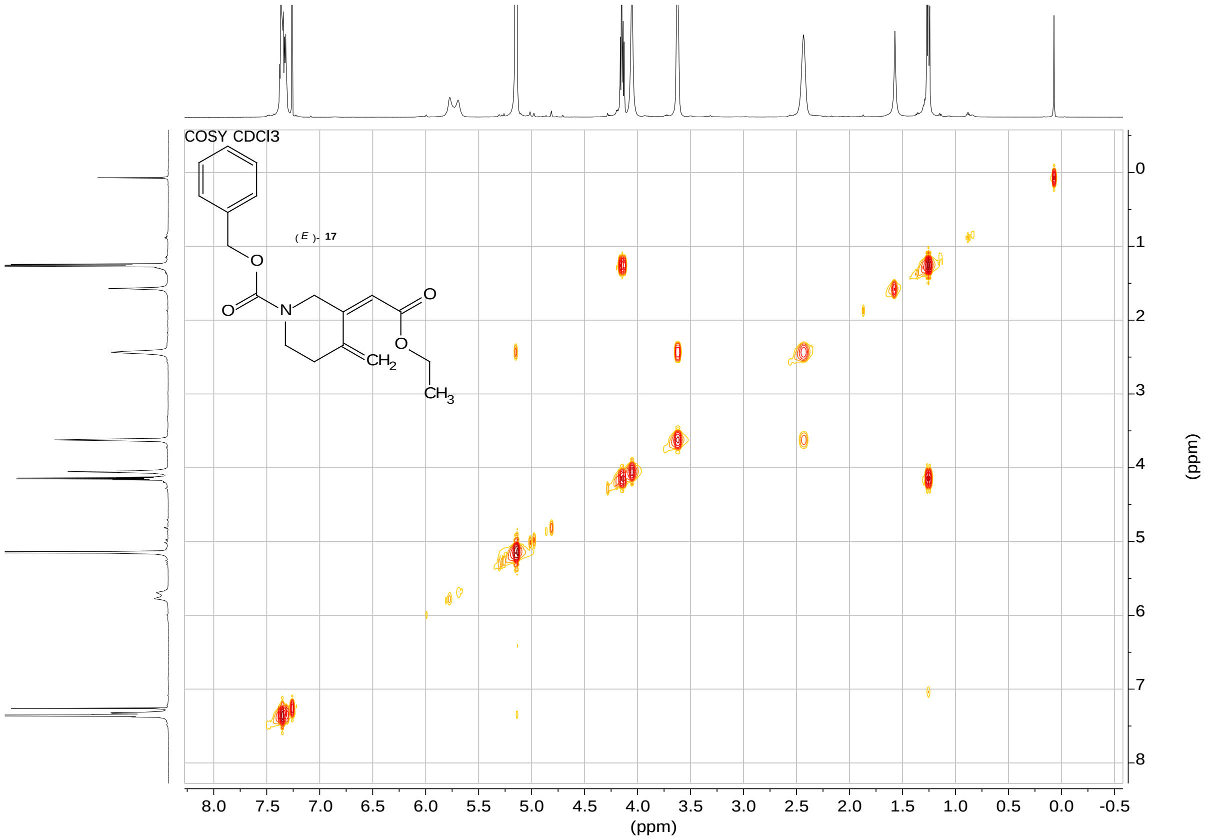
COSY CDCl3
(E)- 17
O
O
N
O
O
CH2
CH3
8.0 7.5 7.0 6.5 6.0 5.5 5.0 4.5 4.0 3.5 3.0 2.5 2.0 1.5 1.0 0.5 0.0 -0.5
(ppm)
0 1 2 3 4 5 6 7 8
(ppm)

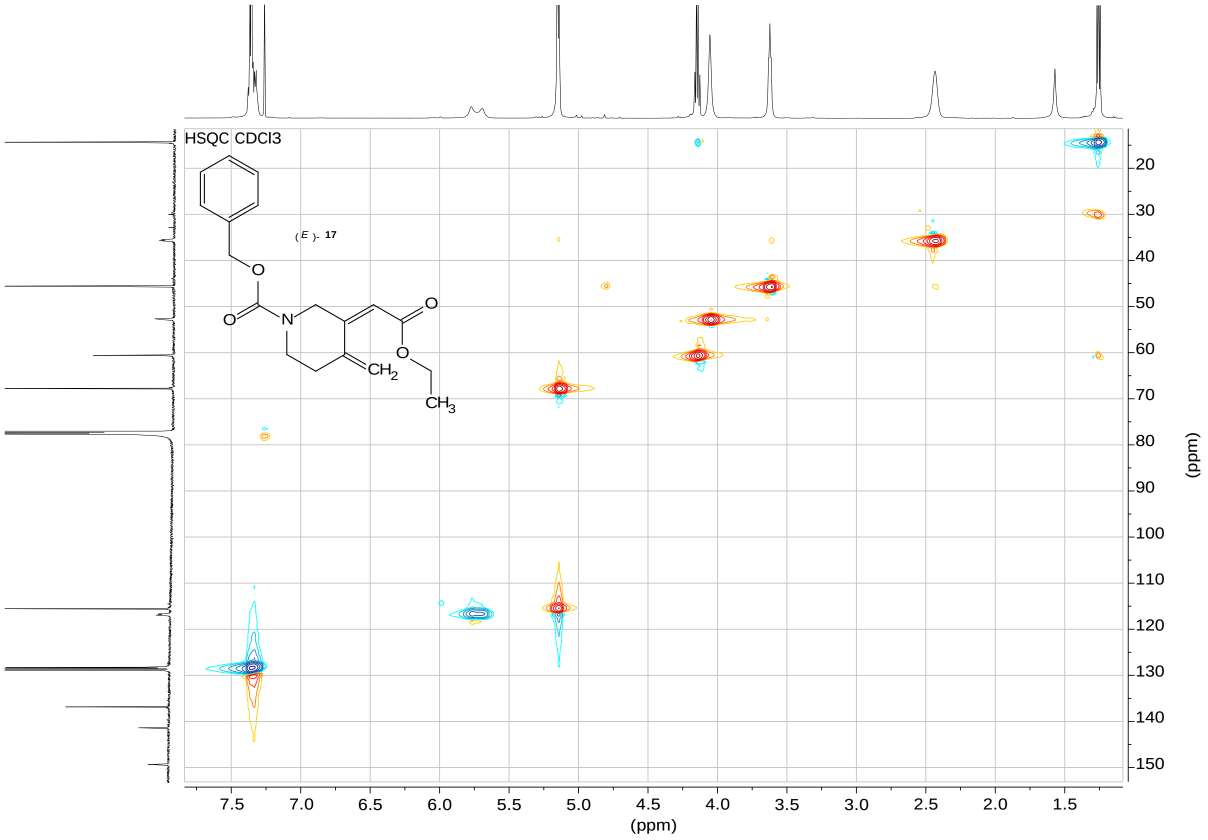
HSQC CDCl3
(E)- 17
O
O
N
O
O
CH2
CH3
7.5 7.0 6.5 6.0 5.5 5.0 4.5 4.0 3.5 3.0 2.5 2.0 1.5
(ppm)
20 30 40 50 60 70 80 90 100 110 120 130 140 150
(ppm)

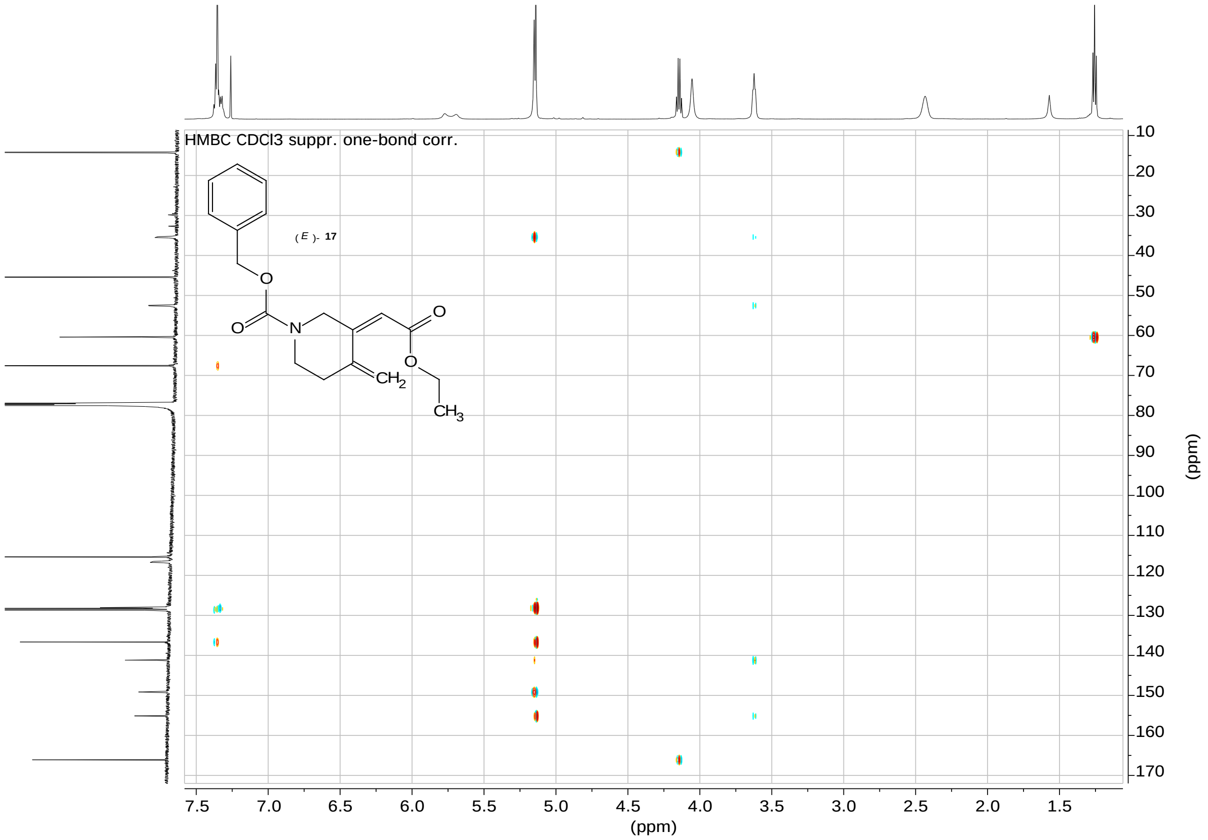
HMBC CDCl3 suppr. one-bond corr.
(E)- 17
O
O
N
O
O
CH2
CH3
(ppm)
(ppm)

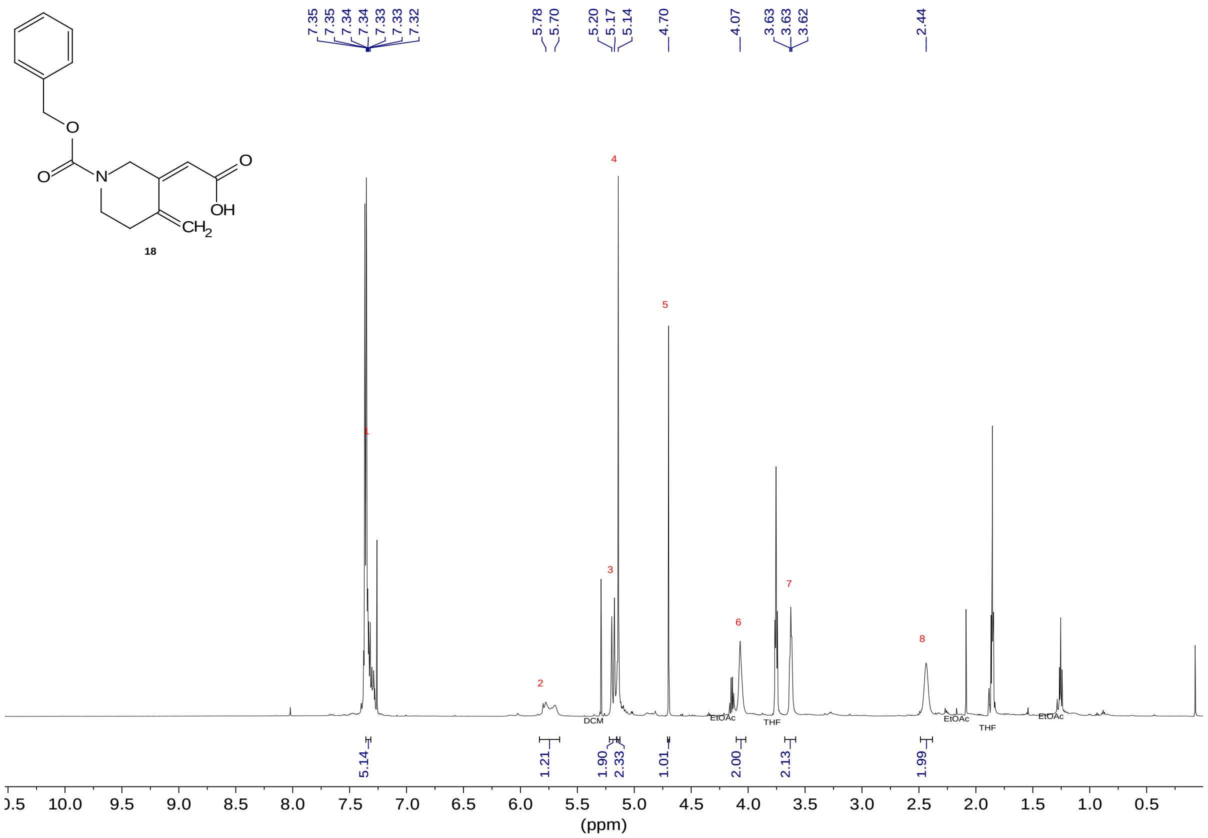
7.35 7.35 7.34 7.34 7.33 7.33 7.32
5.78 5.70
5.20 5.17 5.14
4.70
4.07
3.63 3.63 3.62
2.44
O
O
N
O
OH
CH2
18
DCM
EtOAc
THF
5.14
1.21
1.90
2.33
1.01
2.00
2.13
1.99
10.0 9.5 9.0 8.5 8.0 7.5 7.0 6.5 6.0 5.5 5.0 4.5 4.0 3.5 3.0 2.5 2.0 1.5 1.0 0.5
(ppm)

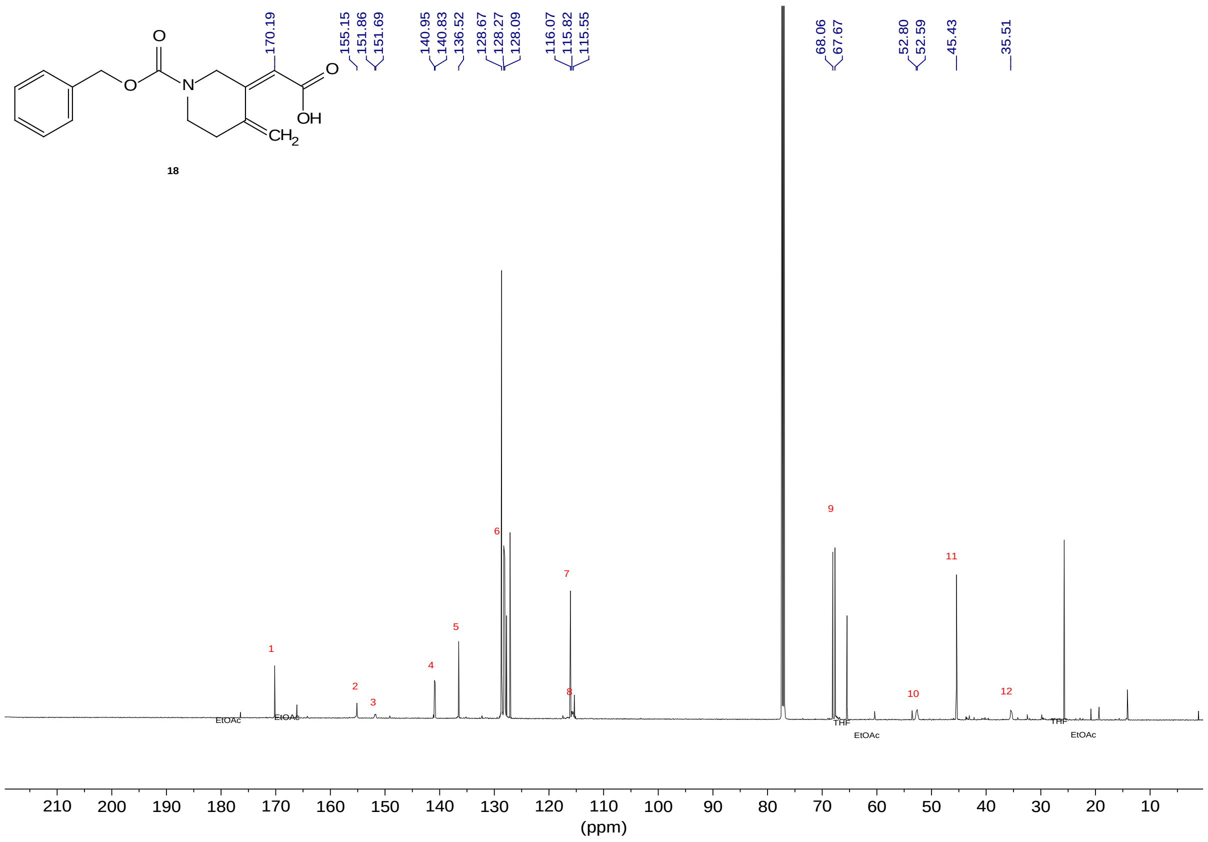
170.19
155.15 151.86 151.69
140.95 140.83 136.52
128.67 128.27 128.09
116.07 115.82 115.55
68.06 67.67
52.80 52.59
45.43
35.51
O
O
N
O
OH
CH2
18
EtOAc
THF
210 200 190 180 170 160 150 140 130 120 110 100 90 80 70 60 50 40 30 20 10
(ppm)

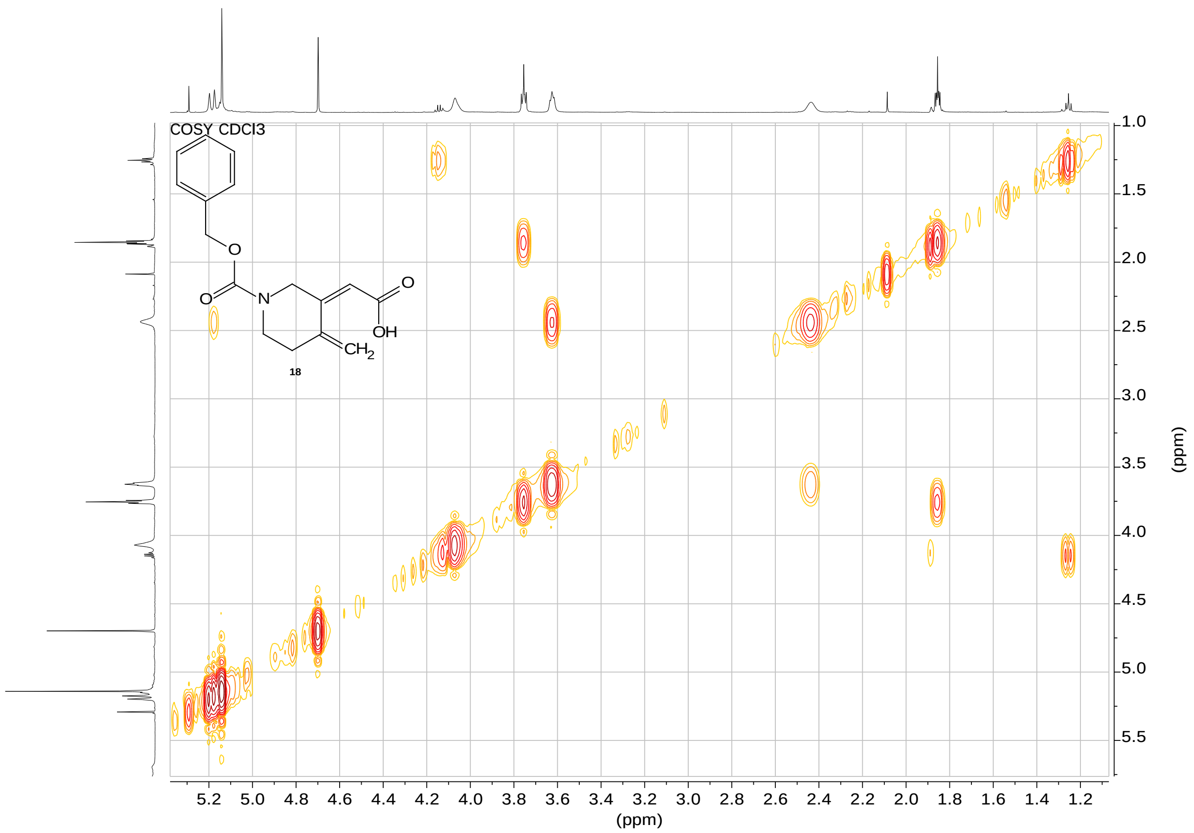
COSY CDCl3
O
O
N
O
OH
CH2
18
(ppm)
(ppm)

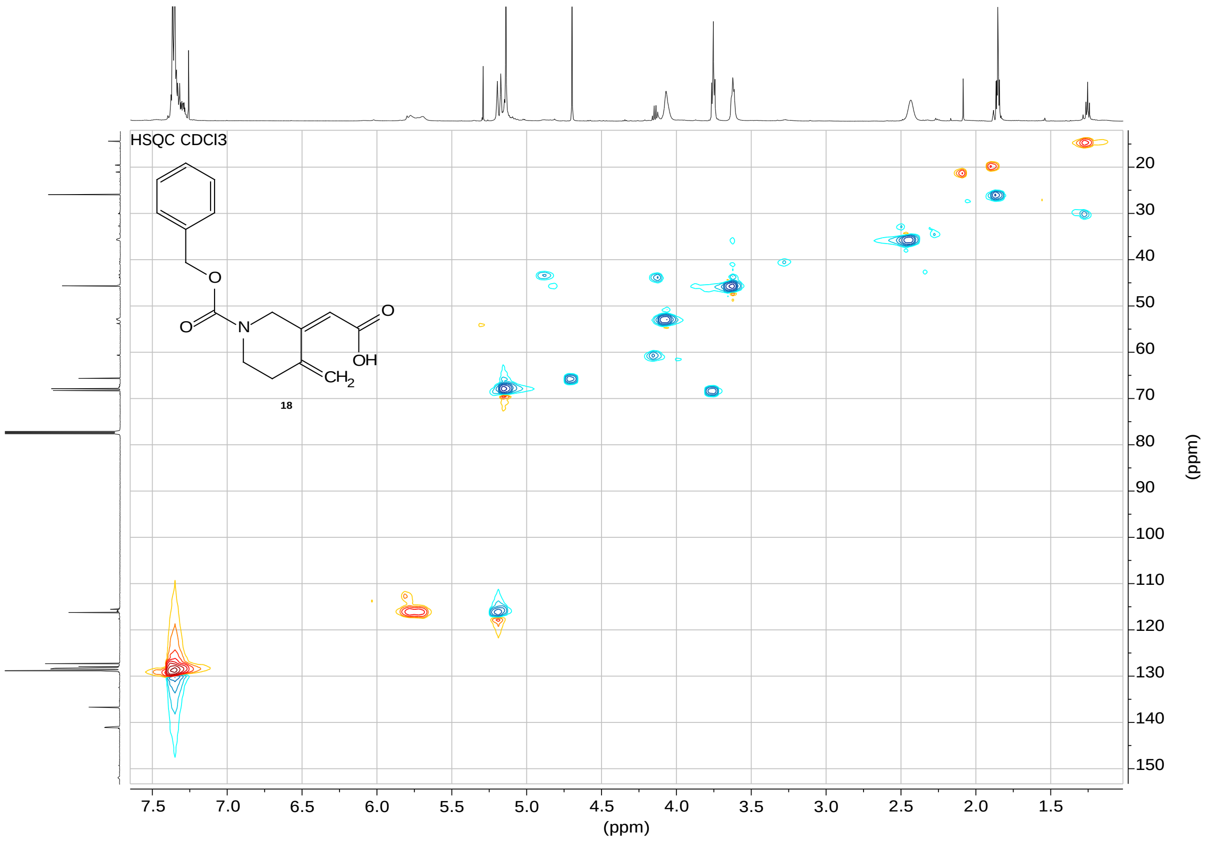
HSQC CDCl3
O
O
N
O
OH
CH2
18
(ppm)
(ppm)

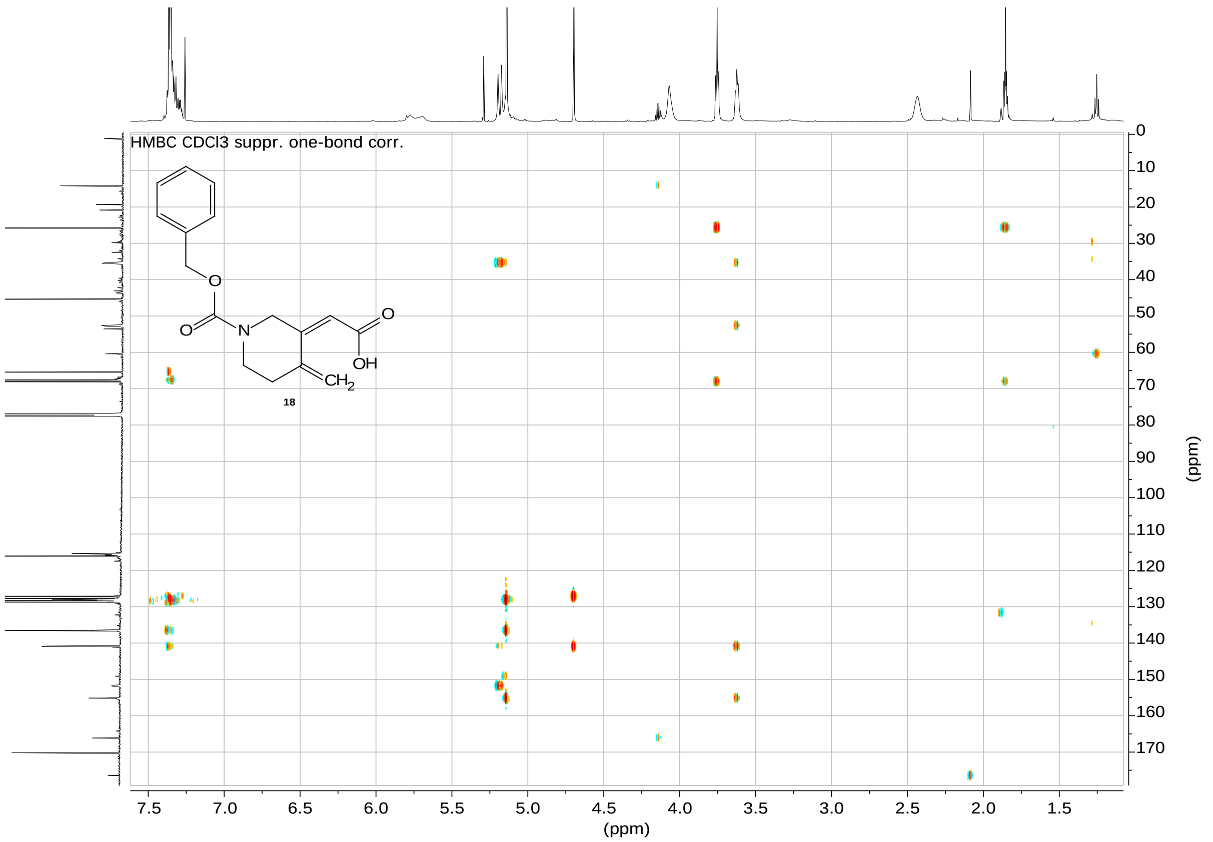
HMBC CDCl3 suppr. one-bond corr.
O
N
O
O
OH
CH2
18
7.5 7.0 6.5 6.0 5.5 5.0 4.5 4.0 3.5 3.0 2.5 2.0 1.5
(ppm)
0 10 20 30 40 50 60 70 80 90 100 110 120 130 140 150 160 170
(ppm)

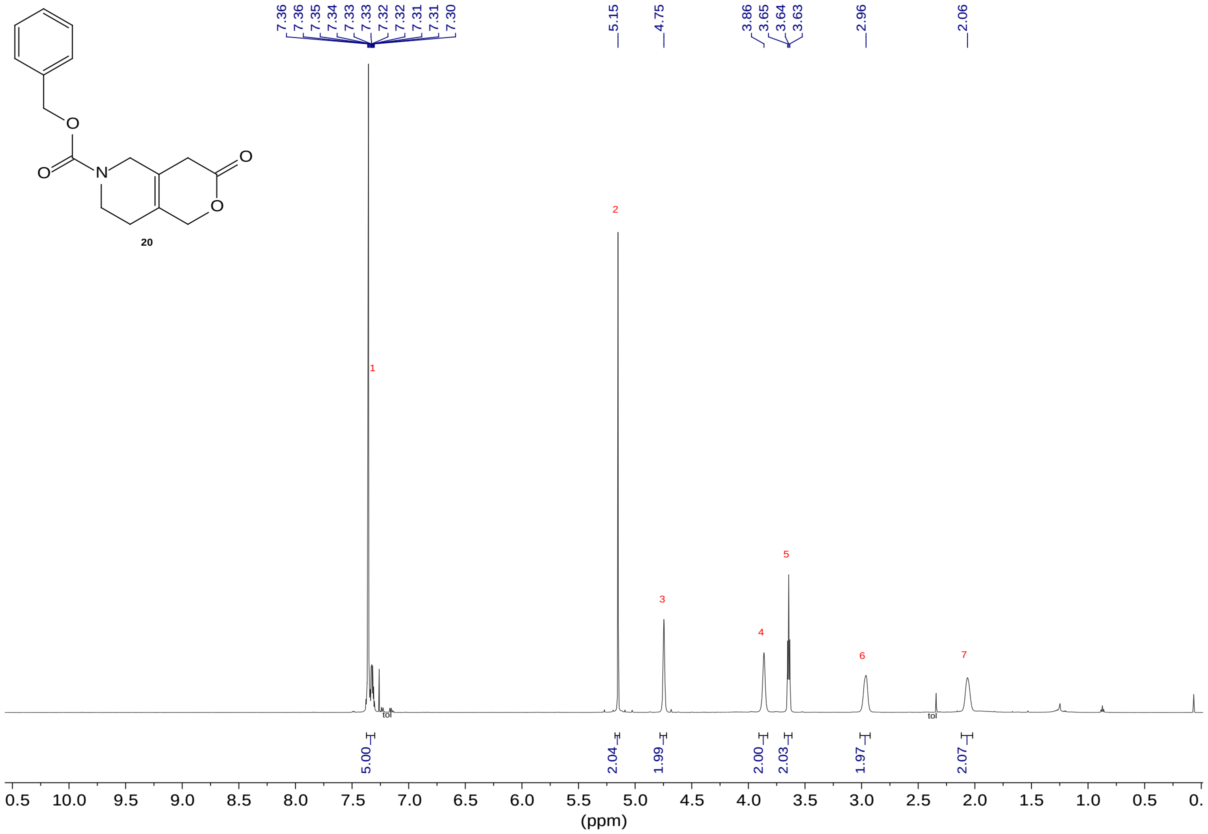
7.36
7.36
7.35
7.34
7.33
7.33
7.32
7.32
7.31
7.31
7.30
5.15
4.75
3.86
3.65
3.64
3.63
2.96
2.06
20
tol
tol
5.00
2.04
1.99
2.00
2.03
1.97
2.07
(ppm)

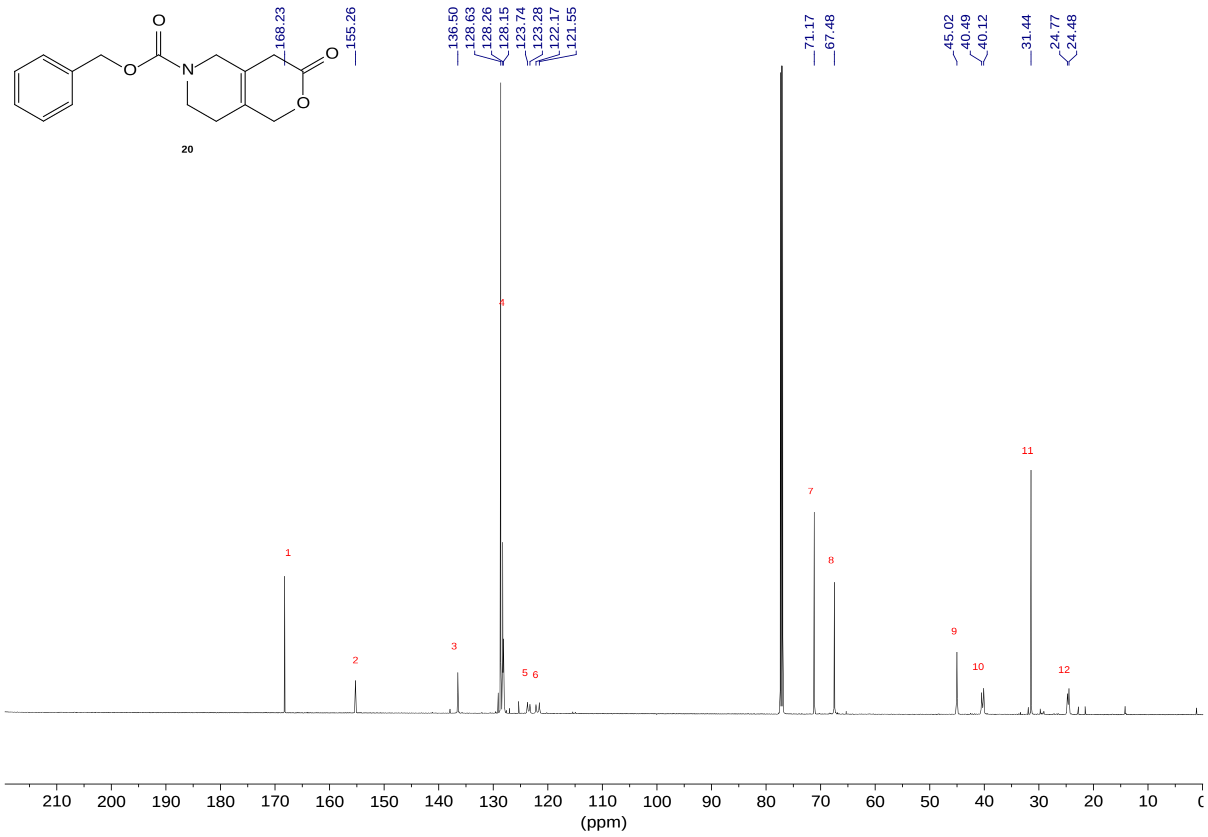
168.23
155.26
136.50
128.63
128.26
128.15
123.74
123.28
122.17
121.55
71.17
67.48
45.02
40.49
40.12
31.44
24.77
24.48
20
(ppm)

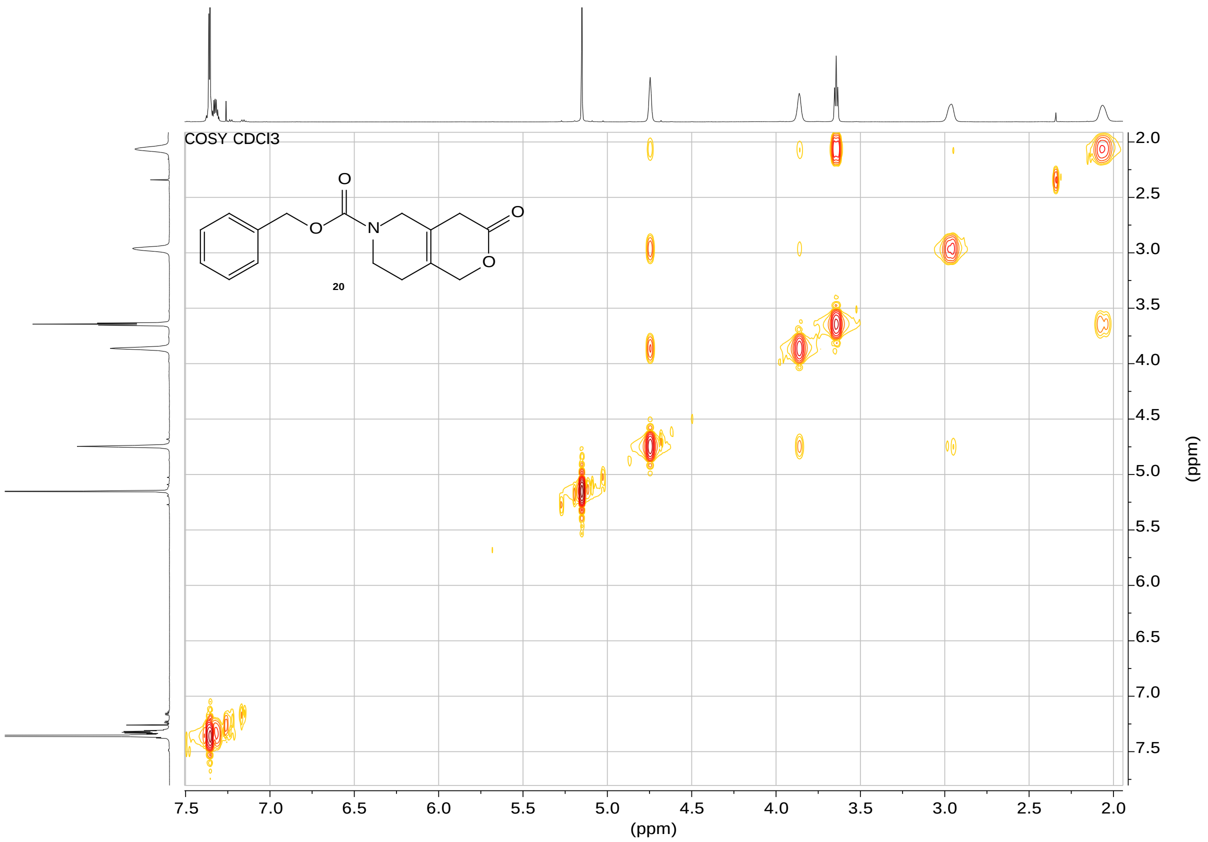
COSY CDCl3
20
7.5 7.0 6.5 6.0 5.5 5.0 4.5 4.0 3.5 3.0 2.5 2.0
(ppm)
2.0 2.5 3.0 3.5 4.0 4.5 5.0 5.5 6.0 6.5 7.0 7.5
(ppm)

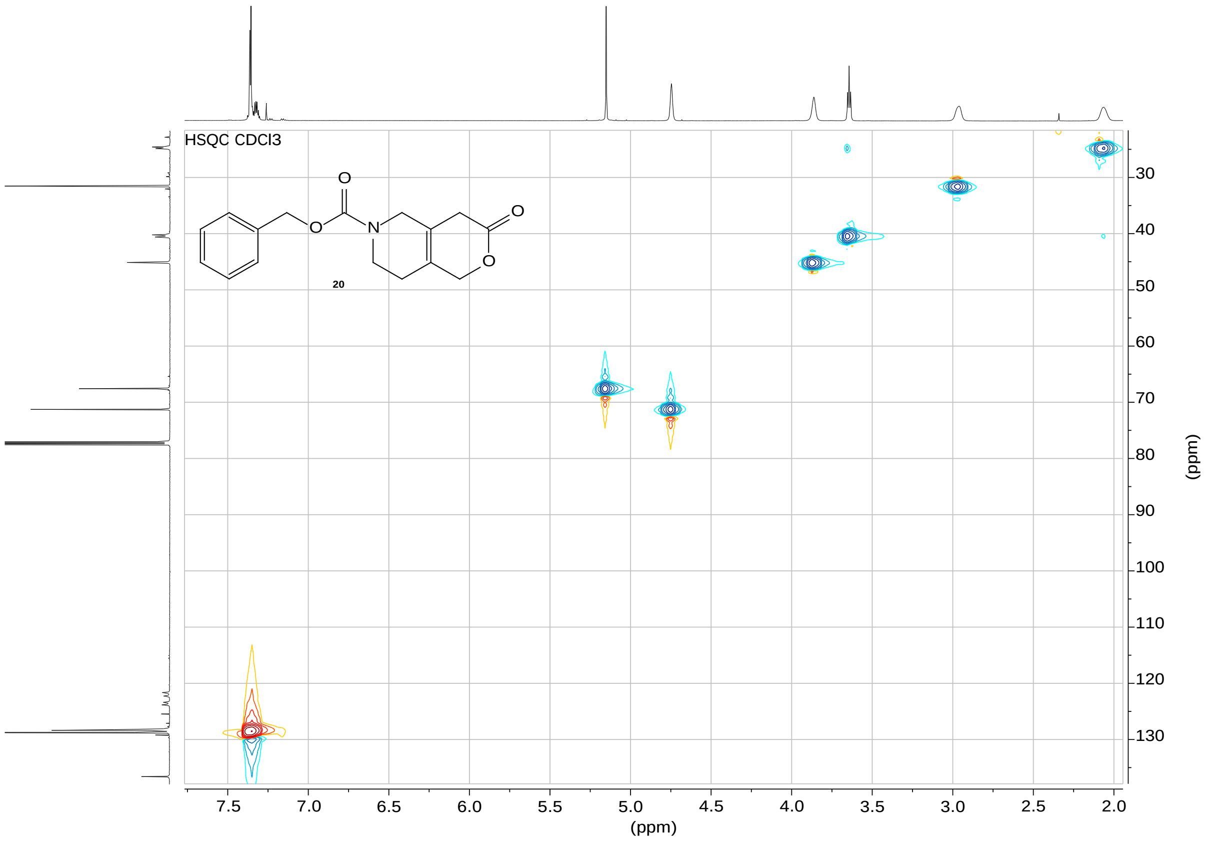
HSQC CDCl3
20
7.5 7.0 6.5 6.0 5.5 5.0 4.5 4.0 3.5 3.0 2.5 2.0
(ppm)
30 40 50 60 70 80 90 100 110 120 130
(ppm)

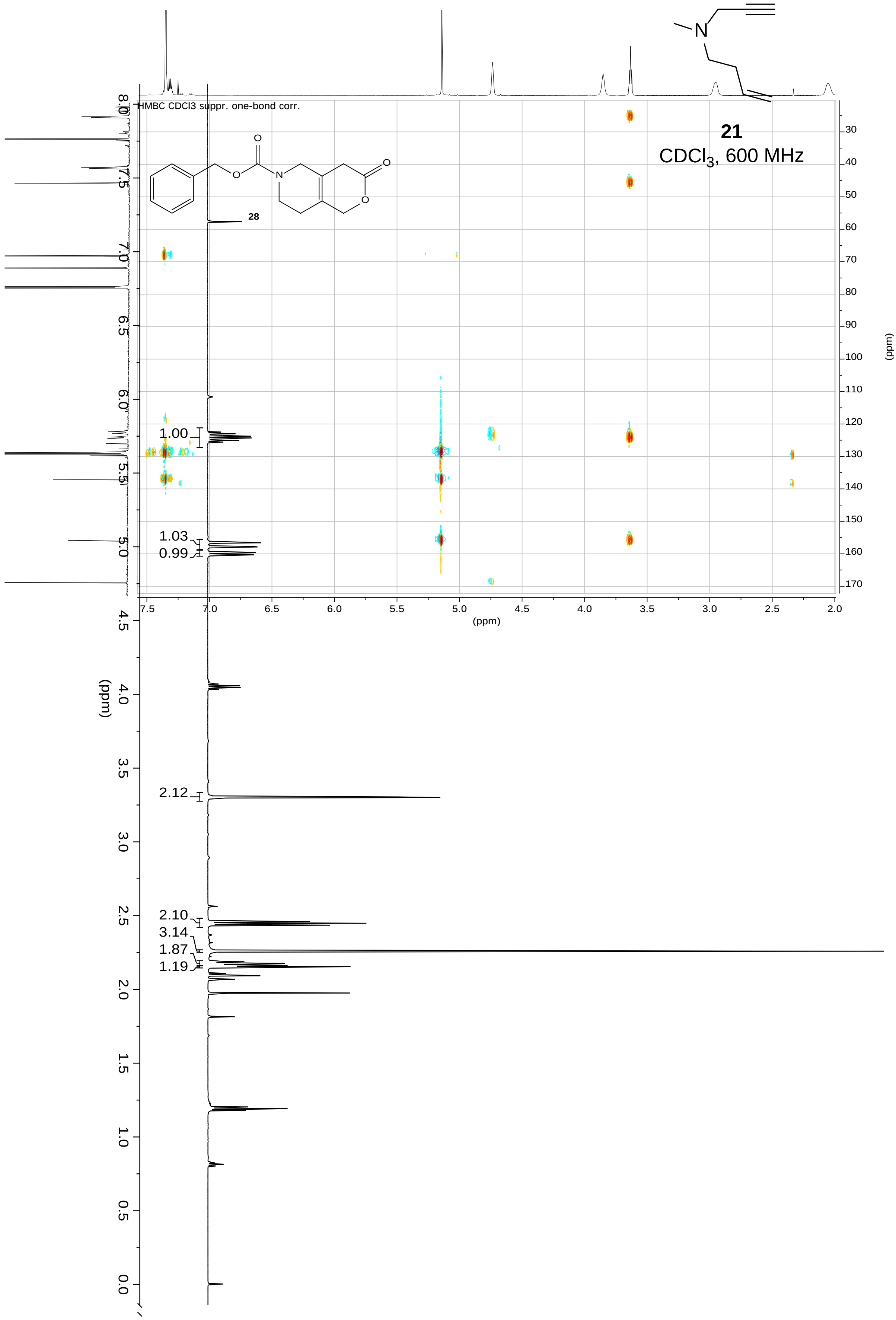
HMBC CDCl3 suppr. one-bond corr.
21
CDCl3, 600 MHz
28
(ppm)
(ppm)
(ppm)

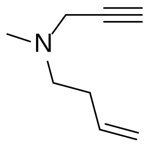

21

$CDCl_3$, 151 MHz

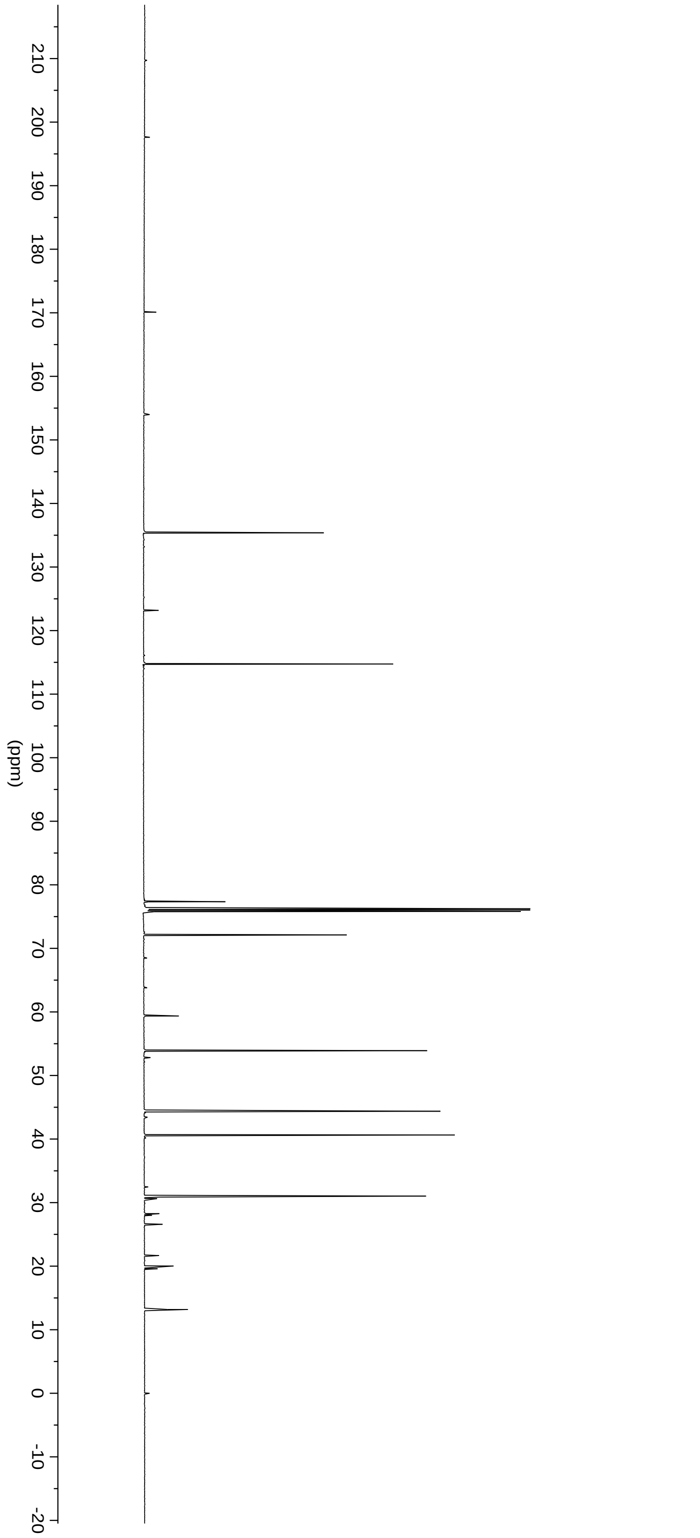

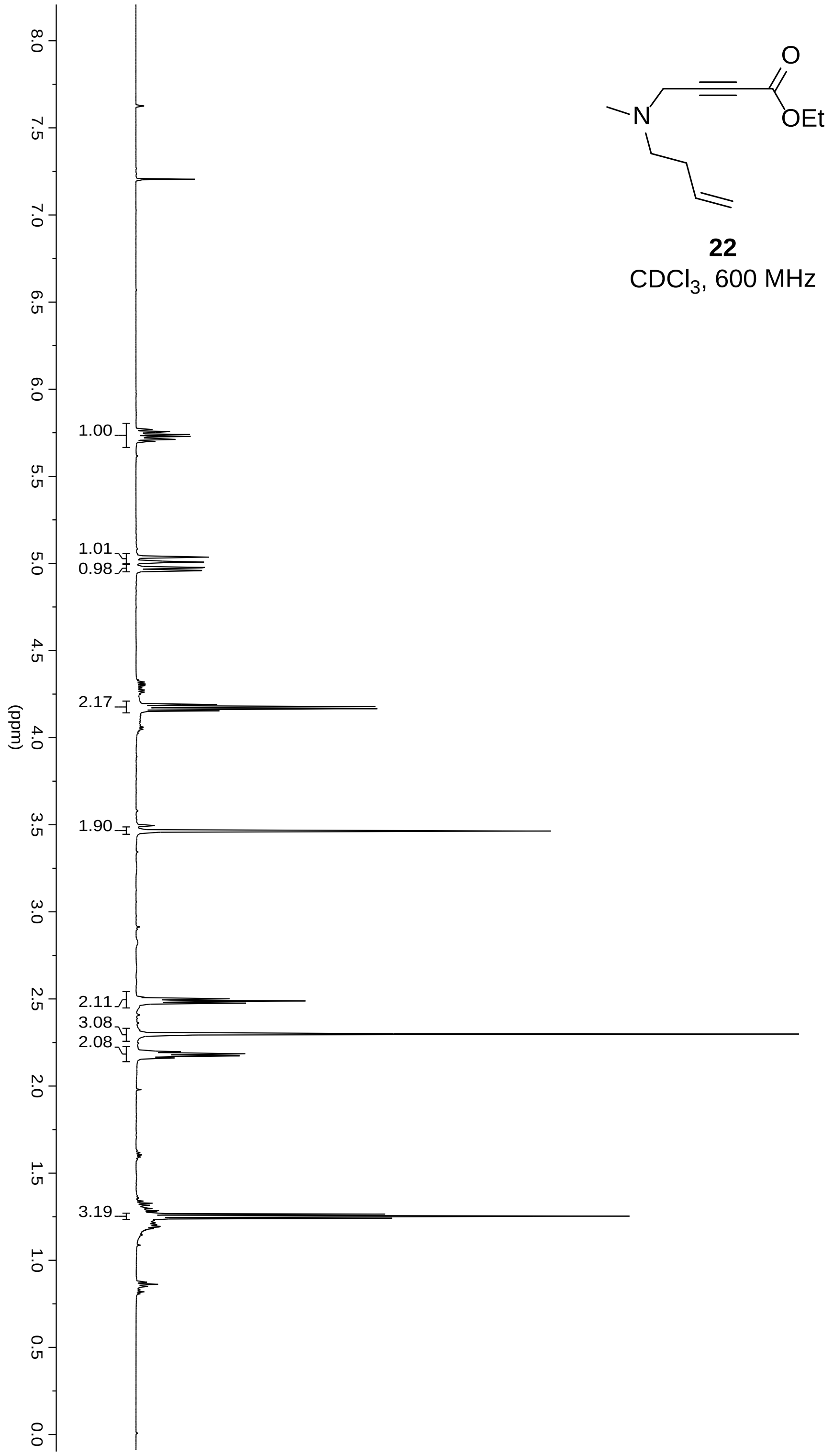

22
$CDCl_3$, 600 MHz

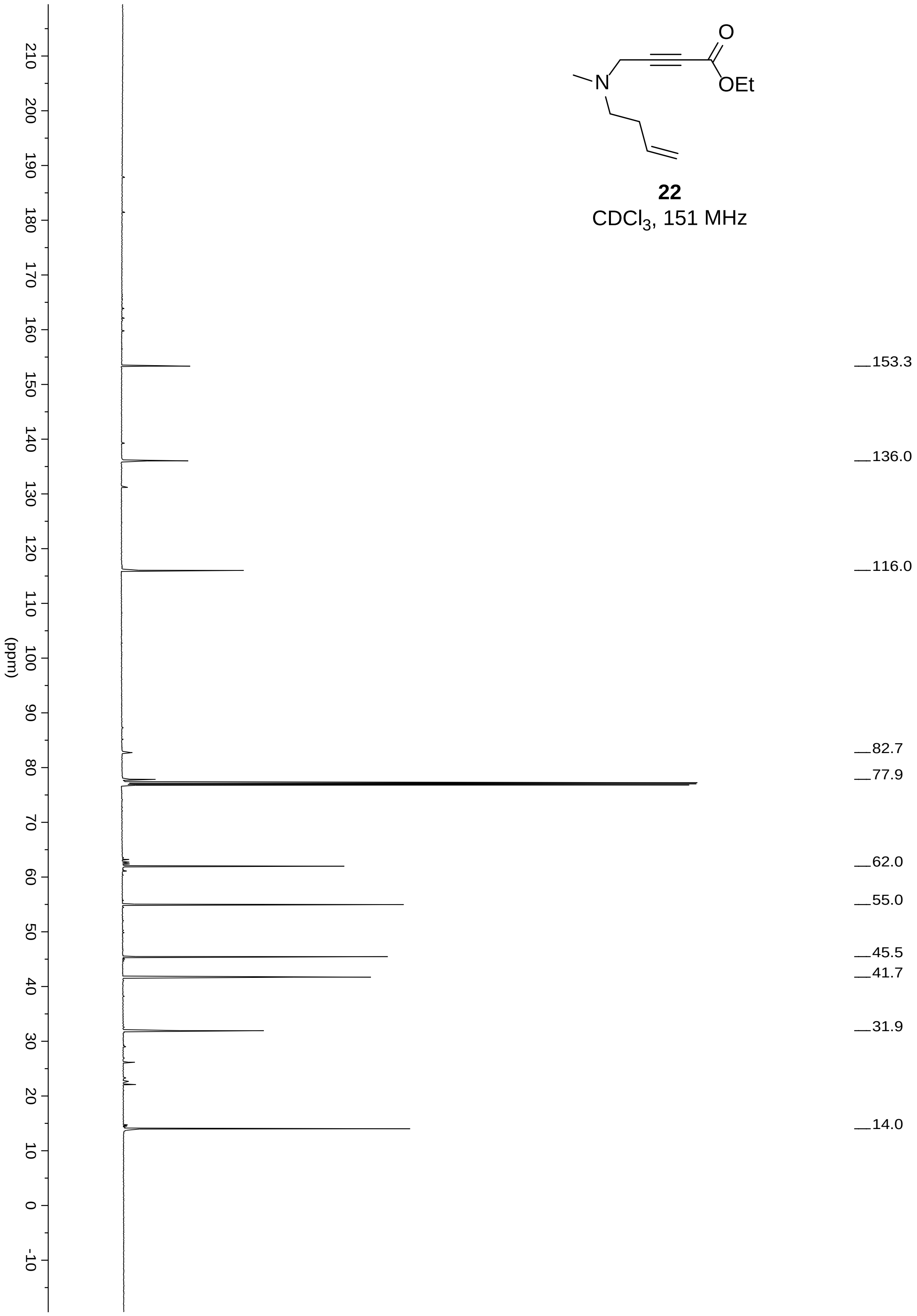
O
N
OEt
22
$CDCl_3$, 151 MHz
153.3
136.0
116.0
82.7
77.9
62.0
55.0
45.5
41.7
31.9
14.0
210
200
190
180
170
160
150
140
130
120
110
100
(ppm)
90
80
70
60
50
40
30
20
10
0
-10

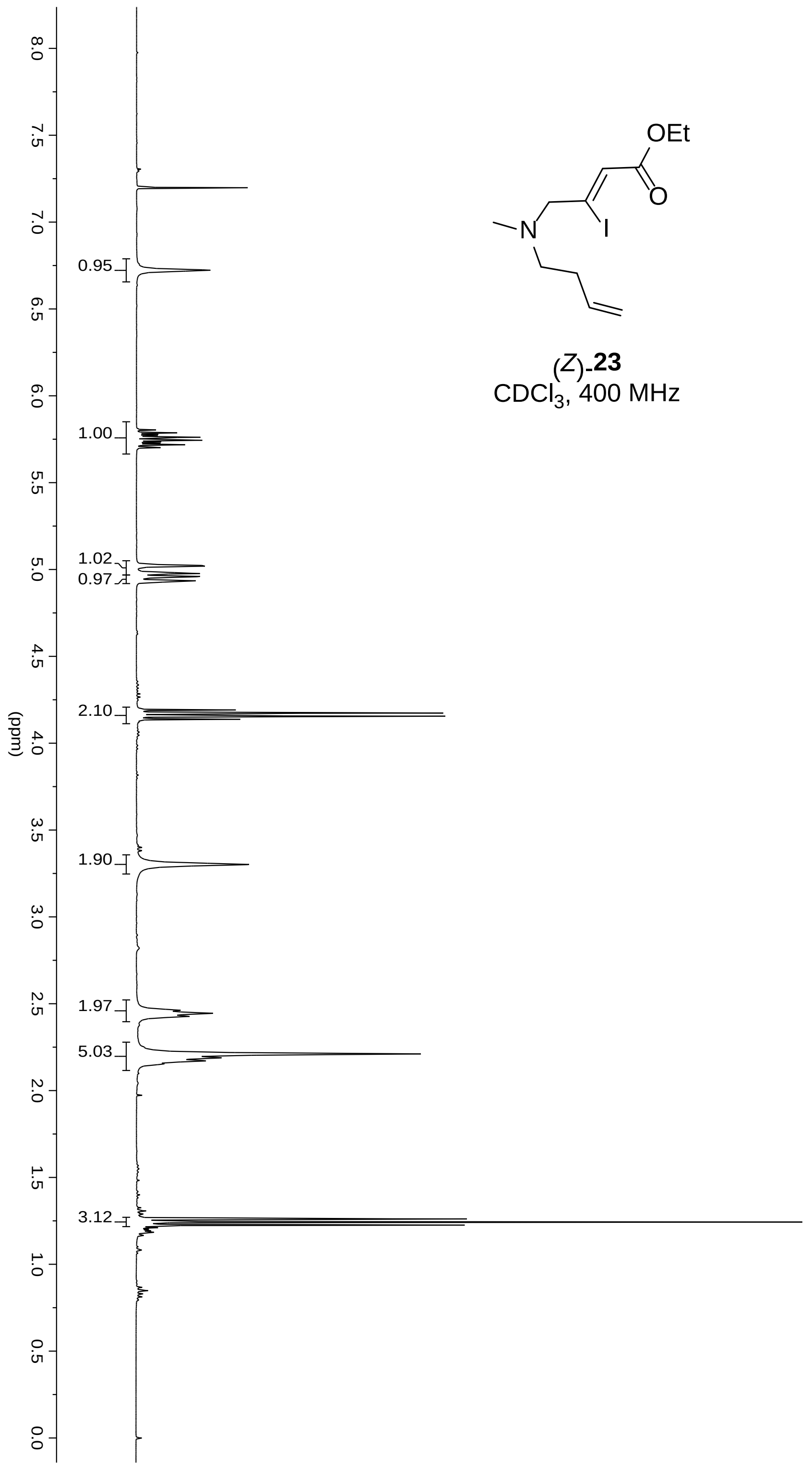
OEt
O
N
I
(Z)-**23**
$CDCl_3$, 400 MHz
(ppm)
8.0
7.5
7.0
6.5
6.0
5.5
5.0
4.5
4.0
3.5
3.0
2.5
2.0
1.5
1.0
0.5
0.0
0.95
1.00
1.02
0.97
2.10
1.90
1.97
5.03
3.12

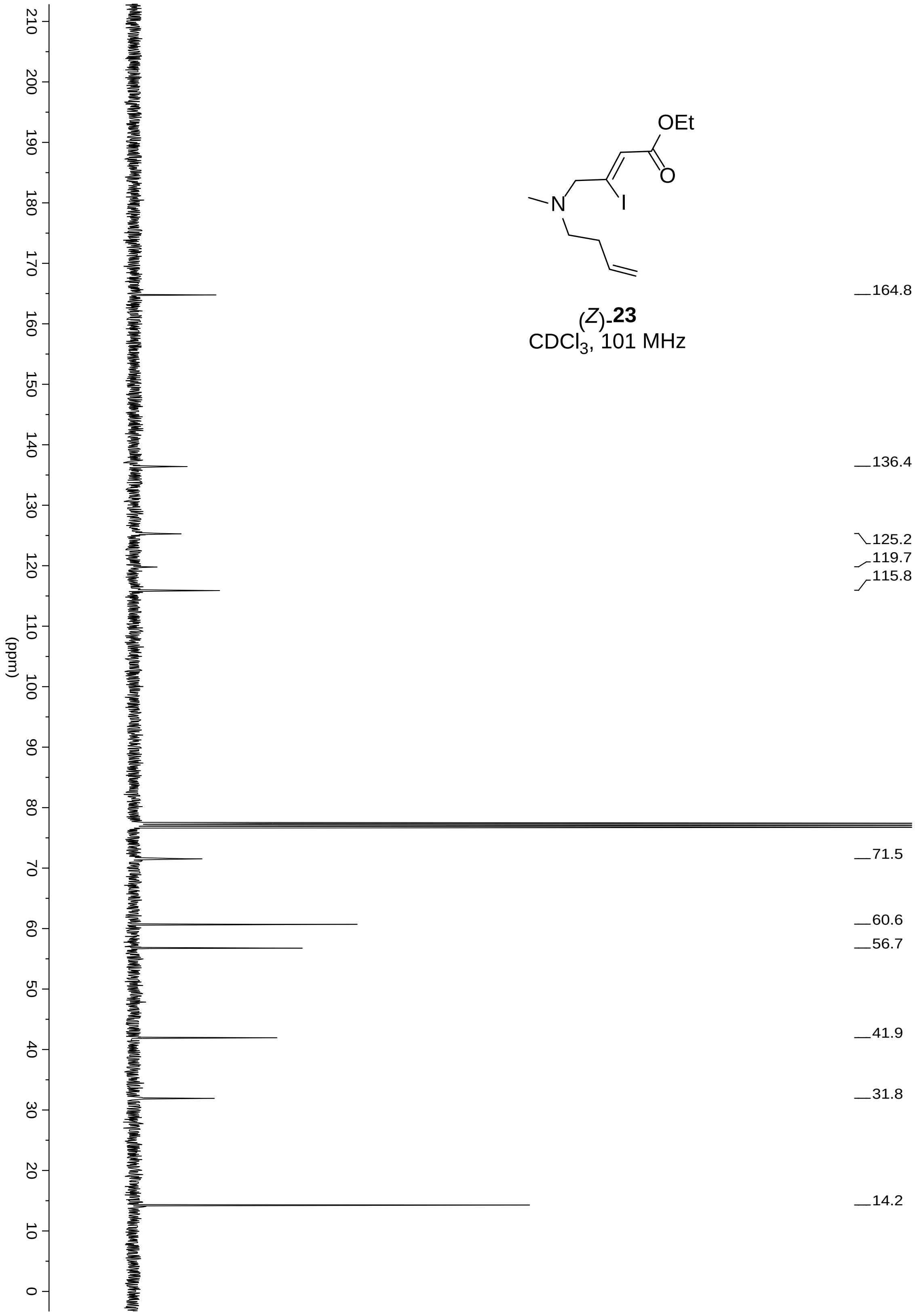

(*Z*)-**23**
$CDCl_3$, 101 MHz

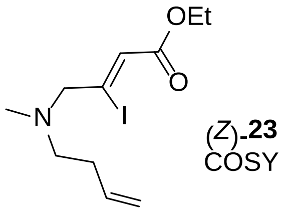

(*Z*)-**23**
COSY

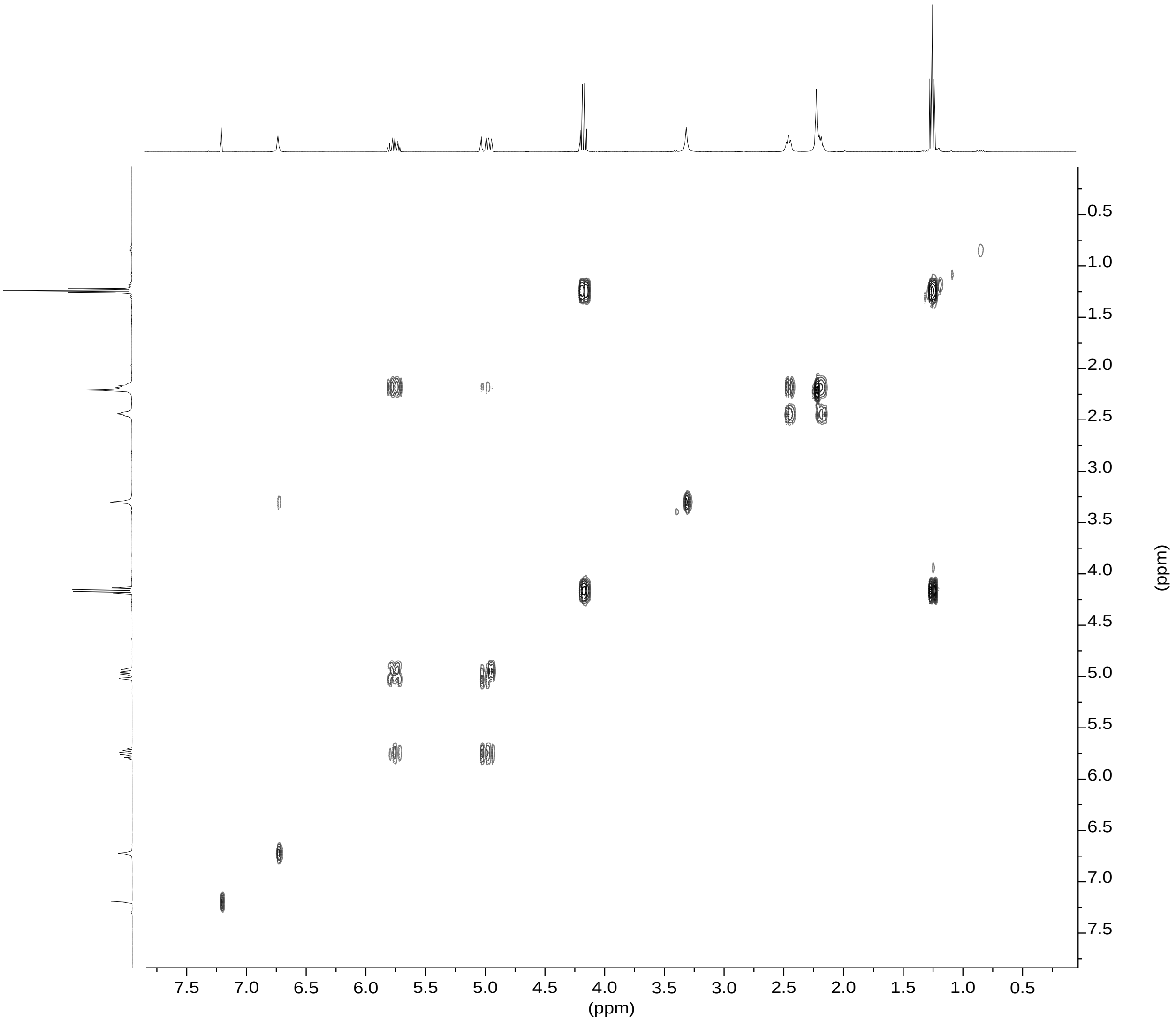

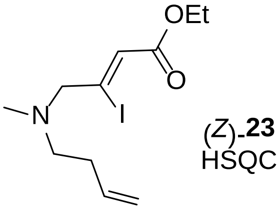

(*Z*)-**23**
HSQC

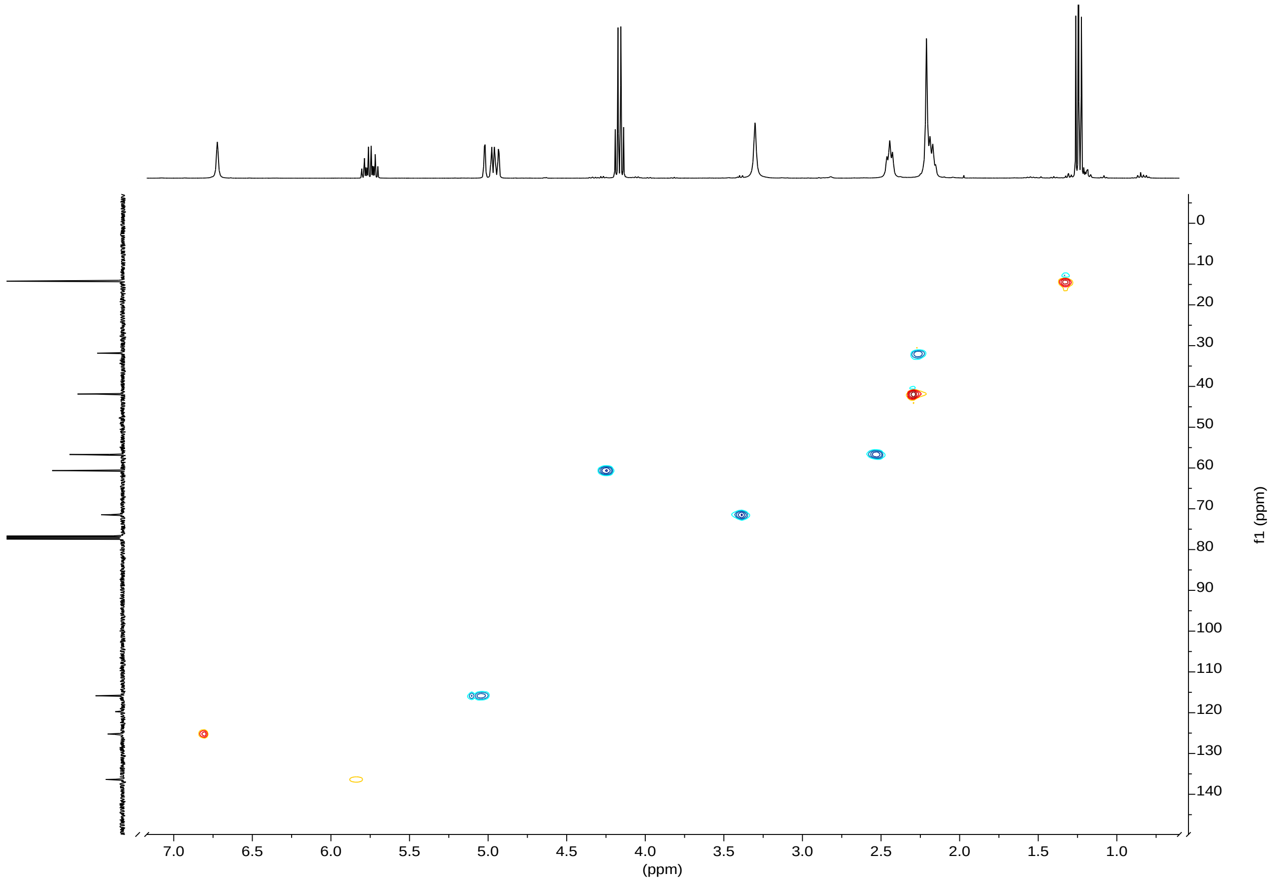

0
10
20
30
40
50
60
70
80
90
100
110
120
130
140
f1 (ppm)
7.0
6.5
6.0
5.5
5.0
4.5
4.0
3.5
3.0
2.5
2.0
1.5
1.0
(ppm)

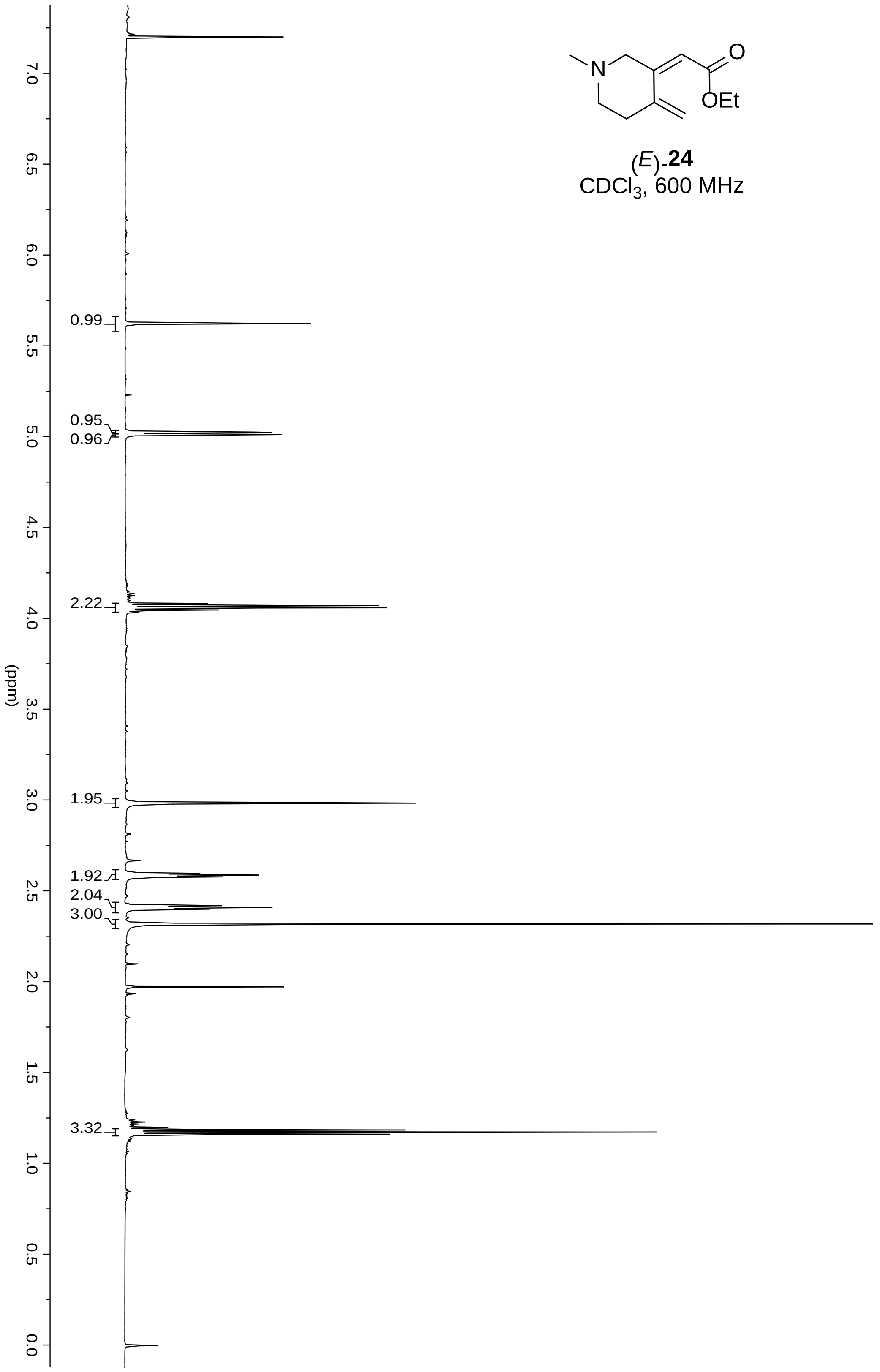
N
O
OEt
(*E*)-**24**
$CDCl_3$, 600 MHz
(ppm)
7.0
6.5
6.0
5.5
5.0
4.5
4.0
3.5
3.0
2.5
2.0
1.5
1.0
0.5
0.0
0.99
0.95
0.96
2.22
1.95
1.92
2.04
3.00
3.32

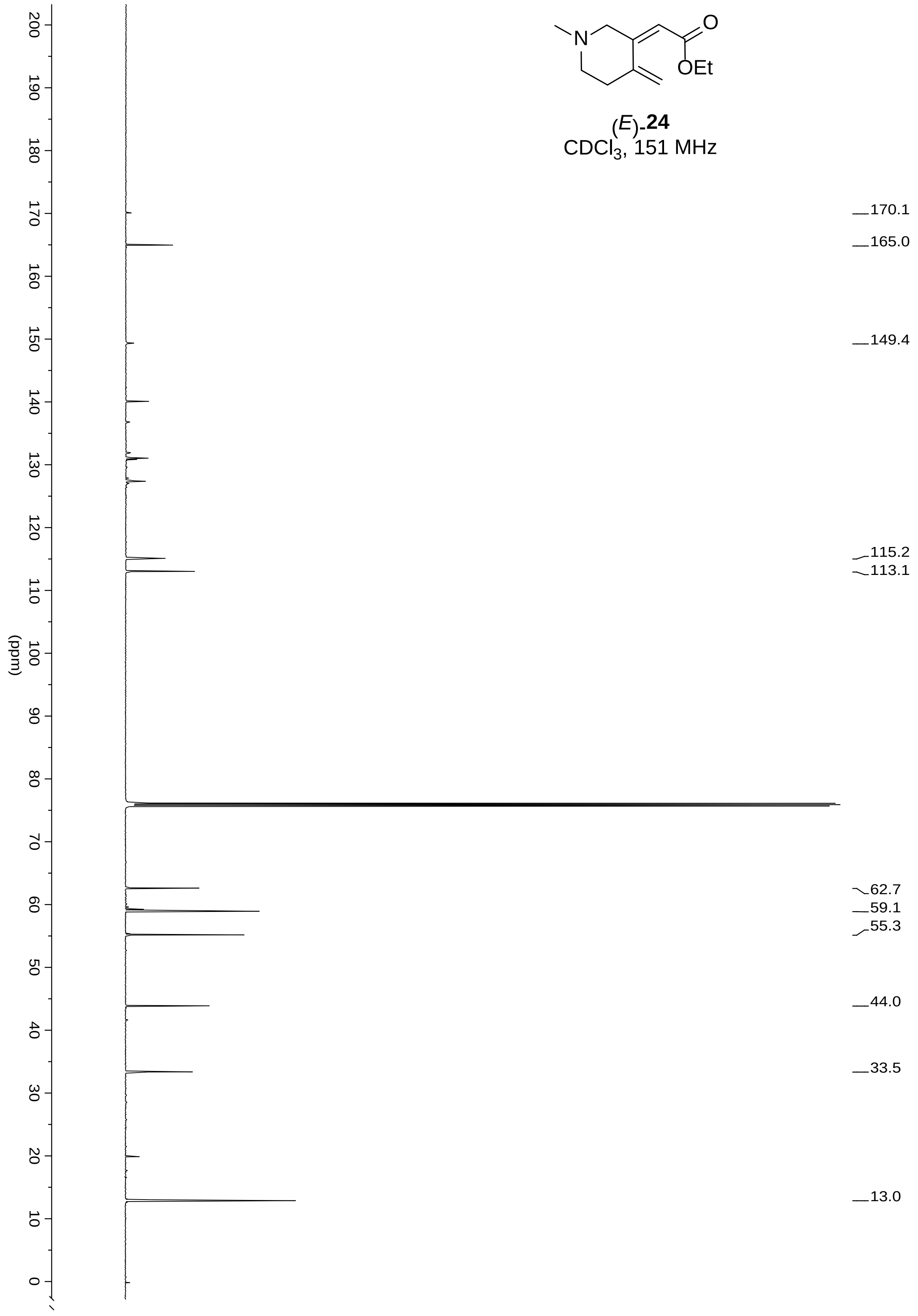
O
N
OEt
(E)-24
CDCl3, 151 MHz
170.1
165.0
149.4
115.2
113.1
62.7
59.1
55.3
44.0
33.5
13.0
(ppm)
200
190
180
170
160
150
140
130
120
110
100
90
80
70
60
50
40
30
20
10
0

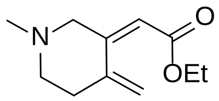
N
O
OEt

(*E*)-**24**
COSY

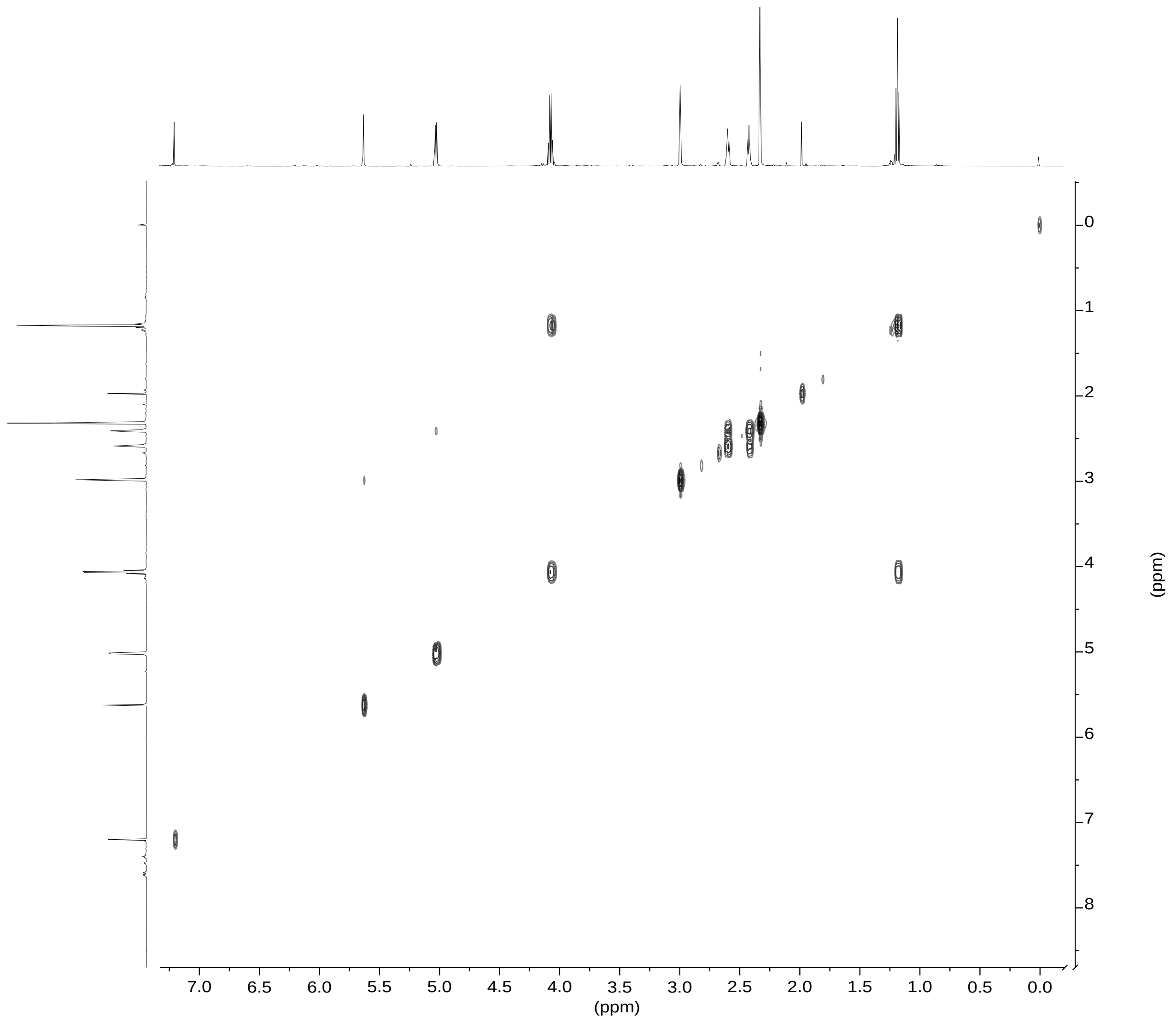
0
1
2
3
4
5
6
7
8
(ppm)
7.0
6.5
6.0
5.5
5.0
4.5
4.0
3.5
3.0
2.5
2.0
1.5
1.0
0.5
0.0
(ppm)

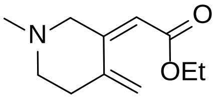

(*E*)-**24**
HSQC

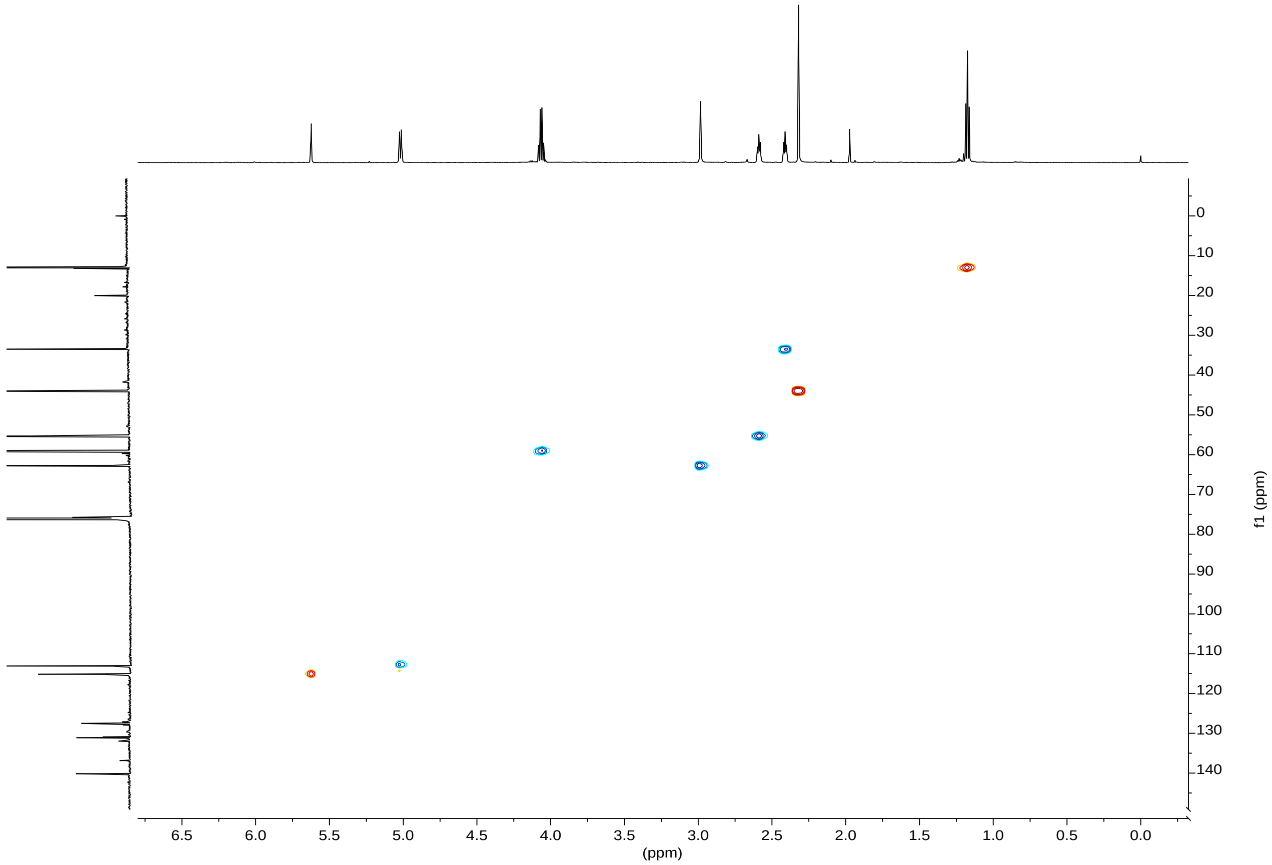

0
10
20
30
40
50
60
70
80
90
100
110
120
130
140
f1 (ppm)
6.5
6.0
5.5
5.0
4.5
4.0
3.5
3.0
2.5
2.0
1.5
1.0
0.5
0.0
(ppm)

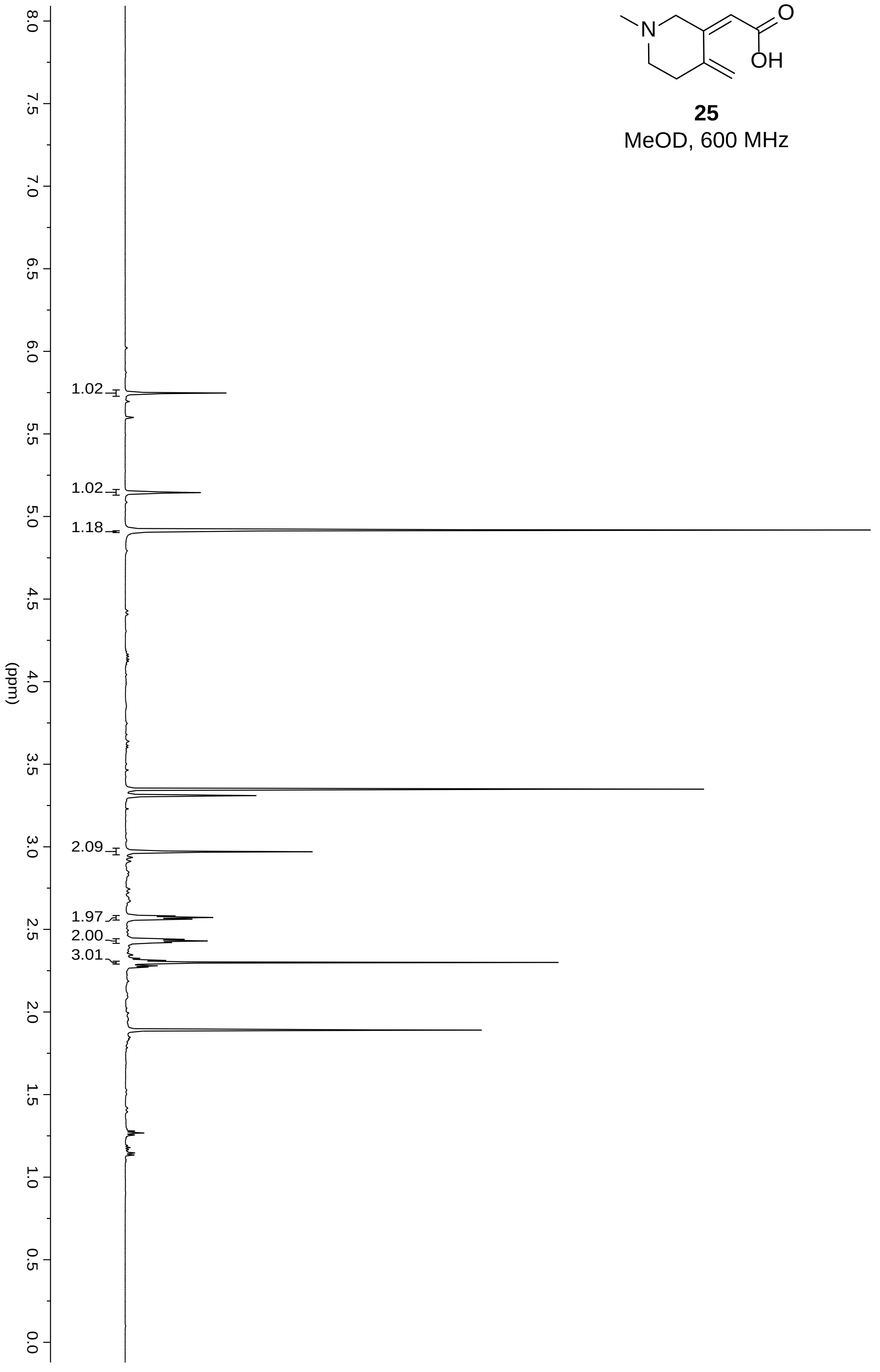
O
N
OH
25
MeOD, 600 MHz
1.02
1.02
1.18
2.09
1.97
2.00
3.01
8.0
7.5
7.0
6.5
6.0
5.5
5.0
4.5
4.0
3.5
3.0
2.5
2.0
1.5
1.0
0.5
0.0
(ppm)

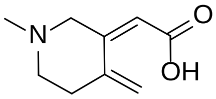
N
O
OH

25
COSY

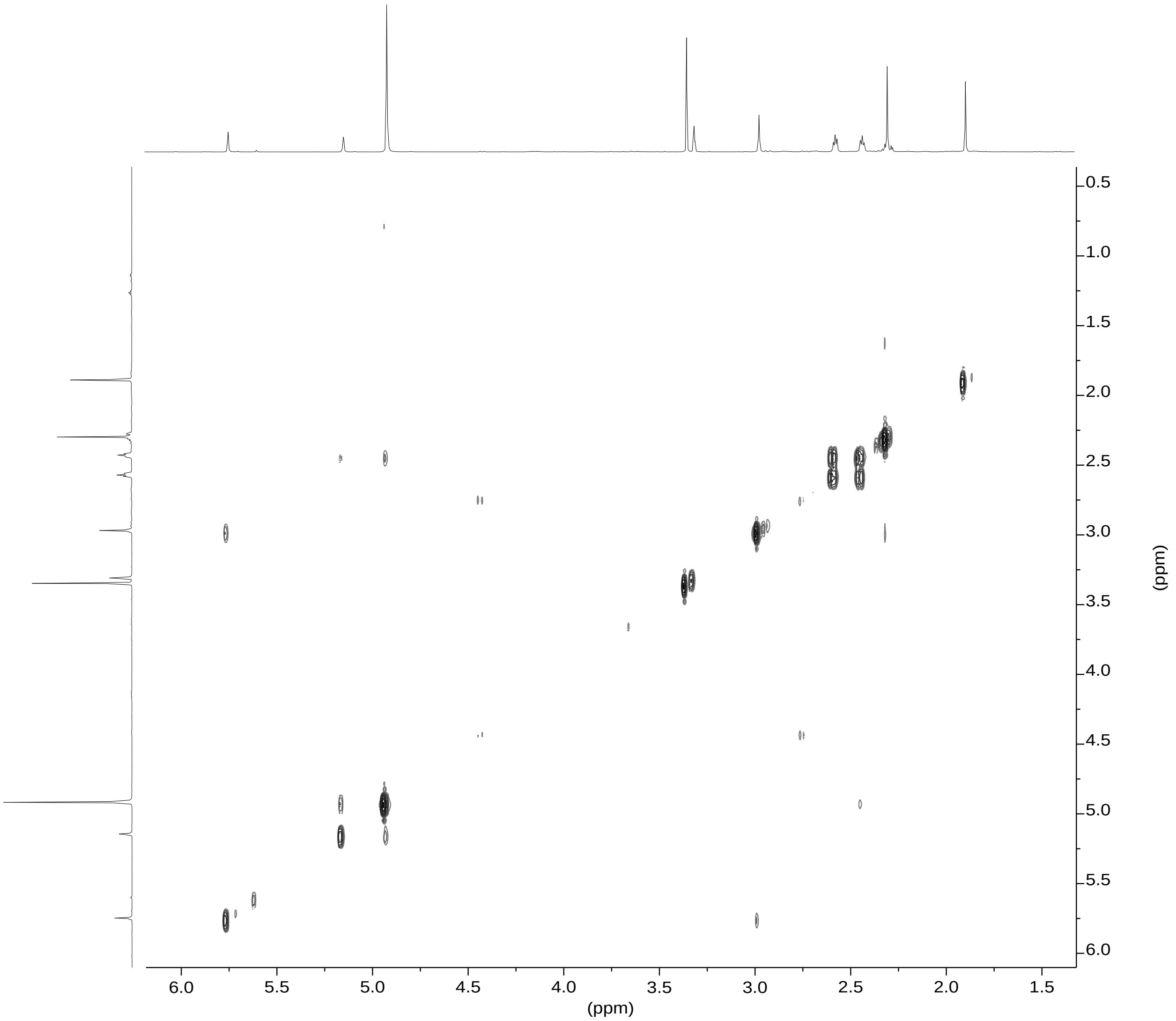
0.5
1.0
1.5
2.0
2.5
3.0
3.5
4.0
4.5
5.0
5.5
6.0
(ppm)
6.0 5.5 5.0 4.5 4.0 3.5 3.0 2.5 2.0 1.5
(ppm)

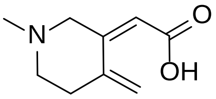
N
O
OH

25
HSQC

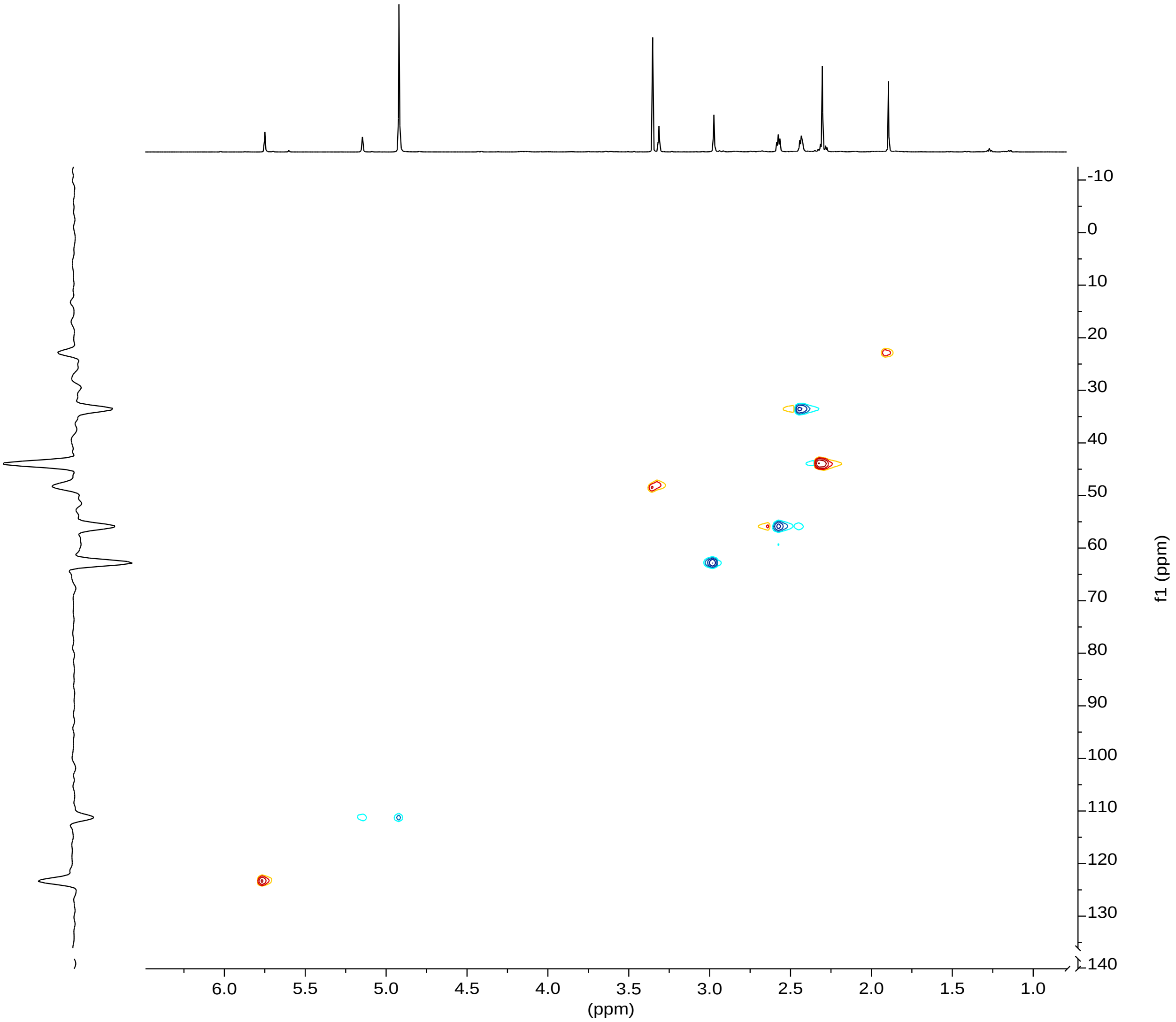
-10
0
10
20
30
40
50
60
70
80
90
100
110
120
130
140
f1 (ppm)
6.0
5.5
5.0
4.5
4.0
3.5
3.0
2.5
2.0
1.5
1.0
(ppm)

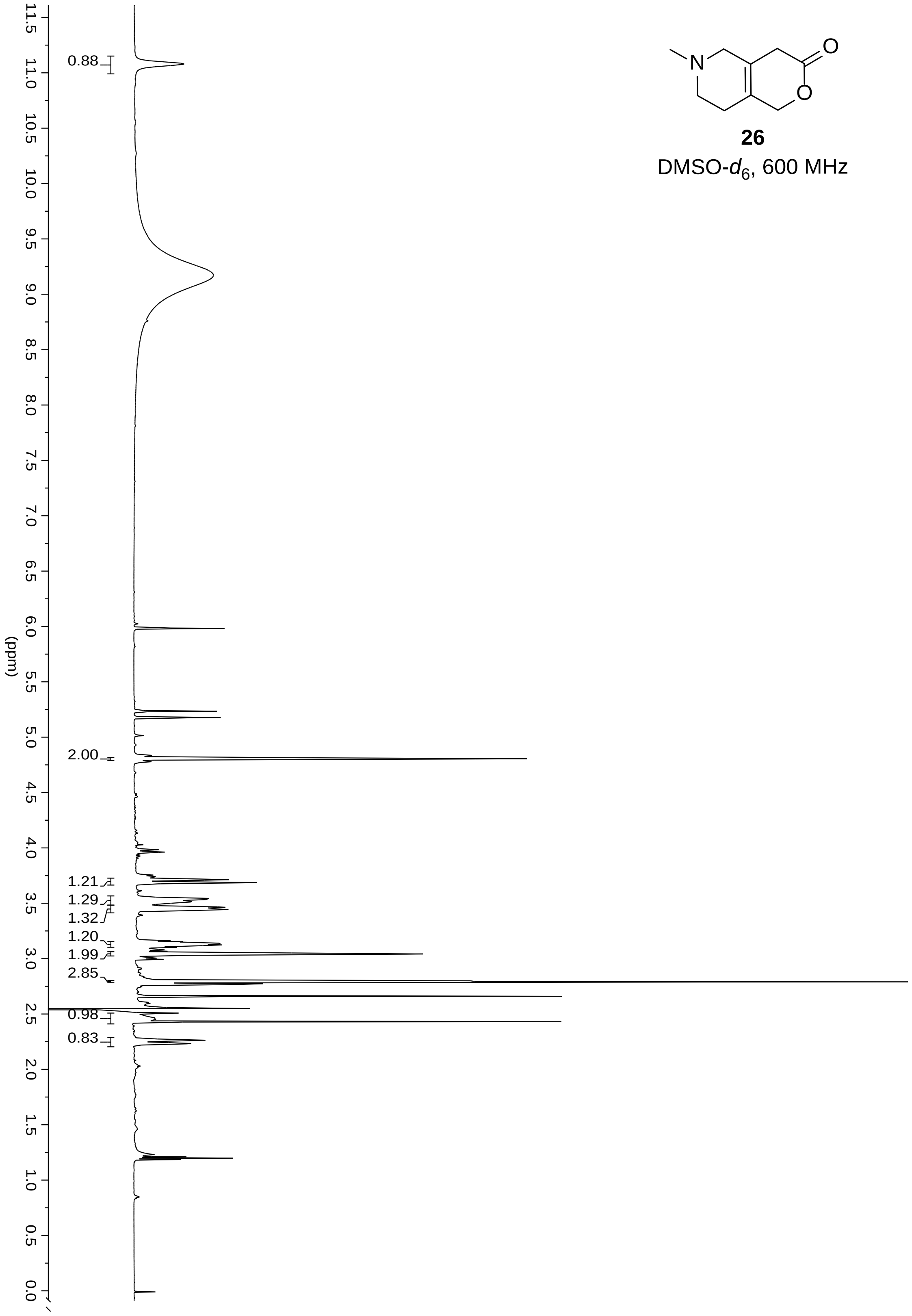

N
O
O
26
DMSO-d_6, 600 MHz
(ppm)
11.5
11.0
10.5
10.0
9.5
9.0
8.5
8.0
7.5
7.0
6.5
6.0
5.5
5.0
4.5
4.0
3.5
3.0
2.5
2.0
1.5
1.0
0.5
0.0
0.88
2.00
1.21
1.29
1.32
1.20
1.99
2.85
0.98
0.83

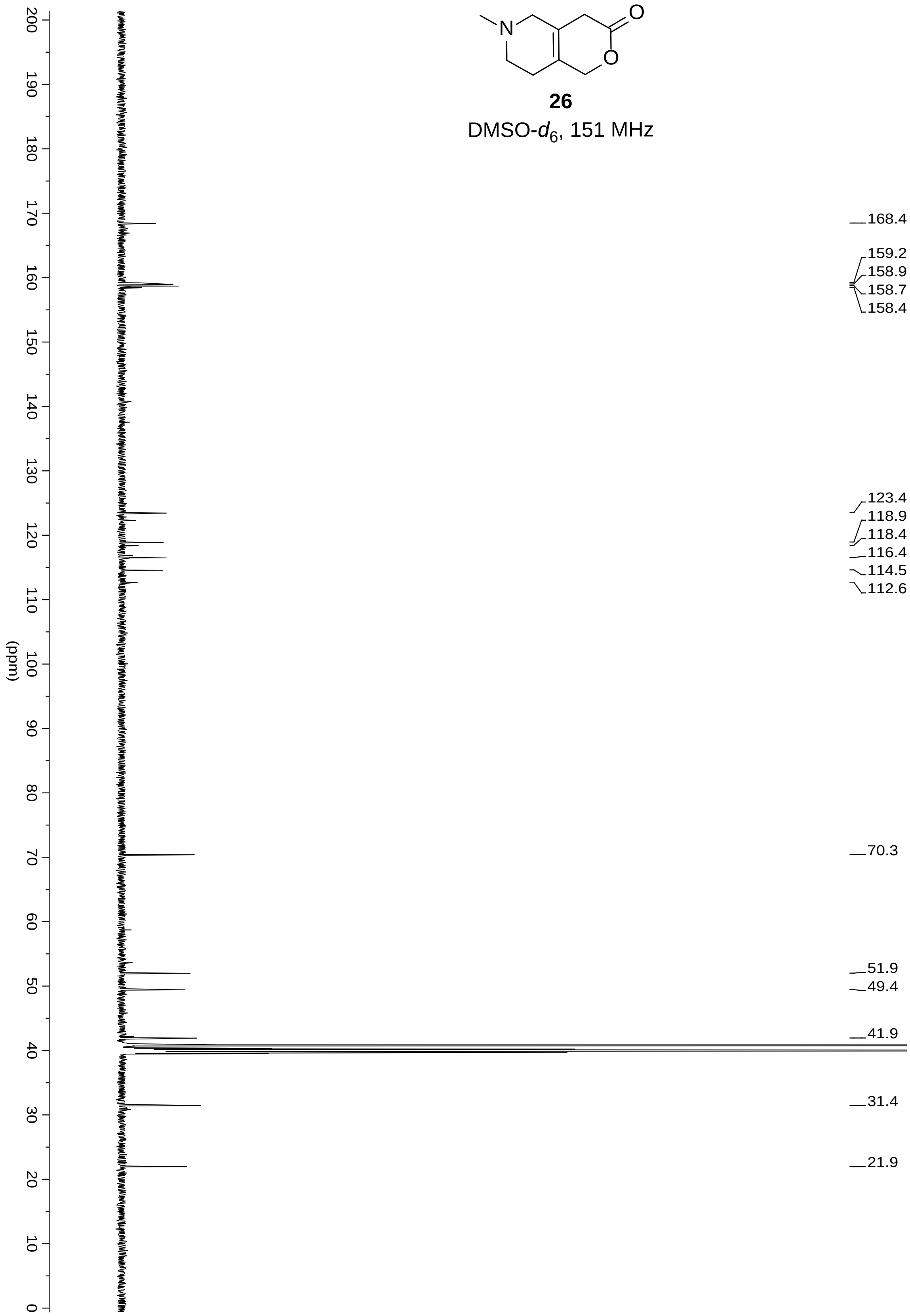

26

DMSO-d_6, 151 MHz

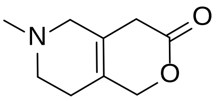

26
COSY

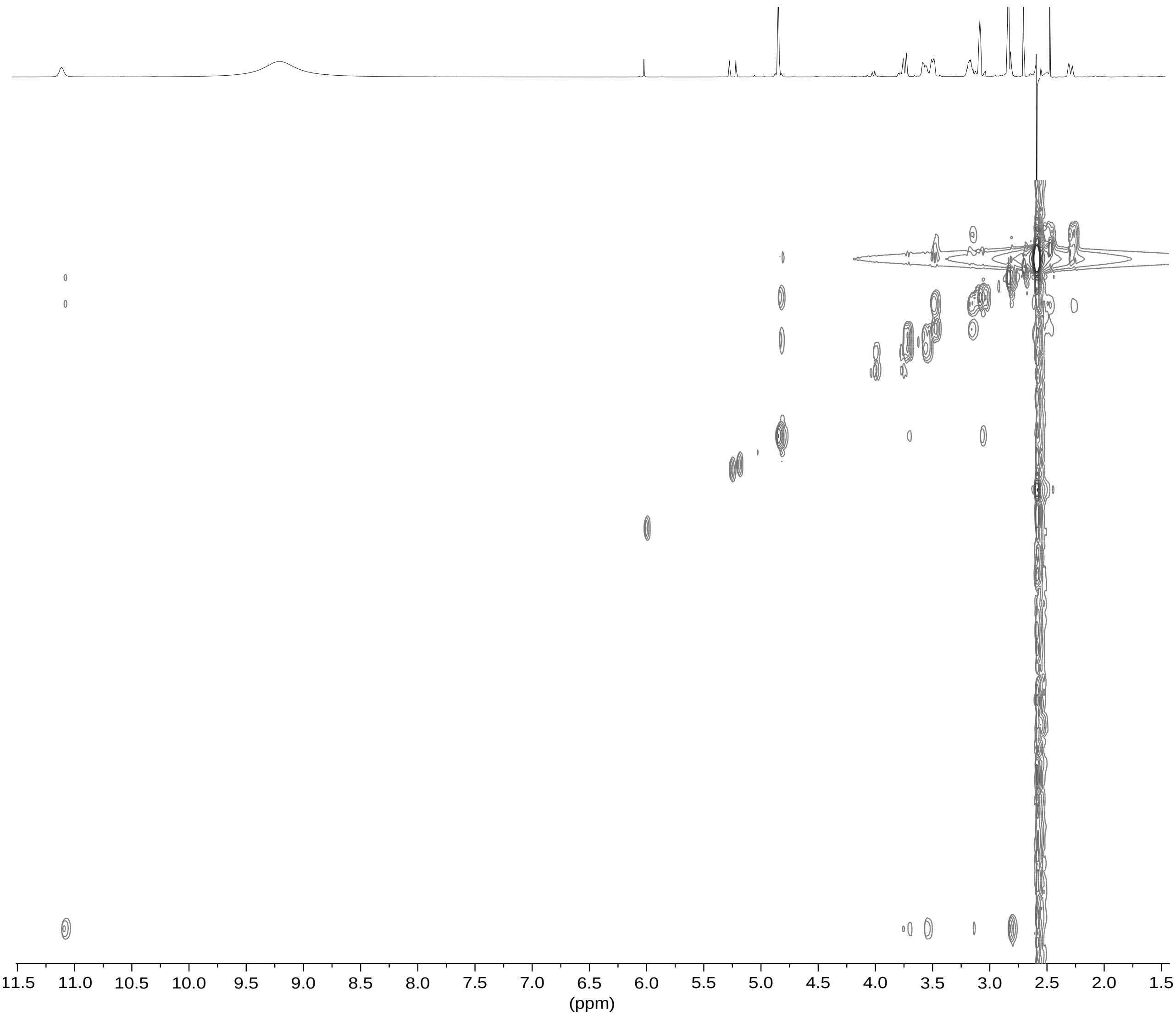

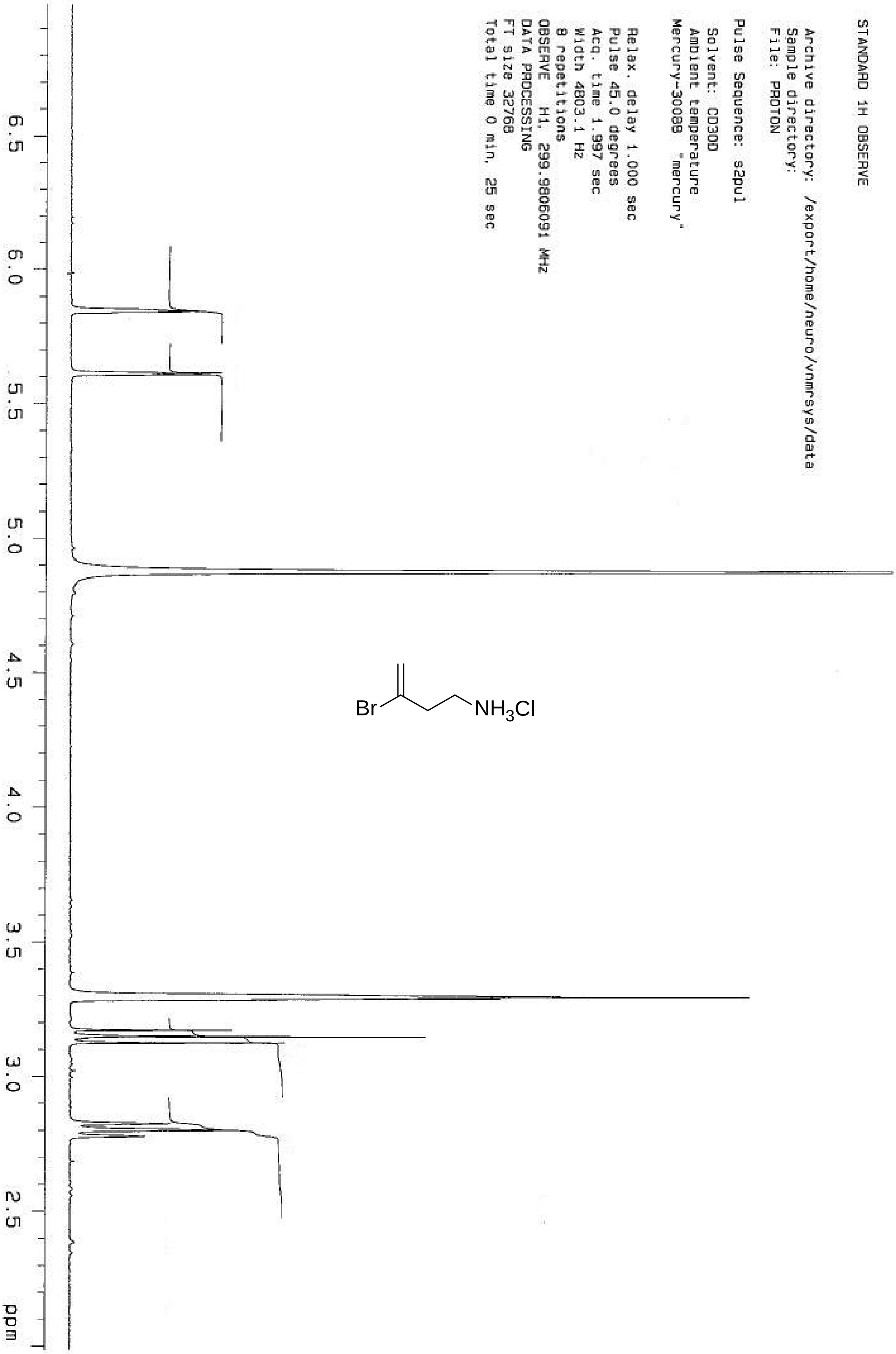
STANDARD 1H OBSERVE
Archive directory: /export/home/neuro/vnmrsys/data
Sample directory:
File: PROTON
Pulse Sequence: s2pul
Solvent: CD3OD
Ambient temperature
Mercury-300BB "mercury"
Relax. delay 1.000 sec
Pulse 45.0 degrees
Acq. time 1.997 sec
Width 4803.1 Hz
8 repetitions
OBSERVE H1, 299.9806091 MHz
DATA PROCESSING
FT size 32768
Total time 0 min, 25 sec
Br
NH3Cl
6.5
6.0
5.5
5.0
4.5
4.0
3.5
3.0
2.5
ppm

5

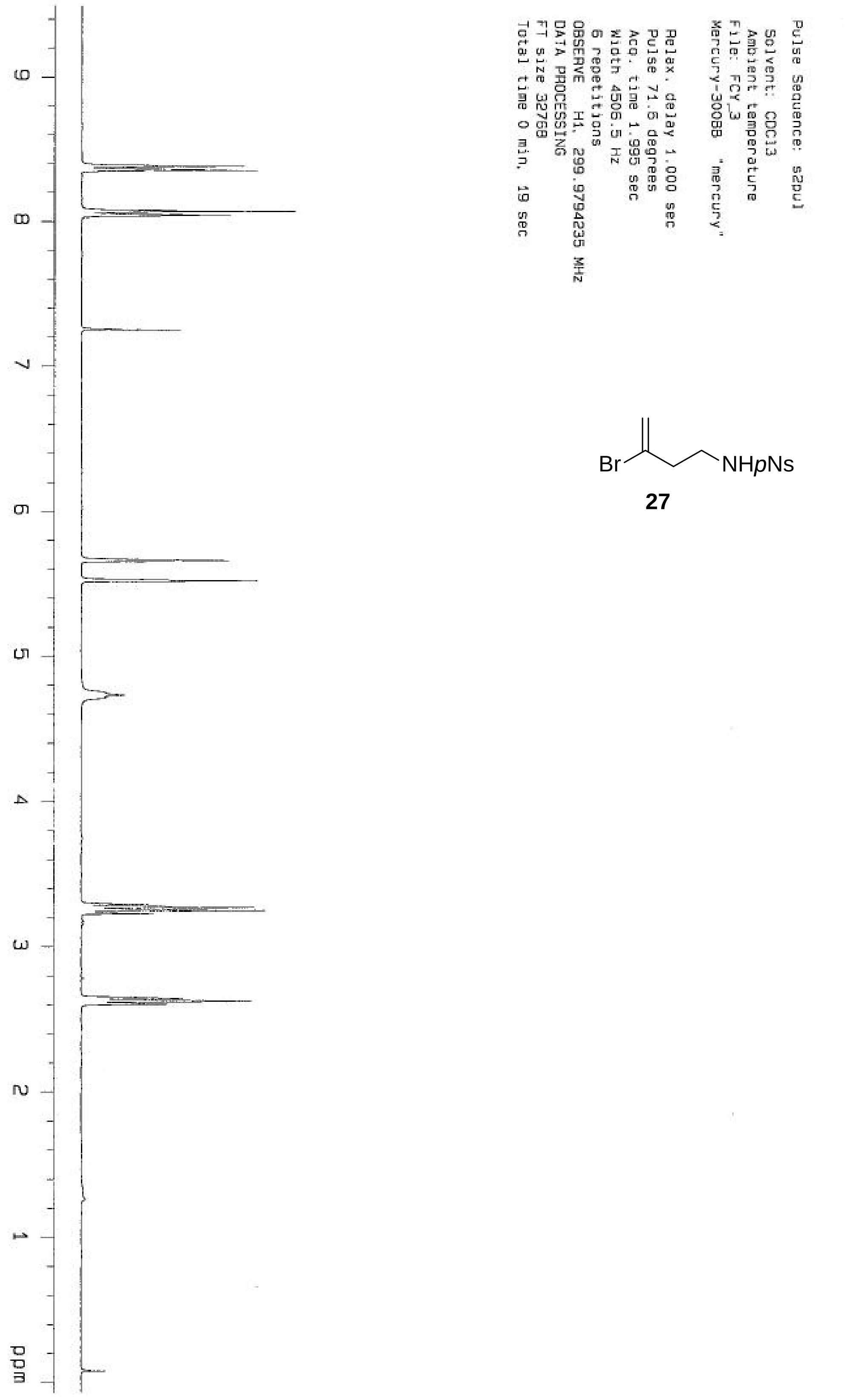
Pulse Sequence: s2pul
Solvent: CDCl3
Ambient temperature
File: FCY_3
Mercury-300BB "mercury"
Relax. delay 1.000 sec
Pulse 71.6 degrees
Acq. time 1.995 sec
Width 4506.5 Hz
6 repetitions
OBSERVE H1, 299.9794235 MHz
DATA PROCESSING
FT size 32768
Total time 0 min, 19 sec
9
8
7
6
5
4
3
2
1
ppm
Br
NH*p*Ns

27

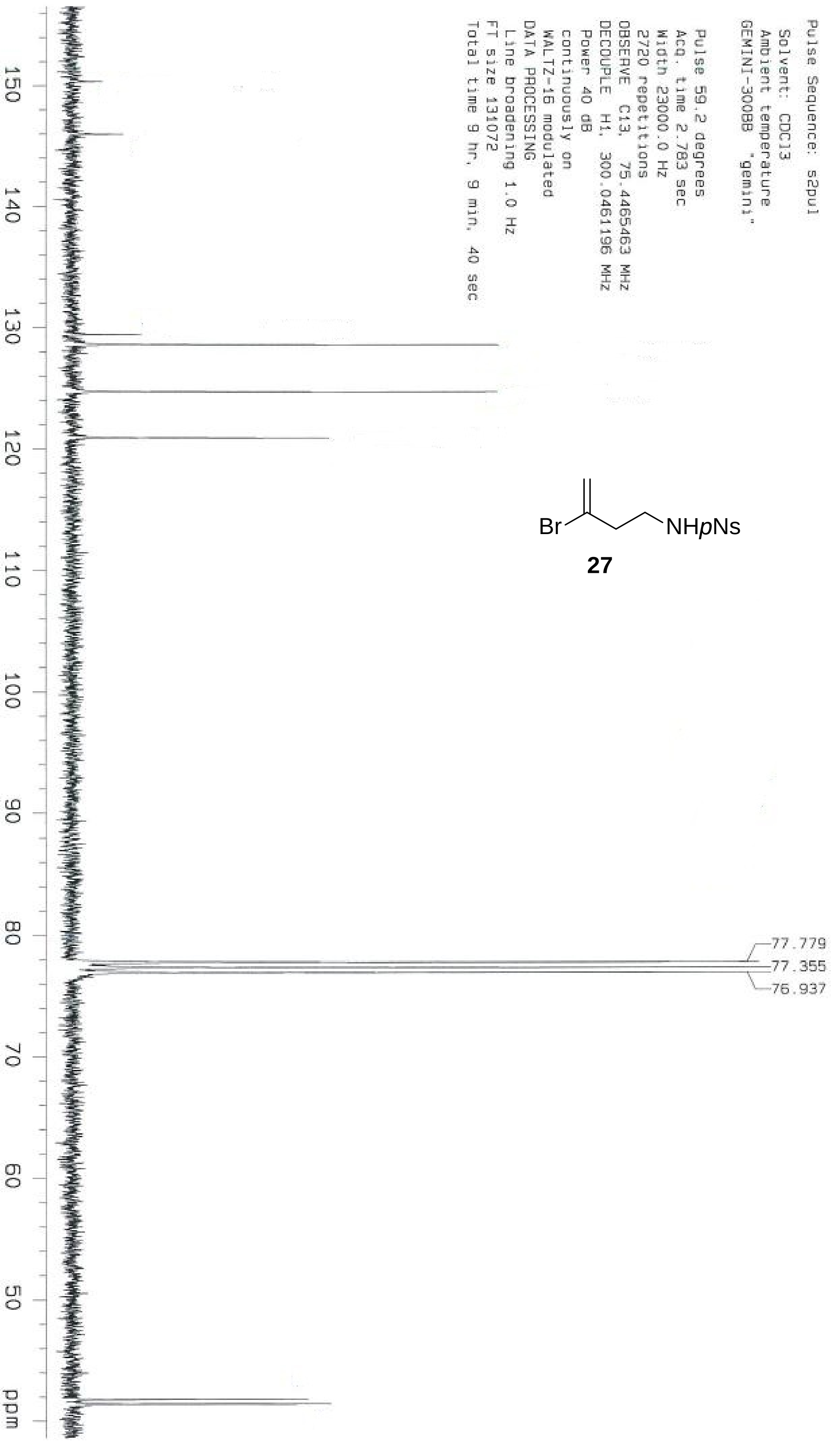
Pulse Sequence: s2pul
Solvent: CDCl3
Ambient temperature
GEMINI-300BB "gemini"
Pulse 59.2 degrees
Acq. time 2.783 sec
Width 23000.0 Hz
2720 repetitions
OBSERVE C13, 75.4465463 MHz
DECOUPLE H1, 300.0461196 MHz
Power 40 dB
continuously on
WALTZ-16 modulated
DATA PROCESSING
Line broadening 1.0 Hz
FT size 131072
Total time 9 hr, 9 min, 40 sec
Br
NHpNs
27
77.779
77.355
76.937
150
140
130
120
110
100
90
80
70
60
50
ppm

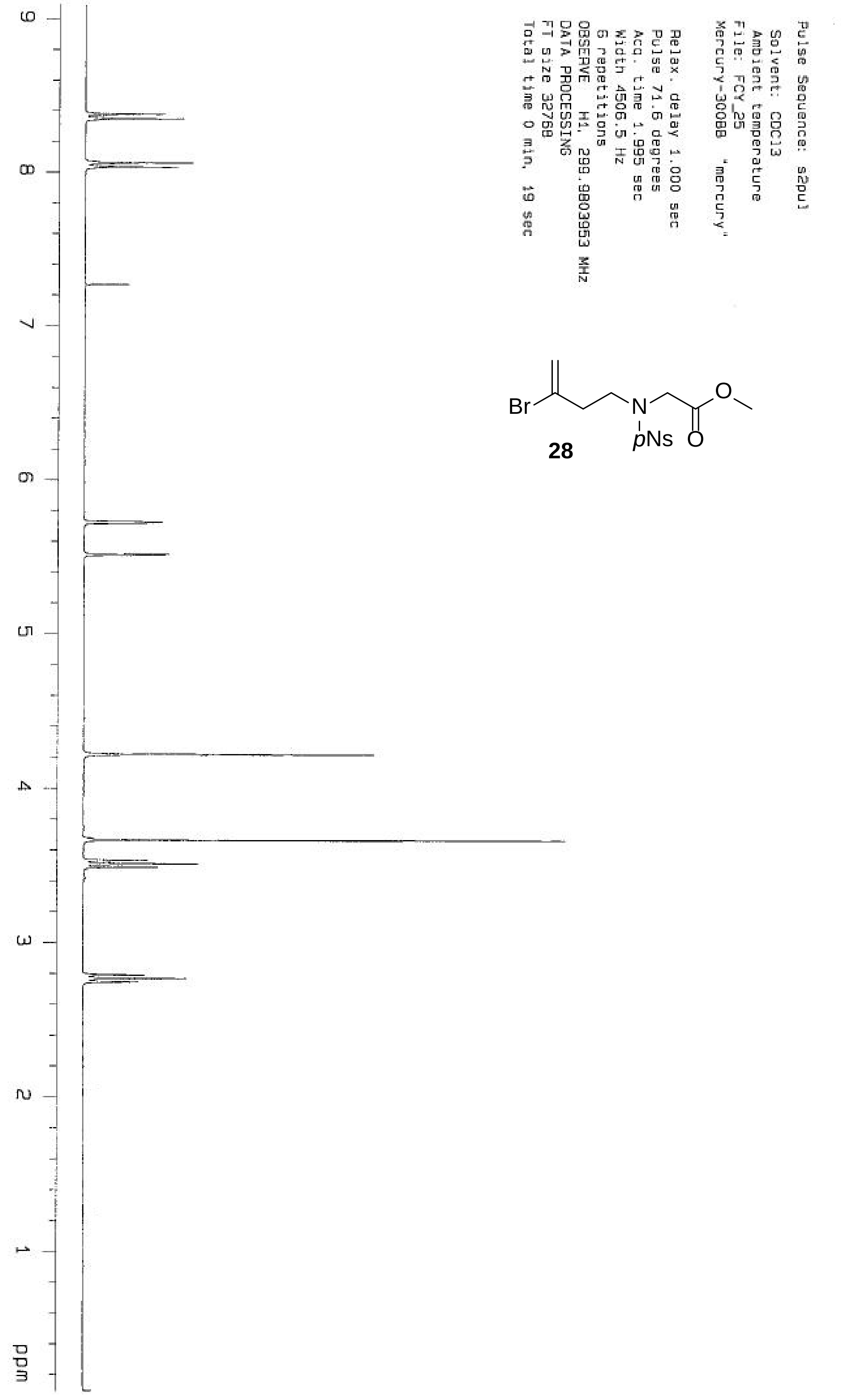
Pulse Sequence: s2pul
Solvent: CDCl3
Ambient temperature
File: FCY_25
Mercury-300BB "mercury"
Relax. delay 1.000 sec
Pulse 71.6 degrees
Acq. time 1.995 sec
Width 4506.5 Hz
6 repetitions
OBSERVE H1, 299.9803953 MHz
DATA PROCESSING
FT size 32768
Total time 0 min, 19 sec
Br
N
pNs
O
O
28
9
8
7
6
5
4
3
2
1
ppm

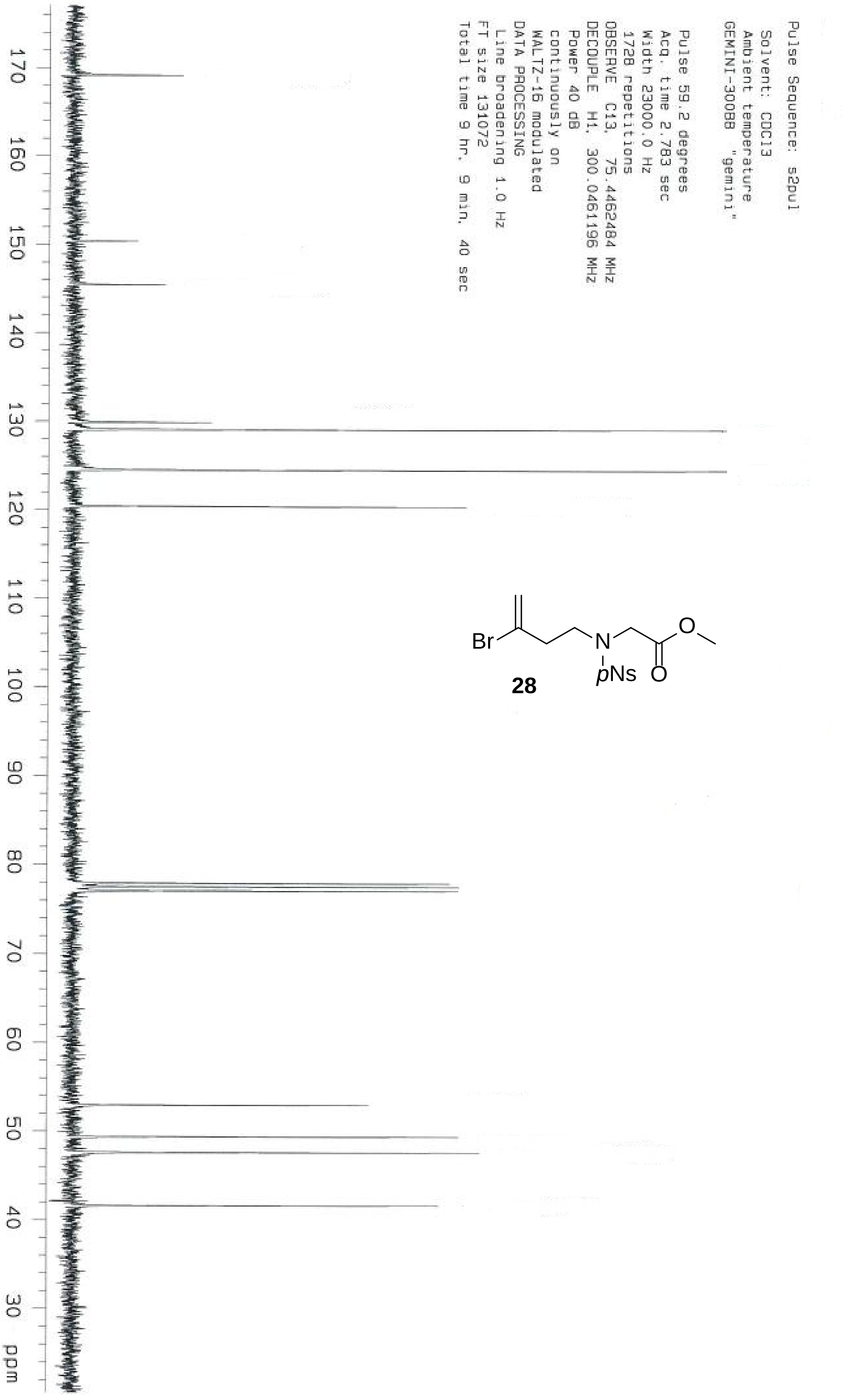

Pulse Sequence: s2pul
Solvent: CDCl3
Ambient temperature
GEMINI-300BB "gemini"
Pulse 59.2 degrees
Acq. time 2.783 sec
Width 23000.0 Hz
1728 repetitions
OBSERVE C13, 75.4462484 MHz
DECOUPLE H1, 300.0461196 MHz
Power 40 dB
continuously on
WALTZ-16 modulated
DATA PROCESSING
Line broadening 1.0 Hz
FT size 131072
Total time 9 hr, 9 min, 40 sec
Br
N
pNs
O
O
28
170
160
150
140
130
120
110
100
90
80
70
60
50
40
30
ppm

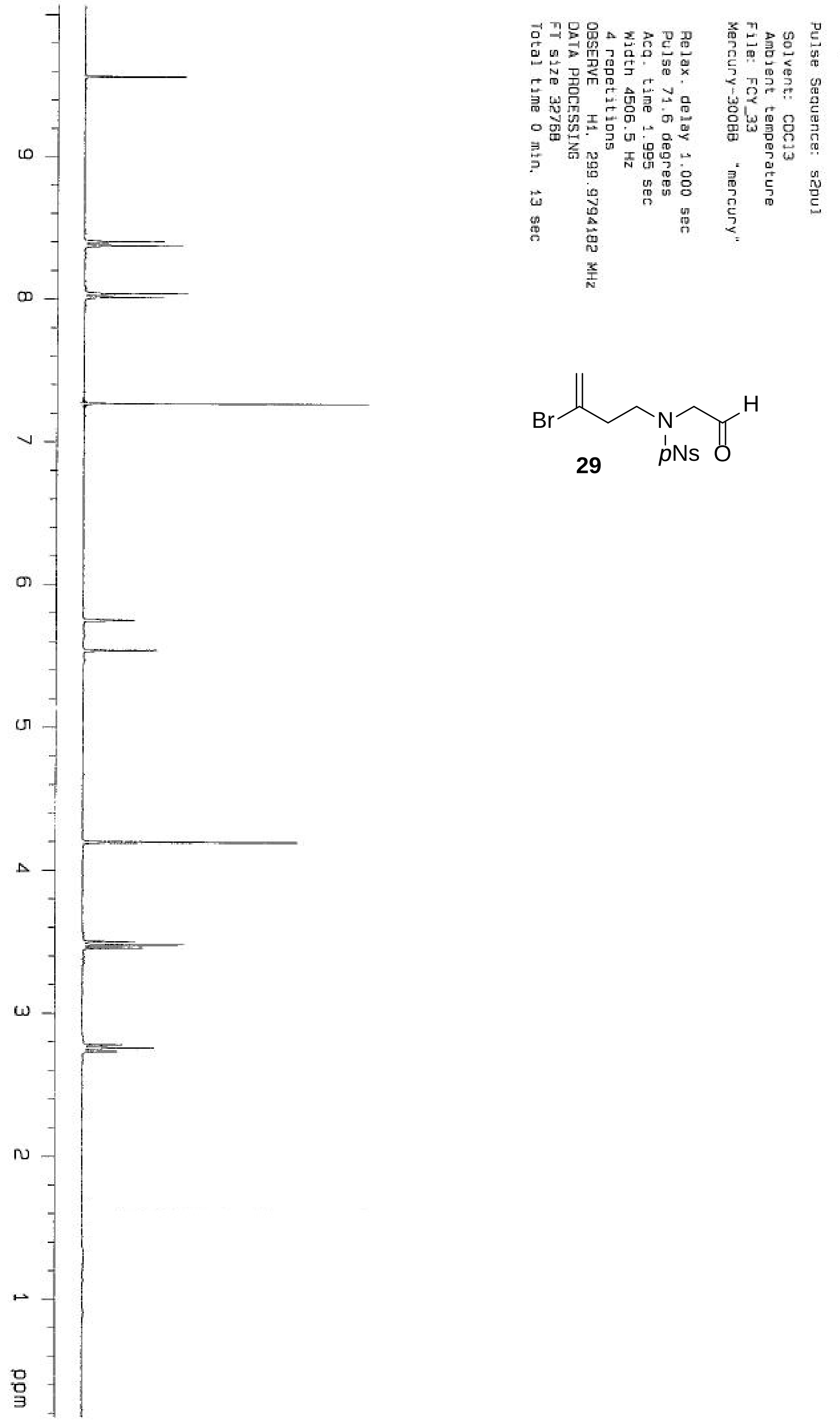
Pulse Sequence: s2pul
Solvent: CDCl3
Ambient temperature
File: FCY_33
Mercury-300BB "mercury"
Relax. delay 1.000 sec
Pulse 71.6 degrees
Acq. time 1.995 sec
Width 4506.5 Hz
4 repetitions
OBSERVE H1, 299.9794182 MHz
DATA PROCESSING
FT size 32768
Total time 0 min, 13 sec
Br
N
pNs
H
O
29
9
8
7
6
5
4
3
2
1
ppm

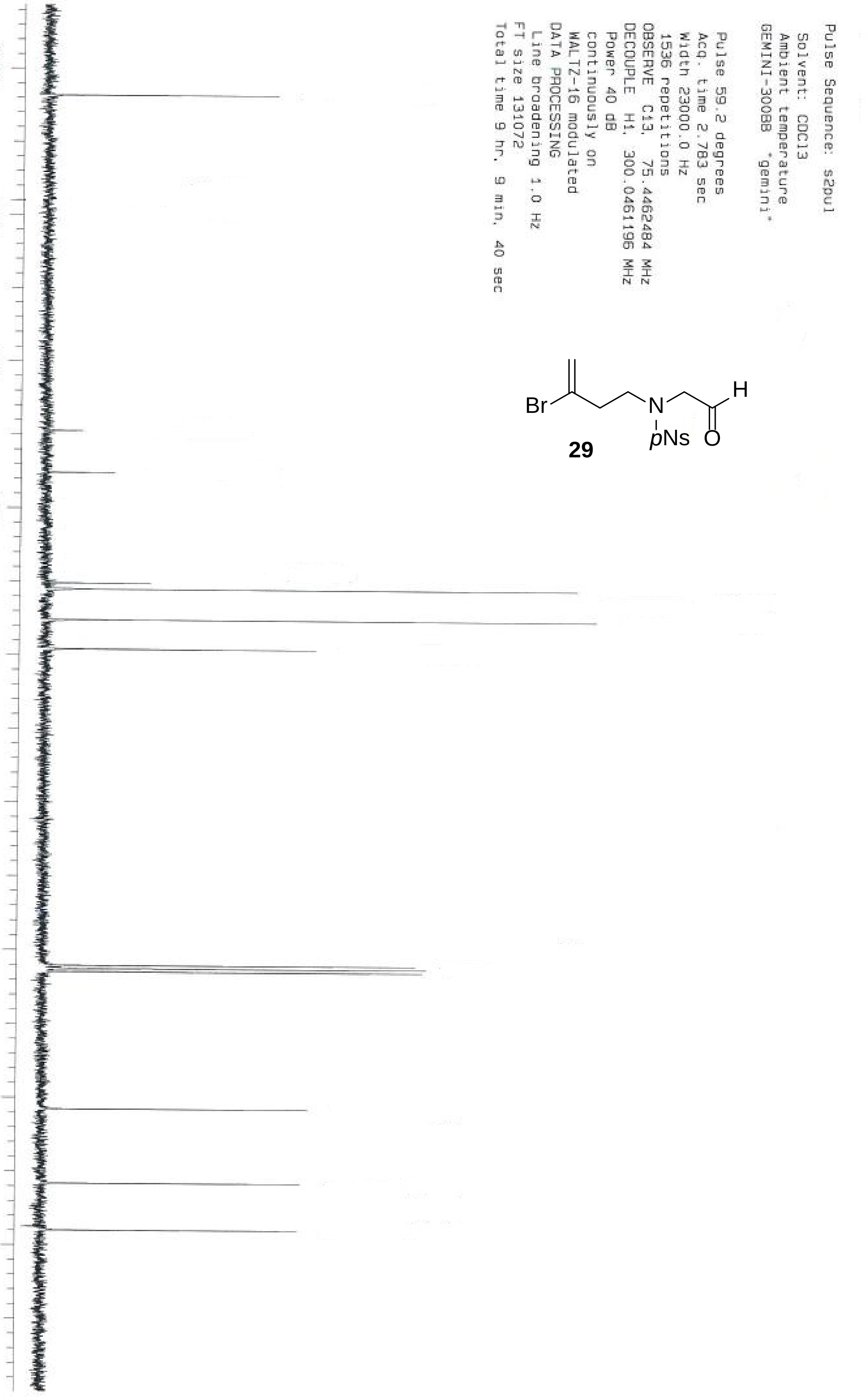
Pulse Sequence: s2pul
Solvent: CDCl3
Ambient temperature
GEMINI-300BB "gemini"
Pulse 59.2 degrees
Acq. time 2.783 sec
Width 23000.0 Hz
1536 repetitions
OBSERVE C13, 75.4462484 MHz
DECOUPLE H1, 300.0461196 MHz
Power 40 dB
continuously on
WALTZ-16 modulated
DATA PROCESSING
Line broadening 1.0 Hz
FT size 131072
Total time 9 hr, 9 min, 40 sec
Br
N
H
pNs
O
29
200
180
160
140
120
100
80
60
40
ppm

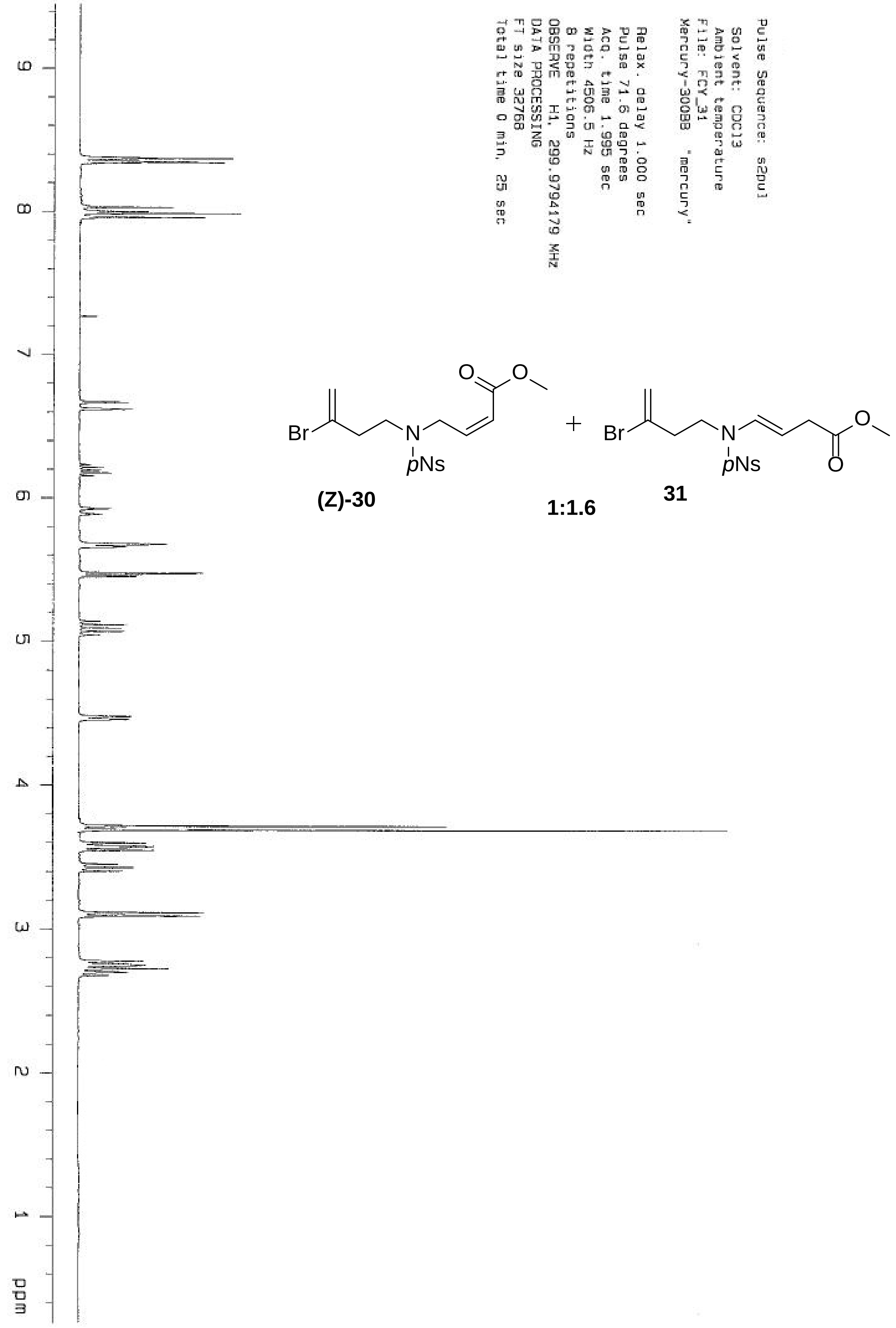
Pulse Sequence: s2pul
Solvent: CDCl3
Ambient temperature
File: FCY_31
Mercury-300BB "mercury"
Relax. delay 1.000 sec
Pulse 71.6 degrees
Acq. time 1.995 sec
Width 4506.5 Hz
8 repetitions
OBSERVE H1, 299.9794179 MHz
DATA PROCESSING
FT size 32768
Total time 0 min, 25 sec
Br
N
pNs
O
(Z)-30
+
1:1.6
31
9
8
7
6
5
4
3
2
1
ppm

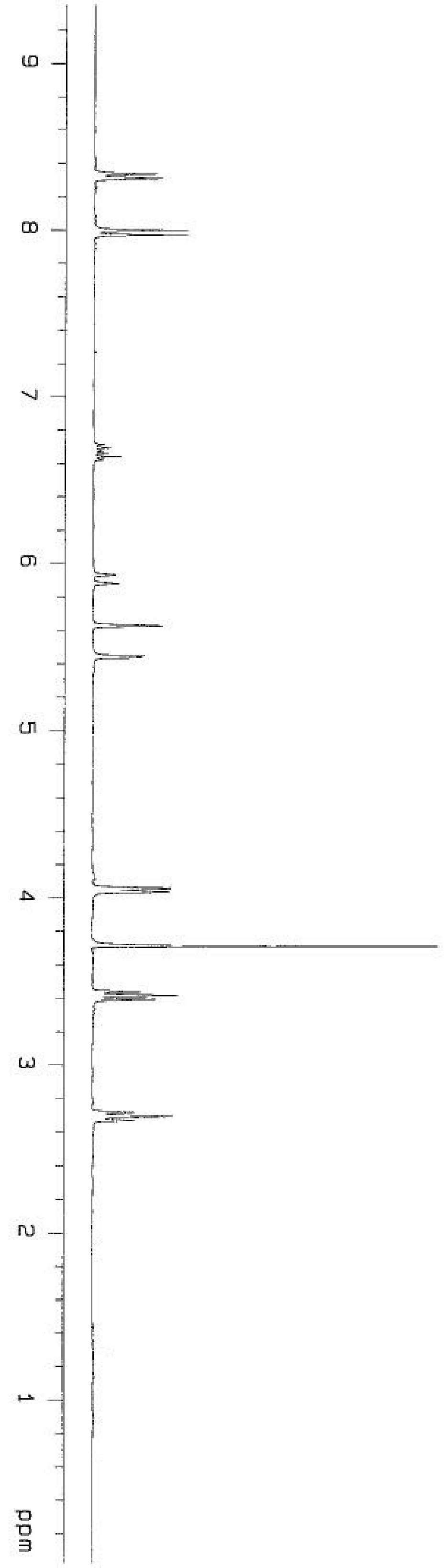
Pulse Sequence: s2pul
Solvent: CDCl3
Ambient temperature
File: FCY_53
Mercury-300BB "mercury"
Relax. delay 1.000 sec
Pulse 71.6 degrees
Acq. time 0.640 sec
Width 100.0 kHz
6 repetitions
OBSERVE H1, 299.9806863 MHz
DATA PROCESSING
FT size 131072
Total time 0 min, 17 sec
9
8
7
6
5
4
3
2
1
ppm

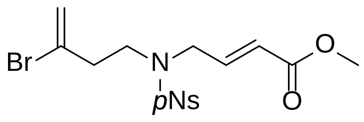
Br
N
pNs
O
O

***(E)*-30**

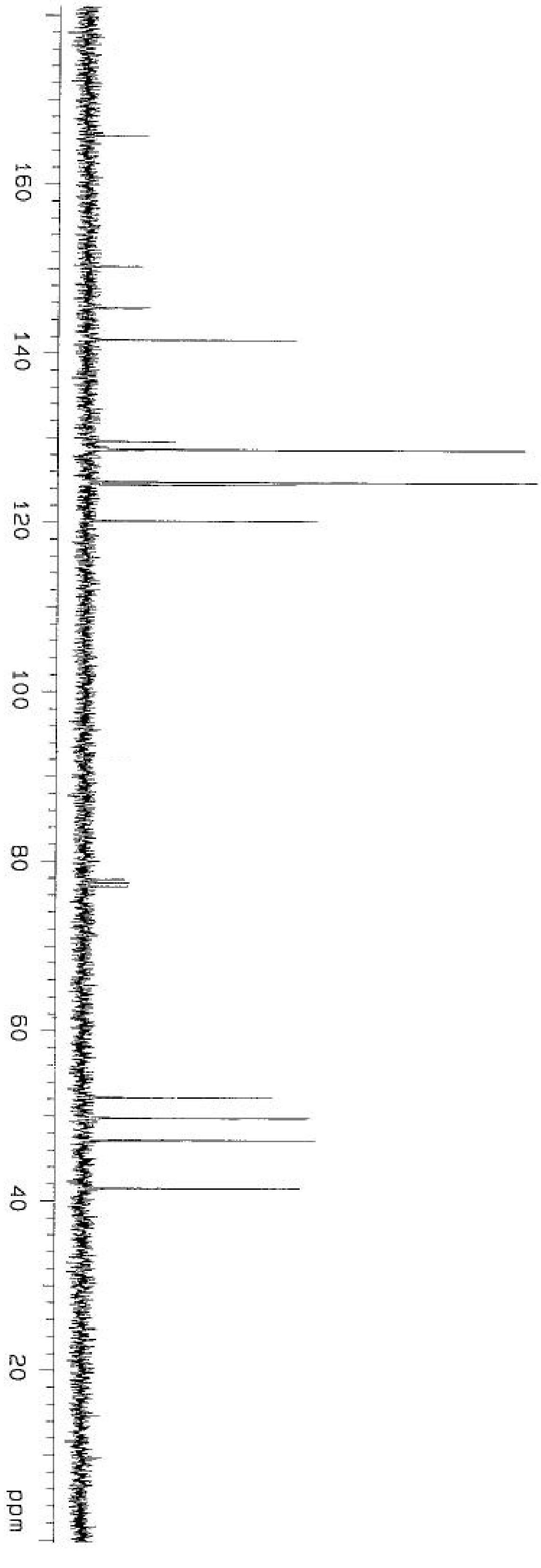

Pulse Sequence: s2pul
Solvent: CDCl3
Ambient temperature
File: FCY_53_C13
Mercury-300BB "mercury"
Pulse 55.7 degrees
Acq. time 1.815 sec
Width 18761.7 Hz
192 repetitions
OBSERVE C13, 75.4298680 MHz
DECOUPLE H1, 299.9808873 MHz
Power 35 dB
continuously on
WALTZ-16 modulated
DATA PROCESSING
Line broadening 1.0 Hz
FT size 131072
Total time 5 hr. 52 min. 1 sec
160
140
120
100
80
60
40
20
ppm

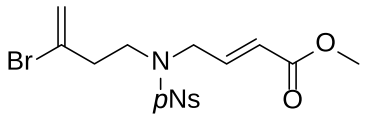

Br
N
pNs
O
O

***(E)*-30**

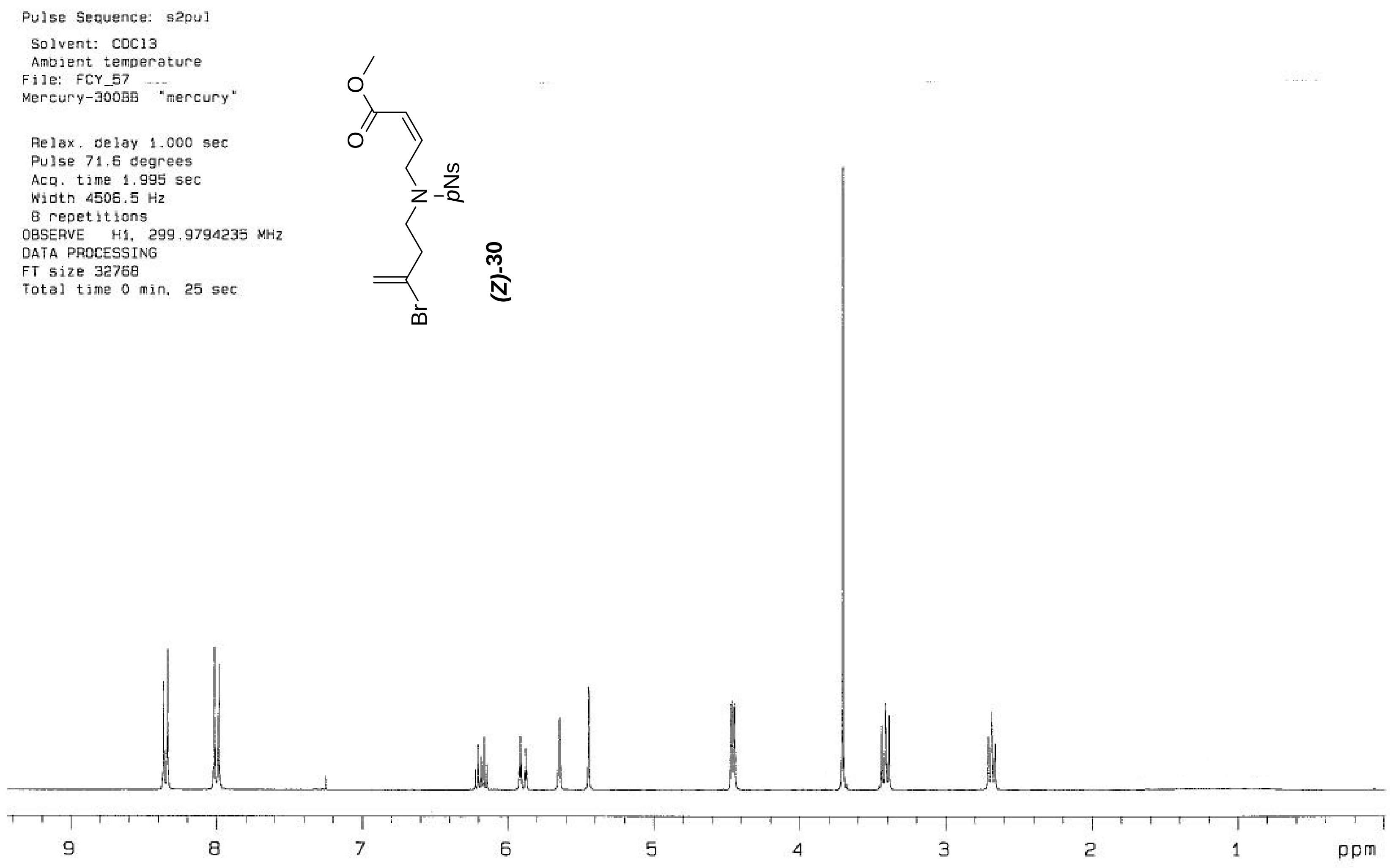
Pulse Sequence: s2pul
Solvent: CDCl3
Ambient temperature
File: FCY_57
Mercury-300BB "mercury"
Relax. delay 1.000 sec
Pulse 71.6 degrees
Acq. time 1.995 sec
Width 4506.5 Hz
8 repetitions
OBSERVE H1, 299.9794235 MHz
DATA PROCESSING
FT size 32768
Total time 0 min, 25 sec
O
O
N
pNs
Br
9
8
7
6
5
4
3
2
1
ppm

***(Z)*-30**

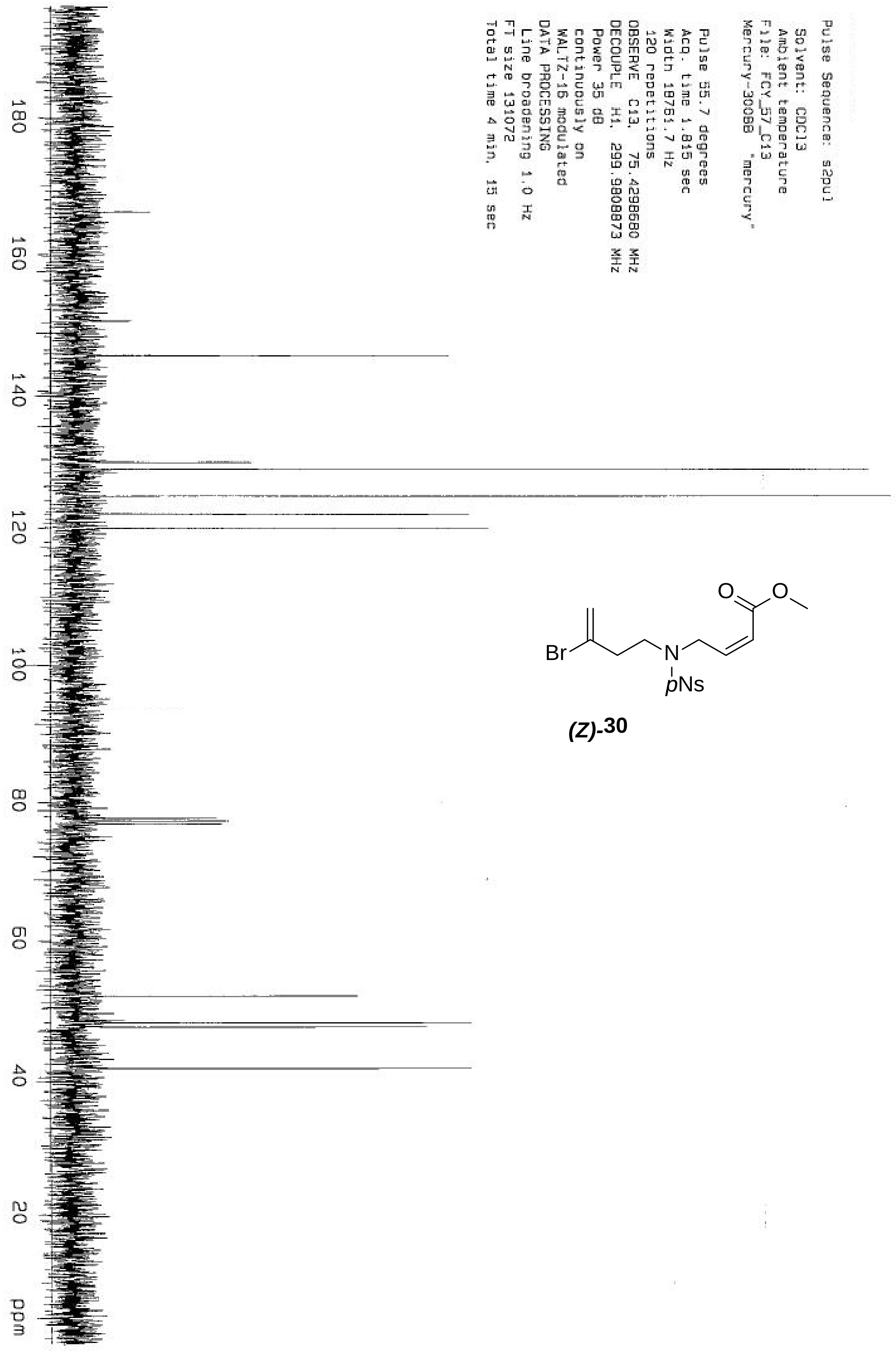
Pulse Sequence: s2pul
Solvent: CDCl3
Ambient temperature
File: FCY_57_C13
Mercury-300BB "mercury"
Pulse 55.7 degrees
Acq. time 1.815 sec
Width 18761.7 Hz
120 repetitions
OBSERVE C13, 75.4298680 MHz
DECOUPLE H1, 299.9808873 MHz
Power 35 dB
continuously on
WALTZ-16 modulated
DATA PROCESSING
Line broadening 1.0 Hz
FT size 131072
Total time 4 min, 15 sec
180
160
140
120
100
80
60
40
20
ppm
Br
N
pNs
O
O

(Z)-30

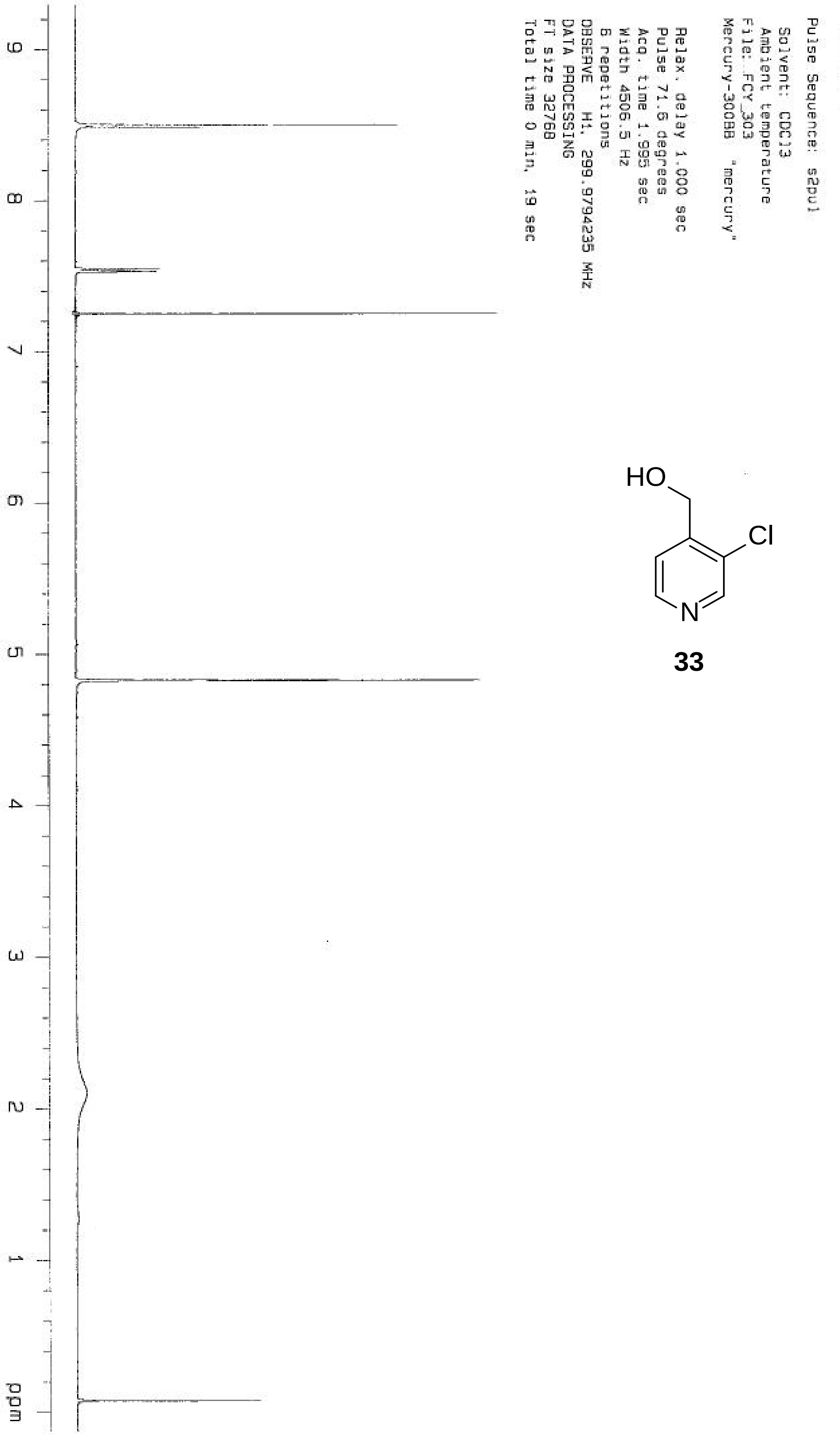
Pulse Sequence: s2pul
Solvent: CDCl3
Ambient temperature
File: FCY_303
Mercury-300BB "mercury"
Relax. delay 1.000 sec
Pulse 71.6 degrees
Acq. time 1.995 sec
Width 4506.5 Hz
6 repetitions
OBSERVE H1, 299.9794235 MHz
DATA PROCESSING
FT size 32768
Total time 0 min, 19 sec
9
8
7
6
5
4
3
2
1
ppm
HO
Cl
N

33

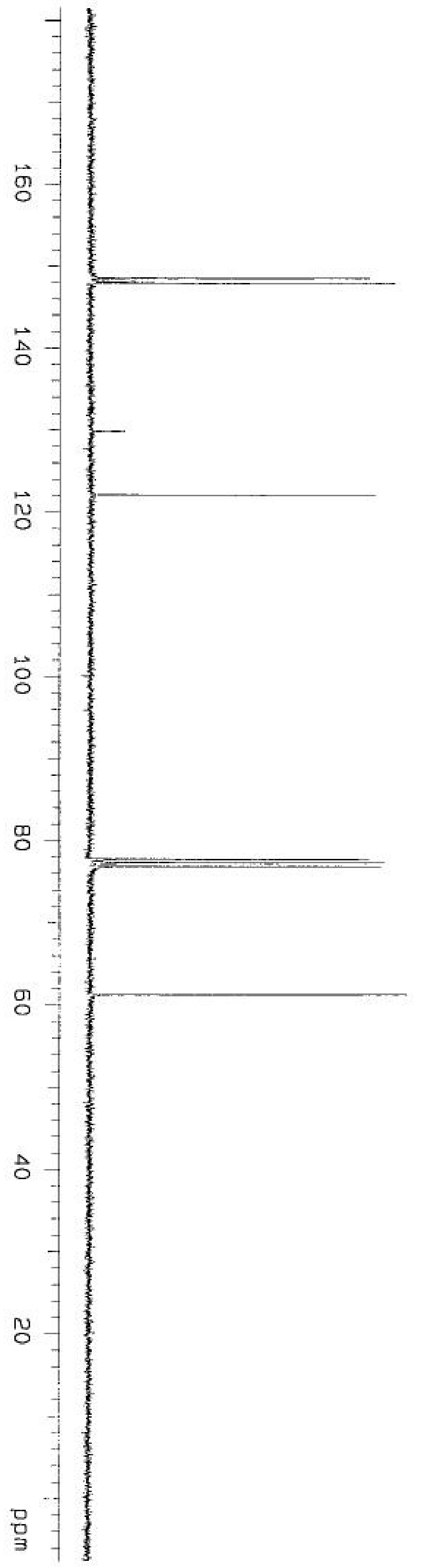
Pulse Sequence: s2pul
Solvent: CDCl3
Ambient temperature
File: FCY_303_C13
Mercury-300BB "mercury"
Pulse 55.7 degrees
Acq. time 1.815 sec
Width 18761.7 Hz
28496 repetitions
OBSERVE C13, 75.4298680 MHz
DECOUPLE H1, 299.9808873 MHz
Power 35 dB
continuously on
WALTZ-16 modulated
DATA PROCESSING
Line broadening 1.0 Hz
FT size 131072
Total time 23 hr, 28 min, 4 sec
160
140
120
100
80
60
40
20
ppm

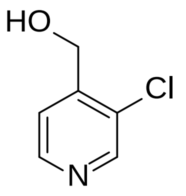
HO
Cl
N

33

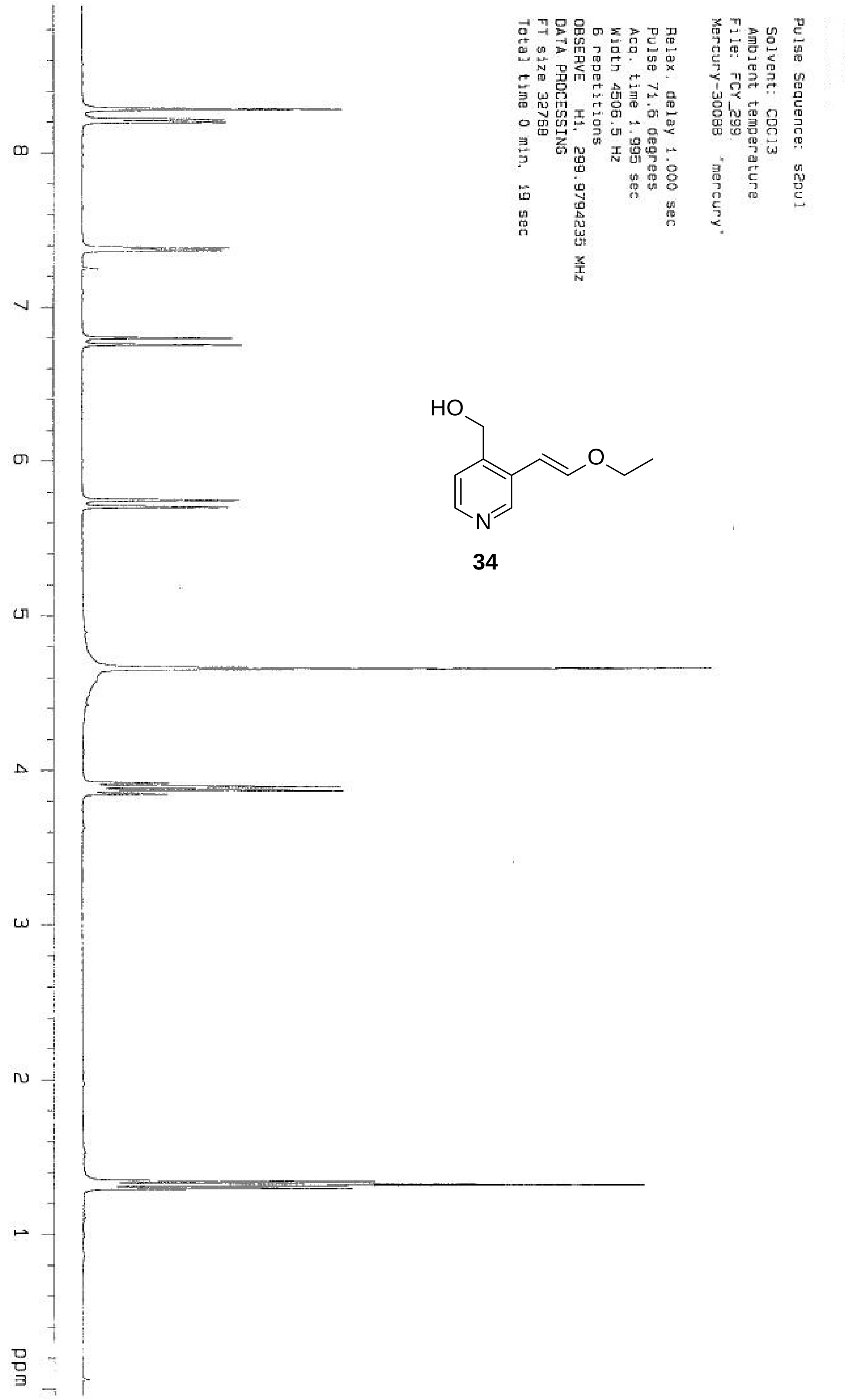
Pulse Sequence: s2pul
Solvent: CDCl3
Ambient temperature
File: FCY_299
Mercury-300BB "mercury"
Relax. delay 1.000 sec
Pulse 71.6 degrees
Acq. time 1.995 sec
Width 4506.5 Hz
6 repetitions
OBSERVE H1, 299.9794235 MHz
DATA PROCESSING
FT size 32768
Total time 0 min, 19 sec
HO
O
N
34
8
7
6
5
4
3
2
1
ppm

Pulse Sequence: s2pul

Solvent: CDCl3
Ambient temperature
File: FCY_299_C13
Mercury-300BB "mercury"

Pulse 55.7 degrees
Acq. time 1.815 sec
Width 18761.7 Hz
144 repetitions
OBSERVE C13, 75.4298680 MHz
DECOUPLE H1, 299.9808873 MHz
Power 35 dB
continuously on
WALTZ-16 modulated
DATA PROCESSING
Line broadening 1.0 Hz
FT size 131072
Total time 11 hr, 44 min, 2 sec

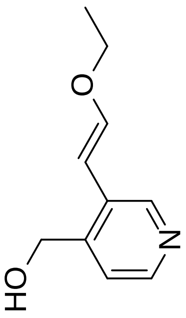

34

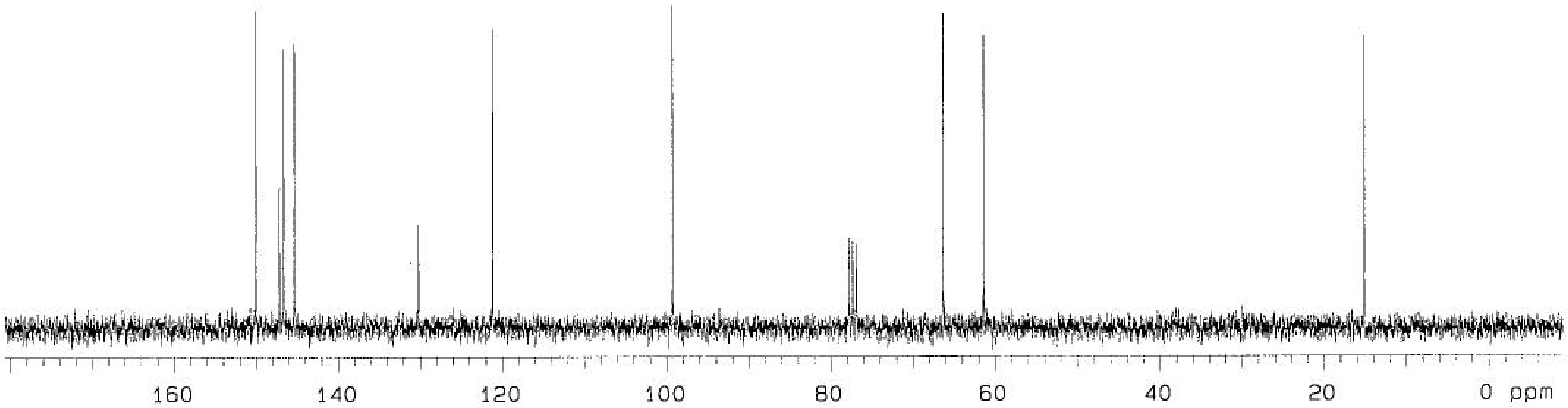

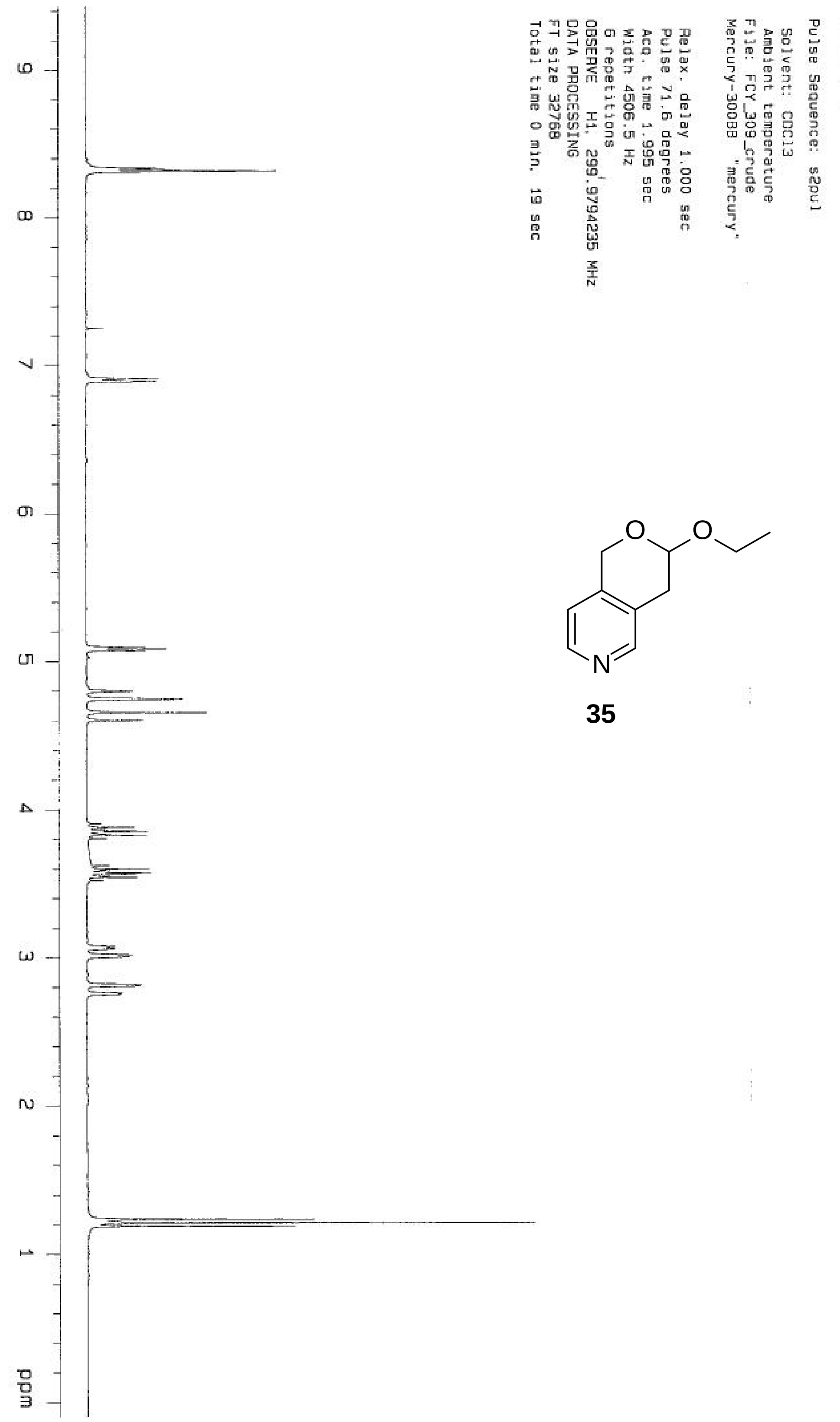
Pulse Sequence: s2pul
Solvent: CDCl3
Ambient temperature
File: FCY_309_crude
Mercury-300BB "mercury"
Relax. delay 1.000 sec
Pulse 71.6 degrees
Acq. time 1.995 sec
Width 4506.5 Hz
6 repetitions
OBSERVE H1, 299.9794235 MHz
DATA PROCESSING
FT size 32768
Total time 0 min, 19 sec
O
O
N
35
9
8
7
6
5
4
3
2
1
ppm

Pulse Sequence: s2pul

Solvent: CDCl3
Ambient temperature
GEMINI-300BB "gemini"

Pulse 59.2 degrees
Acq. time 2.783 sec
Width 23000.0 Hz
1056 repetitions
OBSERVE C13, 75.4446103 MHz
DECOUPLE H1, 300.0395342 MHz
Power 40 dB
continuously on
WALTZ-16 modulated
DATA PROCESSING
Line broadening 1.0 Hz
FT size 131072
Total time 18 hr, 19 min, 20 sec

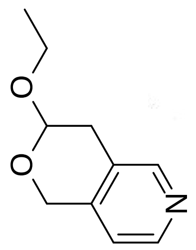

35

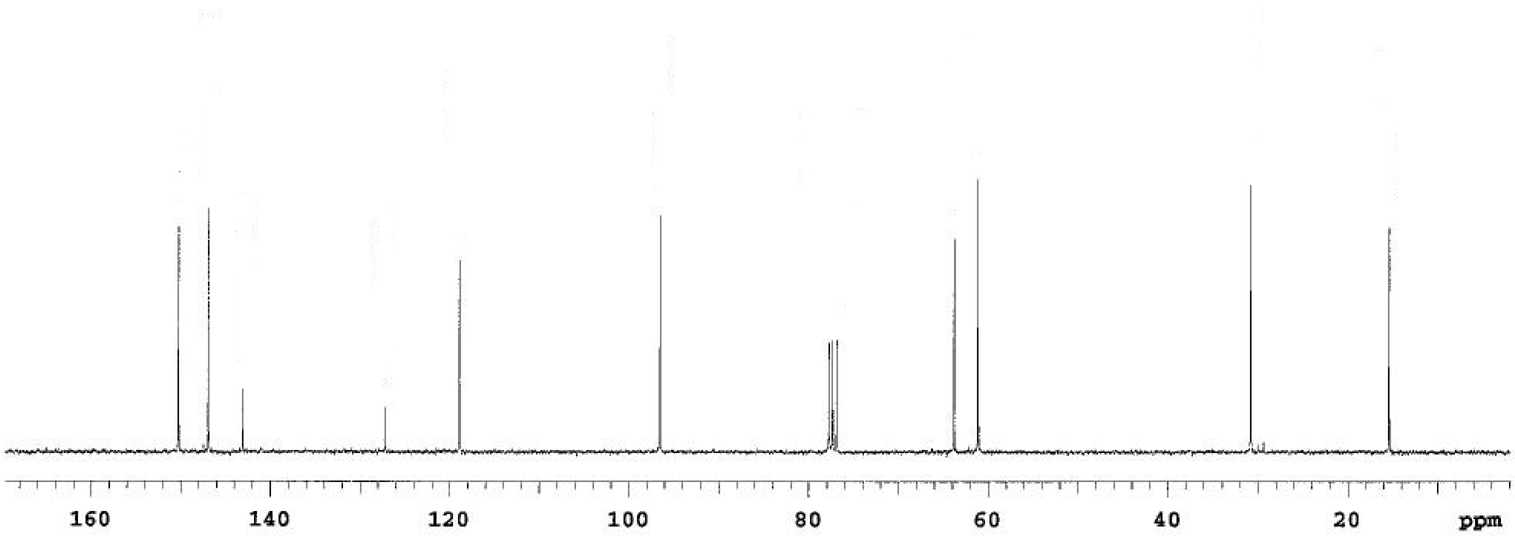

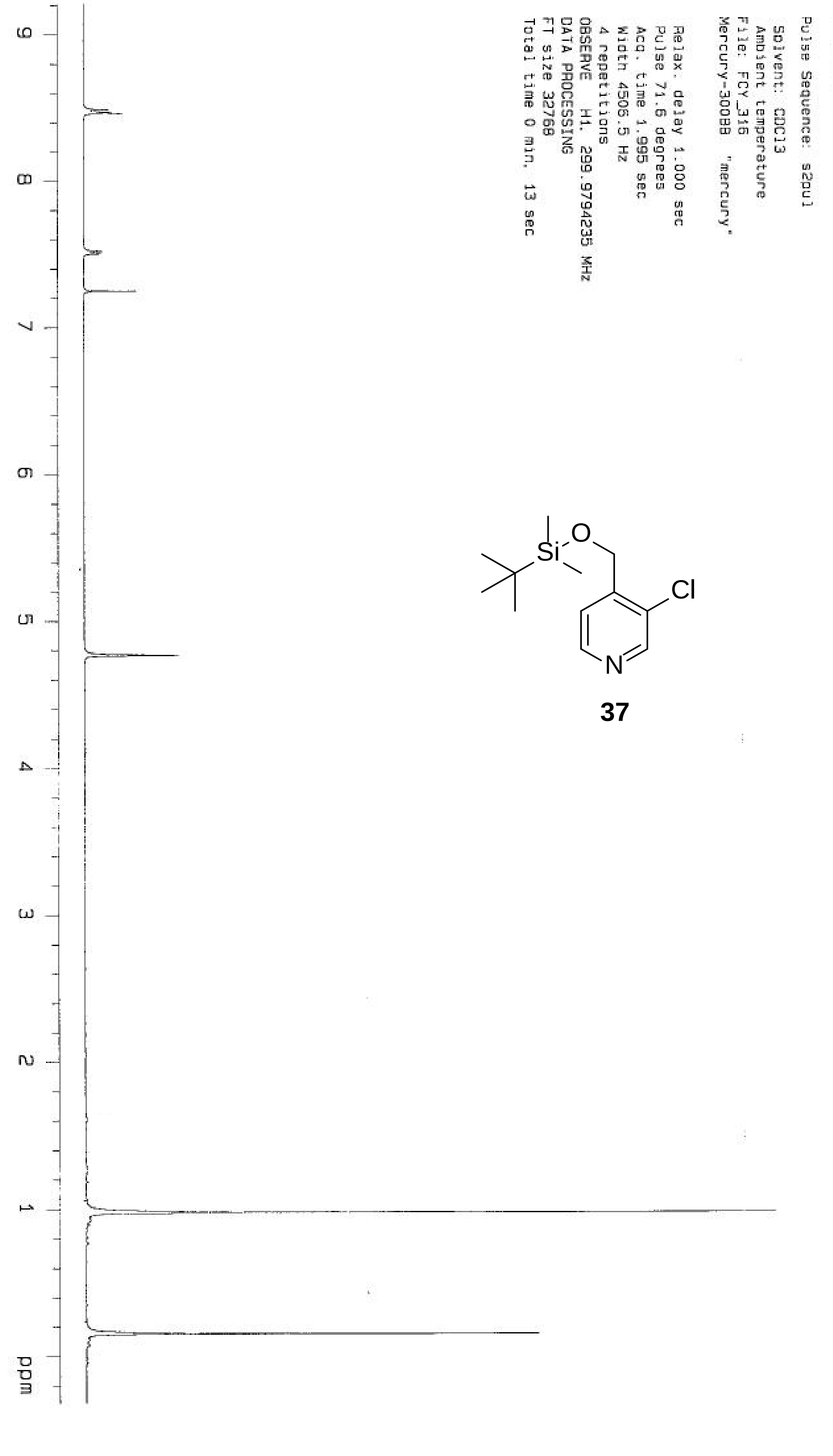
Pulse Sequence: s2pul
Solvent: CDCl3
Ambient temperature
File: FCY_316
Mercury-300BB "mercury"
Relax. delay 1.000 sec
Pulse 71.6 degrees
Acq. time 1.995 sec
Width 4506.5 Hz
4 repetitions
OBSERVE H1, 299.9794235 MHz
DATA PROCESSING
FT size 32768
Total time 0 min, 13 sec
Si
O
Cl
N
37
9
8
7
6
5
4
3
2
1
ppm

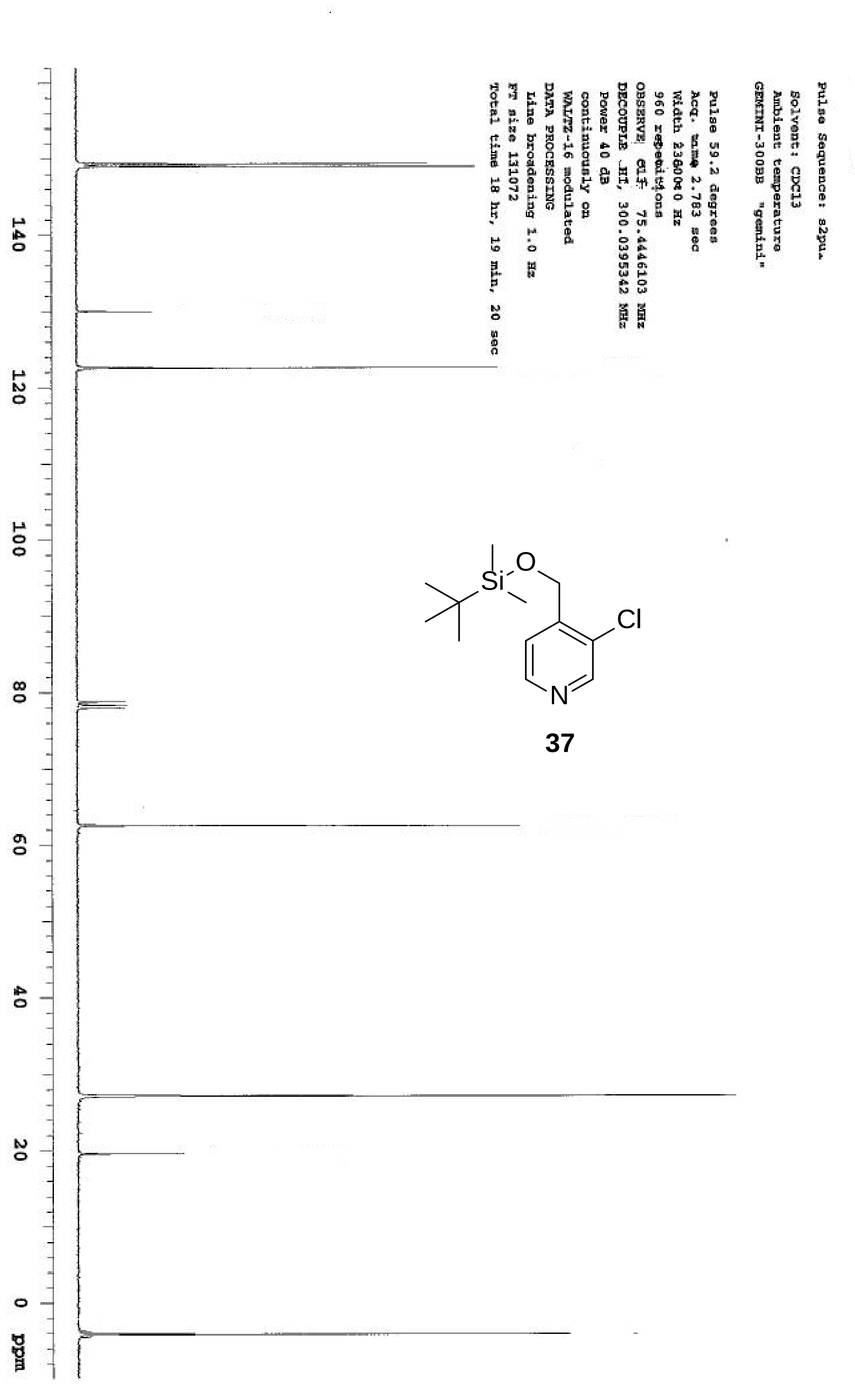
Pulse Sequence: s2pul
Solvent: CDCl3
Ambient temperature
GEMINI-300BB "gemini"
Pulse 59.2 degrees
DATA PROCESSING
Line broadening 1.0 Hz
FT size 131072
Total time 18 hr, 19 min, 20 sec
Power 40 dB
continuously on
WALTZ-16 modulated
140
120
100
80
60
40
20
0
ppm
Si
O
Cl
N

37

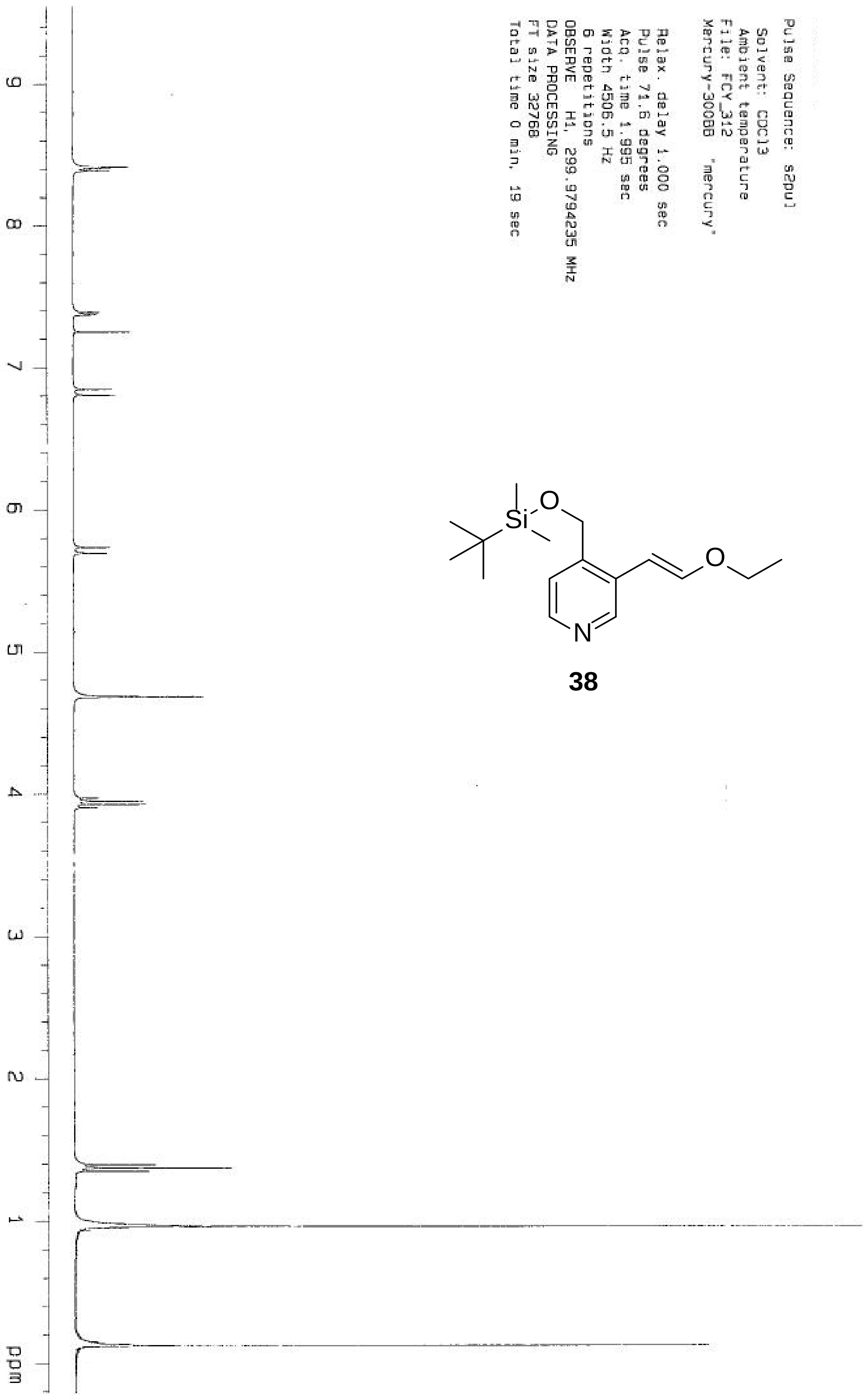
Pulse Sequence: s2pul
Solvent: CDCl3
Ambient temperature
File: FCY_312
Mercury-300BB "mercury"
Relax. delay 1.000 sec
Pulse 71.6 degrees
Acq. time 1.995 sec
Width 4506.5 Hz
6 repetitions
OBSERVE H1, 299.9794235 MHz
DATA PROCESSING
FT size 32768
Total time 0 min, 19 sec
9
8
7
6
5
4
3
2
1
ppm
Si
O
O
N

38

Pulse Sequence: s2pul

Solvent: CDCl3
Ambient temperature
File: FCY_312_C13
Mercury-300BB "mercury"

Pulse 55.7 degrees
Acq. time 1.815 sec
Width 18761.7 Hz
400 repetitions
OBSERVE C13, 75.4298680 MHz
DECOUPLE H1, 299.9808873 MHz
Power 35 dB
continuously on
WALTZ-16 modulated
DATA PROCESSING
Line broadening 1.0 Hz
FT size 131072
Total time 117 hr, 20 min, 24 sec

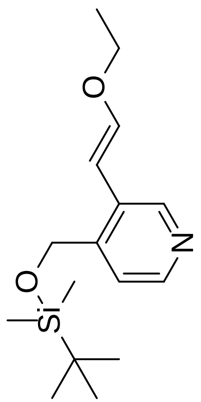

38

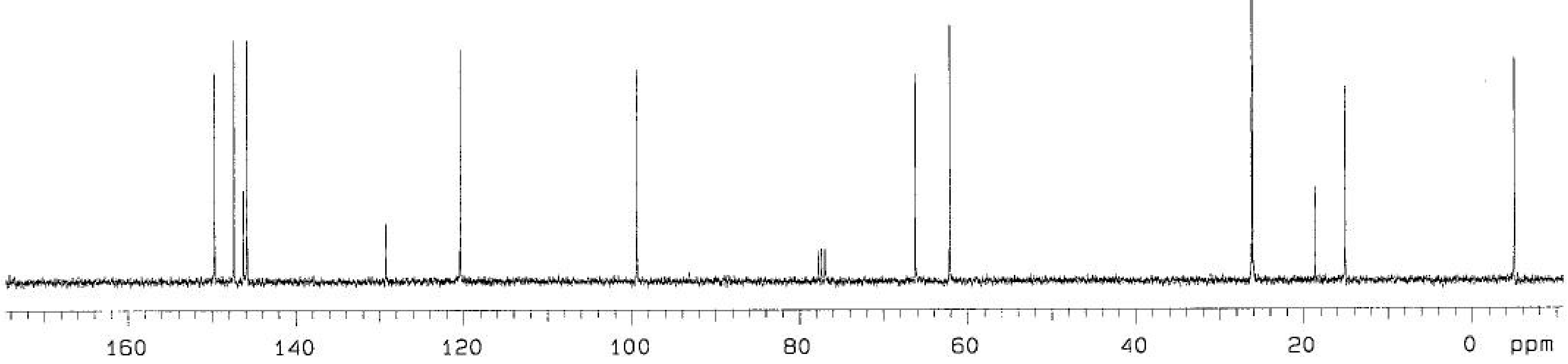

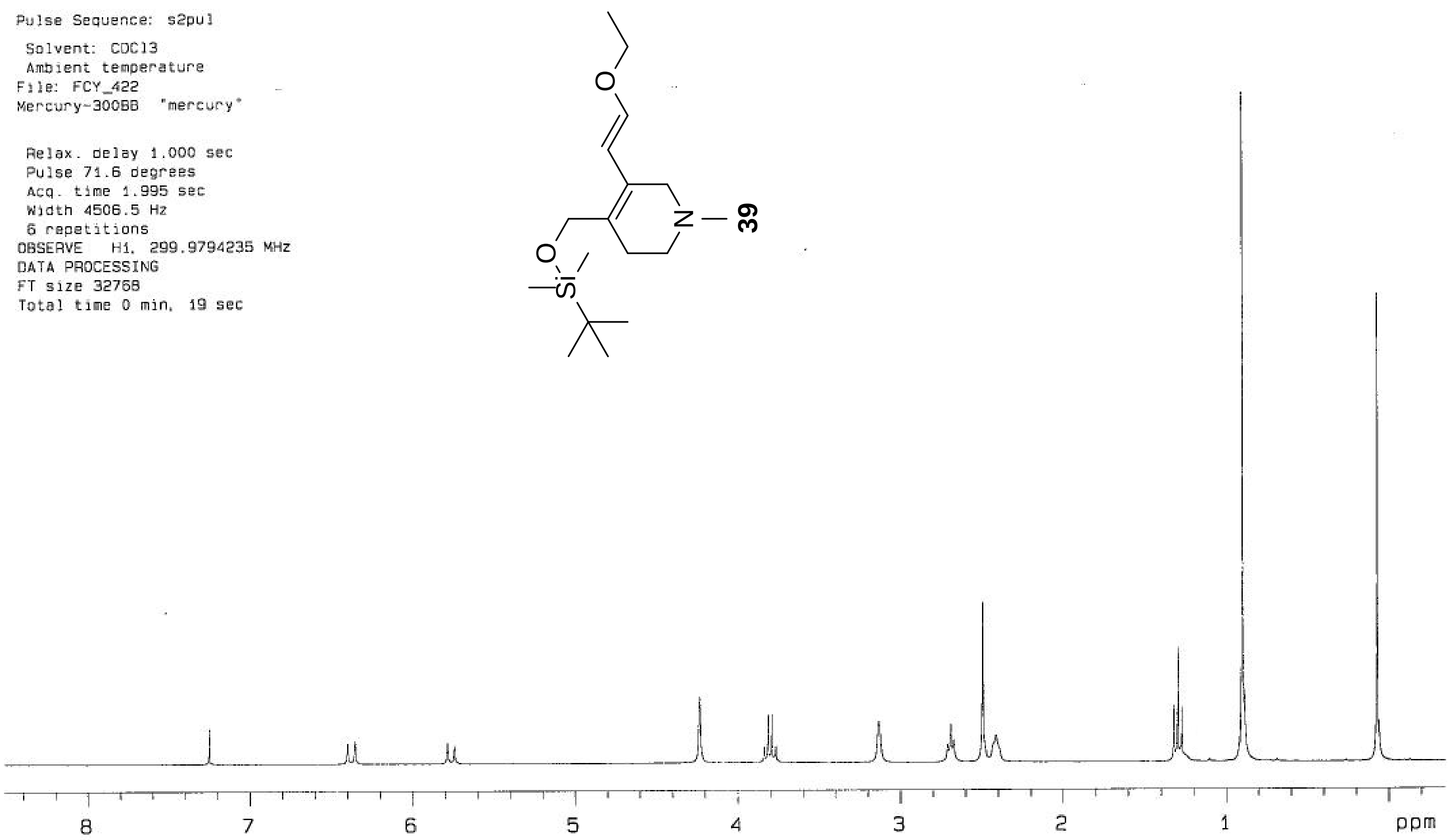
Pulse Sequence: s2pul
Solvent: CDCl3
Ambient temperature
File: FCY_422
Mercury-300BB "mercury"
Relax. delay 1.000 sec
Pulse 71.6 degrees
Acq. time 1.995 sec
Width 4506.5 Hz
6 repetitions
OBSERVE H1, 299.9794235 MHz
DATA PROCESSING
FT size 32768
Total time 0 min, 19 sec
O
O
Si
N
39
8
7
6
5
4
3
2
1
ppm

Pulse Sequence: s2pul

Solvent: CDCl3
Ambient temperature
File: FCY_422_C13
Mercury-300BB "mercury"

Pulse 55.7 degrees
Acq. time 1.815 sec
Width 18761.7 Hz
28960 repetitions
OBSERVE C13, 75.4298680 MHz
DECOUPLE H1, 299.9808873 MHz
Power 35 dB
continuously on
WALTZ-16 modulated
DATA PROCESSING
Line broadening 1.0 Hz
FT size 131072
Total time 117 hr, 20 min, 24 sec

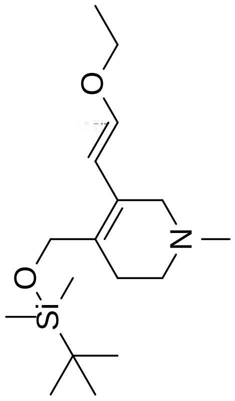

39

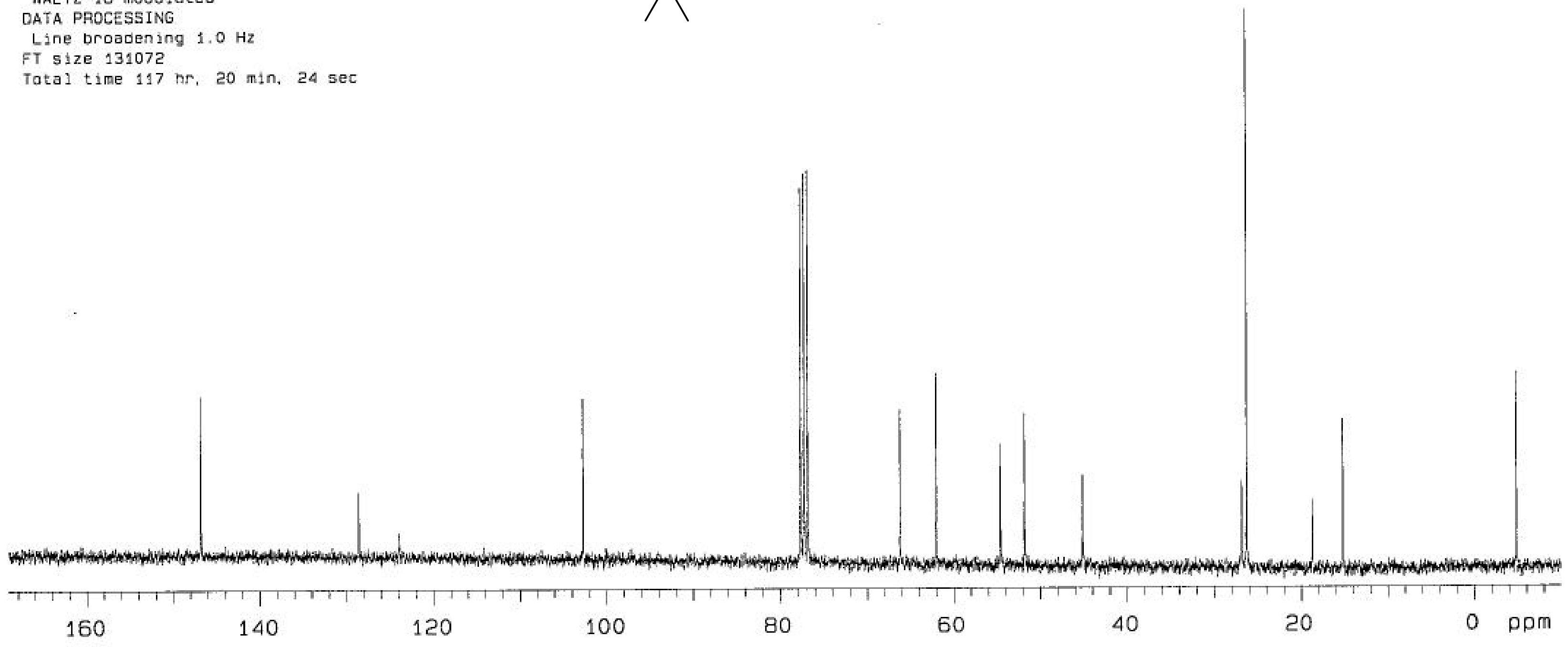

Supplement: File 2 — Copies of NMR spectra. [file Beilstein_J_Org_Chem-13-988-s002.pdf]
